# Extensive Evolutionary Changes in Regulatory Element Activity during Human Origins Are Associated with Altered Gene Expression and Positive Selection

**DOI:** 10.1371/journal.pgen.1002789

**Published:** 2012-06-28

**Authors:** Yoichiro Shibata, Nathan C. Sheffield, Olivier Fedrigo, Courtney C. Babbitt, Matthew Wortham, Alok K. Tewari, Darin London, Lingyun Song, Bum-Kyu Lee, Vishwanath R. Iyer, Stephen C. J. Parker, Elliott H. Margulies, Gregory A. Wray, Terrence S. Furey, Gregory E. Crawford

**Affiliations:** 1 Institute for Genome Sciences and Policy, Duke University, Durham, North Carolina, United States of America; 2 Biology Department, Duke University, Durham, North Carolina, United States of America; 3 Center for Systems and Synthetic Biology, Institute for Cellular and Molecular Biology, Section of Molecular Genetics and Microbiology, University of Texas at Austin, Austin, Texas, United States of America; 4 Genome Technology Branch, National Human Genome Research Institute, National Institutes of Health, Bethesda, Maryland, United States of America; 5 Department of Genetics, Carolina Center for Genome Sciences, The University of North Carolina at Chapel Hill, Chapel Hill, North Carolina, United States of America; 6 Department of Biology, Carolina Center for Genome Sciences, and Lineberger Comprehensive Cancer Center, The University of North Carolina at Chapel Hill, Chapel Hill, North Carolina, United States of America; 7 Department of Pediatrics, Division of Medical Genetics, Duke University, Durham, North Carolina, United States of America; University of Washington, United States of America

## Abstract

Understanding the molecular basis for phenotypic differences between humans and other primates remains an outstanding challenge. Mutations in non-coding regulatory DNA that alter gene expression have been hypothesized as a key driver of these phenotypic differences. This has been supported by differential gene expression analyses in general, but not by the identification of specific regulatory elements responsible for changes in transcription and phenotype. To identify the genetic source of regulatory differences, we mapped DNaseI hypersensitive (DHS) sites, which mark all types of active gene regulatory elements, genome-wide in the same cell type isolated from human, chimpanzee, and macaque. Most DHS sites were conserved among all three species, as expected based on their central role in regulating transcription. However, we found evidence that several hundred DHS sites were gained or lost on the lineages leading to modern human and chimpanzee. Species-specific DHS site gains are enriched near differentially expressed genes, are positively correlated with increased transcription, show evidence of branch-specific positive selection, and overlap with active chromatin marks. Species-specific sequence differences in transcription factor motifs found within these DHS sites are linked with species-specific changes in chromatin accessibility. Together, these indicate that the regulatory elements identified here are genetic contributors to transcriptional and phenotypic differences among primate species.

## Author Summary

The human genome shares a remarkable amount of genomic sequence with our closest living primate relatives. Researchers have long sought to understand what regions of the genome are responsible for unique species-specific traits. Previous studies have shown that many genes are differentially expressed between species, but the regulatory elements contributing to these differences are largely unknown. Here we report a genome-wide comparison of active gene regulatory elements in human, chimpanzee, and macaque, and we identify hundreds of regulatory elements that have been gained or lost in the human or chimpanzee genomes since their evolutionary divergence. These elements contain evidence of natural selection and correlate with species-specific changes in gene expression. Polymorphic DNA bases in transcription factor motifs that we found in these regulatory elements may be responsible for the varied biological functions across species. This study directly links phenotypic and transcriptional differences between species with changes in chromatin structure.

## Introduction

Understanding the molecular basis of phenotypic differences between humans and other primates has been a priority in medicine, behavior, and evolution research [1]–[3]. The genetic basis for these differences can now be explored genome-wide due in part to the rising number of completely sequenced primate genomes. However, finding genotype-phenotype connections is difficult since the vast majority of sequence changes do not contribute to phenotypic differences across species. It was hypothesized over 40 years ago that phenotypic differences between humans and our closest primate relatives are shaped largely by changes in non-coding regulatory elements [4]. Variation in gene regulation have been indirectly confirmed by studying gene expression differences across matched cell or tissue types isolated from different primates [5]–[12], but these studies have failed to pinpoint the regulatory elements responsible for these changes [13]. Genome-wide scans of non-coding DNA sequences under branch-specific positive selection have identified putative regulatory elements that have undergone functional changes [14]–[16]. These studies identified hundreds of regulatory regions with evidence of accelerated sequence substitution during human origins, possibly reflecting adaptive changes in gene regulation. Scans for selection do not, however, provide information about the functional or trait consequences of these evolutionary changes.

**Figure 1:**
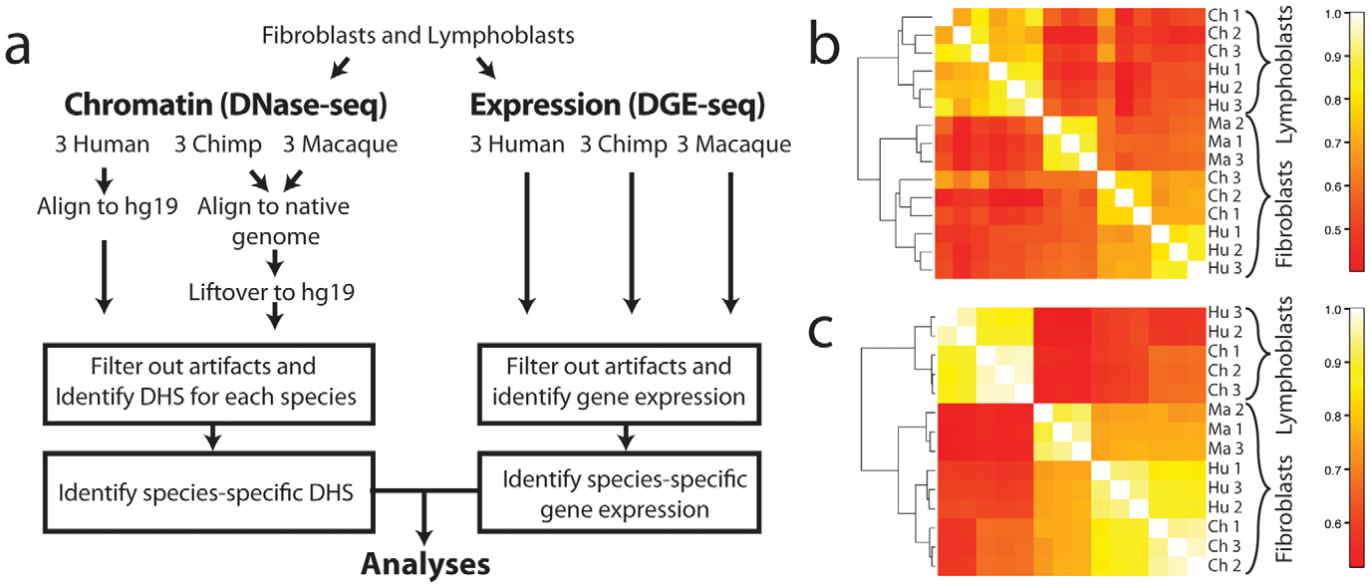
Comparison of DHS sites and DGE-seq data across species. (a) Analysis pipeline. DNase-sequences from each species were aligned to the native genome and lifted over to the human genome for analysis. Regions are filtered at various steps of the analysis to remove alignment and orthology artifacts (Materials and Materials). Correlation plots of DNase-seq signals (b) and DGE-seq signals (c) expression data show that both chromatin and expression data from human (Hu), chimpanzee (Ch), and macaque (Ma) are more highly correlated between biological replicates from the same tissue within a single species. Additionally, the same cell type from different species is more similar than different cell types from the same species.

Understanding the relationship between mutation, natural selection, and variation in gene regulation is an important goal in evolutionary genomics. Heritable differences in gene expression must have a genetic basis, but exactly what sequence variants have led to these differences are largely unknown. In this study, we used changes in chromatin configuration to better understand this genotype-phenotype relationship. We identified evolutionary conserved and altered regulatory element activity by performing genome-wide DNase-seq [17], [18] in primary skin fibroblasts and lymphoblastoid cell lines (LCLs) isolated from three human and three chimpanzee individuals (Figure 1a and Table S1). Each DNase-seq experiment identifies nucleosome-depleted DNaseI hypersensitive (DHS) sites that mark all types of regulatory elements, including promoters, enhancers, silencers, insulators, and locus control regions. The comprehensiveness of this assay is supported by ChIP experiments for active histone marks, p300, CTCF, and other transcription factors [19]–[21]. In addition to human and chimpanzee, we performed DNase-seq on fibroblasts from three Rhesus macaque individuals to polarize human-chimpanzee chromatin differences and to distinguish between gains and losses of regulatory elements on the human and chimpanzee branches (EBV-derived lymphoblastoid cells are not available for this species). We also performed Digital Gene Expression sequence (DGE-seq) experiments using the same cell cultures to simultaneously compare levels of mRNA abundance [7], [22]. Analyses of these data provide insights into the relationship between evolutionary changes in regulatory elements, their tissue-specific activity, and the resulting functional consequences in gene expression.

## Results

### DNase-seq identifies species-specific DHS sites

To directly compare DNase-seq data generated from human and non-human primate fibroblast and lymphoblastoid cell line (LCL) samples, we mapped all data to the human genome (build hg19). Non-human DNase-seq sequences were first aligned to their native primate genome and then converted to human coordinates using liftOver [23] (Figure 1a). We limited analyses to high confidence orthologous regions of the human, chimp, and macaque genomes to eliminate potential artifacts due to mis-aligned, missing sequence, or CNVs (Materials and Methods). Comparisons across individuals within a species and against tiling array DNase-chip [24], [25] data generated from the same material supported data accuracy and reproducibility (Materials and Methods and Table S2). DNase-seq signals from individuals within a species were more highly correlated than signals from different species (Figure 1b). Human and chimpanzee DNase-seq signals from fibroblasts were better correlated than human and macaque signals as expected since human and chimp share a more recent common ancestor. Chromatin structure differed more in cell types (fibroblasts vs. LCLs) from the same species than in the same cell-type across different species (Figure 1b). For example, human and chimpanzee fibroblast DNase-seq signals are more similar than human fibroblast and human LCL DNase-seq signals. The same correlation patterns were also found in gene expression data generated from the same samples (Figure 1c).

**Figure 2:**
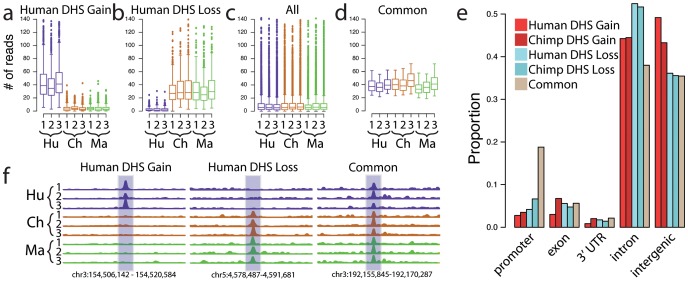
Identification of species-specific differences in DHS sites. Species-specific DHS sites were identified by edgeR (Materials and Methods). Boxplots show the distribution of number of reads per sample in 300 bp windows. For human DHS gains (a), the 3 human samples are all significantly more open than the other 2 species. Likewise, human DHS losses (b) show lower signal in human compared to both chimpanzee and macaque. A representative sampling of distributions from all DHS is shown in (c), as well as Common DHS sites (d) found in all three species that are matched for signal intensity compared to human DHS gains and human DHS losses. (e) Distribution of species-specific DHS Gains and DHS Losses relative to promoters, introns, 3′ UTR, and intergenic regions. (f) Representative screen shots of human-specific DHS Gains and Losses compared to a Common region.

**Figure 3:**
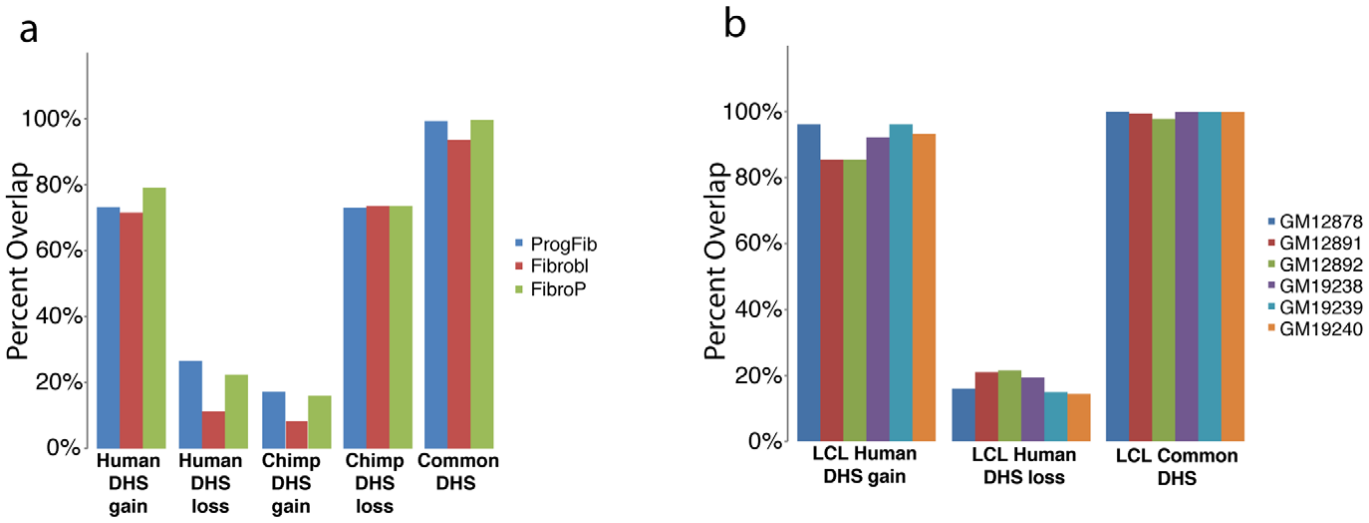
Comparison of species-specific DHS to independently derived cells. Human DHS gains show a high level of overlap to DHS regions identified in (a) three independently analyzed human fibroblast cell lines and (b) 5 independently analyzed human LCL samples, compared to human DHS losses. Common DHS are also similarly detected.

We identified genomic regions exhibiting significant differences in DNase-seq signal between species [26] (Materials and Methods). Data from macaque samples were used to classify regions as DHS gains or DHS losses on the human or chimpanzee branch (Materials and Methods). More specifically, we defined a human DHS gain as a region with significantly more DNase-seq signal in human than in either chimpanzee or macaque (Figure 2a), and a human DHS loss as a region with significantly less DNase-seq signal in human than in either chimpanzee or macaque (Figure 2b). In essence, these data identify regulatory regions that originated or disappeared in fibroblasts during human origins. Chimpanzee DHS gains and DHS losses were similarly defined (Figure S1). For approximately 90% of gains, a corresponding DHS site was completely absent in all three individuals from each of the other species (Figure 2f). For the remaining sites, DHS sites were annotated in multiple species, but a consistently higher DNase-seq signal was present in one species compared to the others (data not shown). We found that the majority of the human DHS gains (72–79%) and chimpanzee DHS losses (73–74%), and a minority of the human DHS losses (11–27%) and chimpanzee DHS gains (8–17%), overlapped a DHS site found in one or more of three independently derived human fibroblasts (Figure 3a, Table S3). We also found similar trends comparing six independently derived LCLs analyzed by our group (Figure 3b, Table S4), and 20 independently derived human fibroblast samples analyzed by another ENCODE group (Figure S2a-S2b) [27], [28]. These provide evidence that the identified DHS gains and losses represent significant and reproducible functional changes between species. Fibroblasts have been shown to have specific expression profiles associated with different biopsy locations [29], [30]. We note that DHS gains and losses are not enriched around these genes (Materials and Methods).

Identified DHS gains (Figure 2a) and losses (Figure 2b) deviated in sequence read depth from the general chromatin spectrum (Figure 2c). To more directly compare DHS gains and losses with sites that do not change between species, we also identified a set of DHS regions with similar DNase-seq signal intensity across all three species, which we call Common DHS regions (Figure 2d, Materials and Methods, Supplemental data file 1 in Dataset S1). Using a false discovery rate (FDR) of 1%, we detected 836 human DHS gains, 286 human DHS losses, 676 chimpanzee DHS gains, 211 chimpanzee DHS losses, and 1259 Common regions (Supplemental data file 1 in Dataset S1). The higher number of DHS gains compared to DHS losses could be due to purifying selection, or more simply may be related to the asymmetry in their detection criteria (see Materials and Methods for a more complete discussion). True species-specific DHS gains and losses could not be identified in LCLs due to the lack of macaque EBV-derived LCL samples. However, we identified 103 DHS sites with higher DNase-seq signals in human (LCL human DHS gain), 181 DHS sites with lower signals in human (LCL human DHS loss), and 1583 DHS sites with similar signals in both (LCL common DHS; Supplemental data file 1 in Dataset S1).

Similar numbers of gains and losses were found when comparing chimpanzee DNase-seq data to data from an independent set of human fibroblasts and LCLs at the same FDR (Figure S3a–S3b). Furthermore, only 66 differential open chromatin sites were detected when comparing human fibroblast data to additional independently derived human fibroblasts. Likewise, only 1 differential DHS site was detected when comparing human LCLs to additional independently derived human LCLs. This is less than 1% of all differential open chromatin sites when comparing human vs. chimpanzee, indicating a low false positive rate (Figure S3a–S3b).

### Species-specific DHS sites are cell type–specific

**Figure 4:**
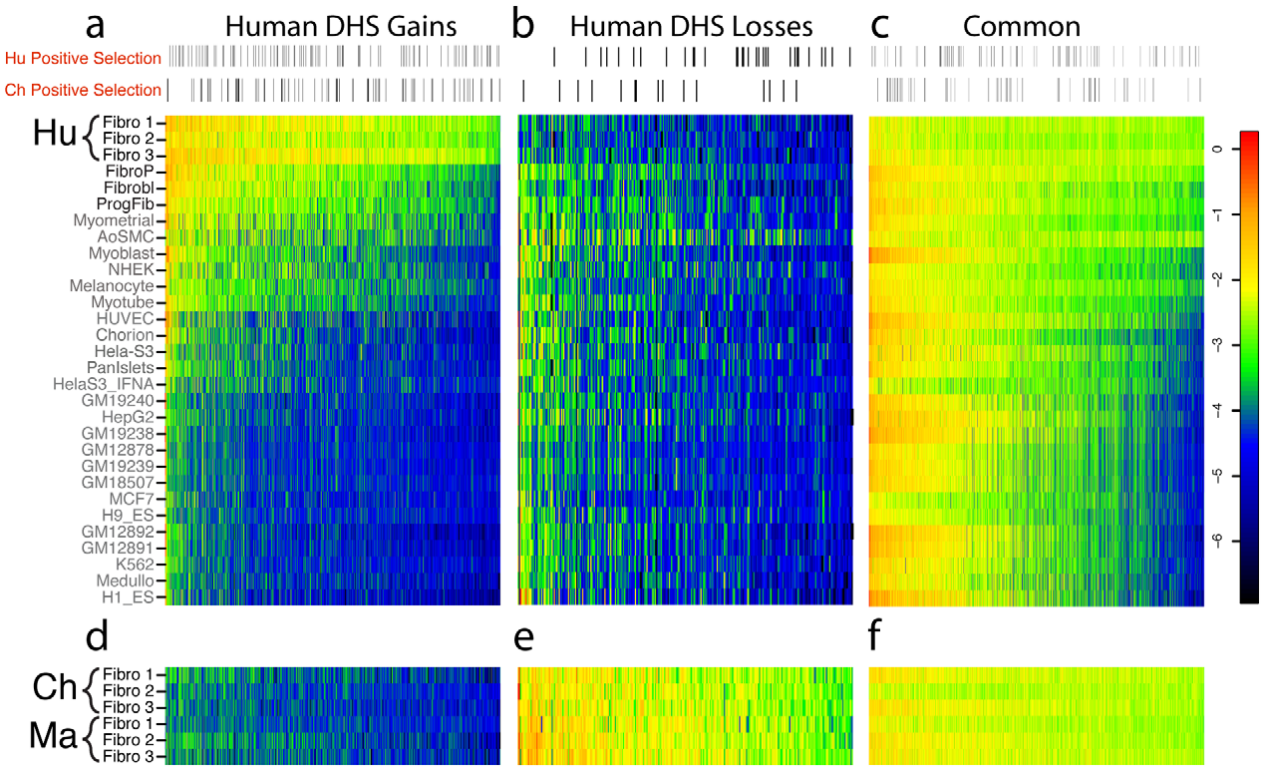
Comparison of human DHS site gains and losses to DNase-seq data from other human cell types. The log of the DNase-seq signal intensity value, defined as the maximum parzen score (output of F-seq) for each of the coordinates that are represented along the x-axis, are represented as a heatmap in these figures. The color red represents a higher score, and thus a relatively higher DNase-seq signal, and the color blue represents a lower score. (a) 836 DHS sites were identified as differentially open (human DHS gain) in human fibroblasts compared to chimpanzee/macaque fibroblasts. These regions from Human Fibroblasts (Hu Fibro 1–3) were compared to DNase-seq data generated from 27 other human cell types (Table S3). Additional human skin fibroblast samples (listed in black) are highly similar, while some non-fibroblast cell types show less but substantial overlap and the remaining cell types show much less overlap. Only a small fraction of DHS sites were active in all 27 cell lines (Figure S5). Sites with evidence for positive selection are indicated in the horizontal bar above the heatmap. The distribution appears roughly uniform. (b) 286 DHS sites identified as differentially closed (human DHS loss) compared to chimp and macaque fibroblasts. (c) DNase-seq signal values for Common regions representing DHS sites in all three species. More than 50% of Common regions are also DHS sites in other human tissues. (d, e, f) DNase-seq values for same regions as (a, b, c), but DNase data is from orthologous region from chimpanzee and macaque fibroblasts.

As part of the ENCyclopedia Of DNA Elements (ENCODE) project [31], we have generated DNase-seq data from 27 diverse human cell types [32] (Table S3, Crawford unpublished ENCODE data). We determined the overlap of our identified DHS gains and losses in fibroblasts with DHS sites in these other human cell types. Seven hundred and sixty-seven (92%) fibroblast human DHS gains were found in at least one of three other independently derived human skin fibroblast ENCODE cell lines from normal (Fibrobl) and diseased individuals (Parkinson's: FibroP; Progeria: ProgFib) supporting the reproducibility of these data (Figure 4a, Figure S3a). Additionally, human DHS gains showed a high level of overlap with some, but not all, non-fibroblast human cell types (Figure 4a, Table S4, Figures S4, S5). This suggests that DHS gains are largely cell-type specific. Few human DHS losses were identified as a DHS site in any of the other human cell types (Figure 4b and Figures S4, S5). In contrast, Common DHS sites were detected in most other human cell types (Figure 4c and Figures S4, S5) suggesting DHS sites active among all three primates have more general roles in regulating transcription. Similar trends were seen when comparing LCL human DHS gain/loss/common regions (Table S5). This suggests Common DHS sites mark DHS sites present in most or all non-human primate cell types, as can be seen for chimp lymphoblast DHS sites (Figure S6i). Expected chimp and macaque DNase signal intensity are detected in orthologous regions (Figure 4d–4f). Similar to previous analysis of cell-type specific DHS sites [32], we found species-specific gains and losses of DHS sites depleted in promoter regions relative to Common DHS sites and enriched in distal intergenic regions and within introns (Figure 2e).

We also compared chimpanzee DHS gains and losses to DNase-seq results from a diverse set of 27 human cell types. We found that chimpanzee DHS gains did not largely overlap with DHS sites from any of the 27 human cell types (5–23%, Figure S6a, Table S4) while chimpanzee DHS losses were more likely to overlap human DHS sites, especially those from human fibroblasts (73%, Figure S6b, Table S4). Thus, comparisons to diverse cell types indicate that Common DHS sites have been selectively maintained through millions of years of primate evolution suggesting a role in housekeeping function. In contrast, more recently evolved DHS sites unique to humans and chimpanzees are likely functional in a small fraction of cell types with related functions.

### Species-specific DHS sites are associated with biological function

**Figure 5:**
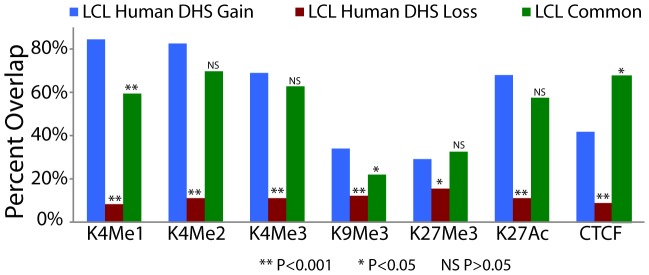
Species-specific DHS sites are associated with functional chromatin marks. DHS sites detected only in human, but not chimpanzee, LCLs are more enriched for ChIP signals in matched human cell types. LCL histone modification and CTCF ChIP-seq data were previously generated from the GM12878 cell line [33]. Fisher's exact test *P* value significance levels indicated by asterisks or NS (not significant) are provide for LCL human DHS gains compared to LCL human DHS loss and LCL common DHS regions (Table S6).

Species-specific DHS sites were compared to cell-type matched human ChIP-seq data for multiple active histone marks and transcription factor binding sites. We found that human-only DHS sites were better associated with these marks compared to chimpanzee-only DHS sites (Figure 5). This enrichment was highest for H3K4me1, H3K4me2, H3K4me3, and H3K27ac, consistent with chromatin marks predictive of enhancers [20], [33] (Figure 5, Table S6). H3K4 methylation signals were detected in a higher percentage of LCL human DHS gains compared to Common DHS sites, while CTCF, a known insulator protein, is enriched in LCL Common DHS sites (Figure 5, Table S6). The combination of adjacent chromatin marks and their location relative to genes (Figure 2e) provides further evidence that species-specific regulatory elements are functional. These data suggest most regulatory elements gained or lost after the human-chimpanzee divergence are preferentially associated with enhancers, while Common regions are preferentially associated with promoters and insulators.

### Species-specific DHS sites are near genes that exhibit species-specific expression

**Figure 6:**
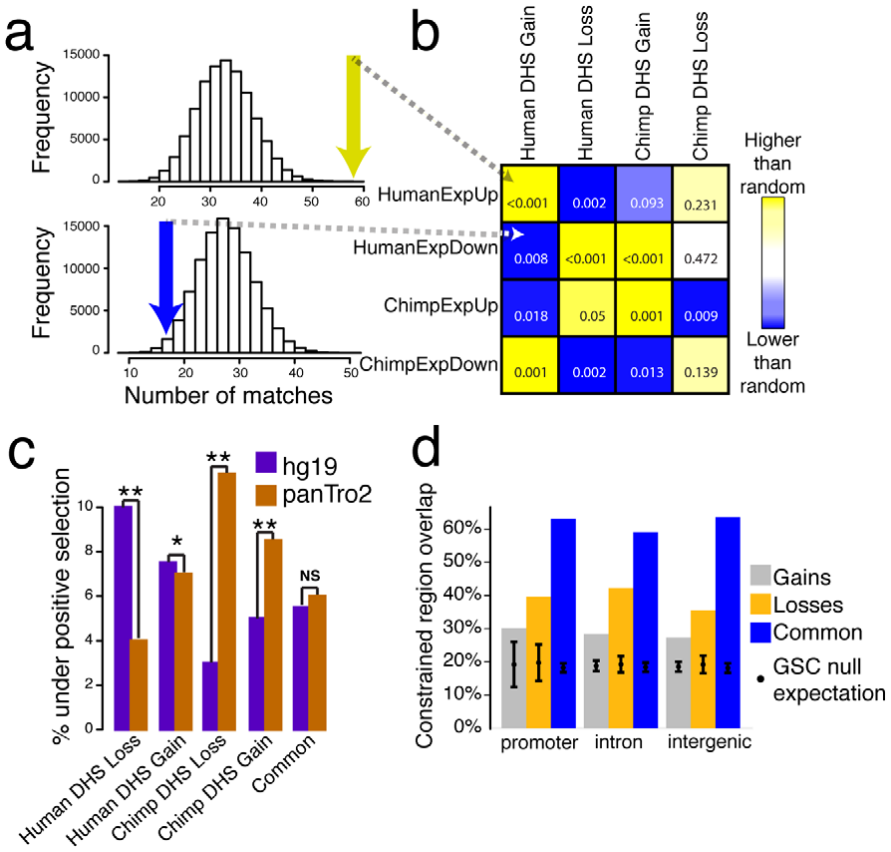
Species-specific DHS sites are correlated with expression and evolutionary selection. (a) 58 human DHS gains (yellow arrow) overlapped human upregulated genes (HumanExpUp), a highly significant enrichment compared to 100,000 random permutations (*P* = 0.00039). Only 17 human DHS gains (blue arrow) overlapped human downregulated genes (HumanExpDown), which is lower than random permutations (*P* = 0.008) (b) Comparison of DHS gains and losses with expression gains and losses. Yellow represents DHS and expression matches that occur more often than random permutations, while blue represent less often. *P* value indicated in each box. (c) Percentage of regions that display evidence of positive selection on the human (purple) or chimpanzee (brown) branch. Both human-specific DHS site gains and losses show more evidence of positive selection on the human branch, while chimpanzee-specific DHS site gains and losses show more evidence of positive selection on the chimpanzee branch (*P<0.03, ** *P*<0.002). Common sites show an equivalent amount of selection on both branches. (d) Percentage overlap of DHS Gains (combined from both human and chimpanzee), DHS Losses, and Common DHS sites compared to evolutionarily constrained regions generated using GSC (Materials and Methods). Regions were divided into three compartments: promoter, intron, and intergenic regions. The black dot represents the null expectation of finding a constrained region and error bars represent one standard deviation.

We expect species-specific DHS sites that contribute to phenotypic differences would be located near genes differentially expressed across species. To test this, we measured the proximity of fibroblast DHS site gains and losses to genes with variable expression (Figure 1a). From matched fibroblast expression data, we used edgeR [26] analysis to identify 1047 human upregulated genes, 881 human downregulated genes, 785 chimpanzee upregulated genes and 788 chimpanzee downregulated genes (Supplemental data file 1 in Dataset S1). Human DHS gains were significantly enriched (permutation test, *P value* = 0.00039) near genes with increased expression in human and depleted (*P* = 0.008) near genes with decreased expression in human (Figure 6a–6b). Similarly, human DHS losses were enriched (*P* = 0.008) near genes downregulated in humans and depleted (*P* = 0.002) near genes upregulated in humans (Figure 6b). The same relationships between DNase-seq signal and expression held true for chimpanzee (Figure 6b, and Table S7). Analogously, we found that significantly upregulated genes were more likely to be near chromatin gains and downregulated genes near chromatin losses in each species compared to genes similarly expressed in both species (Figure S7a–S7b, Table S8, Materials and Methods). These results support a direct role for species-specific DHS site differences in species-specific gene regulation. The direction of these correlations indicate that DHS site gains and losses are more commonly associated with enhancers than repressors. The LCL DNase-seq and expression data from human and chimp show a similar trend (Figure S8).

Many species-specific expression differences were not readily explained by the presence of a nearby species-specific DHS site. For example, though statistically, genes upregulated in human were enriched near human DHS gains, this was true for only 58 of 1182 higher expressed genes (Figure 6a, Table S7). This may be partially explained by our strict definition of human DHS gains. Also, long-range interactions may confound the simple way we assigned DHS sites to the nearest gene. Future studies involving chromatin conformation capture (e.g., 3C, 4C, 5C) could be used to better map DHS sites to target gene(s). Lastly, expression differences between species may result from transcription factor binding characteristics that do not alter chromatin structure. Comparative ChIP-seq studies for specific transcription factors will be necessary to determine the extent of this phenomenon.

We conducted gene ontology enrichment analysis for both species-specific DHS sites using GREAT [34] and differentially expressed genes using GO (http://david.abcc.ncifcrf.gov/), but did not find many highly enriched categories in either analysis (Table S9). This indicates that chromatin gains and losses occur near many different types of unrelated genes representing a broad spectrum of gene ontologies.

### Species-specific DHS sites show evolutionary selection and constraint

The functional interpretations of Common and species-specific DHS sites outlined above naturally lead to predictions about the operation of natural selection. We used HyPhy [35] to test for signatures of positive selection within DHS gains and DHS losses on either the human or chimpanzee lineage [15], [16] (Materials and Methods). Consistent with a functional change unique to humans, both human DHS gains and losses showed significantly more evidence for positive selection on the human branch than on the chimpanzee branch (Mann-Whitney *P* = 0.03 for gains and *P* = 0.0009 for losses, Figure 6c, Table S10). Similarly, both chimpanzee DHS gains and losses showed increased positive selection on the chimp branch (*P* = 0.002 for gains and *P* = 0.0004 for losses, Figure 6c, Table S10). Signatures of selection for Common DHS sites were not significant on either branch. These results provide evidence that positive selection contributes to species-specific changes in chromatin, both gains and losses, and in the altered use and activity of gene regulatory elements.

Despite this connection with evolutionary pressures, only two DHS gains or losses in fibroblasts overlap previously defined human accelerated conserved non-coding sequences (HACNSs), chimpanzee accelerated conserved non-coding sequences (CACNSs), or human accelerated regions (HARs; Table S11) [14], [36]–[38]. More generally, few DHS sites from any human cell type we have analyzed, including embryonic stem cells, correspond to genomic regions of accelerated turnover (Table S11e). This lack of overlap may be due to the absence of DNase-seq data from specific developmental cell types since HACNSs, CACNSs, and HARs have been associated with developmental gene regulation, or to regions of accelerated turnover representing a different type of genetic element not detected by DNase mapping.

We examined sequence conservation in DHS gains, losses, and Common sites using evolutionarily constrained regions defined by PhastCons [14], [39] and GERP [40] algorithms with Genome Structure Correction (GSC) overlap test statistic [19], [41], [42]. By PhastCons analysis, we found that Common DHS sites were the most conserved, a characteristic of regions under negative selection (Figure S9). Common regions also had the greatest overlap with evolutionarily conserved elements, as defined by GERP (Figure 6d; Materials and Methods). The presence of Common DHS sites in most human cell types (Figure 4c) with presumably greater functional demands may contribute to their higher conservation levels relative to gains and losses. Additionally, losses in both species were more conserved and overlapped more with conserved elements than gains (Figure S9) suggestive of relaxed selection and positive selection, respectively. These trends held true even when noncoding genomic regions were partitioned based on their relationship to genes (promoter, intron, intergenic; Figure 6d). In general, higher degree of conservation within specific regions of the genome can result from local differences either in mutation rate or selection [43]. Given that localized decreases in mutation rate below background are unusual, our data suggest that sequence conservation within Common DHS sites is primarily driven by negative selection to maintain function.

A large fraction of DHS gains (∼70%), losses (∼60%), and Common (∼40%) sites did not overlap any highly conserved elements (Figure 6d). Thus, many DHS sites present in all three species, and possibly many or all of 27 human cell types, are not highly conserved. Understanding how these regions function in all species and cell types without high sequence conservation poses an interesting challenge for evolutionary genomics.

### DHS gains near DHS losses are potential binding site turn-over regions

Previous studies have shown that individual transcription factor binding sites (TFBS) “turn over” rapidly during evolution [44]–[46]. Transposon-mediated shifts in the position of enhancers have also been documented between mouse and human [47]. While these showed evidence of TFBS positional change, the turnover of entire DHS sites have not been shown previously. We identified ten possible instances of regulatory-element shuffling where a human DHS gain maps near (<50 kb) a human DHS loss (Figure S10). These regions were found near genes associated with obesity (MCR4, Figure S11), imprinting (GNAS, Figure S12), and glial cell formation (METRNL, Figure S13). We also found cases of nearby (<50 kb) human and chimpanzee DHS sites that were independently gained (Figure S14, Figure S10). One region mapped within an intron of the SRGAP2 gene (Figure S14), which is involved in neuronal guidance during brain development. Overall, the number of DHS gains and losses that mapped within close proximity to each other was not largely enriched or depleted based on randomized permutation tests, thus we cannot disprove that these findings are due to chance observations. Further detailed functional analyses are needed to determine the biological significance, if any, of these closely mapped regulatory changes.

### DHS sites are found in genomic segments deleted in the human or chimp lineage

Our analyses above focused exclusively on DHS sites mapped to genome sequences shared between all three primate species. Recently, segments of DNA broadly conserved among mammals were found deleted specifically in the human (hCONDELs) or chimpanzee (cCONDELs) genome [48]. It has been proposed that these largely gene-desert regions contain regulatory elements that contribute to species-specific phenotypes [41]. We found human and chimp DHS sites mapped to 6% of cCONDELs and 11% of hCONDELs supporting their role in species-specific gene expression (Table S11). Many human fibroblast DHS sites that overlap cCONDELs were also present in other human cell types (Figure S15) indicating that some CONDELs contain regulatory elements with pleiotropic consequences.

### Specific motifs may confer species-specific DHS sites

**Figure 7:**
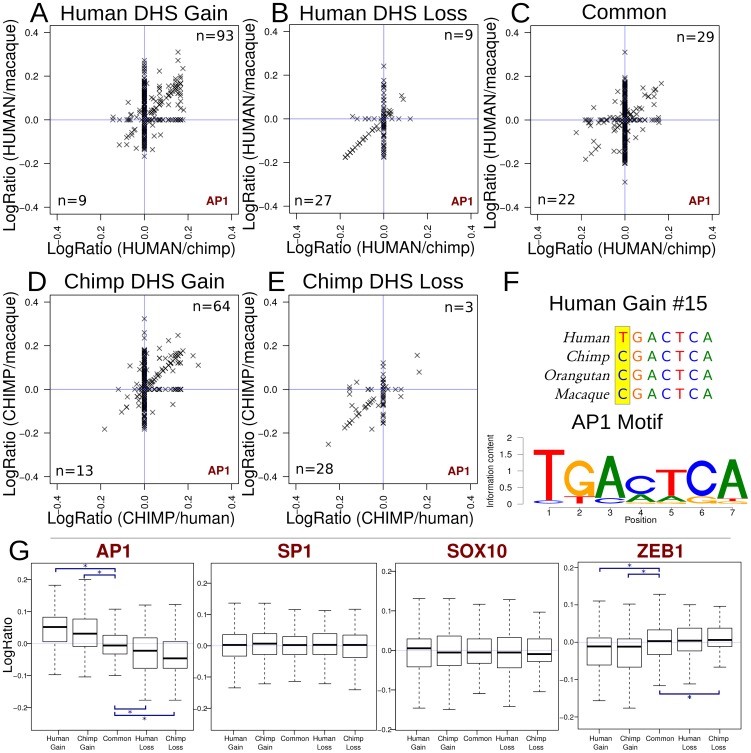
Functional mutations associated with DHS gains and losses. (a–e) Scatterplots showing the enrichment of AP1 motif matches in species with increased hypersensitivity. Each “x” represents a single DHS site. (a–c) positive values on each axis indicate better motif matches on the human branch. For these regions, points in the upper-right quadrant are regions where the AP1 motif scores better in human than either chimp or macaque, where the lower left represent AP1 motif scores worse in human. The number of DHS sites in these quadrants are indicated. (d–e) For chimp gain and loss regions, positive values for each axis indicate a better motif match in the chimp branch. (f) The AP1 motif from JASPAR and an example alignment of a representative human gain region representing a point along the diagonal in the upper-right quadrant in panel a. (g) Boxplots summarizing the results from AP1 and three other motifs. The boxplots show the distribution of (combined) log-ratios (relative to the appropriate species). *P* values for differences relative to common regions are significant (asterisk) in all 4 comparisons: human DHS gains, *P*<10^−31^; human DHS losses *P*<10^−3^; chimp DHS gains, *P*<10^−13^; chimp DHS losses, *P*<10^−8^ (Materials and Methods). In AP1, the significant trends illustrate the same principal observed in panels a–e. Most other transcription factors have plots that show no pattern in motif score among species, such as SP1 and SOX10 (Supplemental data file 3 in Dataset S1). ZEB1, a transcriptional repressor, displays an inverse relationship with hypersensitivity.

We analyzed TFBS motifs found within DHS gain, loss, and common sites across species to identify motifs associated with differences in hypersensitivity. To quantify differences, we determined log ratios of the best position weight matrix (PWM) score in a DHS site between species (Materials and Methods). Most motif scores from the JASPAR database were distributed evenly between species (log ratio near zero) indicating no species-specificity trend for that motif (Supplemental data file 2 and 3 in Dataset S1). However, log ratios of AP1 motif scores deviated from zero and correlated with species-specific DHS sites (Figure 7). For example, in human DHS gains, AP1 motif match scores were higher in the human sequences and lower in the orthologous regions in chimp and macaque (Figure 7a). In contrast, AP1 motif scores in human DHS losses were higher overall in both chimpanzee and macaque sequences compared to human (Figure 7b). Common regions showed even distributions of AP1 motif scores across all three species (Figure 7c). This trend was also found in chimpanzee where chimp DHS gains had higher AP1 motif scores in chimp sequences compared to orthologous regions from human and macaque (Figure 7d), and chimpanzee DHS losses had higher AP1 motif scores in human and macaque (Figure 7e). In a representative human DHS gain, we see that the human allele results in a better match to the canonical AP1 motif than the non-human primate alleles (Figure 7f).

These results suggest species-specific sequence changes within the AP1 motif promote hypersensitivity in some species-specific DHS sites in the human, chimp, and macaque genomes. AP1 was the clearest example of this from motifs represented in JASPAR (Figure 7g, Supplemental data files 2–3 in Dataset S1). Other transcription factors may be acting similarly, but less frequently. For example, we also found that ZNF354C (Supplemental data file 3 in Dataset S1, page 81) and NFE2L2 (Supplemental data file 3 in Dataset S1, page 108) showed similar trends to AP1. In these cases, motif scores positively correlated with the presence of a species-specific DHS sites. In contrast, ZEB1 displayed the opposite trend where higher motif scores correlated with the lack of a species-specific DHS site (Figure 7g and Supplemental data file 3 in Dataset S1, page 65). While the mechanism is not yet clear, our findings and ZEB1's known role as repressor [49] is suggestive of its ability to induce a closed chromatin state via binding to CtBP and HDAC [50].

## Discussion

Precise measurements of transcript abundance enabled by RNA-seq experiments have revealed extensive differences in gene expression among closely related species [51] with 10–20% of transcripts within a given tissue found differentially expressed between humans and chimpanzees [12], [22]. Many transcripts are tissue-specific, and given the relatively small number of cell types explored, the total number of differentially expressed genes is likely to be considerably larger. An important goal of molecular evolution research is to understand how differences in transcript abundance have evolved, both because the changes are extensive and because some may underlie the origin of functionally significant traits [13], [52], [53].

Most gene expression differences across species likely have a genetic basis, but it is difficult to relate expression changes to variation in genome sequences. While many non-coding sequence differences are unlikely to impact transcription, for the subset that do, it is often not clear what genes are directly affected. In addition, a non-coding regulatory mutation may only affect gene expression in a subset of tissues or developmental stages, so many functional consequences have gone unrecognized given the limited number of studies performed thus far. Further complicating analysis, transcription is influenced by environmental factors and by epigenetic modifications. But the lack of a complete regulatory element map across species and tissues is perhaps the most important impediment to understanding gene expression differences in terms of genome sequence evolution. Changes in transcript abundance may be caused by genetic differences within individual regulatory elements in *cis* that affect transcription factor binding affinity [54], or within transcription factors that affect binding to many regulatory elements in *trans.* Even when the genetic basis is known to be in *cis,* there is no reliable method for identifying the causal mutations from sequence comparisons. As a result, distributions of positive and negative selection genome-wide correlate poorly with changes in transcript abundance [12], [55], [56].

In this study, we showed that analyzing chromatin accessibility using DNase-seq provides a powerful approach to link genome sequence changes to species- and tissue-specific differences in gene expression. Chromatin accessible DHS sites have three properties that make them especially valuable for evolutionary analyses of gene expression [17], [18]. First, DHS sites identify all known functional classes of regulatory elements, including core promoters, enhancers, repressors, boundary elements, and locus control regions, thus revealing all *cis* components of transcription through a single genome-wide assay. Second, DHS sites are only found when a regulatory element is active or poised, which means that DNase-seq can be used to identify evolutionary changes in tissue- and developmental stage-specific regulatory elements. And third, DHS sites represent only ∼2% of the genome, making it possible to focus analyses on regions that are involved in transcriptional regulation and ignore regions that are not.

We performed DNase-seq on fibroblasts from three primate species and identified more than two thousand regulatory elements apparently gained or lost since the divergence of humans and chimpanzees. Turnover of regulatory elements was enriched near genes that display species-specific expression differences, indicating that gains and losses in DHS sites have functional consequences on transcript abundance. To our knowledge, this is the first evidence correlating changes in DNase chromatin accessibility and gene expression across species at a genome-wide scale. We found most expression differences occurred without a detectable change in a nearby regulatory element. One possibility is that mutations within DHS sites affect transcription factor binding without causing large changes in overall chromatin accessibility. Future experiments are needed to identify the specific sequence changes that regulate expression at long distances and/or via post-transcriptional mRNA stability mechanisms.

Most regulatory element changes occurred within intergenic regions and introns and were predominantly associated with cell type-specific DHS sites. These results are consistent with expected differences in the extent of pleiotropy: loss of core promoter elements will more likely affect transcription in many tissues and stages of development, while loss of distal enhancers will more likely affect transcription in a subset of tissues. Lower rates of change in core promoter elements and in regulatory elements actively utilized in multiple tissues suggest negative selection is operating to maintain regulatory elements with more critical functions.

Analysis of the DNA sequences within regulatory elements provides evidence for the operation of natural selection within these elements. Sequence within DHS sites utilized across all three species show lower rates of substitution than surrounding DNA, which is a proxy for neutral evolution by drift, consistent with negative selection operating to maintain their function. In contrast, regulatory element gains on the human and chimpanzee branches have significantly elevated rates of substitution, consistent with positive selection for altered function, while regulatory element losses show slightly elevated rates, perhaps due to relaxed selection. Explicit tests for positive selection using branch-specific likelihood ratio tests [16] reveal that the highest association is with regulatory element gains and the lowest with common regulatory elements utilized in all three species. Thus, the genome-wide distribution of both negative and positive selection within regulatory elements correlates in predicted ways with the evolutionary conservation and change in their function. Although we are not aware of any previous evidence for such a relationship, it seems likely in principle that the operation of natural selection is often tied to gains, losses, and conservation of regulatory elements. Most instances of inferred positive selection we identified do not overlap previously described HARs [14] or HACNs [36] highlighting that our DHS gains and losses represent a novel set of differential regulatory elements may have played a role in adaptation during human evolution.

Many studies have documented evolutionary gains and losses of individual transcription factor binding sites or H3K4me3 histone marks among related species [44]–[46], [57], but this is the first evidence showing gains and losses of entire DHS sites. Since we only examined two cell types and applied conservative identification criteria, the full extent of regulatory element changes between humans and chimpanzees is likely to be considerably greater than we report. Nonetheless, the instances of turnover we identified suggest regulatory element gains and losses are a common class of functional change within evolving genomes.

We show that sequence differences among species within particular motifs may result in species-specific DHS sites, which suggests one way non-coding regulatory variants can alter chromatin structure. In particular, mutations that produce better matches to the activator protein 1 (AP1) motif on either the human or chimpanzee genome correlate with the presence of species-specific DHS sites, a result detected in human DHS gains and losses as well as chimpanzee gains and losses. Sequence changes increasing the affinity for AP1 motif more likely drive species-specific changes in chromatin structure rather than species-specific coding mutations within the AP1 components, FOS and JUN proteins, altering the sequence-binding preference of AP1. Since only a minority of species-specific DHS gains and losses has differential AP1 motif scores (Figure 7), this indicates the majority of factors that govern species-specific DHS sites remain to be discovered. AP1 has been implicated in many aspects of cellular function ranging from proliferation, transformation, differentiation, oncogenesis, apoptosis, hormone activation, to tumor suppression [58]–[61]. We provide evidence that other factors act similarly to AP1 or in the opposite direction as repressors, such as ZEB1. Mapping DHS sites across a more diverse set of primate samples, as well as using additional *de novo* motif discovery and performing ChIP-seq to reveal binding sites, will be an important part of identifying additional factors that confer changes in chromatin structure across species.

## Materials and Methods

### Cell types and tissue culture

We obtained two cell types from Coriell for this study: skin fibroblast cells and lymphoblastoid cell lines (LCLs). Primary skin fibroblast cells from three human, three chimpanzee, and three macaque individuals. LCLs, which are B cells immortalized with Epstein-Barr Virus, were obtained from the same three human and three chimpanzee individuals that fibroblasts were isolated from (Table S1). EBV does not reliably transfect macaque lymphocyte cells, so matched macaque LCLs cells were not available for this study. Importantly, other recent genome-wide studies that used macaque LCLs were of B-Lymphocyte cells transformed with rhesus herpes papio virus, a close relative of human EBV [57]. Cells from all species were grown in standard growth media. Fibroblast growth media consisted of Gibco's MEM (10370-021), L-Glutamine (25030-081), Pen/Strep (15140-122), and 10% FBS (Hyclone SH30070). LCLs growth media consisted of Gibco's RPMI (21870) media with L-Glutamine, Pen/Strep, and 15% FBS. We harvested fifty million cells for each individual biological replicate and allocated 35 million cells for DNase assays (DNase-seq and DNase-chip), 10 million for genomic DNA (used as control for DNase-chip array hybridization), and 5 million for RNA DGE-seq expression analysis.

### DNaseI HS library preparation

DNase-seq libraries we generated as previously described [17], [18] and sequenced via Illumina's GAII sequencer. DNase-chip library preparations, used for validation of our DNase-seq results were performed as previously described [24], [25] and were hybridized to 1% ENCODE Nimblegen arrays [19]. Custom arrays were designed to cover the orthologous regions from chimpanzee and macaque. DNase-chip array intensities were compiled and significant DHS sites were called using ChIPOTle [62] (*P*<0.000001 peak cutoff).

### DNase-seq aligned to native genomes and lifted to hg19

DNase-seq data generated from each species was aligned to the native genome (human hg19, chimpanzee panTro2, and macaque rheMac2) using BWA [63]. To directly compare three different primate species requires that they be aligned to a single reference sequence. Because both the chimpanzee (panTro2) and macaque (rheMac2) reference sequences were built from the existing human reference, we converted all sequences to human coordinates. To do this, we converted each 20-mer DNase-seq sequence from panTro2 or rheMac2 to hg19 with liftOver [23], using a match setting of 80 percent. After conversion to hg19 coordinates, we used F-seq [64] to identify DNaseI hypersensitive (DHS) sites. The F-seq scores from the top 100,000 peaks from each sample were used to determine how well chromatin openness correlates among all 15 samples (Figure 1). This analysis uses a pairwise Pearson correlation to compare the similarity among samples.

### Identifying species-specific hypersensitive sites and filters to eliminate liftOver artifacts and CNVs

We used the bioconductor edgeR package to define species-specific hypersensitive regions [26]. EdgeR is designed to detect differences in count data among groups of samples. Briefly, it compares within-group variances to between-group variances using a negative binomial model, and selects entries with significant between-group differences. It was designed for differential expression data such as DGE-seq or RNA-seq, but it is similarly applicable to read counts generated by DNase-seq. One key advantage of edgeR is a normalization procedure specifically designed for high-throughput sequencing studies [26].

To locate significant differences in DNase-seq signal between species, we first identified the union set of the top 100,000 DHS sites (as scored by F-seq) from all 15 samples (9 fibroblasts and 6 LCLs). We used bx-python (https://bitbucket.org/james_taylor/bx-python) to analyze the data. We divided these regions into windows, attempting to maximize the resolution of the windows while minimizing the number of windows required:

#### Defining windows

We divided the union set into overlapping windows of 300 bp. DHS sites smaller than 300 bp were expanded to 300 bp. Regions larger than 300 bp were tiled with overlapping windows; the overlap varies depending on the size of the hypersensitive region to tile. We start by finding the number of windows that would fit completely inside the defined HS site using the default overlap (100 bp). If these windows discard fewer than 10% of the bases on each edge of the HS site, we tile the site using these windows, because the initial peak calls tend to run a bit wide. If using the default overlap would cause us to lose more than this edge threshold (10%), we add another window and adjust the overlap so the windows exactly cover the entire HS region. These windows cover almost all of the HS bases in the original, while minimizing the number of non-HS bases considered for the downstream analysis. After defining the windows, we liftOver each window back to chimp and macaque and discard any windows that don't map. We also eliminated any windows mapping to the Y chromosome, because we have female samples. This resulted in ∼1.2 million windows across all DHS sites (Supplemental data file 4 in Dataset S1).

#### Zero counts filter

We next counted the number of reads (DNase cuts) that mapped within each window for each sample. To be certain to compare only regions that are actually found in all 3 primates, we omitted any windows that did not have at least one read count in at least one individual from each species. We believe this is a reasonable filter since there is a basal level of DNase digestion throughout the genome, and regions without any mapping reads in one species are likely caused by alignment ambiguities (such reads are lost in the liftOver process) or reference problems.

#### Significant differences

We then used edgeR to call windows with significantly different counts in each pairwise comparison at a false discovery rate of 1%. This defined species-specific HS windows. For example, to define human-fibroblast-specific (human DHS gain) regions, we selected any windows with significantly more counts in humans compared (pairwise) to both chimp and macaque fibroblasts. We then merged any significant neighboring windows.

#### Reciprocal liftOver filter

To confirm that DNase sites detected in only a single species were not due to a liftOver artifact or copy-number variation (CNV), we performed a reciprocal liftOver. Reciprocal liftOver discards regions that do not map 1∶1 between species assemblies. After defining a species-specific or Common DHS sites (Common DHS described below) in human coordinates, we the lifted these windows back to panTro2 or rheMac2. We then compared number of reads from the original native genome alignment to the read count on the lifted (hg19) reference. We found that, in general, these scores correlate well. However, some windows differ in counts on each reference, indicates liftOver artifacts or CNVs. Windows with native counts exceeding the reference counts could result from counts from the native assembly being lifted to multiple places on hg19. Likewise, windows with more reads on hg19 than on the equivalent native assembly could be caused by several regions from the native genome lifting to a single location on hg19. Since these may possibly artificially appear as chromatin gains or losses, we filter out these scenarios by requiring that the number of reads on hg19 for chimp/macaque data be within 10% of the number of reads on the native assembly.

#### Sharp peaks and assembly filters

Some regions mapped to areas in the genome that are clear artifacts, most likely CNVs. These artifacts are expanses where one of the species has much higher levels of hypersensitivity than the others, and are often located near centromeres or telomeres. A DHS resulting from a massive expansion in one of our samples compared to reference genome appears as a large (kb to Mb-scale) DHS site encompassing the entire CNV and/or DHS sites that cluster closely together. We manually curated a list of such areas and filtered data mapping within these regions (Assembly filter). We also eliminate abnormally sharp peaks, which are likely caused by PCR artifacts, reference assembly inaccuracies, or small differences in copy number between our samples and the reference genomes. If any 30 bp window within a region contains 75% or more of the reads in the entire region, these regions are filtered out as “sharp peaks”. Ultimately, we confirmed the regions by visually inspecting many using both the Integrative Genomics Viewer (IGV) [65] and the UCSC Genome Browser [23]. These filters apply to DHS gains, losses, and common regions.

### Addressing the confounding effects of skin biopsy location and Fibroblast heterogeneity

Chang et al. (2002) showed that human skin- and non-skin- fibroblast samples collected from different locations along the body plane showed notable differences in transcriptional profiles [29]. Aware of this issue, we made an effort to use fibroblast cultures made from only skin samples and from the same region of the upper arm. All 3 replicates of macaque Fibroblasts and one human Fibroblast were confirmed from Coriell to be from skin biopsies from the upper arm (the other two locations were unknown). Since our analysis poses a strict requirement of DHS sites to be present in all three human individuals to be called a human DHS gain, having at least one human sample with the biopsy site confirmed to be from the upper arm ensures that the human gains are not a result of human DHS gains being a result of, for example, all human fibroblasts isolated from lower leg. Likewise, to be called a human chromatin loss, DHS sites have to be absent in all three human samples (but present in all chimp and macaque samples); this biopsy location bias is again mitigated by at least one human sample being from the upper arm.

The Yerkes National Primate Center, from where the chimpanzee skin fibroblasts were obtained from, unfortunately do not document the exact location of biopsy. While the standardized skin punch protocol calls for the location of the skin biopsies to be from the ear pinna (personal communication with Fawn Conner-Stroud from Yerkes), we cannot be 100% sure that the samples were isolated from this location. As described above, human DHS losses are a result of signal being present in all three chimpanzee and macaque samples, supporting that these regions are not due to chimp biopsy location. We also want to reiterate that our skin fibroblast results are highly similar in LCL lines that are all uniformly derived from blood samples.

We find that none of our chromatin gains and losses overlap the Hox genes described in the Chang et al., 2002 paper [29]. In addition, a more recent analysis by Rinn et al. [30], using more comprehensive microarrays and more biopsy sites, identified 337 expression array probes (299 unique genes) that were shown to be highly associated with five different general biopsy site locations. We have compared this list of genes to both species-specific chromatin gains/losses, as well as species-specific gene expression, and find <3% of the species-specific and common DHS overlap with the 299 position specific genes. Similarly, species-specific and commonly expressed genes also show <3% overlap (Table S12).

### Identifying DNaseI hypersensitive sites in all species (“Common” sites)

In order ensure that our tests for selection were meaningful, we wanted to compare chromatin gains and losses with a set of regions that were open in all species. Rather than simply choose DHS sites that have the highest scores, we wanted to mirror the level of hypersensitivity to that of the species-specific regions. This is important because species-specific DHS sites are not necessarily the strongest DHS sites. We also wanted to select a set of regions similar in size to our sets of gains and losses to retain computational tractability.

To select a set of matched Common DHS sites, we required that each window be similarly open in all 9 samples from all 3 species. To be considered “similarly open” in a given sample, the number of counts must lie between the 20% and 80% quantiles for that sample in the corresponding species-specific regions. For example, we used the human DHS gains identified by edgeR to define the distribution for each of the three human samples, and similarly for chimp and macaque. As such, our set of Common regions is the set of all windows with DNase counts within this range for each of the 9 samples (Figure 2d). To reduce the number of Common regions we found to the most representative set (those that *most closely match* the average signal intensity of the differential DHS sites), we narrowed the quantiles until we found a set of around one thousand Common regions, which we reasoned would be a sufficient number to examine summary statistics. To ensure that our results are not biased for a specific set of Common regions, we repeated our experiments on a significantly larger set (∼11,000) of Common DHS sites using less stringent criteria (10%–90% quantile). This larger set is even more enriched for promoter regions but does not change our conclusions (data not shown).

After identifying an initial set of potential Common DHS sites, we filtered out any that appeared to be “appendages” to other hypersensitive sites. Without this step, many Common DHS sites would map to the edge of a strong hypersensitive sites. To ensure that a Common DHS site is a standalone DHS site, we examined the neighboring windows surrounding the initial set of Common DHS sites. If a Common DHS site window contained fewer than 80% of the number of reads in the adjacent window on either side, we filtered it out as most likely an “appendage” to a stronger DHS site. This resulted in a final list of 1259 Common DHS sites matched in intensity to the species-specific DHS sites. We also ran this filter on DHS gains and losses, and found that very few of the gains (3–5%) and losses (3–8%) get flagged as “appendages.” Of these, many of them are flagged as a result of FDR threshold issues that simply didn't quite highlight a neighboring window, and we would actually still consider this a legitimate gain region. Because gain/loss appendages are relatively rare and are largely due to threshold issues, we elected to retain them in our final list.

### Why are there more species-specific increases than decreases?

In every comparison, we reported more species-specific gains than losses. The most important factor in determining the size of these lists is the level of significance we set by choosing a FDR. To obtain lists that match in length, we could simply adjust the FDR value for the lists to yield about the same number of regions. Instead, we decided to keep the FDR constant and select varying numbers of DHS sites for each category.

However, it is still constructive to consider the disparity. In other words, “at a constant FDR (1%), why are there more species-specific increases than decreases?” This is possibly a result of purifying selection. Because DHS sites are regulatory (and therefore tend to be conserved), a loss of a DHS site probably confers a fitness disadvantage. In this case, we would expect to see more gains than losses.

It is also possible that the prevalence of increases is simply a result of the way we constructed the significance test. A DHS site is a sparse signal (there are more “closed” regions than “open” regions). Combined this with the asymmetry of the evolutionary tree: the chimp and human are more similar to each other than either is to the macaque. A human-specific increase requires both macaque and chimp to be closed (the default), while a human-specific decrease requires both macaque and chimp to be open. This latter scenario will happen less often because the relationship between chimp and human is closer than either to the macaque.

In short, the greater number of gains than losses in our analysis may reflect purifying selection on DHS sites; however, it may also simply be a result of the way we constructed the test, particularly due to using an outgroup species to polarize the chromatin structure changes.

### Testing for selection

We tested for evidence of positive selection using the DHS sites indicated as DHS gains, losses, and commons defined by edgeR and common analyses (see above). A branch model test [66] in HYPHY [35] was used to assess evidence for positive selection on each the human and chimp branches. HYPHY uses a likelihood ratio test to compare two opposing models. For the null hypothesis, we specified a composite model that allowed for negative selection, neutral evolution, or relaxed constraint specifically on the branch of interest (*i.e.* the human branch), with negative or neutral evolution across the rest of the tree. The alternative hypothesis modeled positive selection only on the branch of interest, with negative or neutral evolution on the rest of the tree. For each region, HYPHY performed a likelihood ratio test comparing these hypotheses and output a *P* value that can be interpreted as a level of evidence for positive selection. In order to test the likelihood of either the null or alternative hypothesis, we specify both the alignment of the region of interest, as well as a background sequence alignment assumed to be evolving neutrally [67]. For the alignment of the region of interest, we used alignments of human, chimp, macaque, and orangutan precomputed at UCSC. For the background sequence, we collected a separate set of local introns for each region to test, following Haygood et al. [16]. To define these background alignments, we started with the UCSC knownGene definition of intron annotations, and then filtered out all first introns, splice junctions, and hypersensitive sites (in any of the 15 samples in this study). In this way, we aimed to select sequences that are evolving neutrally. After defining this set of neutral introns, we used an expanding window centered on the region of interest to collect introns in a region up to 100 kb surrounding the center. We added introns to this collection sequentially as the window expanded until we reached an alignment of 2000 nucleotides. If we were unable to find 2 kb of background introns within 100 kb of sequence, we discarded these regions (this happens rarely). Introns are commonly assumed to be evolving neutrally [43], [68], particularly when our filtering steps are taken into account; however, there are still likely to be regulatory sequences present in our background model, either due to sequences containing DNaseI HS sites in other cell types not tested or due to unannotated or mis-annotated transcripts. In order to further correct for this possibility, we performed each likelihood-ratio test 50 times, using 50 different bootstrapped versions of the background model. We then averaged these *P* values to assign a final *P* value for each region. This method has the effect of possibly discarding any elements under selection in some of the bootstrap replicates, increasing our ability to detect positive selection even if we inadvertently chose some background regions under selection. To test significance, for a given set of regions (e.g. human DHS gains) we ordered the *P* values for selection on both hg19 and panTro2, then did a Mann-Whitney test to see if one branch has higher *P* values than the other (Table S10).

The fibroblast DHS sites where we can polarize the differences using macaque all have significant differences in the direction we expect, while the Common regions do not have significant differences. In the LCLs, where we are unable to polarize (no macaque LCLs were available), we do not detect a significant difference. This is likely due to a combination of two categories (gains and losses) that have competing selection (i.e. LCL human DHS loss = human DHS loss+chimpanzee DHS gain).

### Testing for evolutionary constraint

We calculated the observed fraction of overlaps between DHS sites and evolutionarily constrained regions using constrained regions defined by the Genome Evolutionary Rate Profiling (GERP) method [40] executed on Enredo, Pecan, Ortheus (EPO) [69], [70] 33-way alignments. EPO alignments and GERP regions are available for download at the Ensembl browser (http://ensembl.org). We then constructed a null distribution of the fraction of expected overlaps by using the conservative Genome Structure Correction (GSC) methodology described previously [19], [41], [42]. We performed 1000 randomizations and calculated the mean and standard deviation from the null distribution to assess the statistical significance of the observed overlap (Figure 6d).

We also used PhastCons to explore degree of sequence conservation. For each region, we extracted the mean and max PhastCons score from the primate PhastCons table at UCSC. We then compared the distribution of these scores across the regions to see how sequence conservation is related to hypersensitivity conservation (Figure S9).

### Expression analysis using digital gene expression (DGE-seq)

Total RNA purified from 5–10 million cells harvested from the same cell culture used for DNase-seq were also used to generate DGE-seq expression libraries as previously described [12], [71]. Polyadenylated RNA is captured for enrichment of mRNA and the oligo dT primer is used to make cDNA. Briefly, DGE-seq is similar to Serial Analysis of Gene Expression (SAGE) where mRNA abundance is assessed via counting short sequences of their cDNA at specific restriction site locations. These DGE-seq libraries were sequenced using Illumina's GAII sequencer, and averaged 10 million 20mer sequences for each sample, which were then aligned to the samples' native reference sequence using BWA. We used EdgeR to detect differences in tag counts across species by comparing intra-species variances to inter-species variances using a negative binomial model, and selects expressed genes with significant between-species differences [26].

### Cross-species correlation of genome-wide expression

Unlike the analysis performed for comparing cross-species DHS sites, we did not liftOver any non-human expression sequences to human. Instead, we simply compared the DGE-seq sequence counts that aligned to exonic regions within each species' native sequence alignment (Supplemental data file 5 in Dataset S1). Because of the high level of homology of the exonic regions between the 3 primate species, we directly compared tag count numbers between each of the orthologous genes. For the genome-wide expression correlation comparison (Figure 1), we normalized tag counts using edgeR to calculate the total library size for each sample and adjusting the tag counts accordingly so that relative differences between the depth of the sequencing reads did not influence the results. Next, we filtered out genes that did not have at least 10 combined tag counts between all of the samples to decrease the noise associated with genes that fall below the meaningful level as recommended for edgeR. Following these filters, we compared the Spearman correlation values between all of the samples and plotted the results as a heatmap with hierarchical clustering to show similarities within and between species and tissue types.

### Identification of differentially expressed genes between species

Using edgeR, we identified genes that were significantly differentially expressed between the 3 primate species. Comparisons between species were performed on a pairwise manner comparing 3 individuals of one species against 3 individuals from a second species. The same normalization method and filters used in the expression correlation analysis was also used prior to defining the differentially expressed genes. Differential gene expression was defined as genes having a *P* value cutoff of 0.05. Using the Macaque expression result as an outgroup, we identified genes that displayed high or low expression specifically on the human and chimp branch. For example, genes we defined as highly expressed in human (human upregulated genes) are differentially expressed in both human/chimp and human/macaque comparisons, but not differentially expressed in the chimp/macaque comparison. Similar criteria were used to identify genes that display low expression in human compared to chimp and macaque (human downregulated genes).

### Correlation of species-specific DHS site gains and losses with species-specific gene expression gains and losses

To firmly establish the connection between differential chromatin and differential expression, we tested for significance in overlap in both directions: First, we tested if differential DHS sites tend to be located near differentially expressed genes, and second, we tested if differentially expressed genes tend to have differential DHS sites nearby.

#### Comparing differential DNase-seq signal to differential gene expression

We compared human DHS gain, human DHS loss, chimpanzee DHS gain, and chimpanzee DHS loss regions to species-specific genes (e.g., human upregulated/downregulated genes, etc.). We first assigned each DHS site its nearest gene, then intersected these with each differential expression set. The number of overlaps for every pairwise combination of DHS sites vs. differentially expressed genes are shown in Table S5a. We calculated *P* values by permuting (100,000 times) the set of all expressed Fibroblast genes, randomly selecting the number of genes for each comparison, and intersecting those random sets with the genes nearest species-specific DHS sites (Table S7b–S7c). This established a null distribution of overlaps in random intersects.

#### Comparing differential gene expression to differential DHS sites

To establish the connection in the opposite direction, we did a similar analysis in the reverse direction. Using the UCSC knownGene table, we merged all isoform coordinates for each gene and found all DHS sites within a surrounding 20 kb window. We calculated pairwise overlaps of DHS sites between each differential DHS site list and these lists of all nearby DHS sites. If a gene contained a differential DHS site within 20 kb, it was counted as a match. The number of overlaps for every pairwise combination of sites near differentially expressed genes vs. differential DHS sites are shown in Table S6a. We then permuted genes similar to above (1,000 times), located all nearby DHS sites, and tested for overlap to create a null distribution of overlap count (Table S8b–S8c).

### Identification of mutations in motifs associated with species-specific DHS sites

To connect sequence changes to species-specific DHS sites, we compared JASPAR motif scores across species. We first extracted the orthologous DNA sequences for human, chimpanzee, and macaque for each of our DHS gain, loss, and common sites. We scanned and scored each of these sequences for all the position weight matrices (PWMs) in the JASPAR database. We scored a sequence for a given PWM as the highest motif score anywhere in that sequence. This resulted in a region-by-motif matrix of scores; each score is the highest score for each motif/sequence combination. To compare species, we took the log ratio of scores (human/chimp, human/macaque, and chimp/macaque). Where this score is 0, the highest score does not differ between species. Deviations from 0 indicate the direction of improvement in motif match (in a human/chimp comparison, a positive number means the best match in the human sequence scored higher than the best match in the chimp sequence). After calculating these scores and each pairwise log-ratio, we plotted the log-ratios (Supplemental data files 2–3 in Dataset S1 and Figure 7) to examine trends. We calculated the pairwise log-ratios for multiple species comparisons and plotted these on different axis to check whether increases over one species correlate with increases over the other. In these two-dimensional plots, each axis quantifies a different pairwise species relationship. Points that cluster in the upper-right quadrant have higher scores than either of the other species; points that cluster in the lower-left have lower scores. The most interesting variation in these plots is whether the points congregate in the upper-right or lower left. To assess significance, we project each data point onto the y = x line to reduce the dimensionality to 1. We then used the Wilcoxson rank-sum test to compare each distribution to the distribution of the common regions (Figure 7g and Supplemental data file 3 in Dataset S1).

## Supporting Information

Dataset S1 Supplemental data file 1. Species-specific DHS Gains, Losses, and Common regions. Excel file containing coordinates (hg19) of differential DHS gains, DHS losses, DHS common regions, as well as a list of upregulated, downregulated, and commonly expressed genes (UCSC gene symbols). A BED format (non-Excel) version of the same datasets are also included (SupplementalDataFile1.zip). Supplemental data file 2. Motif analysis boxplots. Boxplots showing pairwise comparisons of log ratios of the best position weight matrix (PWM) in a DHS Gain/Loss/Common site between each species. Motifs for 130 transcription factors are represented on the X-axis. The Y-axis shows the ratio of the PWM score change for all DHS sites in the set of regions (DHS Gains/Losses/Common) being compared for each TF. Supplemental data file 3. Motif analysis scatterplots. Scatterplots showing the level of enrichment of a transcription factor motif in each species to the level of DNase hypersensitivity. One hundred and thirty TFs were analyzed separately. Supplemental data file 4. BED file containing sequence counts for all DHS regions used for differential DHS analysis. Supplemental data file 5. Excel file containing sequence counts for all genes captured by DGE-seq used for differential Expression analysis.
(GZ) Click here for additional data file.

Figure S1 Representative examples of chimpanzee DHS gain and chimpanzee DHS loss.
(PDF)

Figure S2 Species-specific gains and common DHS sites are highly reproducible in independently isolated matched cell types. (a) Human DHS gains/losses, Chimp DHS gains/losses, and Common DHS sites detected in fibroblast cells were compared to independently derived ENCODE Fibroblast DNase-seq data generated by the University of Washington ENCODE group. (b) LCL human DHS gains/losses/common compared to 4 independently derived ENCODE lymphoblastoid samples also identified by the University of Washington ENCODE group.
(PDF)

Figure S3 Differential edgeR analysis of original human and chimpanzee samples to independently analyzed cell samples. Similar numbers of human gains and losses are identified when comparing independently derived human fibroblasts (a) and LCLs (b) to chimpanzee fibroblasts and LCLs. Very few differentially open chromatin regions were identified by edgeR when comparing the 3 original human samples to 3 independently derived human fibroblast and LCL samples.
(PDF)

Figure S4 Box plot showing DNase intensity of human DHS gains/losses and common regions across 27 human cell types shown in Figure 4 heatmaps.
(PDF)

Figure S5 Boxplot of the binary comparison of human DHS gain/loss, chimpanzee DHS gain/loss, and Common regions to the DHS peak calls from the 27 other human cell types.
(PDF)

Figure S6 Comparison of chimpanzee DHS gains and DHS losses to DNase-seq data from other human cell types. These regions were compared to DNase-seq data generated from 27 other human cell types (Table S3). Heatmap signal intensities are of maximum DNase-seq parzen scores in log scale, where red indicates a higher DNase-seq score and blue indicates lower DNase-seq scores. (a) Chimpanzee DHS sites were identified as differentially open (chimpanzee DHS gain) in chimpanzee fibroblasts compared to human/macaque fibroblasts. (b) Chimpanzee DHS sites identified as differentially closed (Chimpanzee DHS loss) compared to human and macaque fibroblasts. (c) DNase-seq signal values for Common regions representing DHS sites in all three species. Note that more than 50% of Common regions are also DHS sites in other human tissues. (d, e, f) DNase-seq signal values for same regions as (a, b, c), but DNase-seq data is from orthologous region from human and macaque fibroblasts. (g, h, i) DNase-seq values for same regions as (a, b, c), but from human and chimpanzee LCLs. (bottom) Box plot shows intensity values shown in heatmaps.
(TIF)

Figure S7 Species-specific upregulated/downregulated gene expression levels are correlated with species-specific DHS gains/losses, respectively. (a) 48 Human upregulated genes (HumanExpUp) intersect genes that were located closest to human DHS gains (yellow arrow), which is higher than random permutations. 6 Human upregulated genes (HumanExpUp) overlap with genes located closest to human DHS losses (blue arrow), which is less than random permutations. (b) Comparison of upregulated genes (expression gains) and downregulated genes (expression losses) to genes located nearest to DHS gains and losses. *P* values were derived from 1000 random permutations (Materials and Methods).
(PDF)

Figure S8 Comparison of chromatin DHS gains and DHS losses with genes that are upregulated and downregulated in human vs. chimpanzee lymphoblastoid cell lines (LCLs). Yellow represents chromatin and expression matches that occur more often than random permutations, while blue represent less often. *P* value indicated in each box.
(PDF)

Figure S9 Phastcons figure showing sequence conservation. Histograms comparing the distribution of maximum PhastCons scores for pooled DHS gains (both human- and chimp-specific increases), pooled DHS losses (both human- and chimp-specific decreases), and Common regions. We pooled regions because the distributions look similar when divided (data not shown). Common regions have higher PhastCons scores. Average PhastCons scores show similar trends (data not shown).
(PDF)

Figure S10 Potential regulatory element shuffling: Number of instances (within 50 k) where fibroblast human DHS gain, human DHS loss, chimpanzee DHS gain, chimpanzee DHS loss, or Common regions map other nearby species-specific gains, losses, or common regions. This plot shows the number of DHS sites that fall within 50 kb of each other for each pairwise comparison. It also shows a *P* value for the significance of the overlap. To calculate the *P* values (upper number in each box), we used a permutation test to compare the number of true overlaps (lower number in each box) to the number of expected overlaps with a random set of DHS sites of matching size. Significant enrichments are bright yellow and depletions bright blue, with both significance and count shown in the appropriate box. The significance levels are limited by the number of permutations (1000), so a 0 indicates a permutation P value <10∧-3. We find insignificant or only marginally significant overlaps in most comparisons; for example, comparing human DHS gains to human DHS losses: human DHS gains are not located near human DHS losses more often than expected by chance. The same is true for chimp gains and losses. However, we do see more significance when comparing human DHS gains to chimp DHS losses, and chimp DHS gains to human DHS losses. This indicates that these regions tend to be located near one another more often than expected by chance.
(PDF)

Figure S11 Potential regulatory element shuffling associated with obesity. Shown is a human DHS gain nearby a human DHS loss between the PMAIP1 and melanocortin 4 receptor (MC4R). MC4R is a gene shown to cause autosomal dominant obesity.
(PDF)

Figure S12 Potential regulatory element shuffling associated with an imprinted gene. GNAS is an imprinted gene that is regulated by the GNAS1 antisense transcript. A human DHS gain is located at the 3′ end of the GNAS1 gene, and human DHS loss in the intron of GNAS1 (upstream of GNAS).
(PDF)

Figure S13 Potential regulatory element shuffling associated with alternative promoter and glial cell formation. Human DHS loss associated with meteorin-like (METRNL), a gene associated with glial cell formation. Nearby is a human DHS gain near an alternative promoter of the BG3NTL1, a gene with putative glycosyltransferase activity. Also note a great ape specific DHS between METRNL/BG2NTL1 genes (present in chimpanzee/human, but absent in macaque).
(PDF)

Figure S14 Potential case of an independently gained human and chimpanzee DHS sites associated with neuronal guidance. Human DHS gain found nearby (<50 kb) a chimpanzee DHS gain. Both mapped within an intron of the SRGAP2, a gene associated with neuronal guidance during brain development.
(PDF)

Figure S15 Chimp Condel (cCondel) regions heatmap. Shown are DNase-seq signal intensities (maximum parzen scores) of the 344 cCONDEL regions. The color scale (log) ranges from red, representing the highest signal intensities, down to black, representing little/no signal intensity. All 15 primate samples (fibroblasts and LCLs) used in our DNase-seq analysis and 27 different ENCODE cell lines are represented. Ubiquitous DHS sites that overlap cCONDELs are clustered on the right, and more common DHS sites are found in the middle. Note that these regions are do not have signal in chimpanzee data (C_L or C_F samples), and a small number also do not contain signal in macaque data (Q_F samples).
(PDF)

Table S1 Samples used for DNase-seq and DGE-seq analyses. Basic sequence count statistics are provided with each sample.
(PDF)

Table S2 DNase-seq data is validated by DNase-chip. DNase-chip libraries from the 15 samples were hybridized to 1% ENCODE DNA arrays of the corresponding species type. Probes consisted of segments of DNA sequences matching reference sequence builds hg18 (human), panTro2 (chimpanzee), and rheMac2 (macaque), respectively. The array intensities were compiled and significant DHS sites were called using ChIPotle (*P*<0.000001 peak cutoff). We intersected the DNase-chip sites from each of the 3 individuals samples for each species and determined the amount that overlap DNase-seq data. The top 100,000 F-seq called sites were used from each DNase-seq sample for this comparison.
(PDF)

Table S3 Human cell types analyzed by DNase-seq for the ENCODE project by our group. All data is available on the UCSC genome browser (http://genome.ucsc.edu/) and more specifically at: http://genome.ucsc.edu/cgi-bin/hgTrackUi?hgsid=246298273&c=chr21&g=wgEncodeOpenChromDnase
(PDF)

Table S4 Percent overlap of human- and chimpanzee- DHS gains/losses/common detected in fibroblasts with DHS sites identified in ENCODE human cell types. FibroP, Fibrobl, and ProgFib cells were independently derived fibroblasts samples.
(PDF)

Table S5 Percent overlap of human-DHS gains/losses/common detected in lymphoblasts with DHS sites identified in ENCODE human cell types. Note high degree of overlap with independently derived LCLs (GM).
(PDF)

Table S6 Fisher's exact test for Histone modification and CTCF ChIP-seq peaks that intersect LCL Human DHS gains/losses/commons.
(PDF)

Table S7 Relationship between species-specific DHS sites and differentially expressed genes. Species-specific DHS sites (Human DHS gain/loss and Chimpanzee DHS gain/loss regions) for the fibroblast cell type were compared to the expression state of the closest gene (human up/down and chimp up/down expressed genes).
(PDF)

Table S8 Relationship between differentially expressed species-specific genes (human DHS gains/losses, chimpanzee DHS gains/losses) and the likelihood of finding a species-specific DHS site within a 20 kb window surrounding those genes.
(PDF)

Table S9 Gene Ontology enrichments associated with species-specific DHS gain/loss and common regions and human up- and down- regulated genes. (a) Gene ontology analysis tool, GREAT, was used to look for any enrichments of biological process or disease ontologies of genes associated with human and chimpanzee DHS gain/loss/common regions. (b) Genes identified as differentially expressed in human or chimpanzee fibroblast samples were analyzed for GO biological process enrichments using DAVID. A *P* value threshold of 0.001 was set as the minimum.
(PDF)

Table S10 Testing for selection, *P* values (Two-tailed Mann-Whitney test). To test significance, for a given set of regions (Fibroblast: human DHS gain, human DHS loss, chimpanzee DHS gain, chimpanzee DHS loss, Common; LCLs: LCL human DHS gain, LCL human DHS loss, Common) we ordered the *P* values for selection on both hg19 and panTro2, then did a Mann-Whitney test to see if one branch has higher *P* values than the other.
(PDF)

Table S11 The most rapidly evolving Conserved Non-coding Sequences (CNSs) minimally overlap species-specific DHS gains and losses. Human Accelerated Regions (HARs) and Chimpanzee CONDELs (cCONDELs) did not overlap any species-specific DHS gains, losses, or common regions. (a) Genome coordinates of Human Accelerated Conserved Non-coding Sequences (HACNSs) that overlapped 1 human-specific DHS loss and 1 common DHS region identified in fibroblasts. (b) Genome coordinates of Chimp Accelerated Conserved Non-coding Sequences (CACNSs) that overlapped 1 chimp-specific DHS losses and 1 common DHS region. (c) Percent of HACNSs, CACNSs, HARs, and cCONDELs that overlap the top 100 k DHS peaks (defined by p-value) from fibroblast and LCL samples in hg19 space. (d) Percent of Human CONDELs (hCONDELs) that overlap the top 100 k DHS peaks (defined by *P* value) from fibroblast and LCL samples in panTro2 space. (e) The number of HACNSs, CACNSs, HARs, and cCONDELs that overlap DHS sites identified in 27 diverse cell lines is consistently low.
(PDF)

Table S12 Genes located closest to human-specific DHS gain/loss and common regions as well as genes identified as differentially expressed among the 3 primate species are not highly associated with genes used to identify Fibroblast biopsy locations in Rinn et al. 2006 PLoS Genetics [30].
(PDF)
