# Supplementary material for: Extensive Evolutionary Changes in Regulatory Element Activity during Human Origins Are Associated with Altered Gene Expression and Positive Selection
Source: PLoS Genet. 2012 Jun 28;8(6):e1002789. doi: 10.1371/journal.pgen.1002789 (PMC3386175; doi:10.1371/journal.pgen.1002789)
Supplement: Dataset S1 — Supplemental data file 1. Species-specific DHS Gains, Losses, and Common regions. Excel file containing coordinates (hg19) of differential DHS gains, DHS losses, DHS common regions, as well as a list of upregulated, downregulated, and commonly expressed genes (UCSC gene symbols). A BED format (non-Excel) version of the same datasets are also included (SupplementalDataFile1.zip). Supplemental data file 2. Motif analysis boxplots. Boxplots showing pairwise comparisons of log ratios of the best position weight matrix (PWM) in a DHS Gain/Loss/Common site between each species. Motifs for 130 transcription factors are represented on the X-axis. The Y-axis shows the ratio of the PWM score change for all DHS sites in the set of regions (DHS Gains/Losses/Common) being compared for each TF. Supplemental data file 3. Motif analysis scatterplots. Scatterplots showing the level of enrichment of a transcription factor motif in each species to the level of DNase hypersensitivity. One hundred and thirty TFs were analyzed separately. Supplemental data file 4. BED file containing sequence counts for all DHS regions used for differential DHS analysis. Supplemental data file 5. Excel file containing sequence counts for all genes captured by DGE-seq used for differential Expression analysis. (GZ) [file pgen.1002789.s001.gz › ./supplemental_data_files/supplemental_data_file_2_boxplots.pdf]

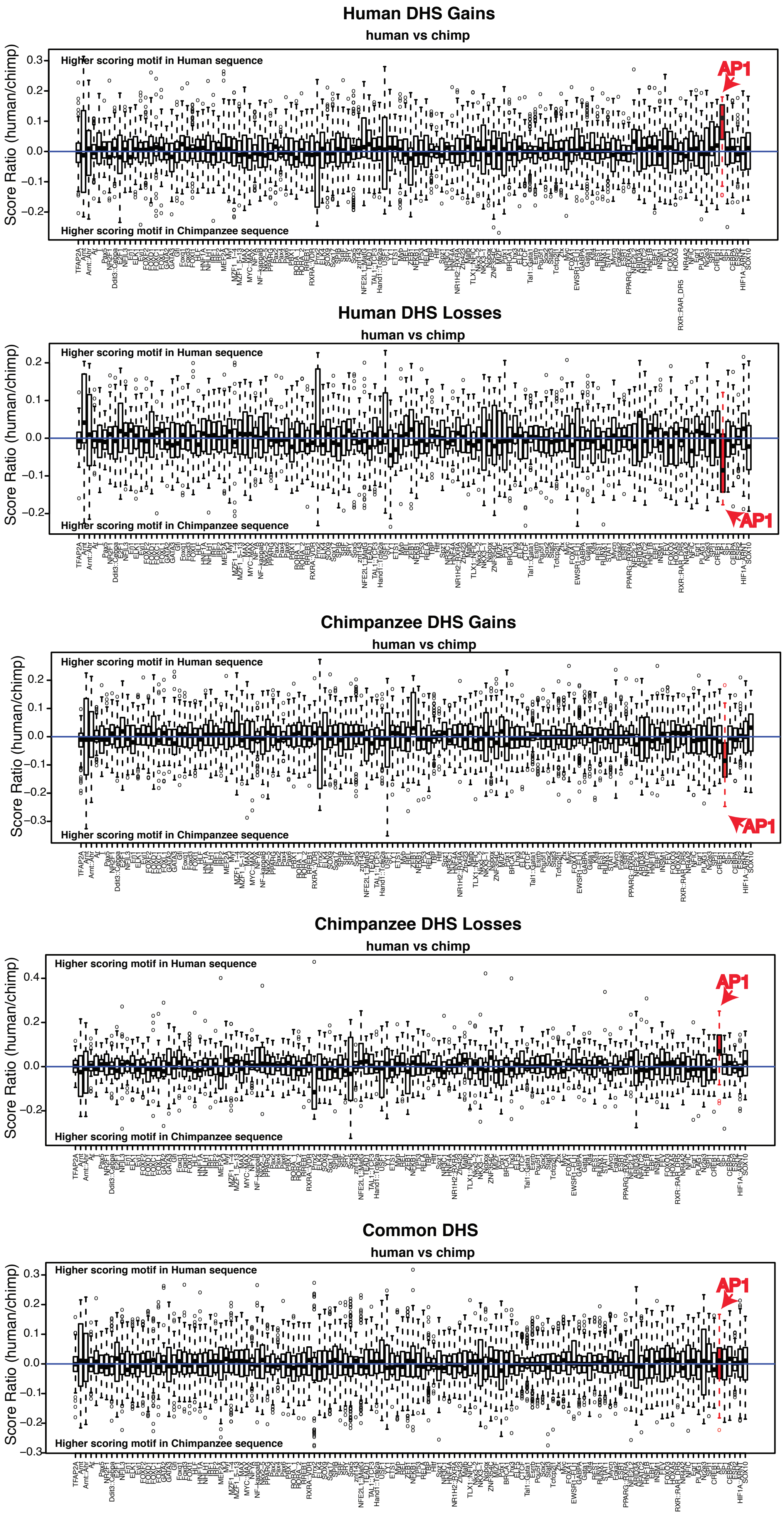
Human DHS Gains
human vs chimp
Higher scoring motif in Human sequence
Higher scoring motif in Chimpanzee sequence
Score Ratio (human/chimp)
AP1
Human DHS Losses
human vs chimp
Higher scoring motif in Human sequence
Higher scoring motif in Chimpanzee sequence
Score Ratio (human/chimp)
AP1
Chimpanzee DHS Gains
human vs chimp
Higher scoring motif in Human sequence
Higher scoring motif in Chimpanzee sequence
Score Ratio (human/chimp)
AP1
Chimpanzee DHS Losses
human vs chimp
Higher scoring motif in Human sequence
Higher scoring motif in Chimpanzee sequence
Score Ratio (human/chimp)
AP1
Common DHS
human vs chimp
Higher scoring motif in Human sequence
Higher scoring motif in Chimpanzee sequence
Score Ratio (human/chimp)
AP1

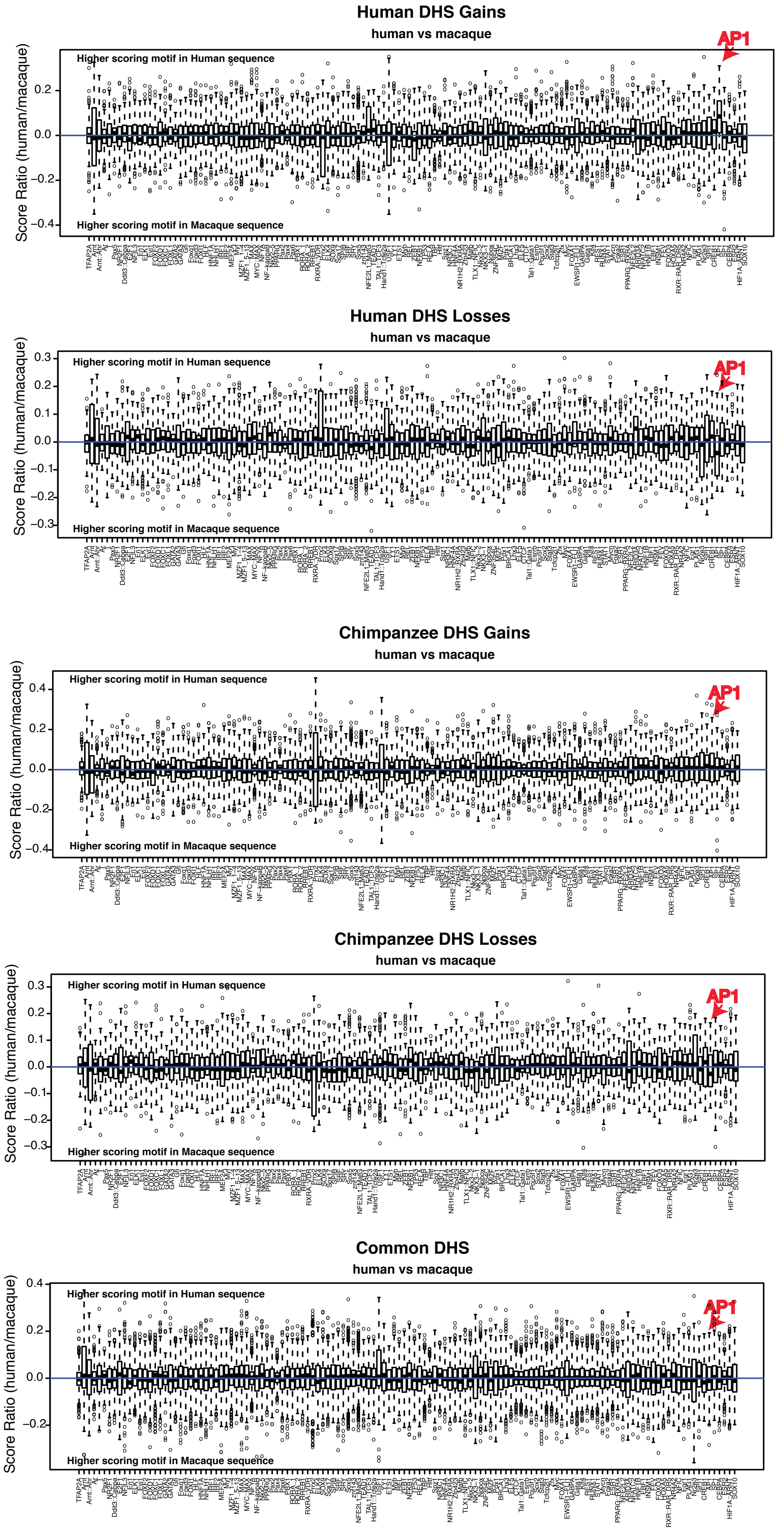
Human DHS Gains
human vs macaque
Higher scoring motif in Human sequence
Higher scoring motif in Macaque sequence
AP1
Score Ratio (human/macaque)
Human DHS Losses
human vs macaque
Higher scoring motif in Human sequence
Higher scoring motif in Macaque sequence
AP1
Score Ratio (human/macaque)
Chimpanzee DHS Gains
human vs macaque
Higher scoring motif in Human sequence
Higher scoring motif in Macaque sequence
AP1
Score Ratio (human/macaque)
Chimpanzee DHS Losses
human vs macaque
Higher scoring motif in Human sequence
Higher scoring motif in Macaque sequence
AP1
Score Ratio (human/macaque)
Common DHS
human vs macaque
Higher scoring motif in Human sequence
Higher scoring motif in Macaque sequence
AP1
Score Ratio (human/macaque)

3

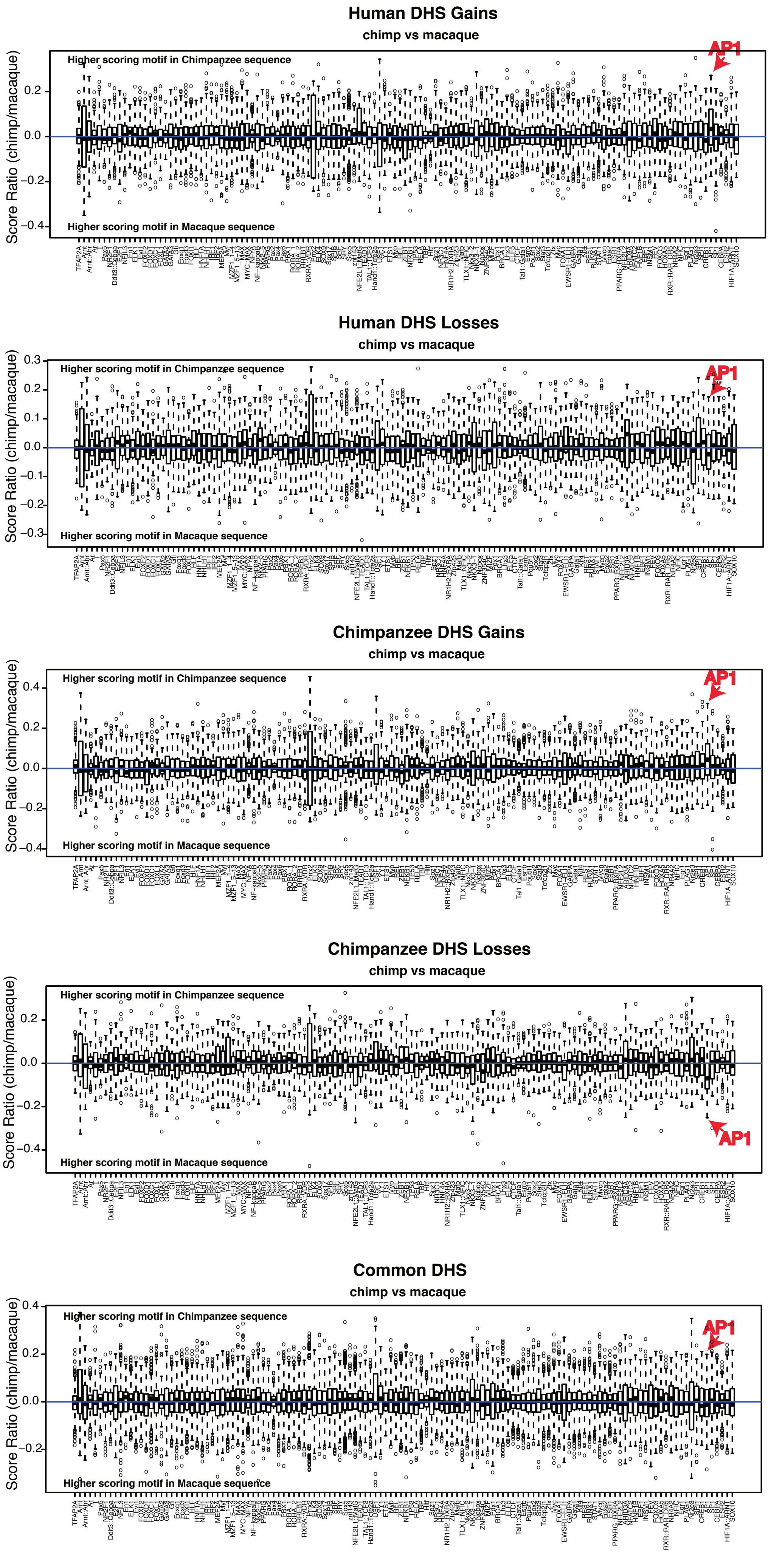
Human DHS Gains
chimp vs macaque
Higher scoring motif in Chimpanzee sequence
Higher scoring motif in Macaque sequence
Score Ratio (chimp/macaque)
AP1
Human DHS Losses
chimp vs macaque
Higher scoring motif in Chimpanzee sequence
Higher scoring motif in Macaque sequence
Score Ratio (chimp/macaque)
AP1
Chimpanzee DHS Gains
chimp vs macaque
Higher scoring motif in Chimpanzee sequence
Higher scoring motif in Macaque sequence
Score Ratio (chimp/macaque)
AP1
Chimpanzee DHS Losses
chimp vs macaque
Higher scoring motif in Chimpanzee sequence
Higher scoring motif in Macaque sequence
Score Ratio (chimp/macaque)
AP1
Common DHS
chimp vs macaque
Higher scoring motif in Chimpanzee sequence
Higher scoring motif in Macaque sequence
Score Ratio (chimp/macaque)
AP1
