# Supplementary material for: Extensive Evolutionary Changes in Regulatory Element Activity during Human Origins Are Associated with Altered Gene Expression and Positive Selection
Source: PLoS Genet. 2012 Jun 28;8(6):e1002789. doi: 10.1371/journal.pgen.1002789 (PMC3386175; doi:10.1371/journal.pgen.1002789)
Supplement: Dataset S1 — Supplemental data file 1. Species-specific DHS Gains, Losses, and Common regions. Excel file containing coordinates (hg19) of differential DHS gains, DHS losses, DHS common regions, as well as a list of upregulated, downregulated, and commonly expressed genes (UCSC gene symbols). A BED format (non-Excel) version of the same datasets are also included (SupplementalDataFile1.zip). Supplemental data file 2. Motif analysis boxplots. Boxplots showing pairwise comparisons of log ratios of the best position weight matrix (PWM) in a DHS Gain/Loss/Common site between each species. Motifs for 130 transcription factors are represented on the X-axis. The Y-axis shows the ratio of the PWM score change for all DHS sites in the set of regions (DHS Gains/Losses/Common) being compared for each TF. Supplemental data file 3. Motif analysis scatterplots. Scatterplots showing the level of enrichment of a transcription factor motif in each species to the level of DNase hypersensitivity. One hundred and thirty TFs were analyzed separately. Supplemental data file 4. BED file containing sequence counts for all DHS regions used for differential DHS analysis. Supplemental data file 5. Excel file containing sequence counts for all genes captured by DGE-seq used for differential Expression analysis. (GZ) [file pgen.1002789.s001.gz › ./supplemental_data_files/supplemental_data_file_3_scatterplot.pdf]

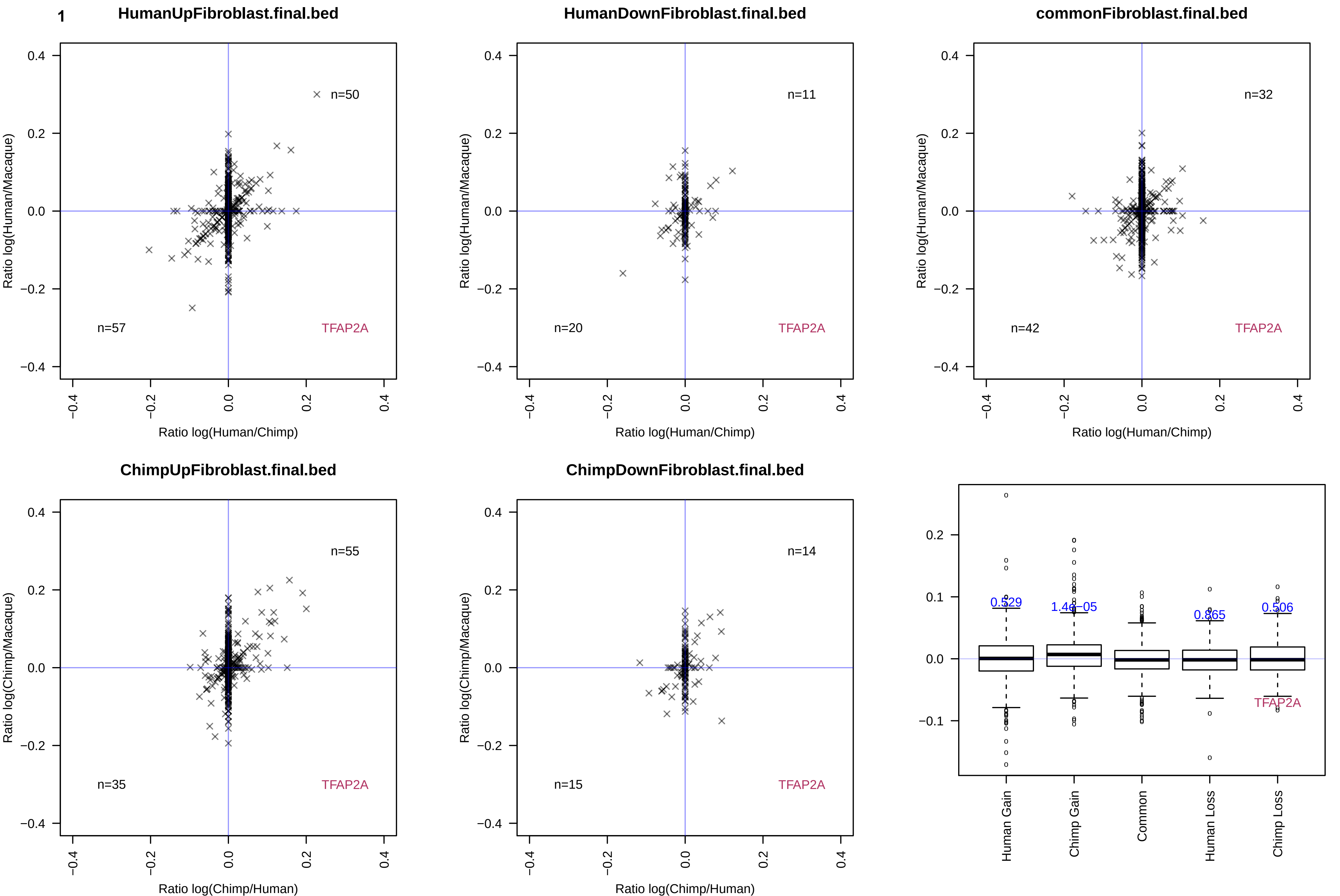
1
HumanUpFibroblast.final.bed
n=50
n=57
TFAP2A
Ratio log(Human/Chimp)
Ratio log(Human/Macaque)
HumanDownFibroblast.final.bed
n=11
n=20
TFAP2A
Ratio log(Human/Chimp)
Ratio log(Human/Macaque)
commonFibroblast.final.bed
n=32
n=42
TFAP2A
Ratio log(Human/Chimp)
Ratio log(Human/Macaque)
ChimpUpFibroblast.final.bed
n=55
n=35
TFAP2A
Ratio log(Chimp/Human)
Ratio log(Chimp/Macaque)
ChimpDownFibroblast.final.bed
n=14
n=15
TFAP2A
Ratio log(Chimp/Human)
Ratio log(Chimp/Macaque)
0.529
1.46e−05
0.865
0.506
TFAP2A
Human Gain
Chimp Gain
Common
Human Loss
Chimp Loss

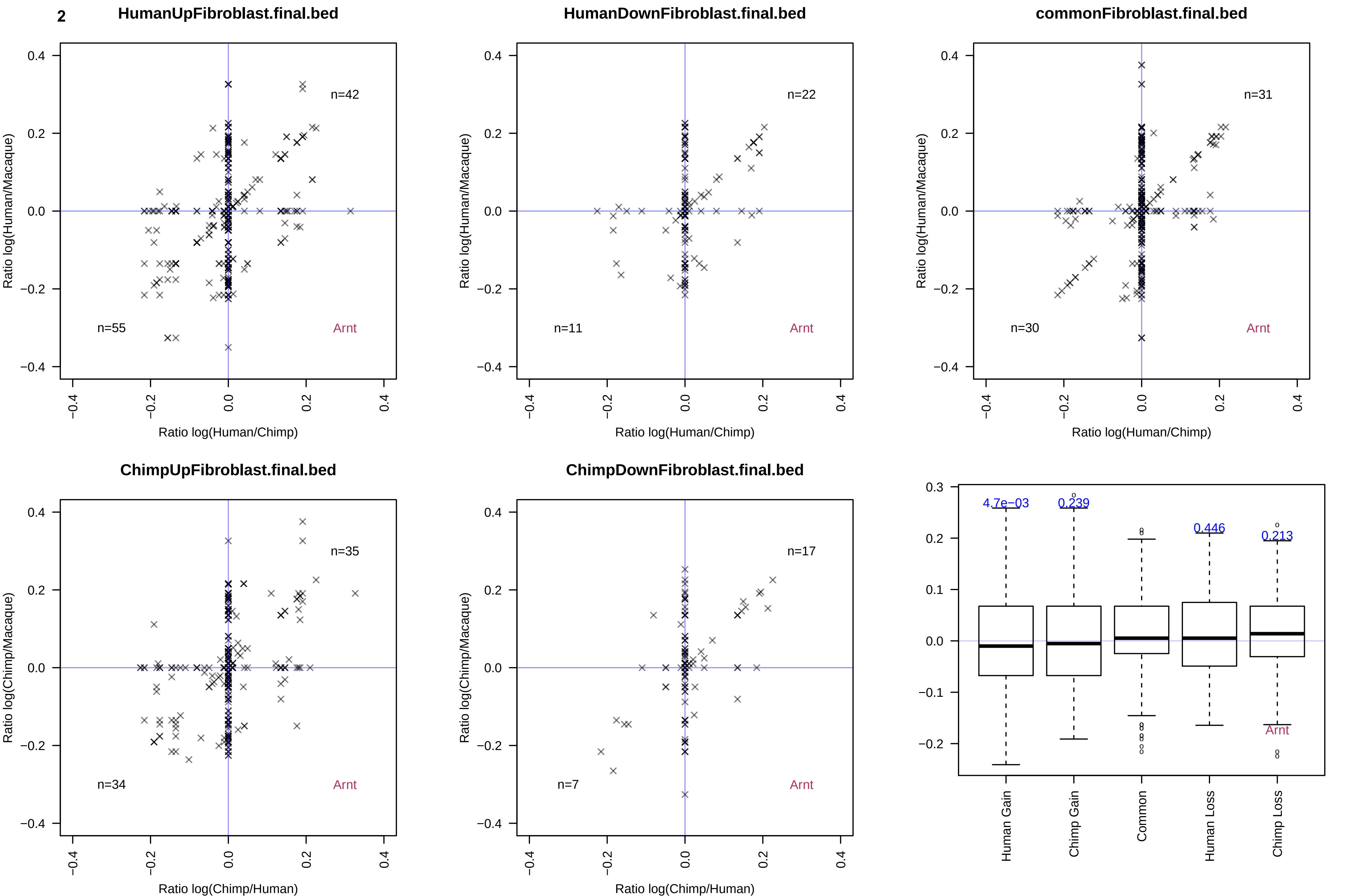

2
HumanUpFibroblast.final.bed
n=42
n=55
Arnt
HumanDownFibroblast.final.bed
n=22
n=11
Arnt
commonFibroblast.final.bed
n=31
n=30
Arnt
Ratio log(Human/Macaque)
Ratio log(Human/Chimp)
ChimpUpFibroblast.final.bed
n=35
n=34
Arnt
ChimpDownFibroblast.final.bed
n=17
n=7
Arnt
Ratio log(Chimp/Macaque)
Ratio log(Chimp/Human)
4.7e−03
0.239
0.446
0.213
Arnt
Human Gain
Chimp Gain
Common
Human Loss
Chimp Loss

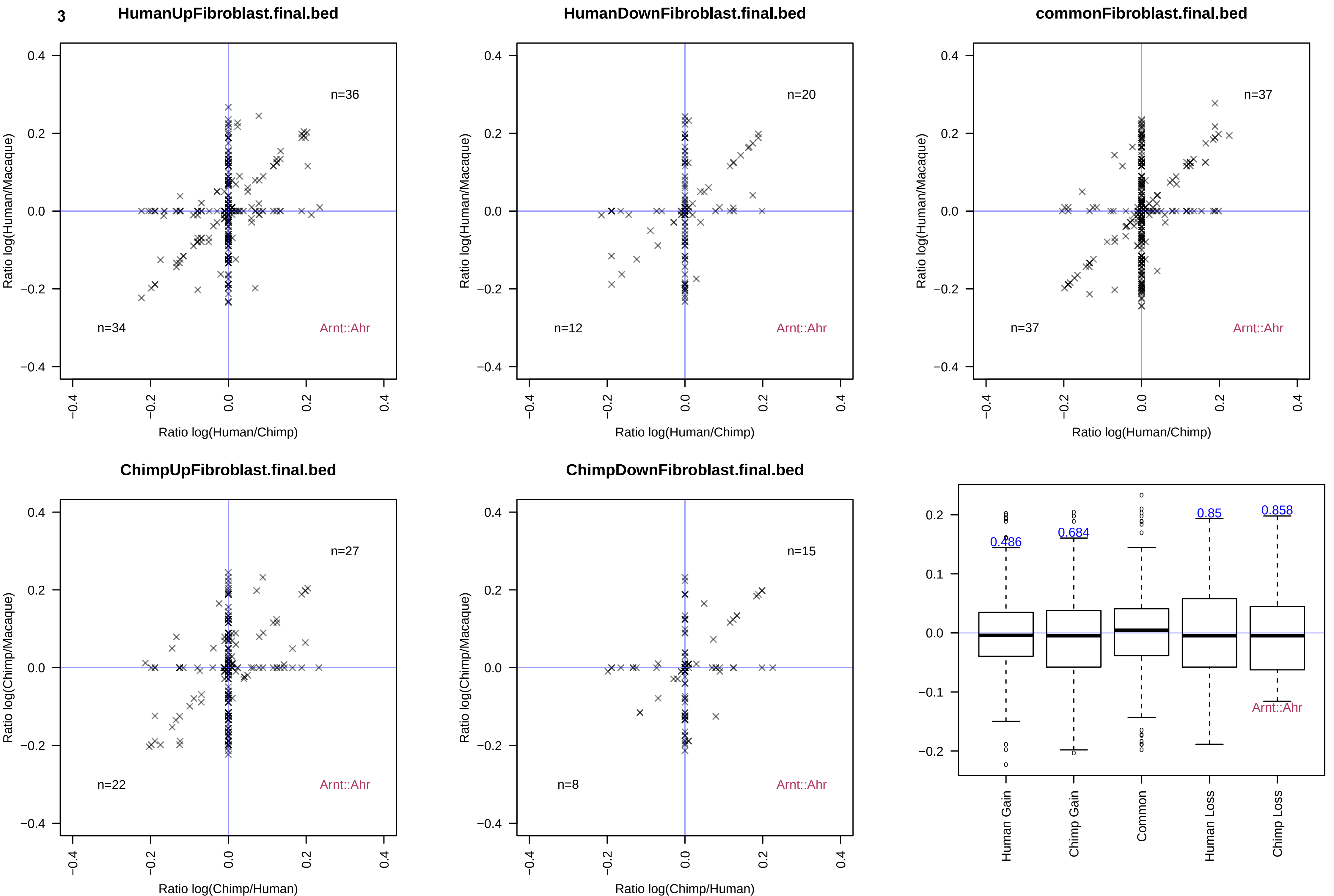
HumanUpFibroblast.final.bed
n=36
n=34
Arnt::Ahr
Ratio log(Human/Macaque)
Ratio log(Human/Chimp)
HumanDownFibroblast.final.bed
n=20
n=12
Arnt::Ahr
Ratio log(Human/Macaque)
Ratio log(Human/Chimp)
commonFibroblast.final.bed
n=37
n=37
Arnt::Ahr
Ratio log(Human/Macaque)
Ratio log(Human/Chimp)
ChimpUpFibroblast.final.bed
n=27
n=22
Arnt::Ahr
Ratio log(Chimp/Macaque)
Ratio log(Chimp/Human)
ChimpDownFibroblast.final.bed
n=15
n=8
Arnt::Ahr
Ratio log(Chimp/Macaque)
Ratio log(Chimp/Human)
0.486
0.684
0.85
0.858
Arnt::Ahr
Human Gain
Chimp Gain
Common
Human Loss
Chimp Loss

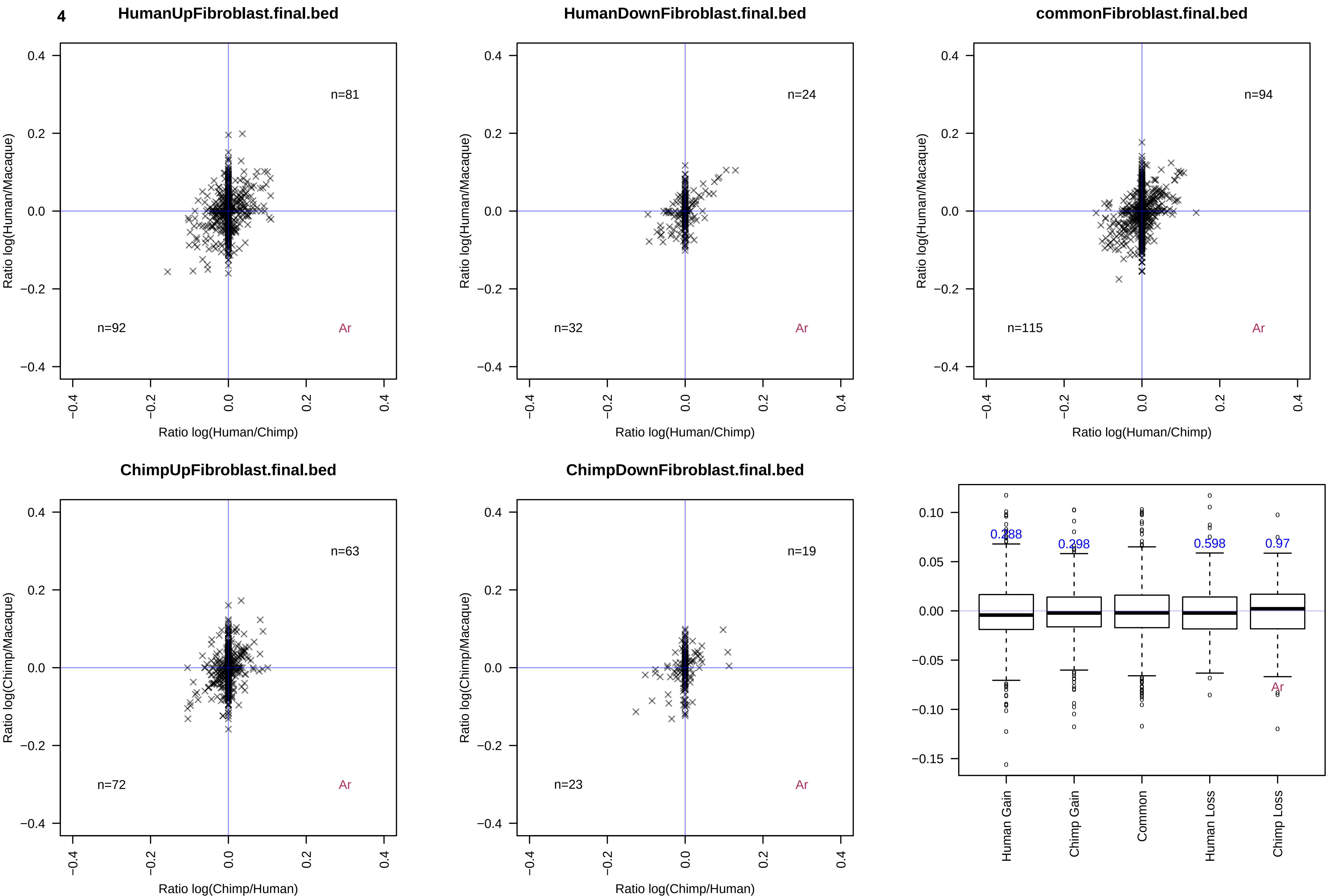
HumanUpFibroblast.final.bed
n=81
n=92
Ar
HumanDownFibroblast.final.bed
n=24
n=32
Ar
commonFibroblast.final.bed
n=94
n=115
Ar
Ratio log(Human/Macaque)
Ratio log(Human/Chimp)
ChimpUpFibroblast.final.bed
n=63
n=72
Ar
ChimpDownFibroblast.final.bed
n=19
n=23
Ar
Ratio log(Chimp/Macaque)
Ratio log(Chimp/Human)
0.288
0.298
0.598
0.97
Ar
Human Gain
Chimp Gain
Common
Human Loss
Chimp Loss

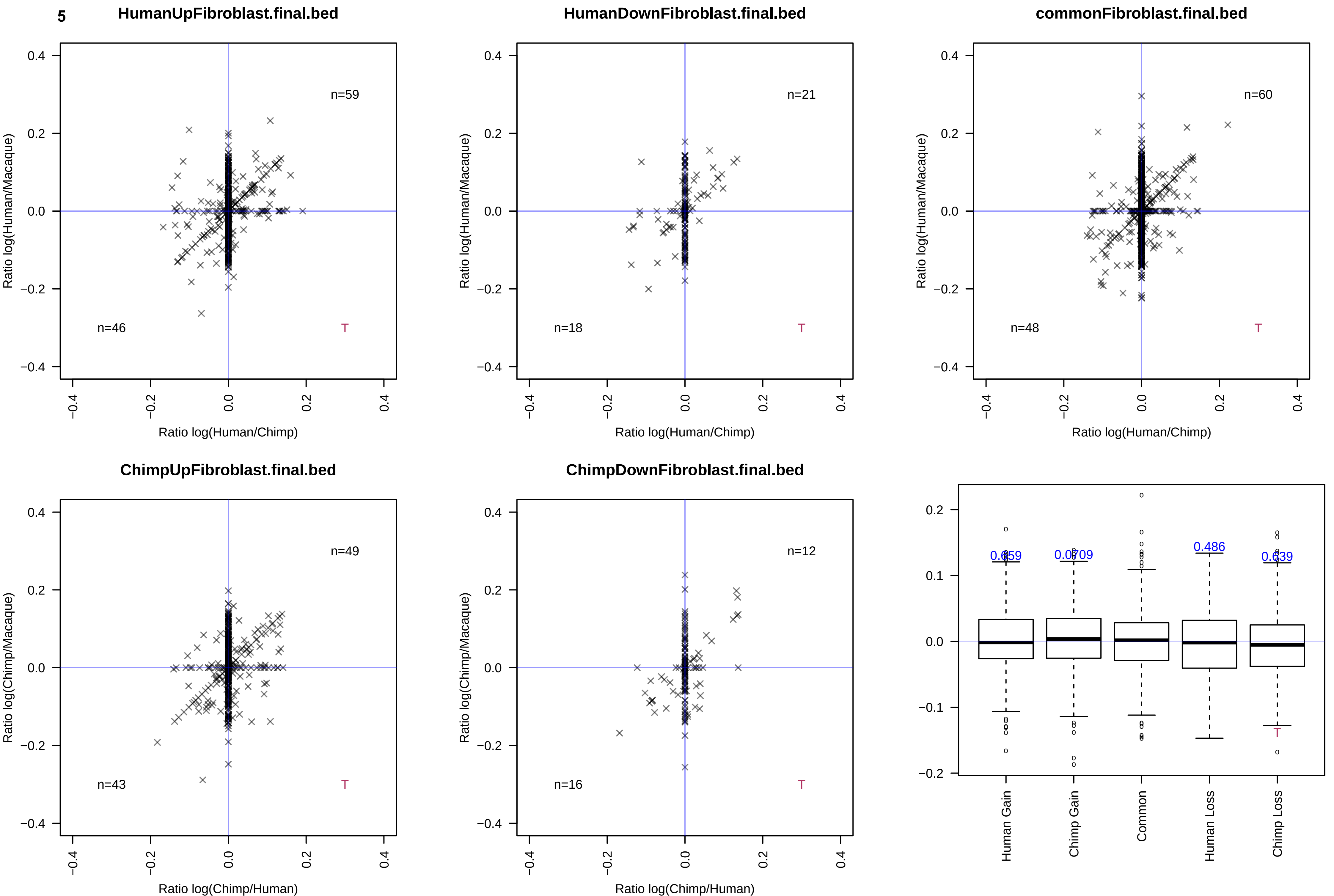
5
HumanUpFibroblast.final.bed
HumanDownFibroblast.final.bed
commonFibroblast.final.bed
ChimpUpFibroblast.final.bed
ChimpDownFibroblast.final.bed
Ratio log(Human/Macaque)
Ratio log(Human/Chimp)
Ratio log(Chimp/Macaque)
Ratio log(Chimp/Human)
n=59
n=46
n=21
n=18
n=60
n=48
n=49
n=43
n=12
n=16
T
0.659
0.0709
0.486
0.639
Human Gain
Chimp Gain
Common
Human Loss
Chimp Loss

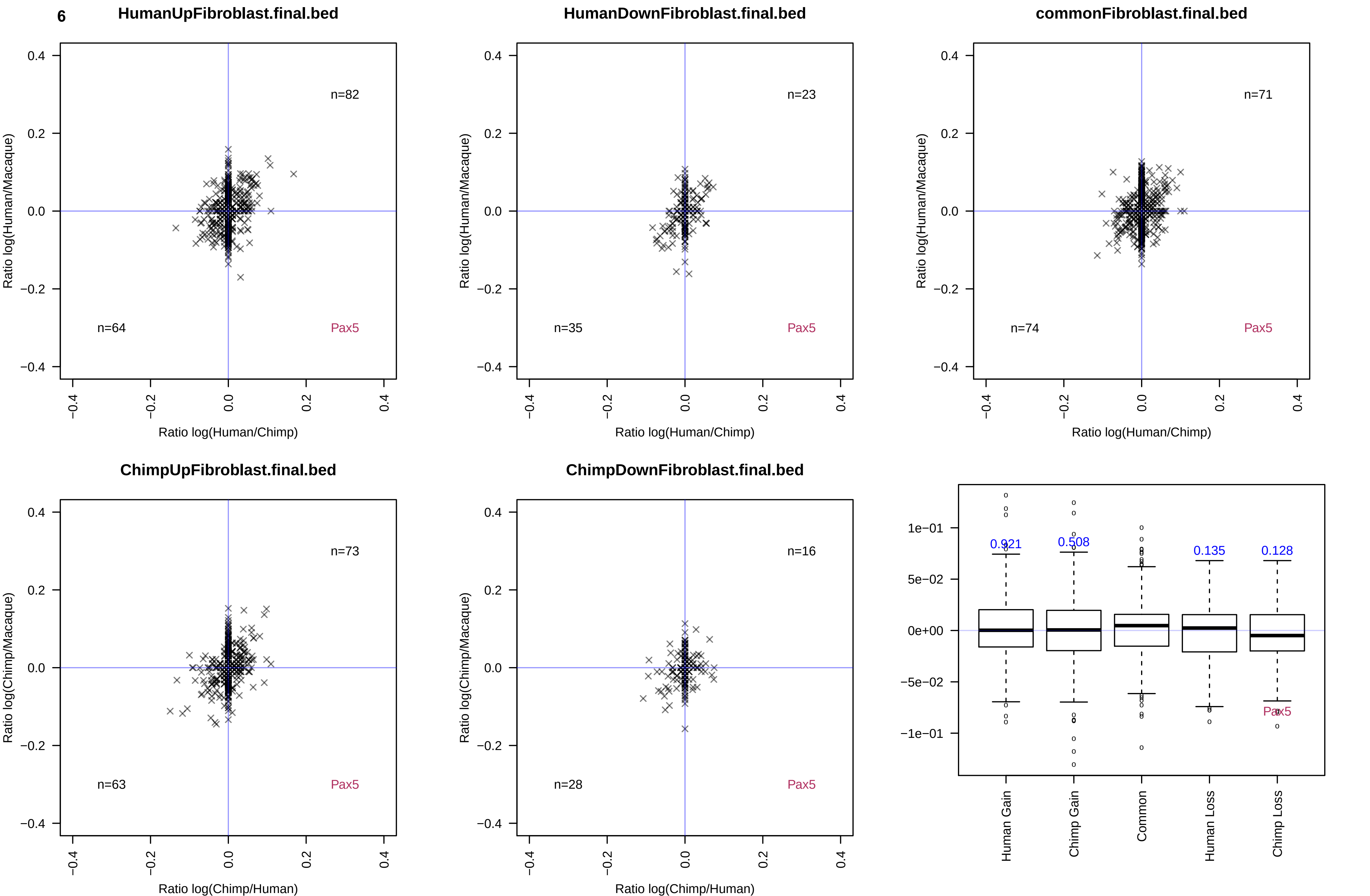
6
HumanUpFibroblast.final.bed
HumanDownFibroblast.final.bed
commonFibroblast.final.bed
ChimpUpFibroblast.final.bed
ChimpDownFibroblast.final.bed
Ratio log(Human/Macaque)
Ratio log(Human/Chimp)
Ratio log(Chimp/Macaque)
Ratio log(Chimp/Human)
n=82
n=64
n=23
n=35
n=71
n=74
n=73
n=63
n=16
n=28
Pax5
0.921
0.508
0.135
0.128
Human Gain
Chimp Gain
Common
Human Loss
Chimp Loss

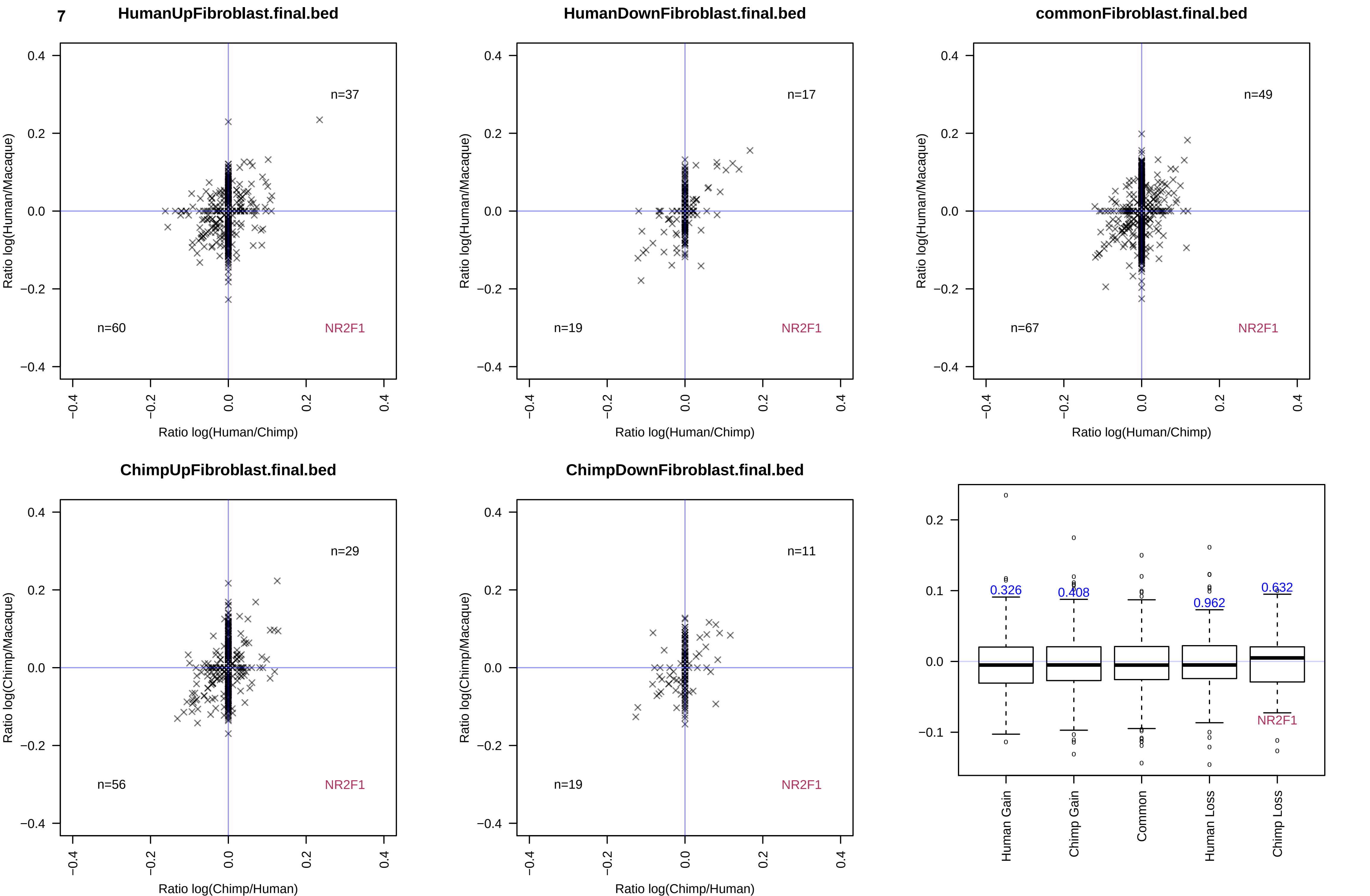
HumanUpFibroblast.final.bed
n=37
n=60
NR2F1
Ratio log(Human/Macaque)
Ratio log(Human/Chimp)
HumanDownFibroblast.final.bed
n=17
n=19
NR2F1
Ratio log(Human/Macaque)
Ratio log(Human/Chimp)
commonFibroblast.final.bed
n=49
n=67
NR2F1
Ratio log(Human/Macaque)
Ratio log(Human/Chimp)
ChimpUpFibroblast.final.bed
n=29
n=56
NR2F1
Ratio log(Chimp/Macaque)
Ratio log(Chimp/Human)
ChimpDownFibroblast.final.bed
n=11
n=19
NR2F1
Ratio log(Chimp/Macaque)
Ratio log(Chimp/Human)
0.326
0.408
0.962
0.632
NR2F1
Human Gain
Chimp Gain
Common
Human Loss
Chimp Loss

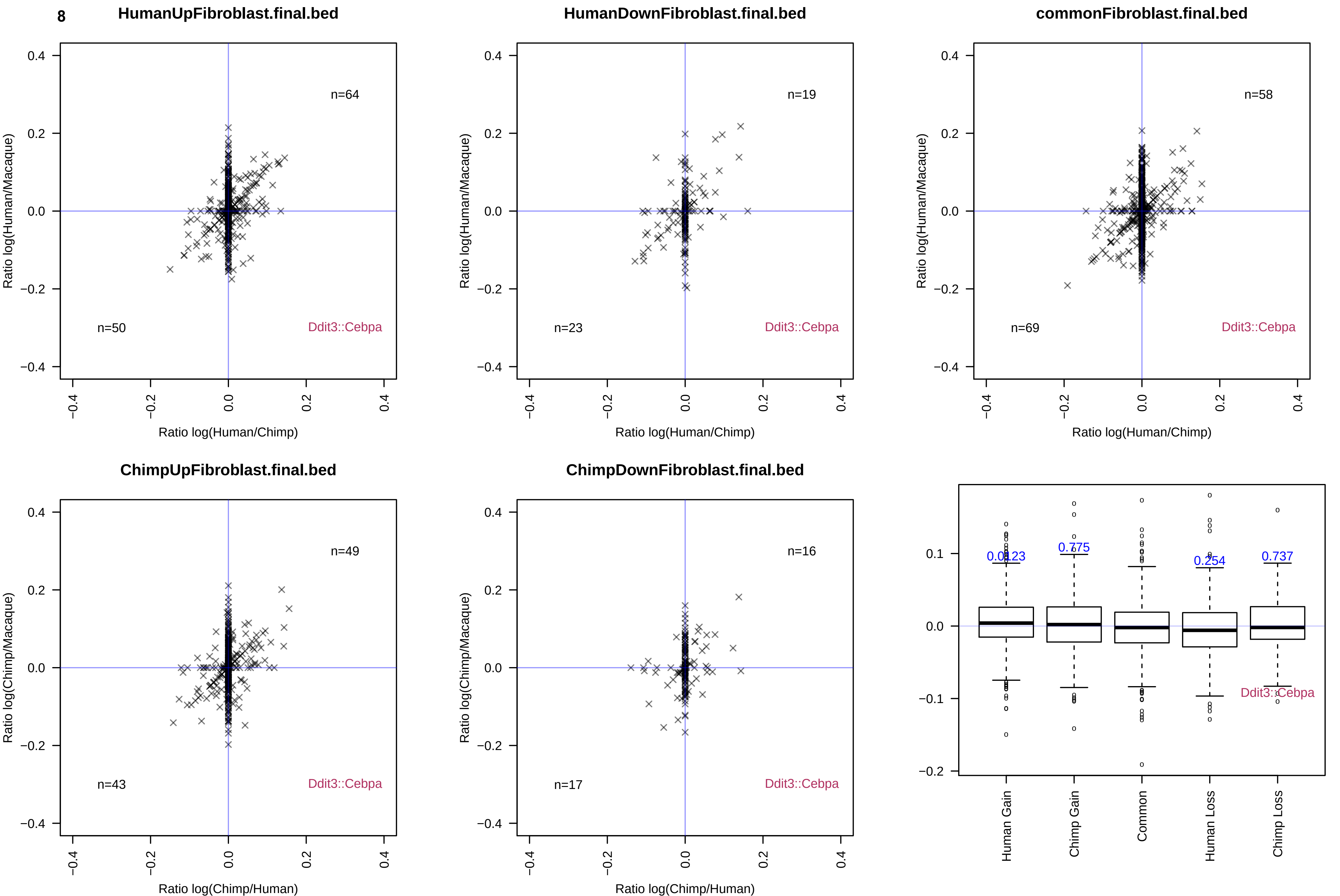

HumanUpFibroblast.final.bed
n=64
n=50
Ddit3::Cebpa
Ratio log(Human/Macaque)
Ratio log(Human/Chimp)
HumanDownFibroblast.final.bed
n=19
n=23
Ddit3::Cebpa
Ratio log(Human/Macaque)
Ratio log(Human/Chimp)
commonFibroblast.final.bed
n=58
n=69
Ddit3::Cebpa
Ratio log(Human/Macaque)
Ratio log(Human/Chimp)
ChimpUpFibroblast.final.bed
n=49
n=43
Ddit3::Cebpa
Ratio log(Chimp/Macaque)
Ratio log(Chimp/Human)
ChimpDownFibroblast.final.bed
n=16
n=17
Ddit3::Cebpa
Ratio log(Chimp/Macaque)
Ratio log(Chimp/Human)
0.0123
0.775
0.254
0.737
Ddit3::Cebpa
Human Gain
Chimp Gain
Common
Human Loss
Chimp Loss

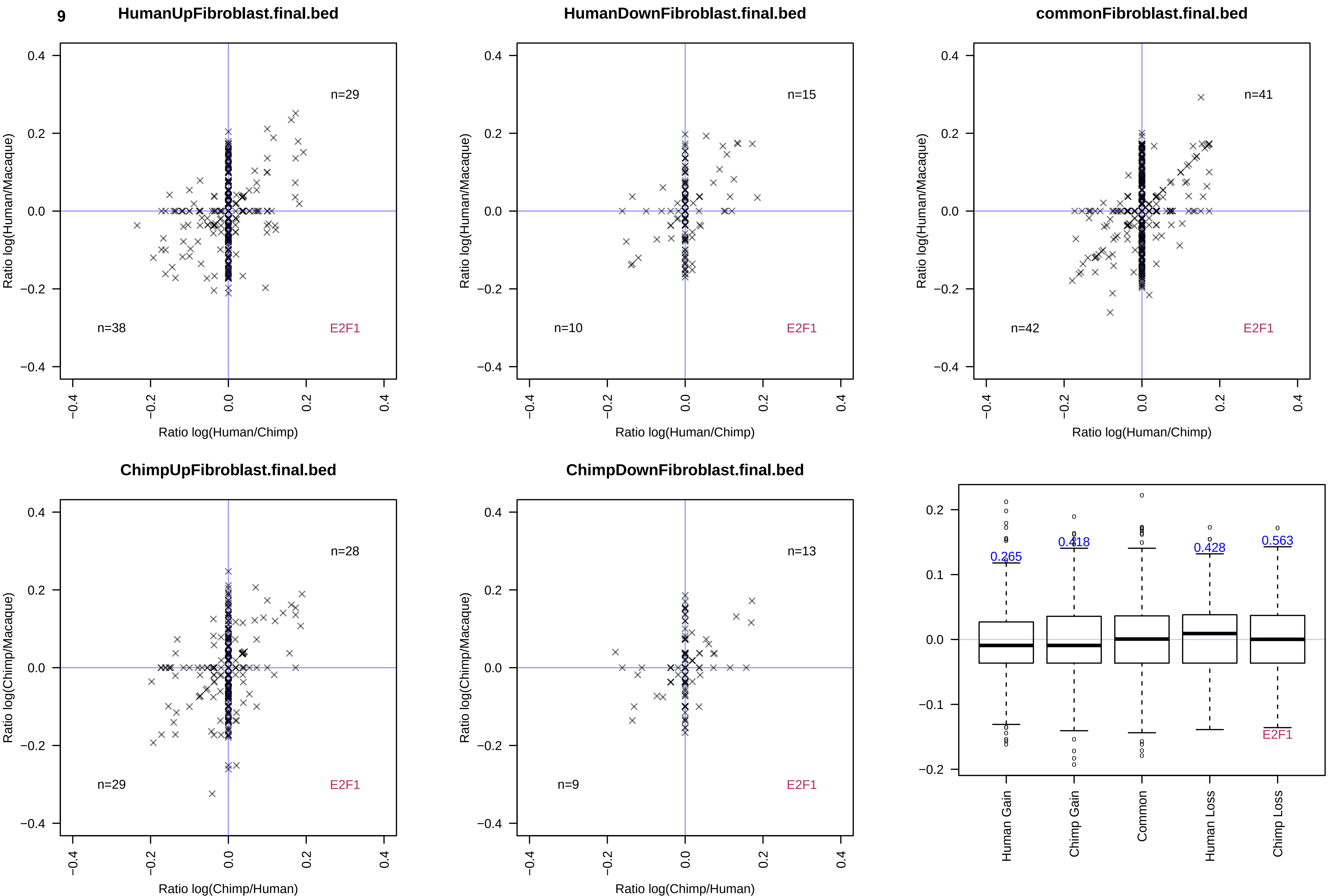
HumanUpFibroblast.final.bed
n=29
n=38
E2F1
Ratio log(Human/Chimp)
Ratio log(Human/Macaque)
HumanDownFibroblast.final.bed
n=15
n=10
E2F1
Ratio log(Human/Chimp)
Ratio log(Human/Macaque)
commonFibroblast.final.bed
n=41
n=42
E2F1
Ratio log(Human/Chimp)
Ratio log(Human/Macaque)
ChimpUpFibroblast.final.bed
n=28
n=29
E2F1
Ratio log(Chimp/Human)
Ratio log(Chimp/Macaque)
ChimpDownFibroblast.final.bed
n=13
n=9
E2F1
Ratio log(Chimp/Human)
Ratio log(Chimp/Macaque)
0.265
0.418
0.428
0.563
E2F1
Human Gain
Chimp Gain
Common
Human Loss
Chimp Loss

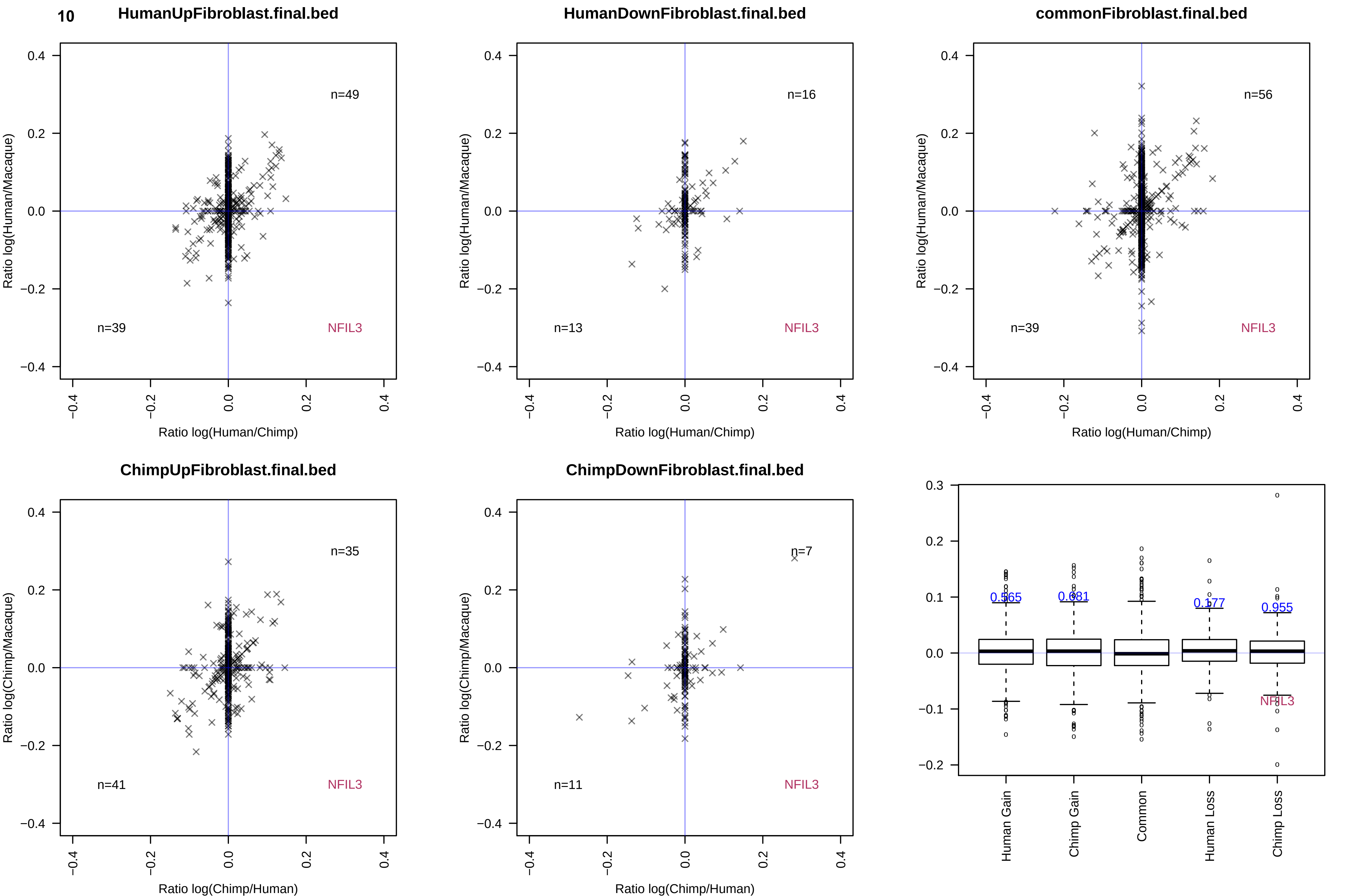
HumanUpFibroblast.final.bed
n=49
n=39
NFIL3
Ratio log(Human/Chimp)
Ratio log(Human/Macaque)
HumanDownFibroblast.final.bed
n=16
n=13
NFIL3
Ratio log(Human/Chimp)
Ratio log(Human/Macaque)
commonFibroblast.final.bed
n=56
n=39
NFIL3
Ratio log(Human/Chimp)
Ratio log(Human/Macaque)
ChimpUpFibroblast.final.bed
n=35
n=41
NFIL3
Ratio log(Chimp/Human)
Ratio log(Chimp/Macaque)
ChimpDownFibroblast.final.bed
n=7
n=11
NFIL3
Ratio log(Chimp/Human)
Ratio log(Chimp/Macaque)
0.565
0.681
0.177
0.955
NFIL3
Human Gain
Chimp Gain
Common
Human Loss
Chimp Loss

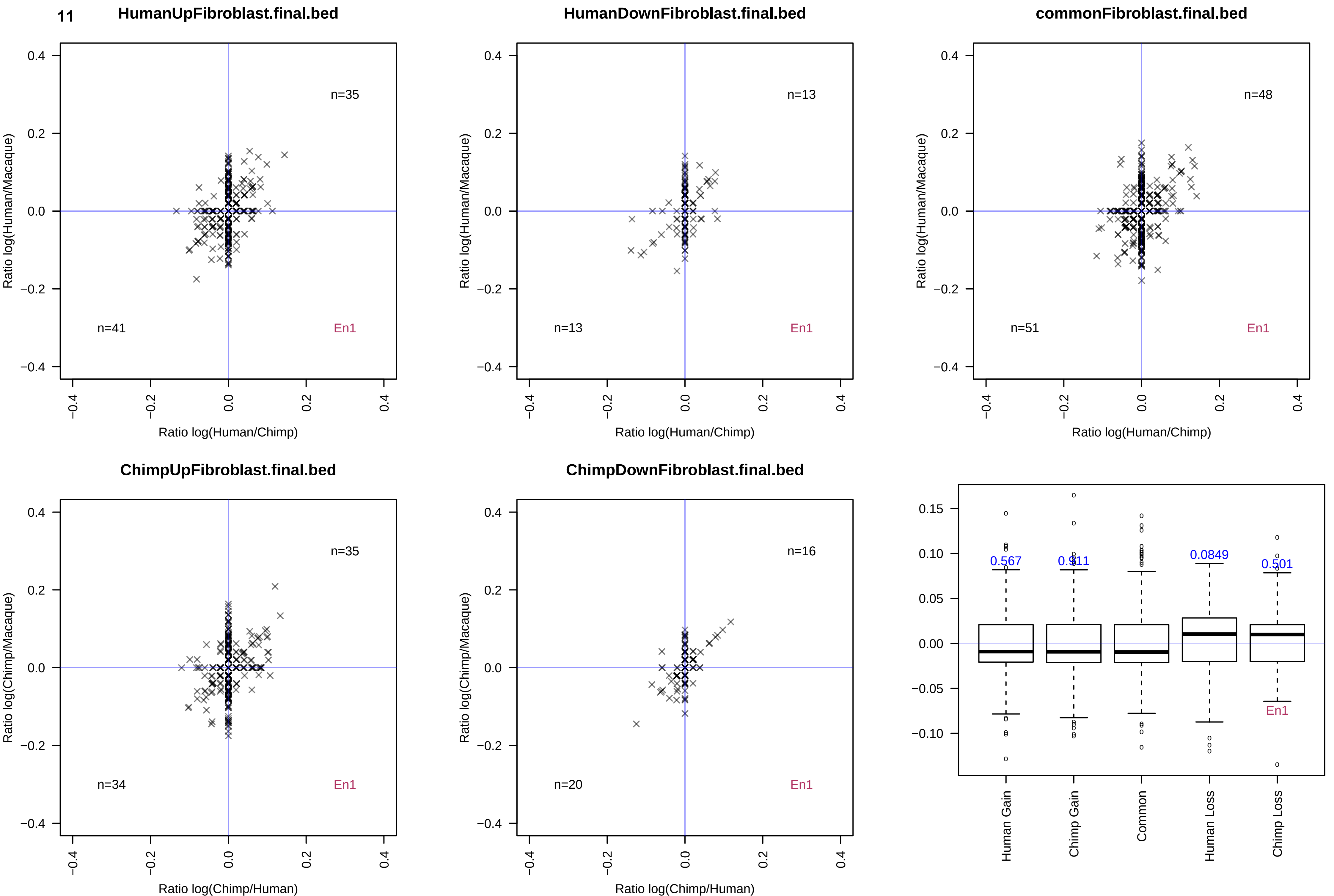
HumanUpFibroblast.final.bed
HumanDownFibroblast.final.bed
commonFibroblast.final.bed
ChimpUpFibroblast.final.bed
ChimpDownFibroblast.final.bed
Ratio log(Human/Macaque)
Ratio log(Human/Chimp)
Ratio log(Chimp/Macaque)
Ratio log(Chimp/Human)
n=35
n=41
n=13
n=13
n=48
n=51
n=35
n=34
n=16
n=20
En1
0.567
0.911
0.0849
0.501
Human Gain
Chimp Gain
Common
Human Loss
Chimp Loss

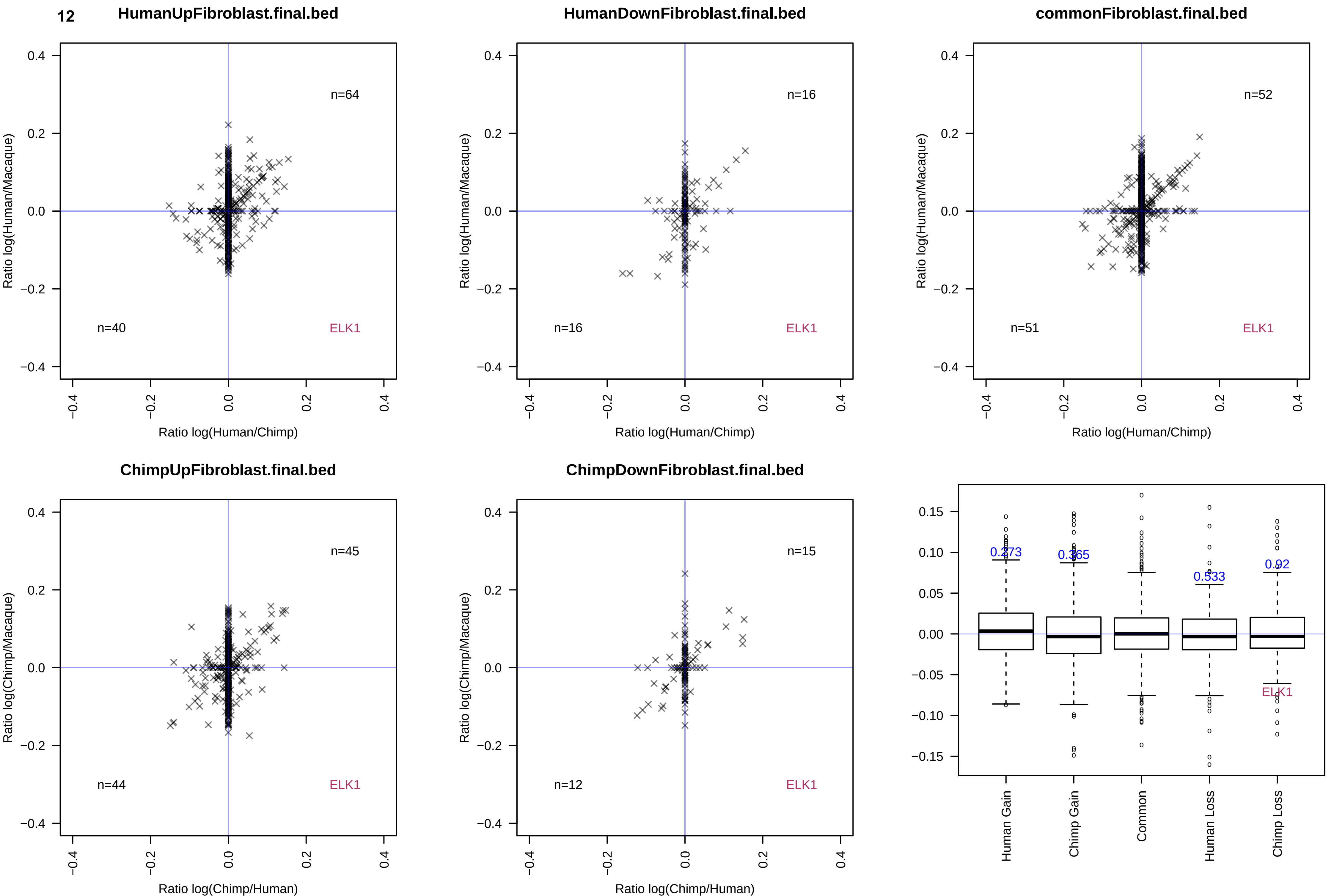
HumanUpFibroblast.final.bed
HumanDownFibroblast.final.bed
commonFibroblast.final.bed
ChimpUpFibroblast.final.bed
ChimpDownFibroblast.final.bed
n=64
n=40
n=16
n=16
n=52
n=51
n=45
n=44
n=15
n=12
ELK1
Ratio log(Human/Macaque)
Ratio log(Human/Chimp)
Ratio log(Chimp/Macaque)
Ratio log(Chimp/Human)
0.273
0.365
0.533
0.92
Human Gain
Chimp Gain
Common
Human Loss
Chimp Loss

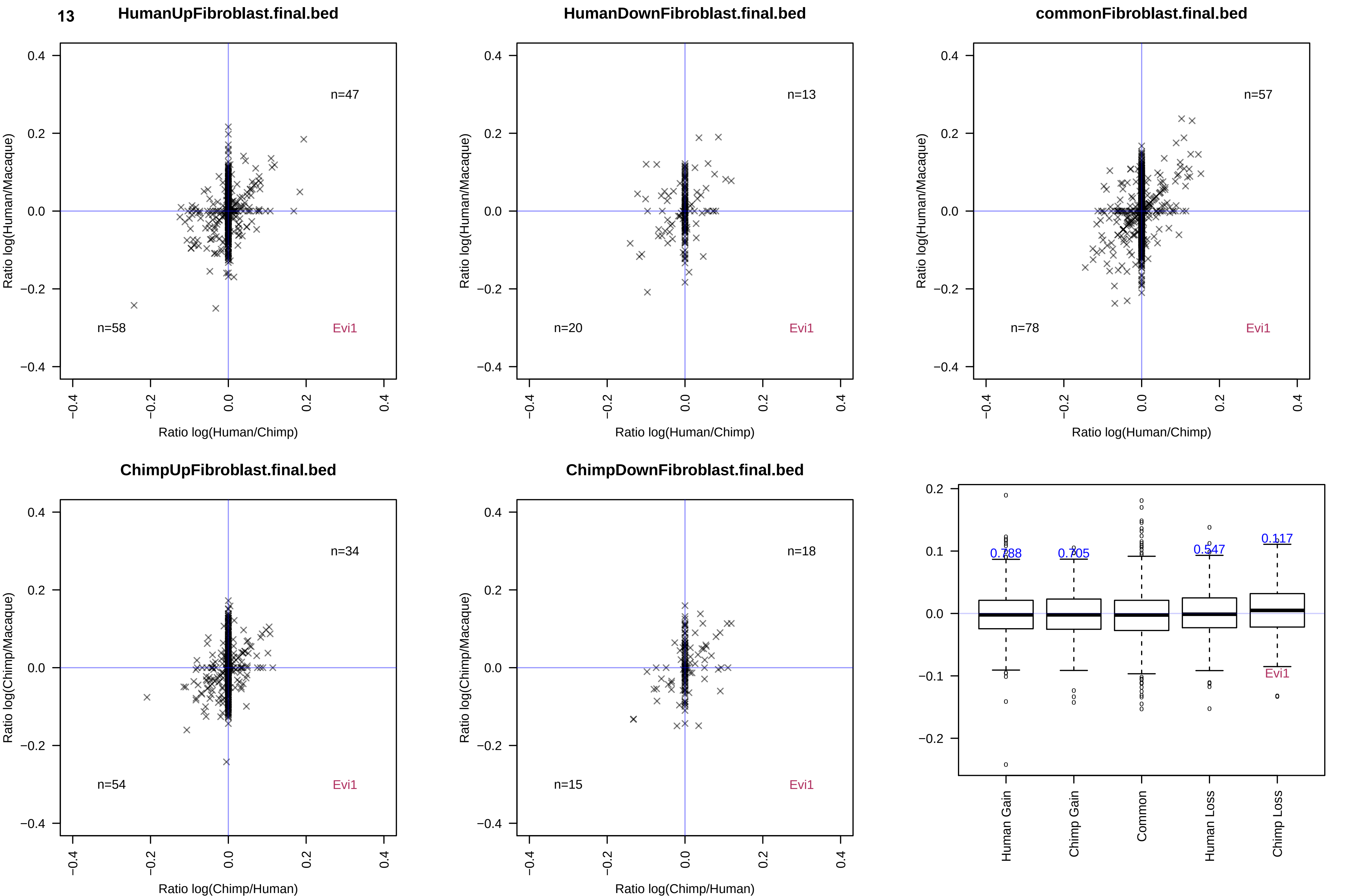
HumanUpFibroblast.final.bed
n=47
n=58
Evi1
Ratio log(Human/Macaque)
Ratio log(Human/Chimp)
HumanDownFibroblast.final.bed
n=13
n=20
Evi1
Ratio log(Human/Macaque)
Ratio log(Human/Chimp)
commonFibroblast.final.bed
n=57
n=78
Evi1
Ratio log(Human/Macaque)
Ratio log(Human/Chimp)
ChimpUpFibroblast.final.bed
n=34
n=54
Evi1
Ratio log(Chimp/Macaque)
Ratio log(Chimp/Human)
ChimpDownFibroblast.final.bed
n=18
n=15
Evi1
Ratio log(Chimp/Macaque)
Ratio log(Chimp/Human)
0.788
0.705
0.547
0.117
Evi1
Human Gain
Chimp Gain
Common
Human Loss
Chimp Loss

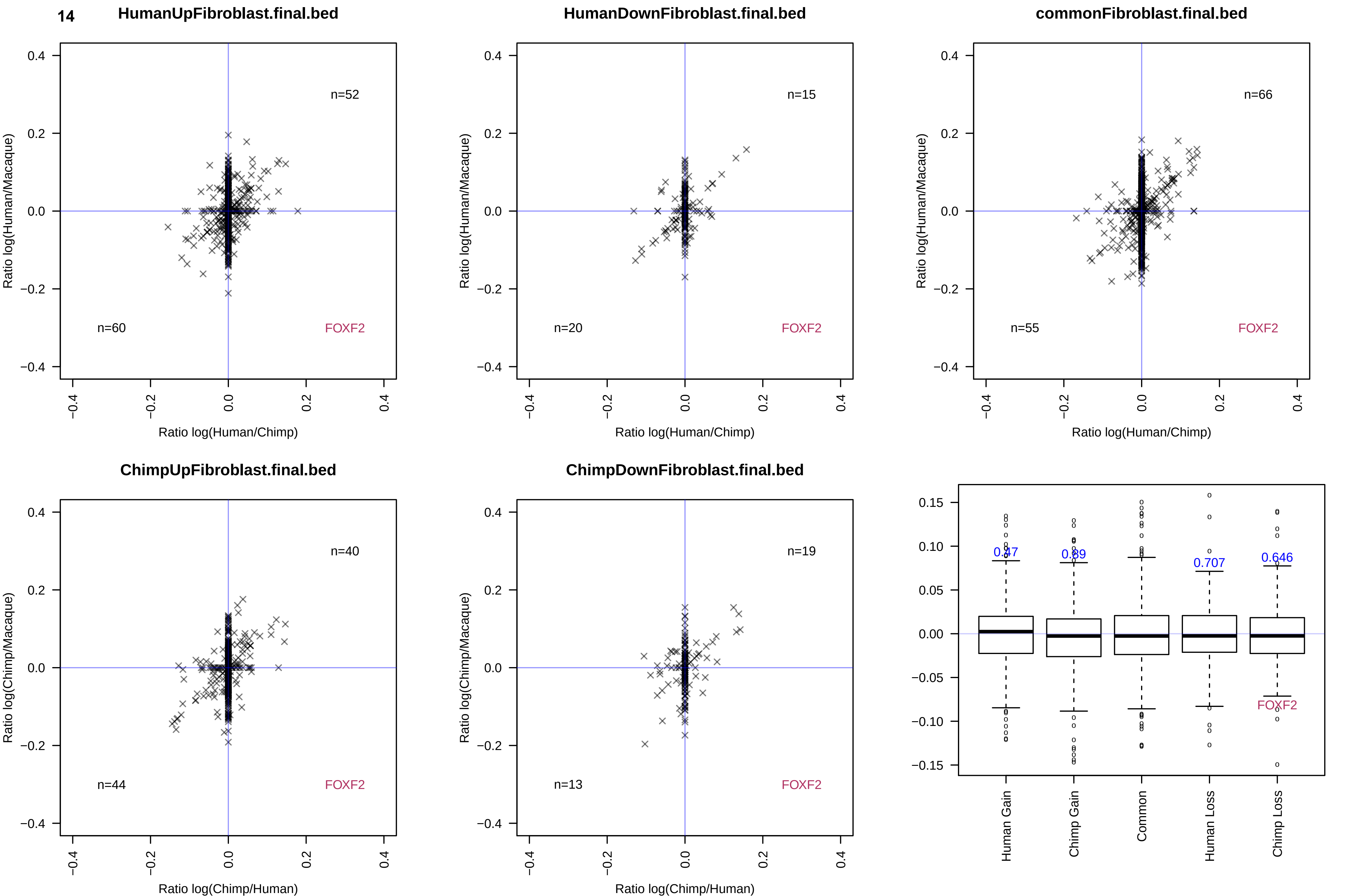
HumanUpFibroblast.final.bed
n=52
n=60
FOXF2
Ratio log(Human/Chimp)
Ratio log(Human/Macaque)
HumanDownFibroblast.final.bed
n=15
n=20
FOXF2
Ratio log(Human/Chimp)
Ratio log(Human/Macaque)
commonFibroblast.final.bed
n=66
n=55
FOXF2
Ratio log(Human/Chimp)
Ratio log(Human/Macaque)
ChimpUpFibroblast.final.bed
n=40
n=44
FOXF2
Ratio log(Chimp/Human)
Ratio log(Chimp/Macaque)
ChimpDownFibroblast.final.bed
n=19
n=13
FOXF2
Ratio log(Chimp/Human)
Ratio log(Chimp/Macaque)
0.47
0.89
0.707
0.646
FOXF2
Human Gain
Chimp Gain
Common
Human Loss
Chimp Loss

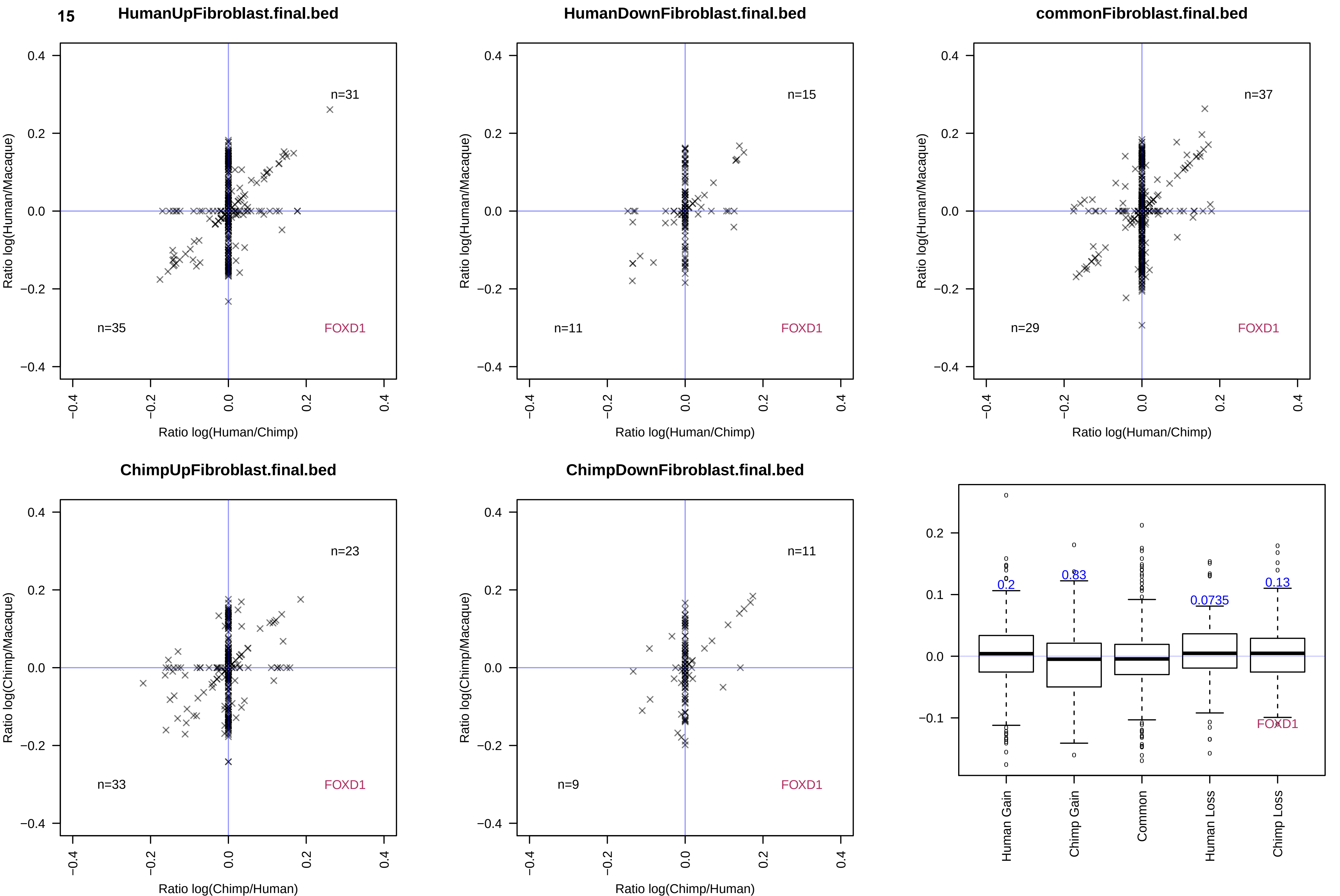
HumanUpFibroblast.final.bed
n=31
n=35
FOXD1
Ratio log(Human/Chimp)
Ratio log(Human/Macaque)
HumanDownFibroblast.final.bed
n=15
n=11
FOXD1
commonFibroblast.final.bed
n=37
n=29
FOXD1
ChimpUpFibroblast.final.bed
n=23
n=33
FOXD1
Ratio log(Chimp/Human)
Ratio log(Chimp/Macaque)
ChimpDownFibroblast.final.bed
n=11
n=9
FOXD1
0.2
0.83
0.0735
0.13
FOXD1
Human Gain
Chimp Gain
Common
Human Loss
Chimp Loss

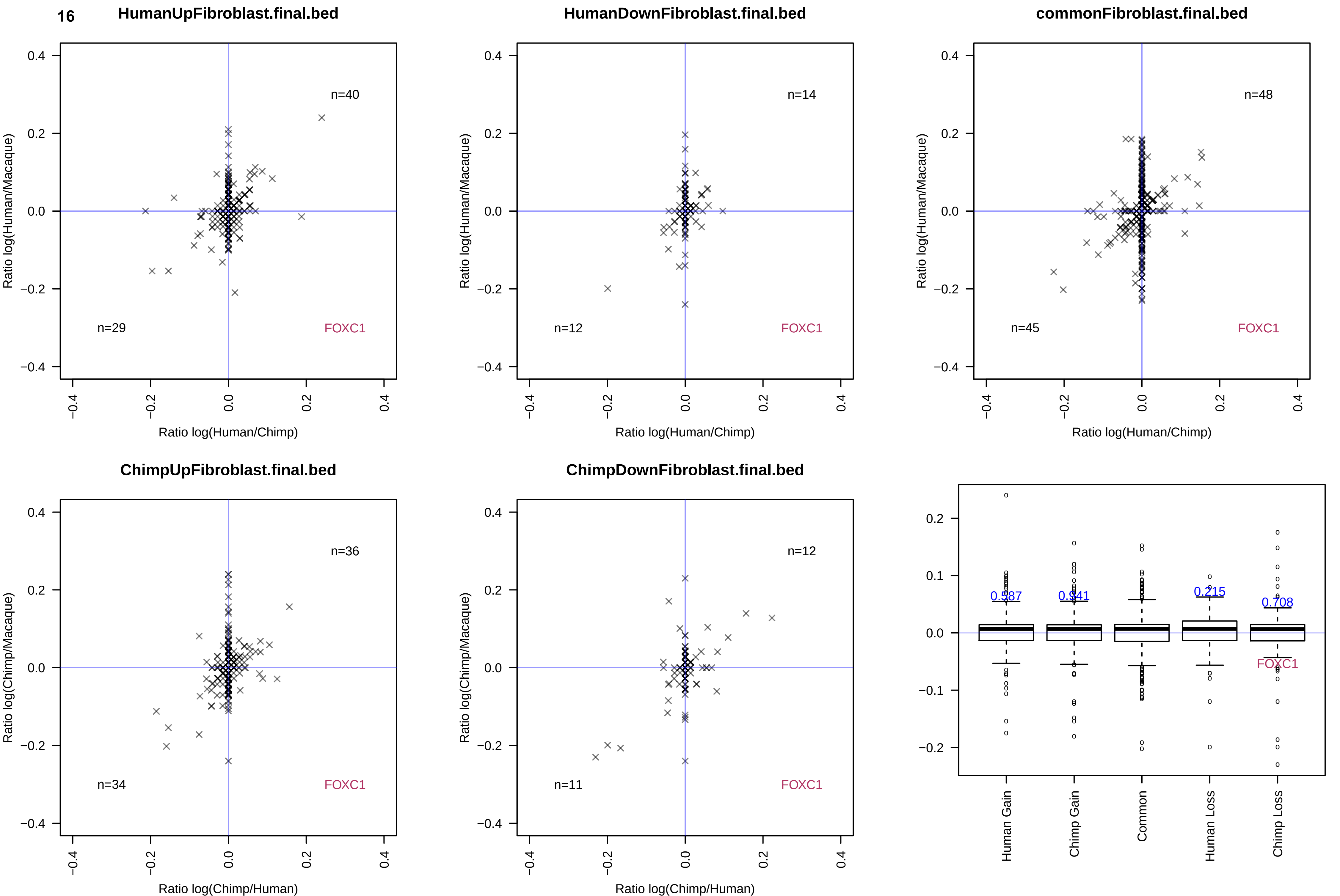
HumanUpFibroblast.final.bed
n=40
n=29
FOXC1
Ratio log(Human/Macaque)
Ratio log(Human/Chimp)
HumanDownFibroblast.final.bed
n=14
n=12
FOXC1
Ratio log(Human/Macaque)
Ratio log(Human/Chimp)
commonFibroblast.final.bed
n=48
n=45
FOXC1
Ratio log(Human/Macaque)
Ratio log(Human/Chimp)
ChimpUpFibroblast.final.bed
n=36
n=34
FOXC1
Ratio log(Chimp/Macaque)
Ratio log(Chimp/Human)
ChimpDownFibroblast.final.bed
n=12
n=11
FOXC1
Ratio log(Chimp/Macaque)
Ratio log(Chimp/Human)
0.587
0.941
0.215
0.708
FOXC1
Human Gain
Chimp Gain
Common
Human Loss
Chimp Loss

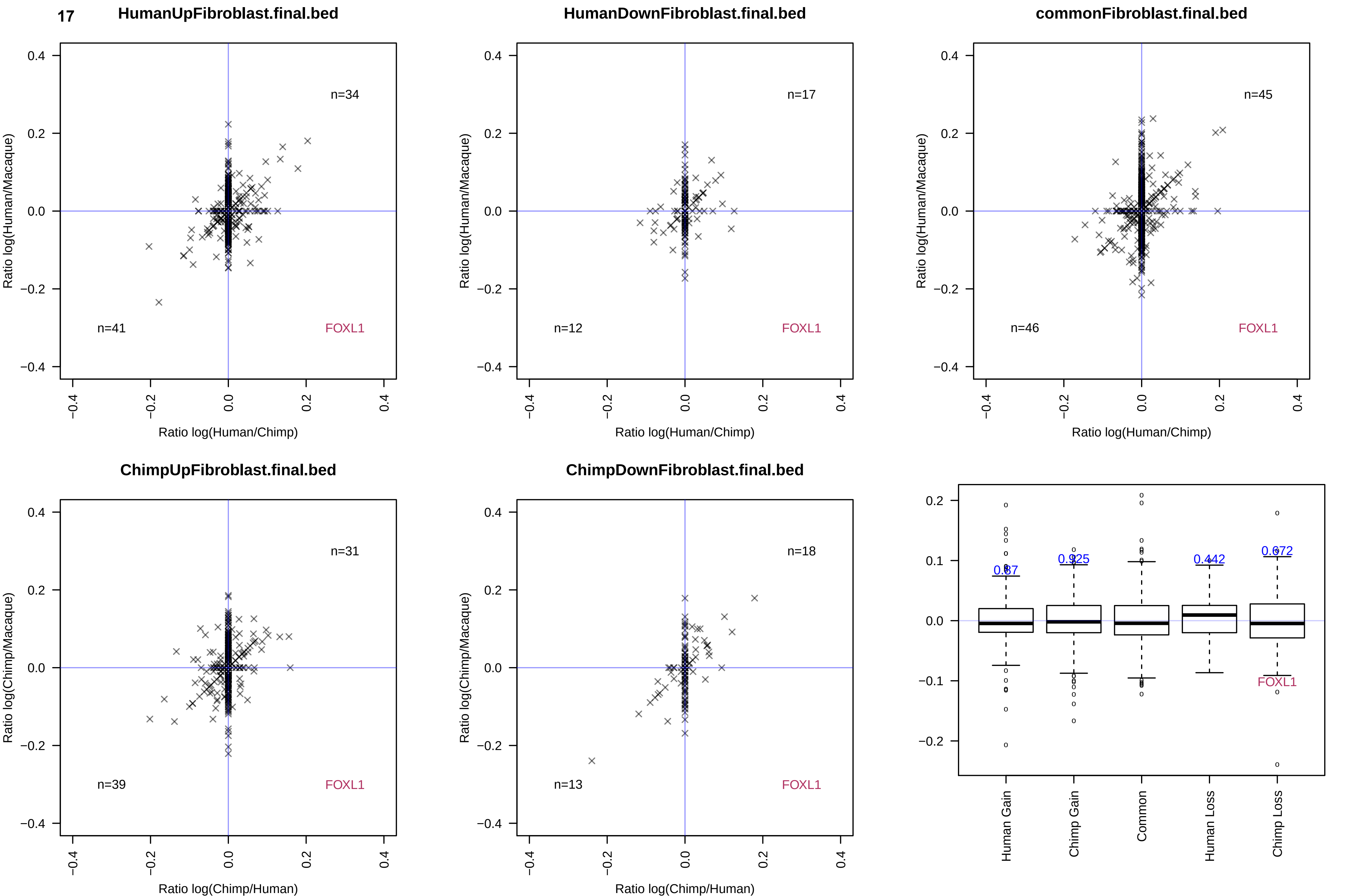
HumanUpFibroblast.final.bed
HumanDownFibroblast.final.bed
commonFibroblast.final.bed
ChimpUpFibroblast.final.bed
ChimpDownFibroblast.final.bed
n=34
n=41
n=17
n=12
n=45
n=46
n=31
n=39
n=18
n=13
FOXL1
Ratio log(Human/Macaque)
Ratio log(Human/Chimp)
Ratio log(Chimp/Macaque)
Ratio log(Chimp/Human)
0.87
0.925
0.442
0.672
Human Gain
Chimp Gain
Common
Human Loss
Chimp Loss

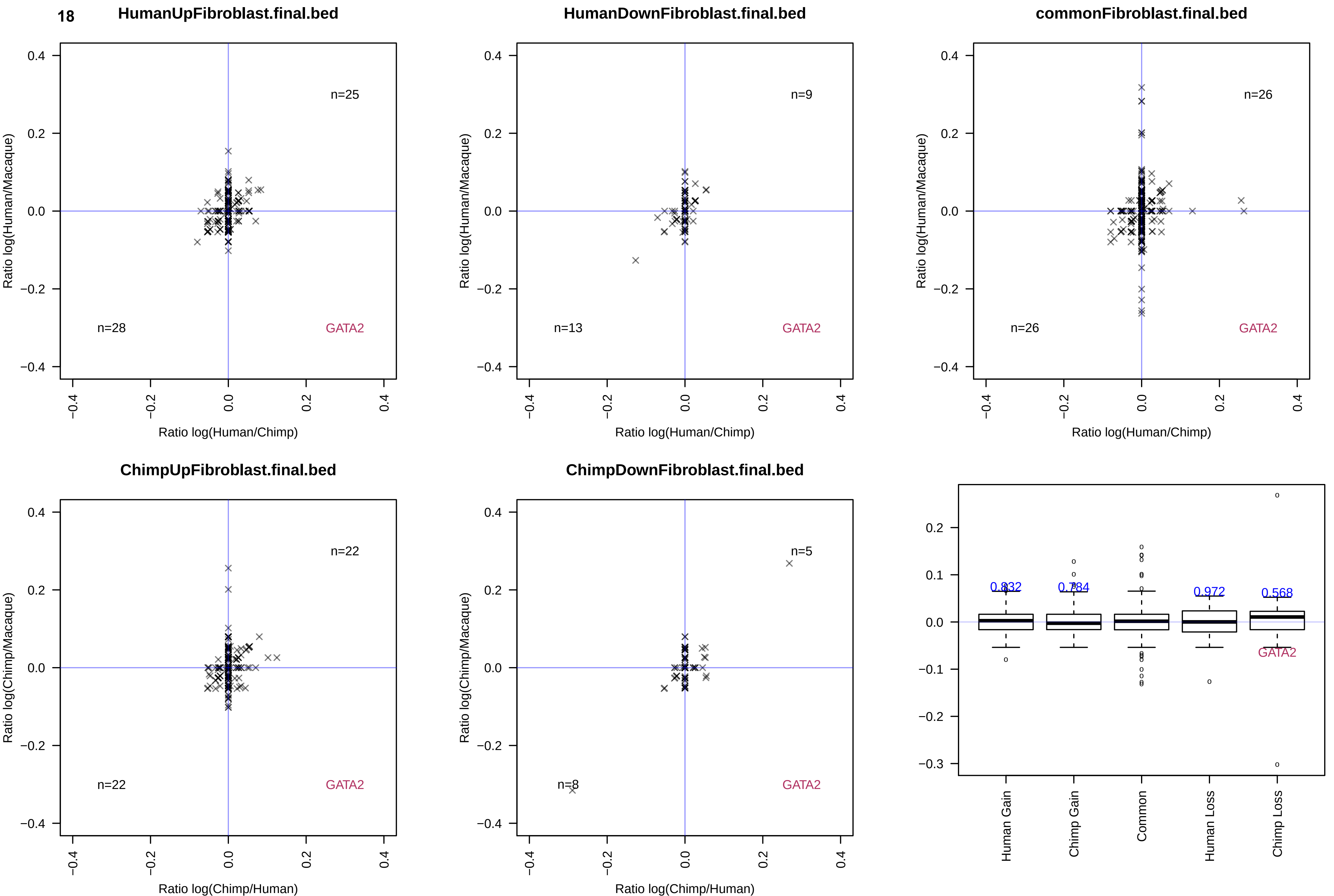
HumanUpFibroblast.final.bed
n=25
n=28
GATA2
Ratio log(Human/Macaque)
Ratio log(Human/Chimp)
HumanDownFibroblast.final.bed
n=9
n=13
GATA2
Ratio log(Human/Macaque)
Ratio log(Human/Chimp)
commonFibroblast.final.bed
n=26
n=26
GATA2
Ratio log(Human/Macaque)
Ratio log(Human/Chimp)
ChimpUpFibroblast.final.bed
n=22
n=22
GATA2
Ratio log(Chimp/Macaque)
Ratio log(Chimp/Human)
ChimpDownFibroblast.final.bed
n=5
n=8
GATA2
Ratio log(Chimp/Macaque)
Ratio log(Chimp/Human)
0.832
0.784
0.972
0.568
GATA2
Human Gain
Chimp Gain
Common
Human Loss
Chimp Loss

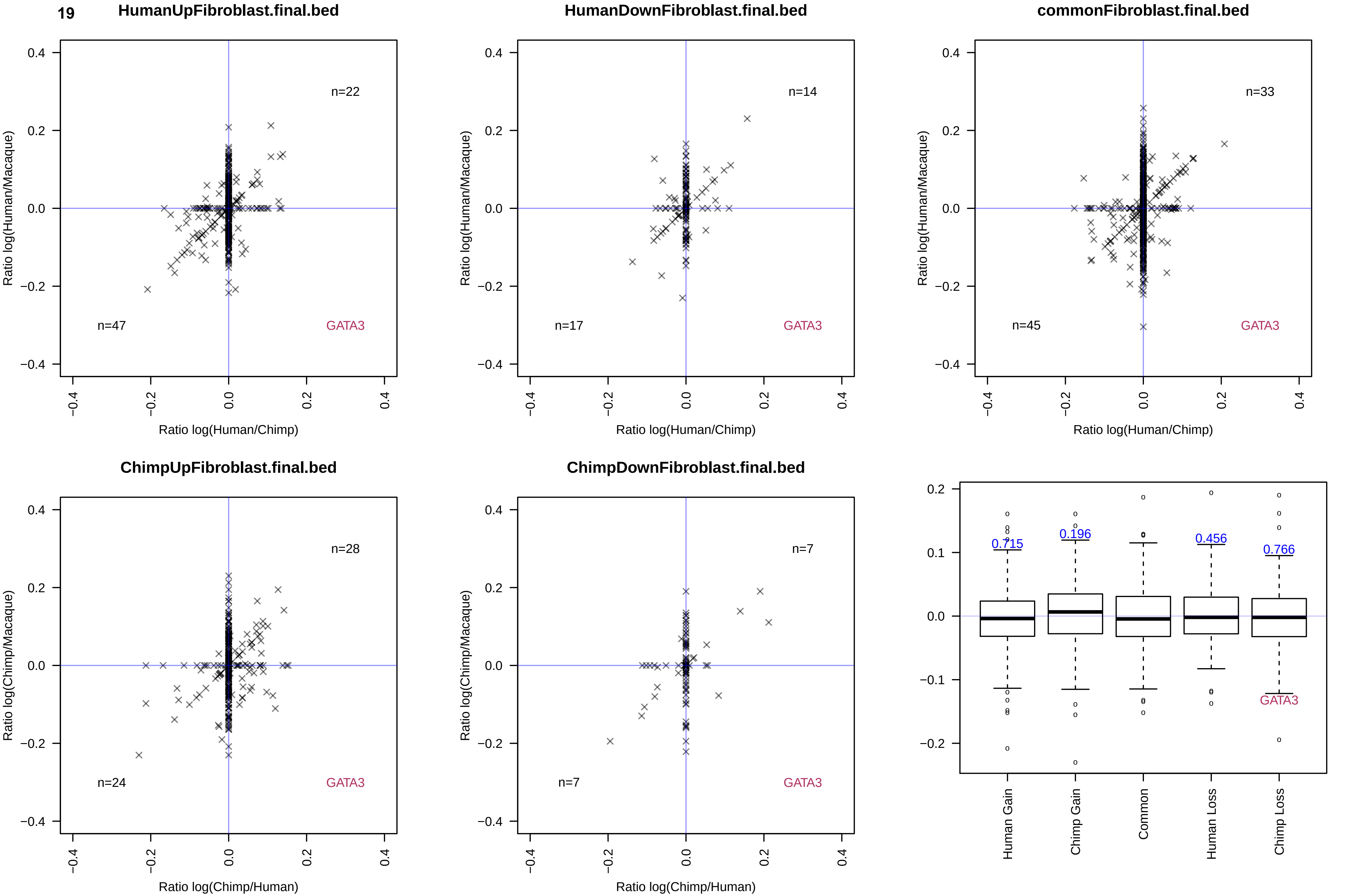
HumanUpFibroblast.final.bed
n=22
n=47
GATA3
Ratio log(Human/Macaque)
Ratio log(Human/Chimp)
HumanDownFibroblast.final.bed
n=14
n=17
GATA3
Ratio log(Human/Macaque)
Ratio log(Human/Chimp)
commonFibroblast.final.bed
n=33
n=45
GATA3
Ratio log(Human/Macaque)
Ratio log(Human/Chimp)
ChimpUpFibroblast.final.bed
n=28
n=24
GATA3
Ratio log(Chimp/Macaque)
Ratio log(Chimp/Human)
ChimpDownFibroblast.final.bed
n=7
n=7
GATA3
Ratio log(Chimp/Macaque)
Ratio log(Chimp/Human)
0.715
0.196
0.456
0.766
GATA3
Human Gain
Chimp Gain
Common
Human Loss
Chimp Loss

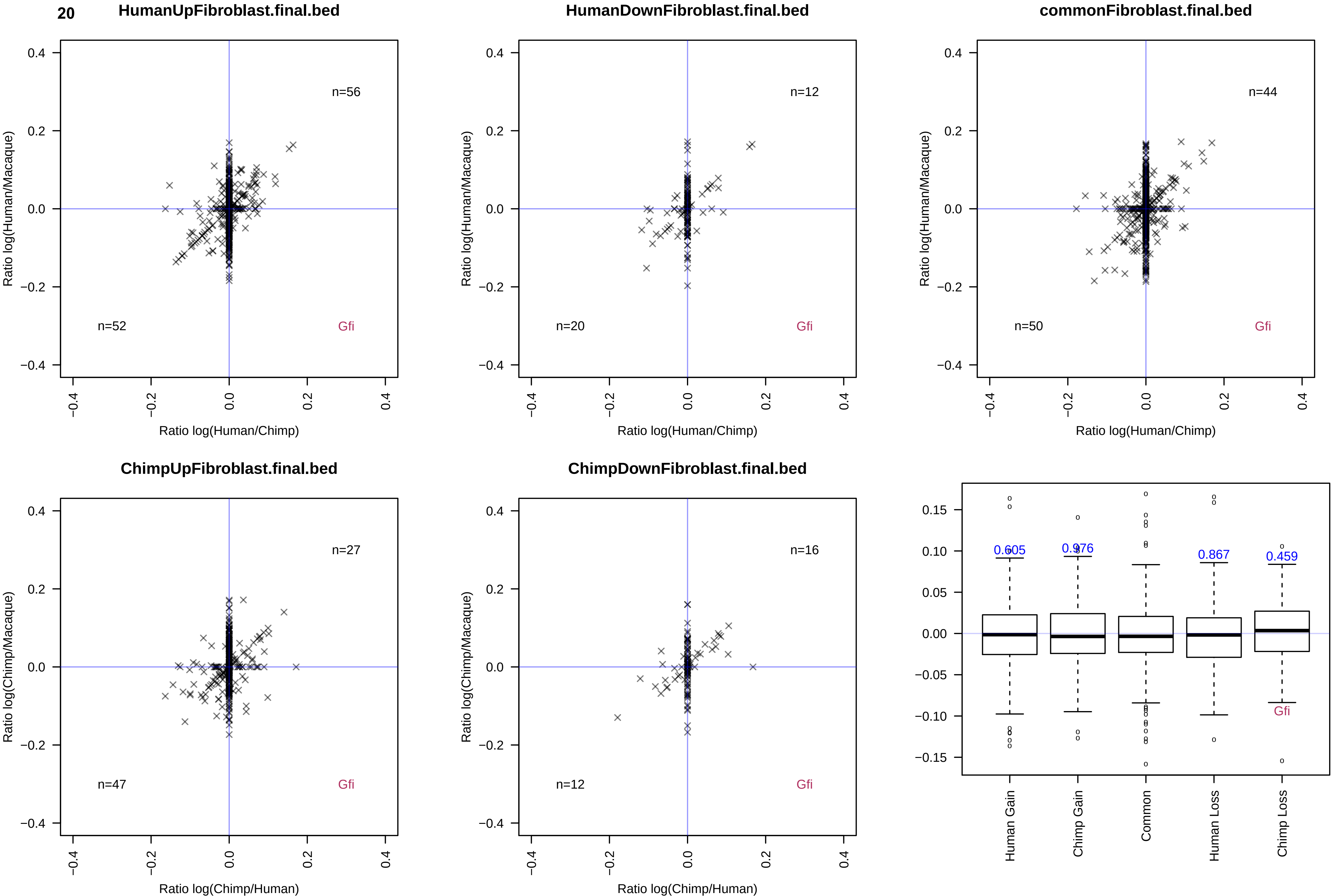
HumanUpFibroblast.final.bed
n=56
n=52
Gfi
Ratio log(Human/Chimp)
Ratio log(Human/Macaque)
HumanDownFibroblast.final.bed
n=12
n=20
Gfi
Ratio log(Human/Chimp)
Ratio log(Human/Macaque)
commonFibroblast.final.bed
n=44
n=50
Gfi
Ratio log(Human/Chimp)
Ratio log(Human/Macaque)
ChimpUpFibroblast.final.bed
n=27
n=47
Gfi
Ratio log(Chimp/Human)
Ratio log(Chimp/Macaque)
ChimpDownFibroblast.final.bed
n=16
n=12
Gfi
Ratio log(Chimp/Human)
Ratio log(Chimp/Macaque)
0.605
0.976
0.867
0.459
Gfi
Human Gain
Chimp Gain
Common
Human Loss
Chimp Loss

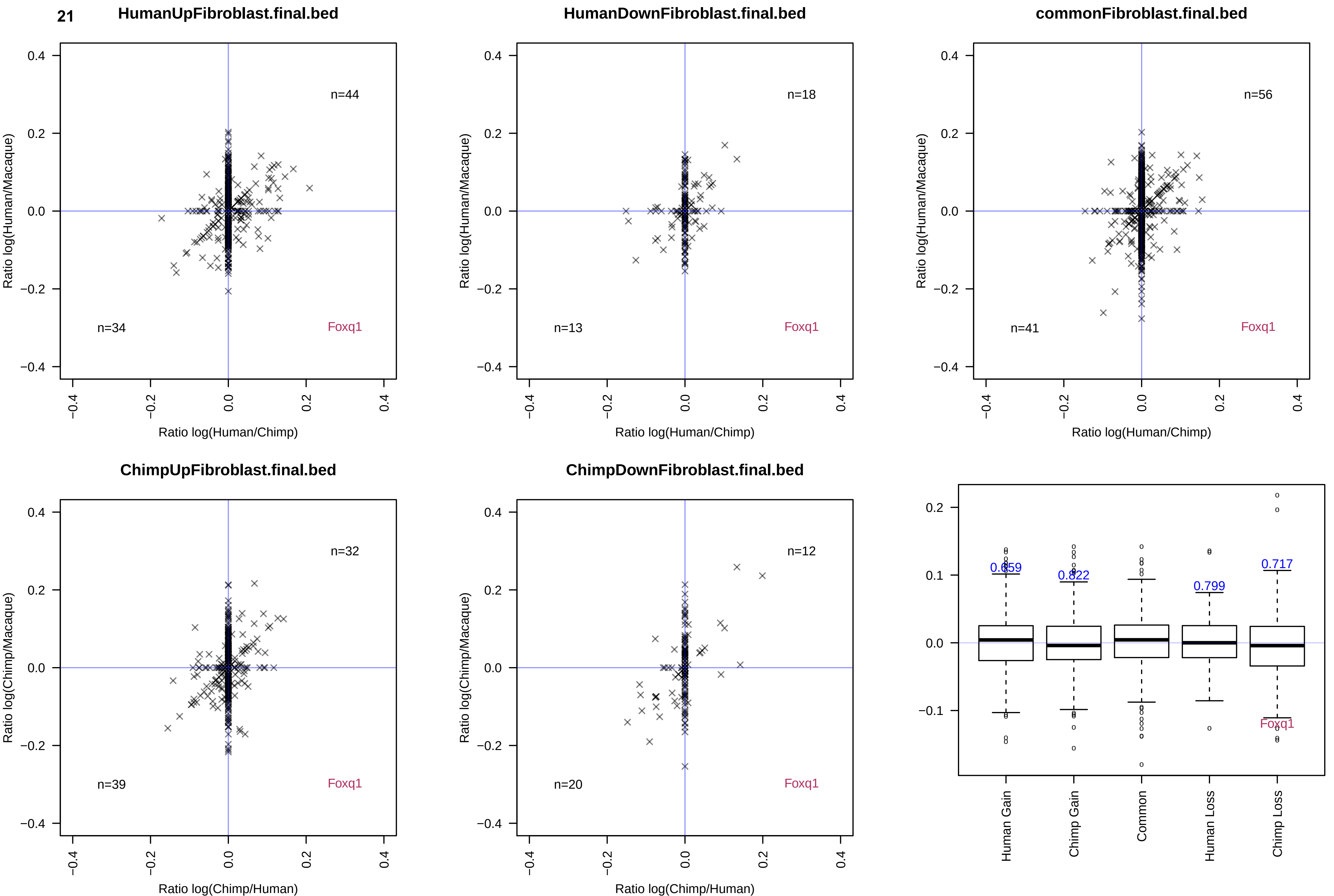
HumanUpFibroblast.final.bed
n=44
n=34
Foxq1
Ratio log(Human/Macaque)
Ratio log(Human/Chimp)
HumanDownFibroblast.final.bed
n=18
n=13
Foxq1
Ratio log(Human/Macaque)
Ratio log(Human/Chimp)
commonFibroblast.final.bed
n=56
n=41
Foxq1
Ratio log(Human/Macaque)
Ratio log(Human/Chimp)
ChimpUpFibroblast.final.bed
n=32
n=39
Foxq1
Ratio log(Chimp/Macaque)
Ratio log(Chimp/Human)
ChimpDownFibroblast.final.bed
n=12
n=20
Foxq1
Ratio log(Chimp/Macaque)
Ratio log(Chimp/Human)
0.659
0.822
0.799
0.717
Foxq1
Human Gain
Chimp Gain
Common
Human Loss
Chimp Loss

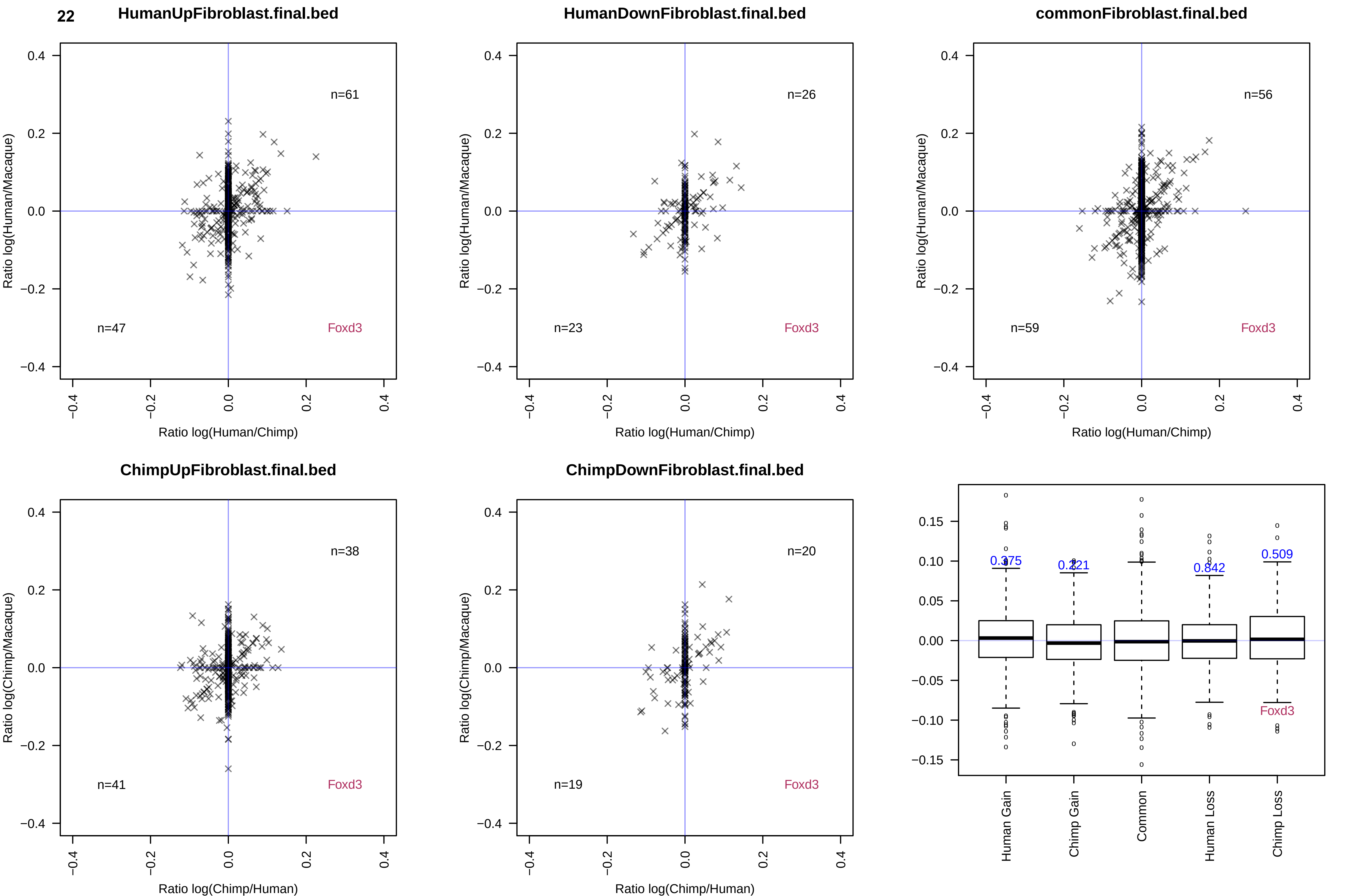
HumanUpFibroblast.final.bed
n=61
n=47
Foxd3
HumanDownFibroblast.final.bed
n=26
n=23
Foxd3
commonFibroblast.final.bed
n=56
n=59
Foxd3
Ratio log(Human/Macaque)
Ratio log(Human/Chimp)
ChimpUpFibroblast.final.bed
n=38
n=41
Foxd3
ChimpDownFibroblast.final.bed
n=20
n=19
Foxd3
Ratio log(Chimp/Macaque)
Ratio log(Chimp/Human)
0.375
0.221
0.842
0.509
Foxd3
Human Gain
Chimp Gain
Common
Human Loss
Chimp Loss

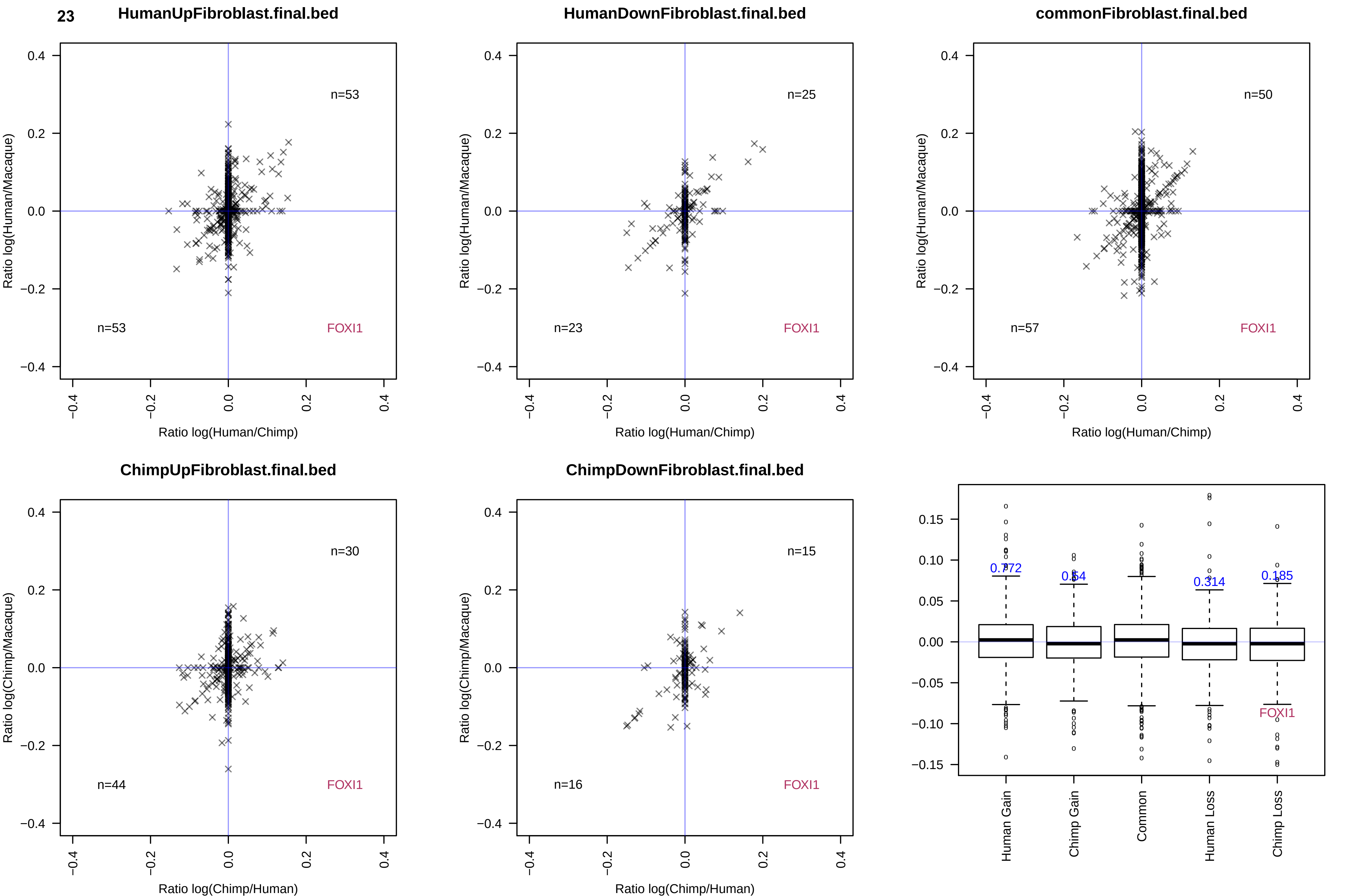
HumanUpFibroblast.final.bed
n=53
n=53
FOXI1
HumanDownFibroblast.final.bed
n=25
n=23
FOXI1
commonFibroblast.final.bed
n=50
n=57
FOXI1
Ratio log(Human/Macaque)
Ratio log(Human/Chimp)
ChimpUpFibroblast.final.bed
n=30
n=44
FOXI1
ChimpDownFibroblast.final.bed
n=15
n=16
FOXI1
Ratio log(Chimp/Macaque)
Ratio log(Chimp/Human)
0.772
0.54
0.314
0.185
FOXI1
Human Gain
Chimp Gain
Common
Human Loss
Chimp Loss

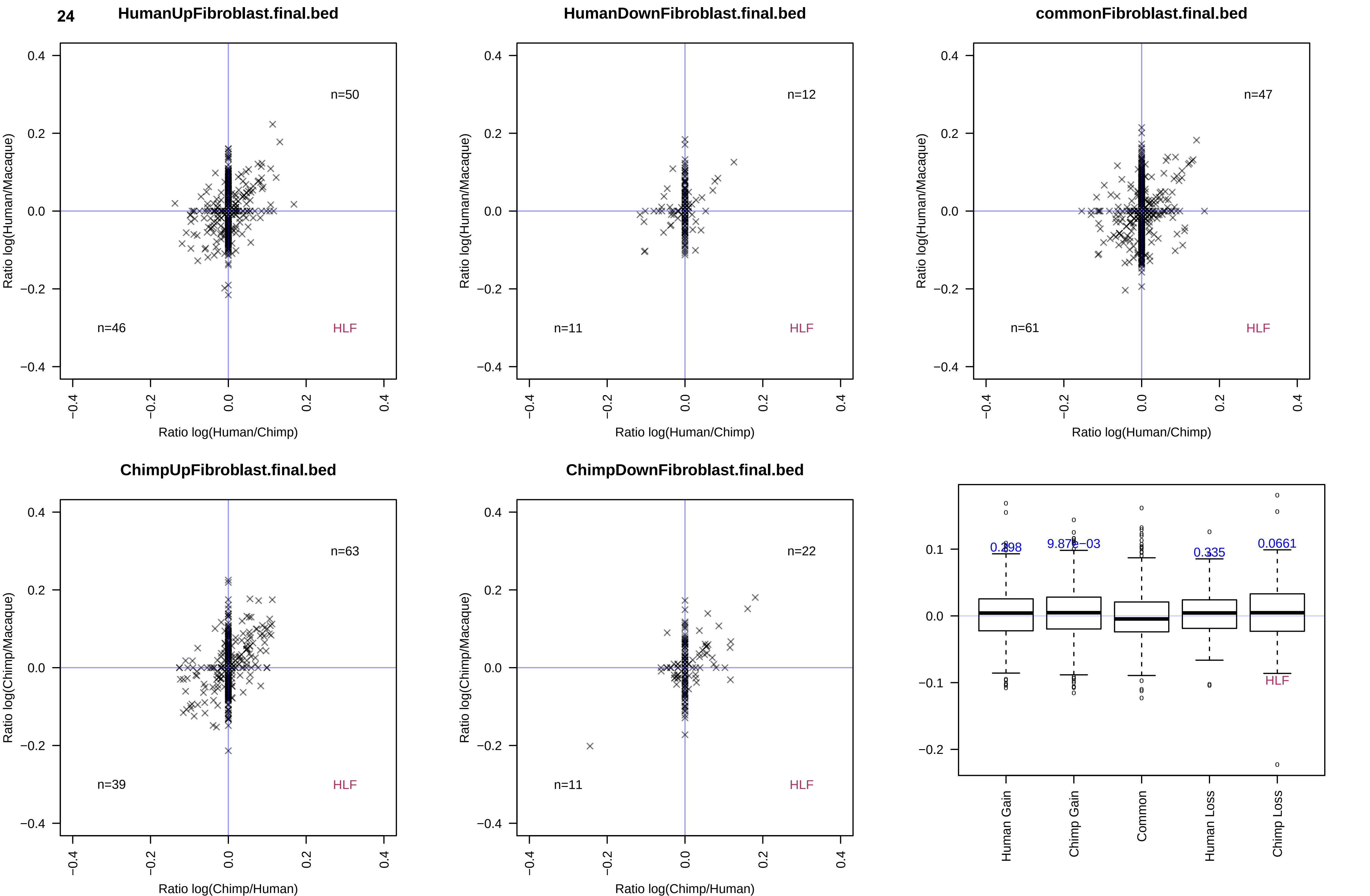
HumanUpFibroblast.final.bed
n=50
n=46
HLF
Ratio log(Human/Macaque)
Ratio log(Human/Chimp)
HumanDownFibroblast.final.bed
n=12
n=11
HLF
Ratio log(Human/Macaque)
Ratio log(Human/Chimp)
commonFibroblast.final.bed
n=47
n=61
HLF
Ratio log(Human/Macaque)
Ratio log(Human/Chimp)
ChimpUpFibroblast.final.bed
n=63
n=39
HLF
Ratio log(Chimp/Macaque)
Ratio log(Chimp/Human)
ChimpDownFibroblast.final.bed
n=22
n=11
HLF
Ratio log(Chimp/Macaque)
Ratio log(Chimp/Human)
0.298
9.87e−03
0.335
0.0661
HLF
Human Gain
Chimp Gain
Common
Human Loss
Chimp Loss

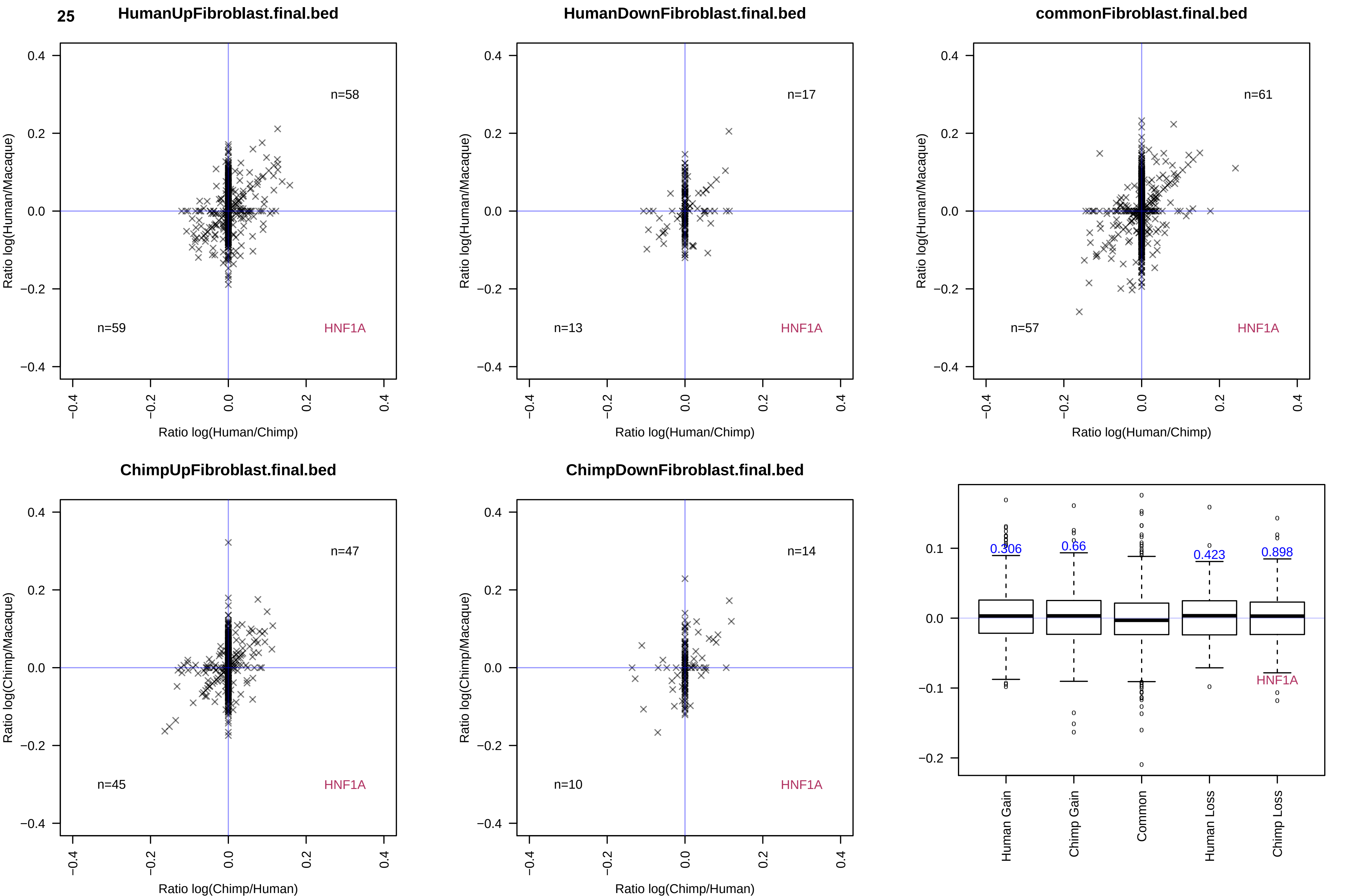
HumanUpFibroblast.final.bed
n=58
n=59
HNF1A
Ratio log(Human/Macaque)
Ratio log(Human/Chimp)
HumanDownFibroblast.final.bed
n=17
n=13
HNF1A
Ratio log(Human/Macaque)
Ratio log(Human/Chimp)
commonFibroblast.final.bed
n=61
n=57
HNF1A
Ratio log(Human/Macaque)
Ratio log(Human/Chimp)
ChimpUpFibroblast.final.bed
n=47
n=45
HNF1A
Ratio log(Chimp/Macaque)
Ratio log(Chimp/Human)
ChimpDownFibroblast.final.bed
n=14
n=10
HNF1A
Ratio log(Chimp/Macaque)
Ratio log(Chimp/Human)
0.306
0.66
0.423
0.898
HNF1A
Human Gain
Chimp Gain
Common
Human Loss
Chimp Loss

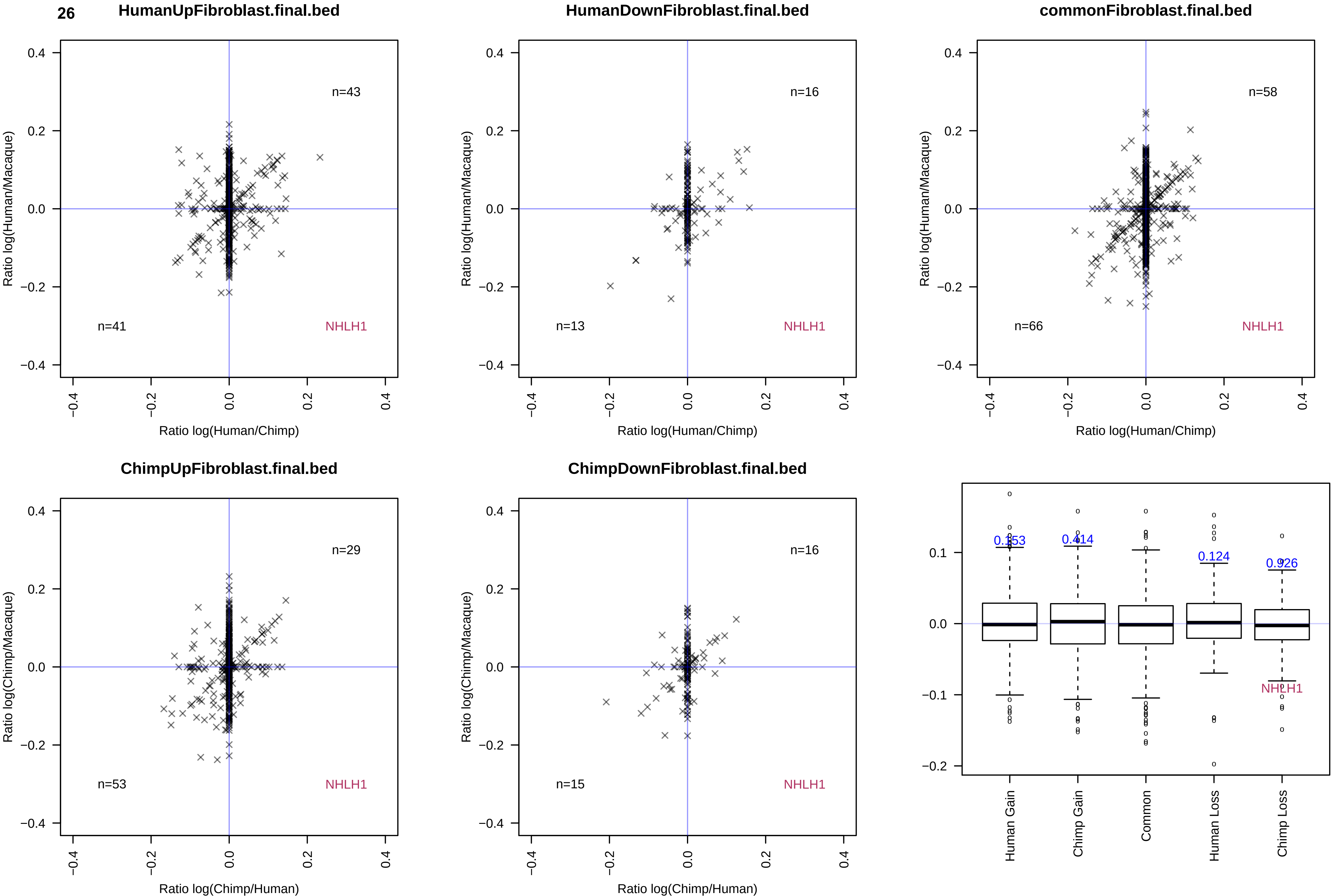
HumanUpFibroblast.final.bed
n=43
n=41
NHLH1
Ratio log(Human/Chimp)
Ratio log(Human/Macaque)
HumanDownFibroblast.final.bed
n=16
n=13
NHLH1
Ratio log(Human/Chimp)
Ratio log(Human/Macaque)
commonFibroblast.final.bed
n=58
n=66
NHLH1
Ratio log(Human/Chimp)
Ratio log(Human/Macaque)
ChimpUpFibroblast.final.bed
n=29
n=53
NHLH1
Ratio log(Chimp/Human)
Ratio log(Chimp/Macaque)
ChimpDownFibroblast.final.bed
n=16
n=15
NHLH1
Ratio log(Chimp/Human)
Ratio log(Chimp/Macaque)
0.153
0.414
0.124
0.926
NHLH1
Human Gain
Chimp Gain
Common
Human Loss
Chimp Loss

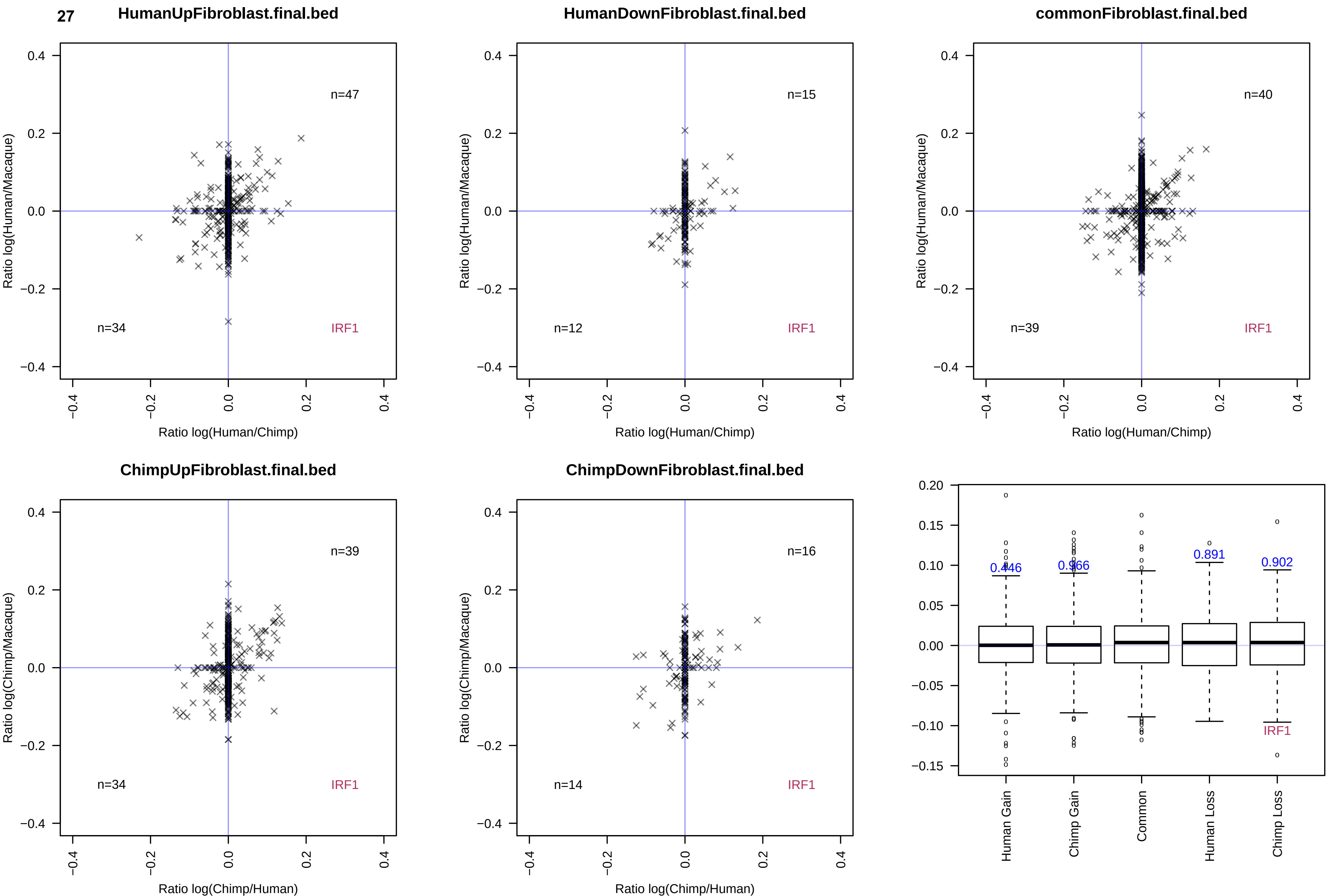
HumanUpFibroblast.final.bed
n=47
n=34
IRF1
Ratio log(Human/Macaque)
Ratio log(Human/Chimp)
HumanDownFibroblast.final.bed
n=15
n=12
IRF1
Ratio log(Human/Macaque)
Ratio log(Human/Chimp)
commonFibroblast.final.bed
n=40
n=39
IRF1
Ratio log(Human/Macaque)
Ratio log(Human/Chimp)
ChimpUpFibroblast.final.bed
n=39
n=34
IRF1
Ratio log(Chimp/Macaque)
Ratio log(Chimp/Human)
ChimpDownFibroblast.final.bed
n=16
n=14
IRF1
Ratio log(Chimp/Macaque)
Ratio log(Chimp/Human)
0.446
0.966
0.891
0.902
IRF1
Human Gain
Chimp Gain
Common
Human Loss
Chimp Loss

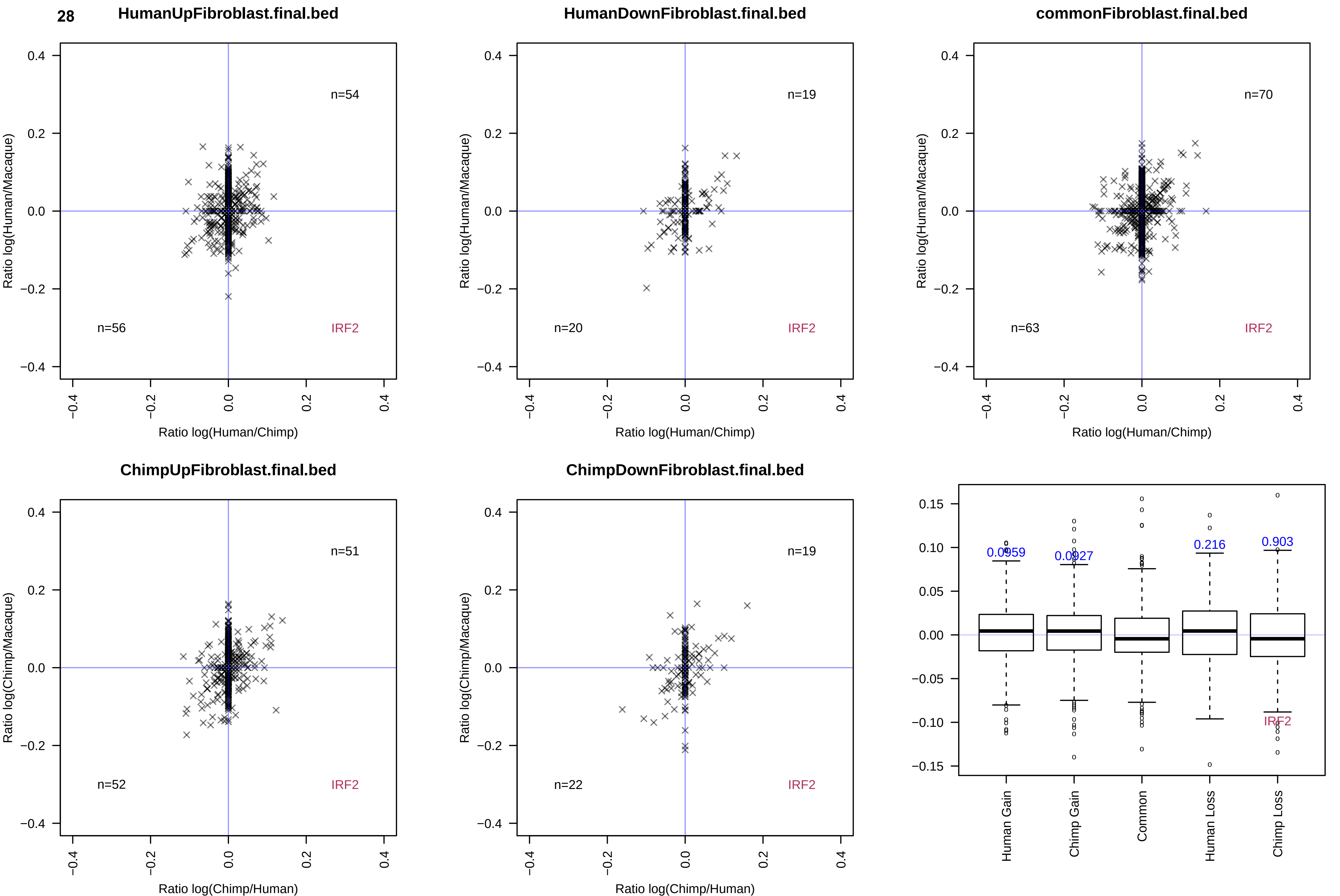

HumanUpFibroblast.final.bed
HumanDownFibroblast.final.bed
commonFibroblast.final.bed
ChimpUpFibroblast.final.bed
ChimpDownFibroblast.final.bed
Ratio log(Human/Macaque)
Ratio log(Human/Chimp)
Ratio log(Chimp/Macaque)
Ratio log(Chimp/Human)
n=54
n=56
n=19
n=20
n=70
n=63
n=51
n=52
n=22
IRF2
0.0959
0.0927
0.216
0.903
Human Gain
Chimp Gain
Common
Human Loss
Chimp Loss

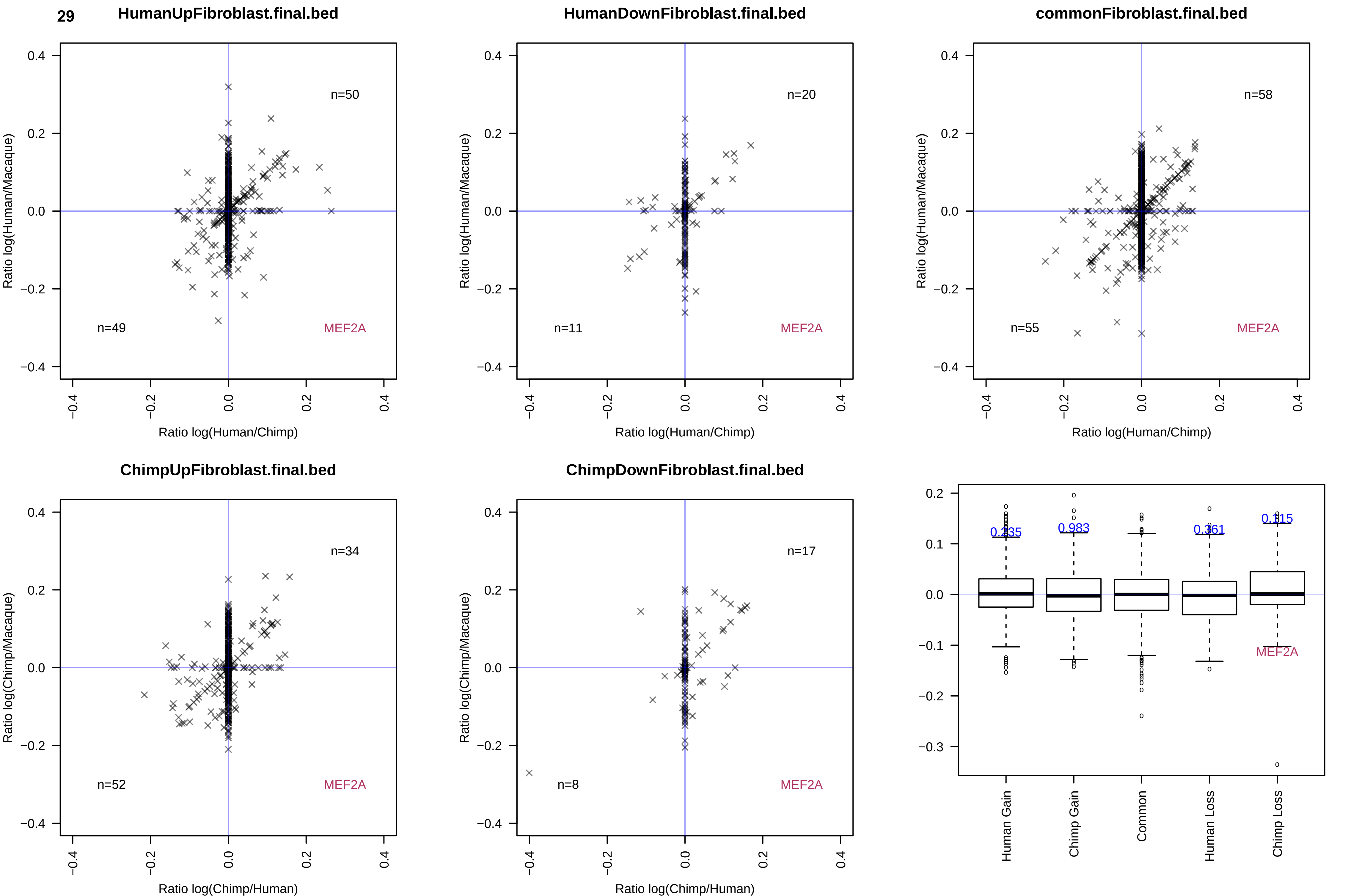
HumanUpFibroblast.final.bed
HumanDownFibroblast.final.bed
commonFibroblast.final.bed
ChimpUpFibroblast.final.bed
ChimpDownFibroblast.final.bed
Ratio log(Human/Macaque)
Ratio log(Human/Chimp)
Ratio log(Chimp/Macaque)
Ratio log(Chimp/Human)
n=50
n=49
n=20
n=11
n=58
n=55
n=34
n=52
n=17
n=8
MEF2A
0.235
0.983
0.361
0.315
Human Gain
Chimp Gain
Common
Human Loss
Chimp Loss

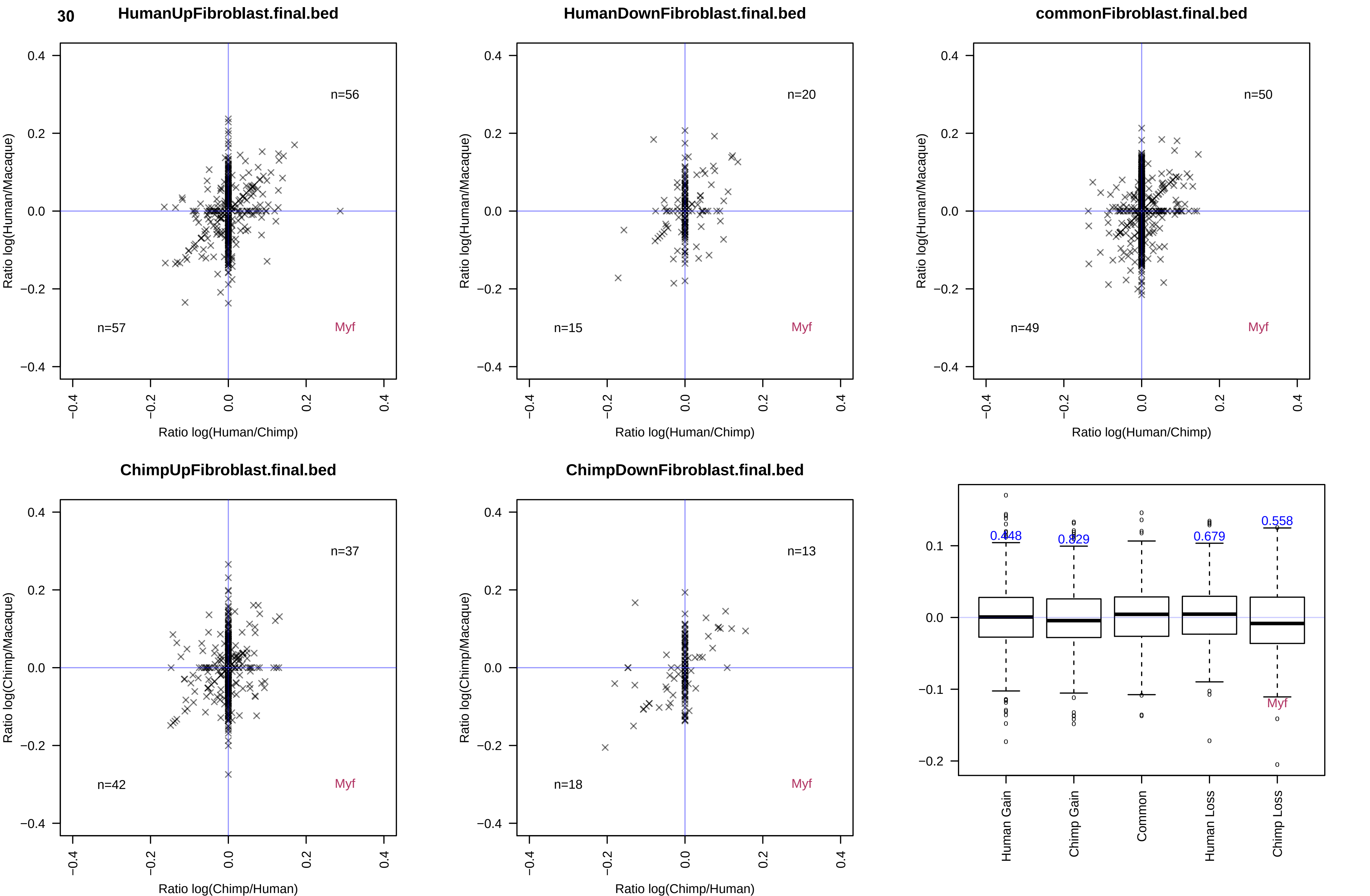
HumanUpFibroblast.final.bed
n=56
n=57
Myf
Ratio log(Human/Macaque)
Ratio log(Human/Chimp)
HumanDownFibroblast.final.bed
n=20
n=15
Myf
Ratio log(Human/Macaque)
Ratio log(Human/Chimp)
commonFibroblast.final.bed
n=50
n=49
Myf
Ratio log(Human/Macaque)
Ratio log(Human/Chimp)
ChimpUpFibroblast.final.bed
n=37
n=42
Myf
Ratio log(Chimp/Macaque)
Ratio log(Chimp/Human)
ChimpDownFibroblast.final.bed
n=13
n=18
Myf
Ratio log(Chimp/Macaque)
Ratio log(Chimp/Human)
0.448
0.829
0.679
0.558
Myf
Human Gain
Chimp Gain
Common
Human Loss
Chimp Loss

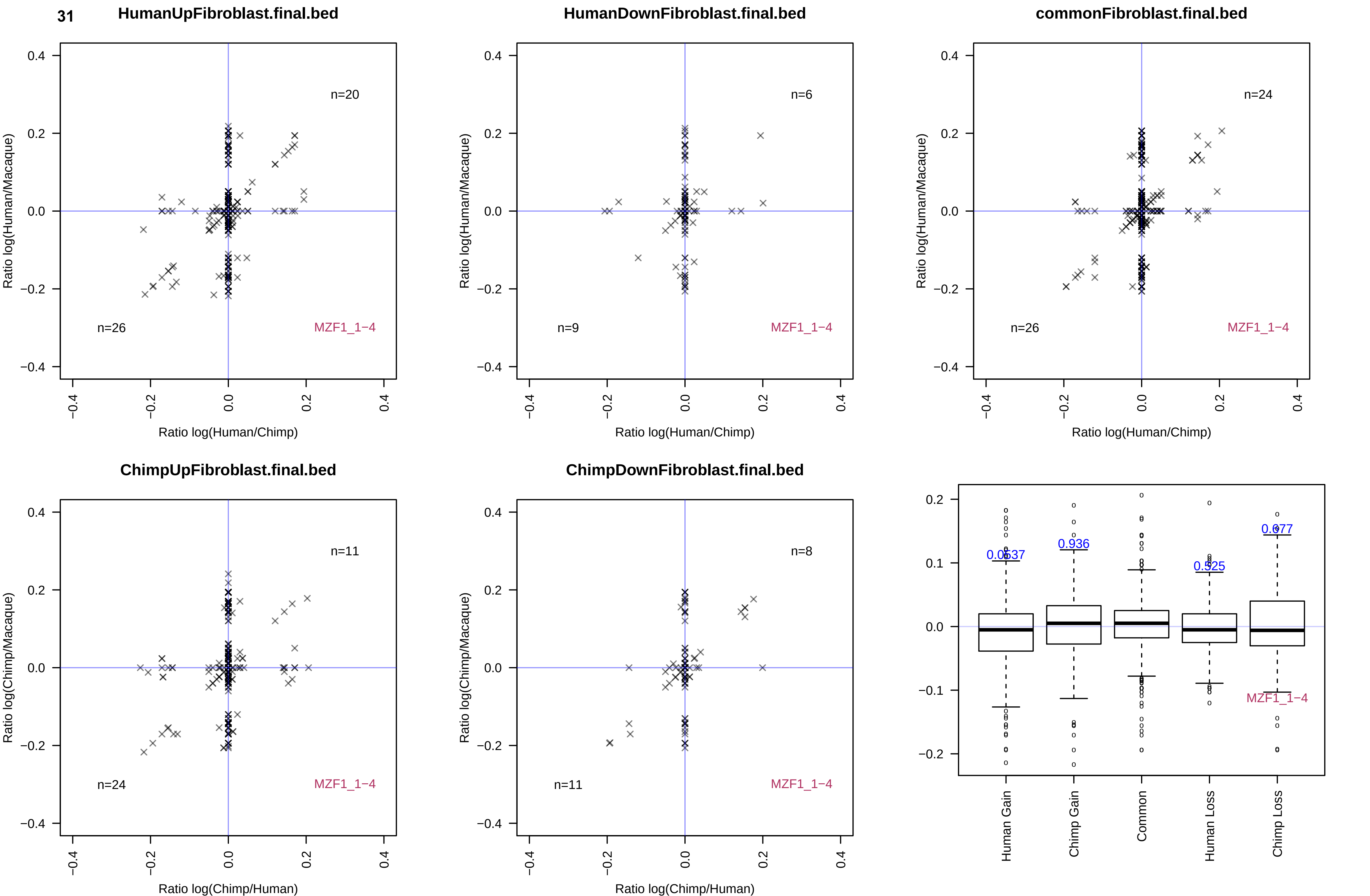
HumanUpFibroblast.final.bed
HumanDownFibroblast.final.bed
commonFibroblast.final.bed
ChimpUpFibroblast.final.bed
ChimpDownFibroblast.final.bed
n=20
n=26
n=6
n=9
n=24
n=26
n=11
n=24
n=8
n=11
MZF1_1−4
Ratio log(Human/Chimp)
Ratio log(Human/Macaque)
Ratio log(Chimp/Human)
Ratio log(Chimp/Macaque)
0.0537
0.936
0.525
0.677
Human Gain
Chimp Gain
Common
Human Loss
Chimp Loss

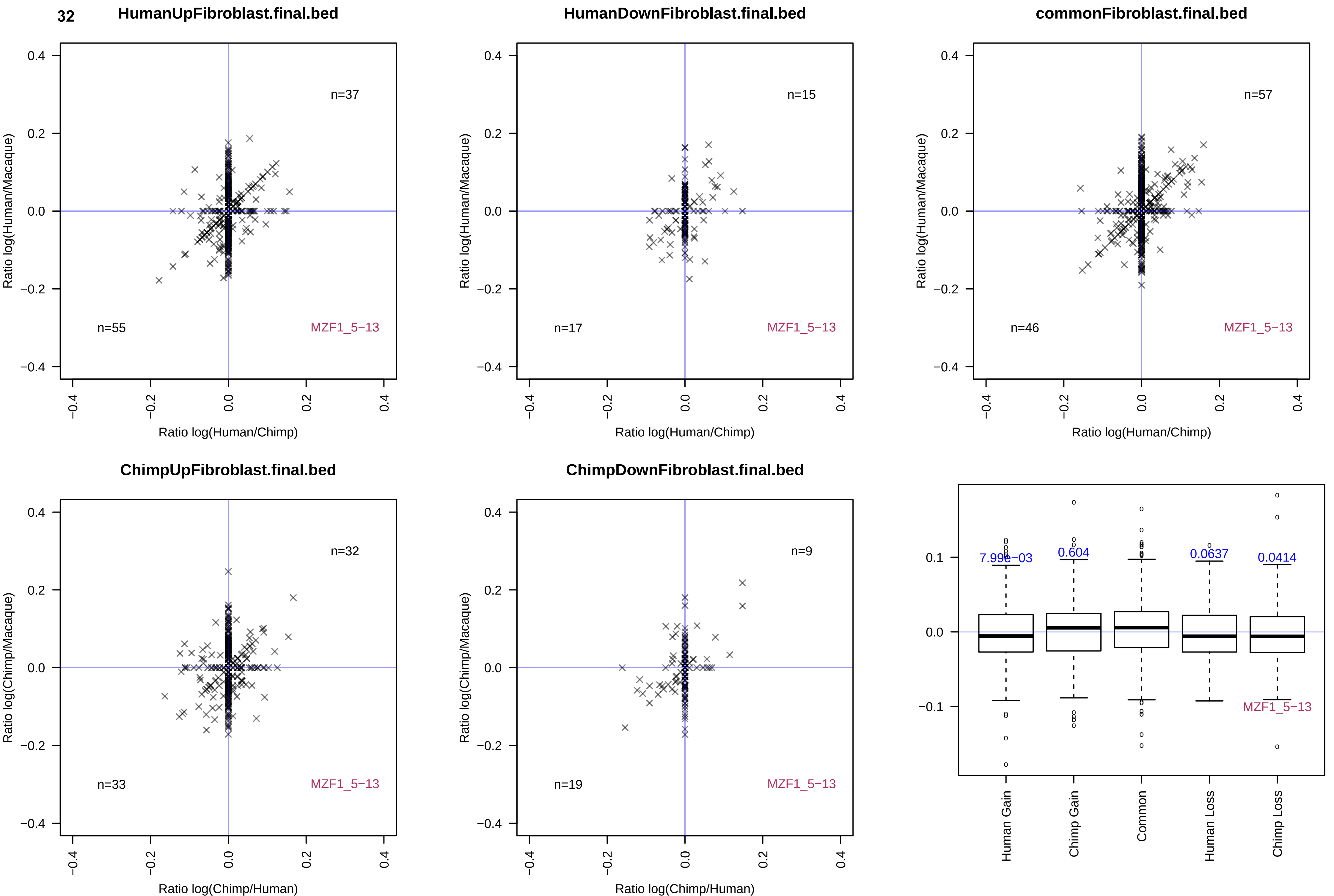
HumanUpFibroblast.final.bed
n=37
n=55
MZF1_5−13
HumanDownFibroblast.final.bed
n=15
n=17
MZF1_5−13
commonFibroblast.final.bed
n=57
n=46
MZF1_5−13
Ratio log(Human/Macaque)
Ratio log(Human/Chimp)
ChimpUpFibroblast.final.bed
n=32
n=33
MZF1_5−13
ChimpDownFibroblast.final.bed
n=9
n=19
MZF1_5−13
Ratio log(Chimp/Macaque)
Ratio log(Chimp/Human)
7.99e−03
0.604
0.0637
0.0414
MZF1_5−13
Human Gain
Chimp Gain
Common
Human Loss
Chimp Loss

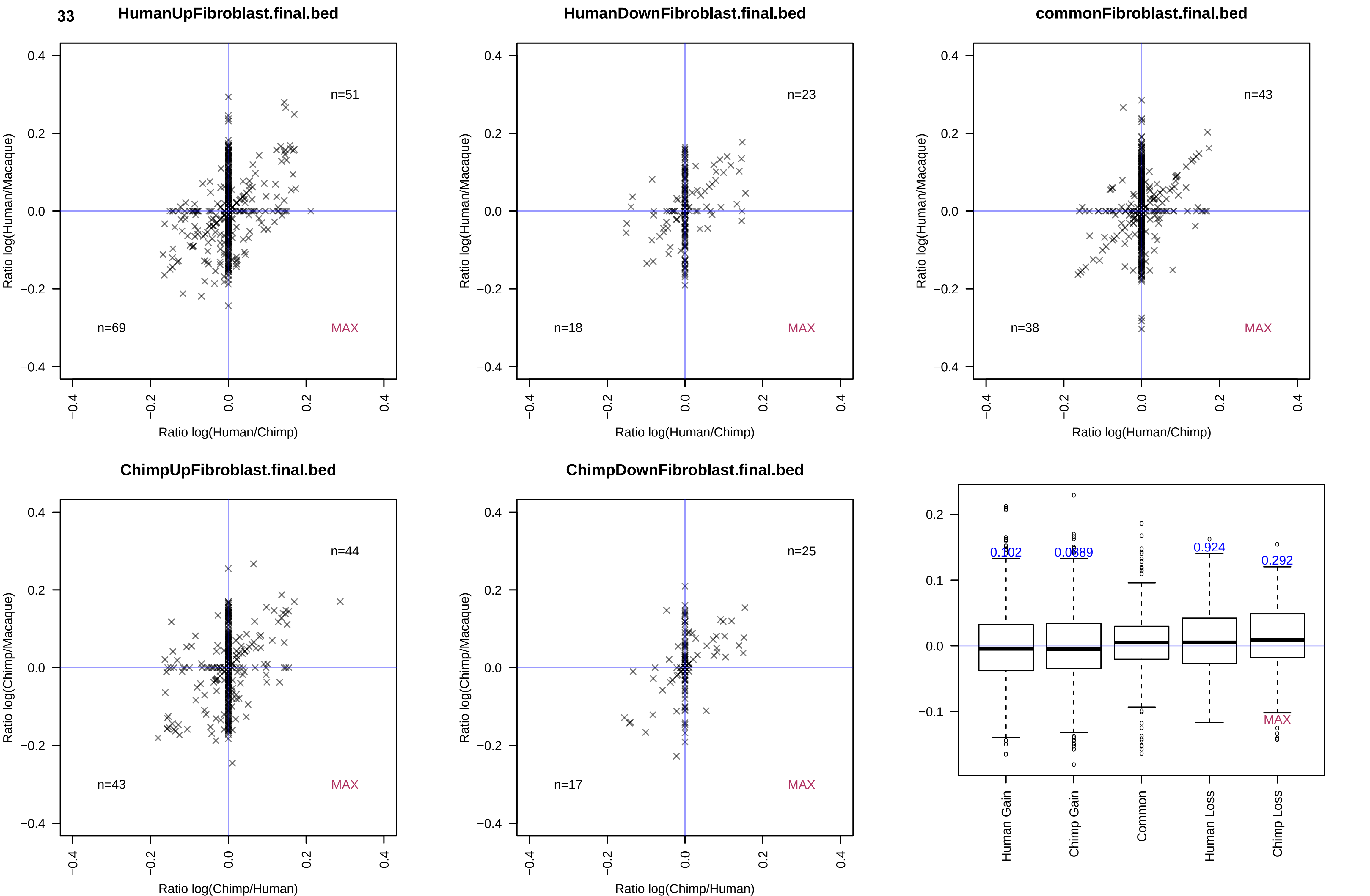
HumanUpFibroblast.final.bed
n=51
n=69
MAX
Ratio log(Human/Chimp)
Ratio log(Human/Macaque)
HumanDownFibroblast.final.bed
n=23
n=18
MAX
commonFibroblast.final.bed
n=43
n=38
MAX
ChimpUpFibroblast.final.bed
n=44
n=43
MAX
Ratio log(Chimp/Human)
Ratio log(Chimp/Macaque)
ChimpDownFibroblast.final.bed
n=25
n=17
MAX
0.102
0.0889
0.924
0.292
MAX
Human Gain
Chimp Gain
Common
Human Loss
Chimp Loss

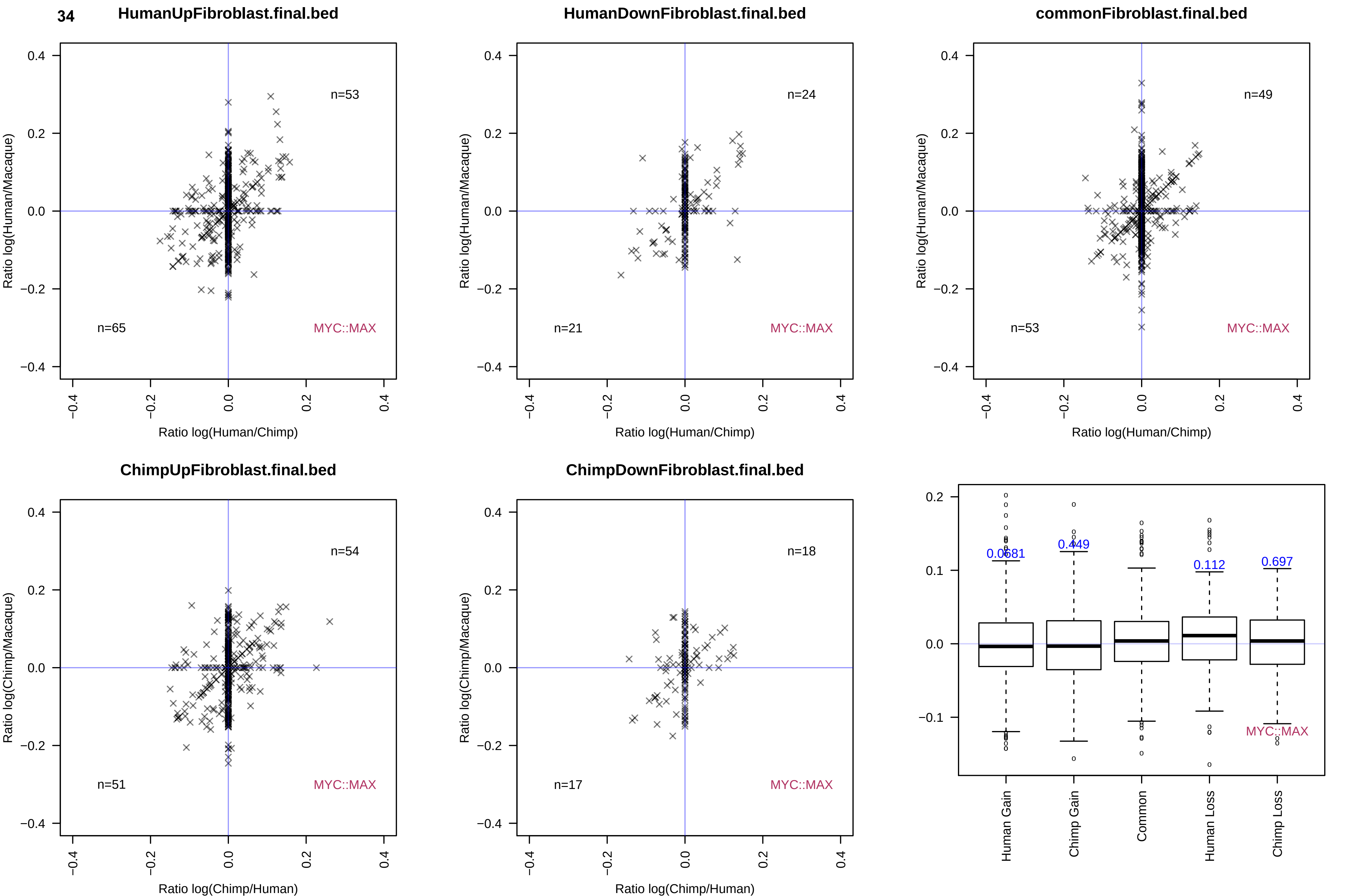
HumanUpFibroblast.final.bed
n=53
n=65
MYC::MAX
Ratio log(Human/Chimp)
Ratio log(Human/Macaque)
HumanDownFibroblast.final.bed
n=24
n=21
MYC::MAX
Ratio log(Human/Chimp)
Ratio log(Human/Macaque)
commonFibroblast.final.bed
n=49
n=53
MYC::MAX
Ratio log(Human/Chimp)
Ratio log(Human/Macaque)
ChimpUpFibroblast.final.bed
n=54
n=51
MYC::MAX
Ratio log(Chimp/Human)
Ratio log(Chimp/Macaque)
ChimpDownFibroblast.final.bed
n=18
n=17
MYC::MAX
Ratio log(Chimp/Human)
Ratio log(Chimp/Macaque)
0.0681
0.449
0.112
0.697
MYC::MAX
Human Gain
Chimp Gain
Common
Human Loss
Chimp Loss

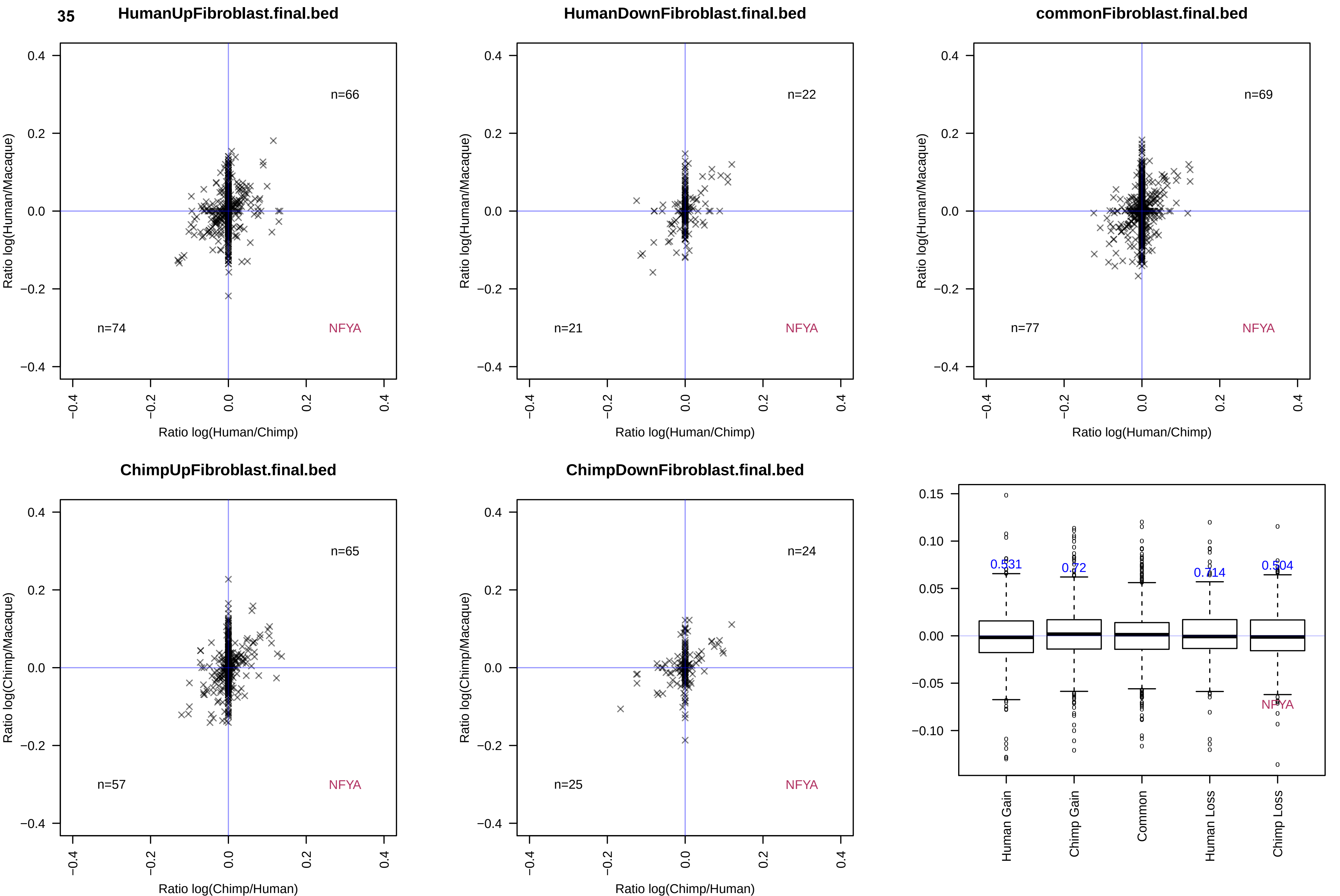
HumanUpFibroblast.final.bed
HumanDownFibroblast.final.bed
commonFibroblast.final.bed
ChimpUpFibroblast.final.bed
ChimpDownFibroblast.final.bed
Ratio log(Human/Macaque)
Ratio log(Human/Chimp)
Ratio log(Chimp/Macaque)
Ratio log(Chimp/Human)
n=66
n=74
n=22
n=21
n=69
n=77
n=65
n=57
n=24
n=25
NFYA
0.531
0.72
0.714
0.504
Human Gain
Chimp Gain
Common
Human Loss
Chimp Loss

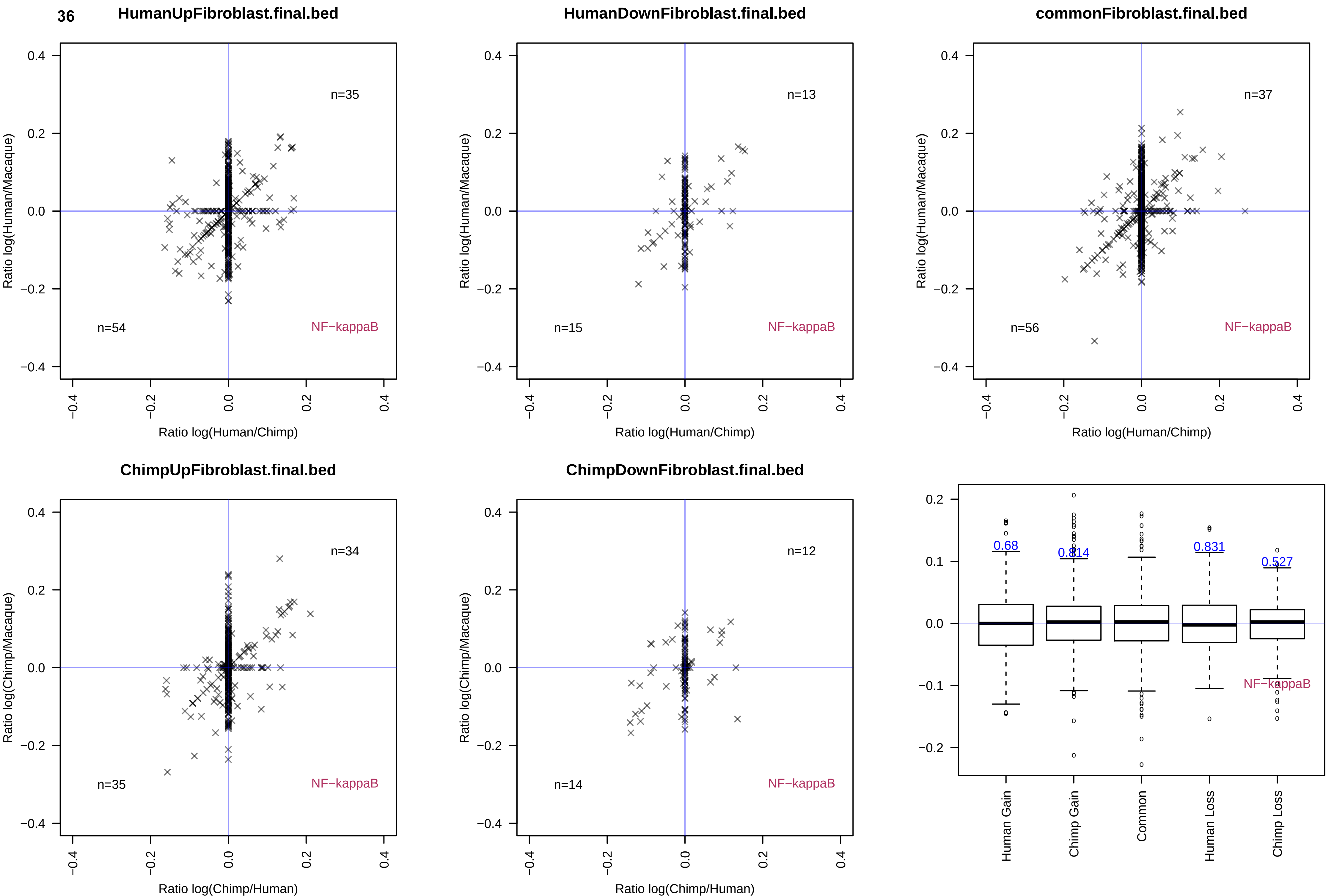
HumanUpFibroblast.final.bed
HumanDownFibroblast.final.bed
commonFibroblast.final.bed
ChimpUpFibroblast.final.bed
ChimpDownFibroblast.final.bed
n=35
n=54
n=13
n=15
n=37
n=56
n=34
n=35
n=12
n=14
NF−kappaB
Ratio log(Human/Macaque)
Ratio log(Human/Chimp)
Ratio log(Chimp/Macaque)
Ratio log(Chimp/Human)
0.68
0.814
0.831
0.527
Human Gain
Chimp Gain
Common
Human Loss
Chimp Loss

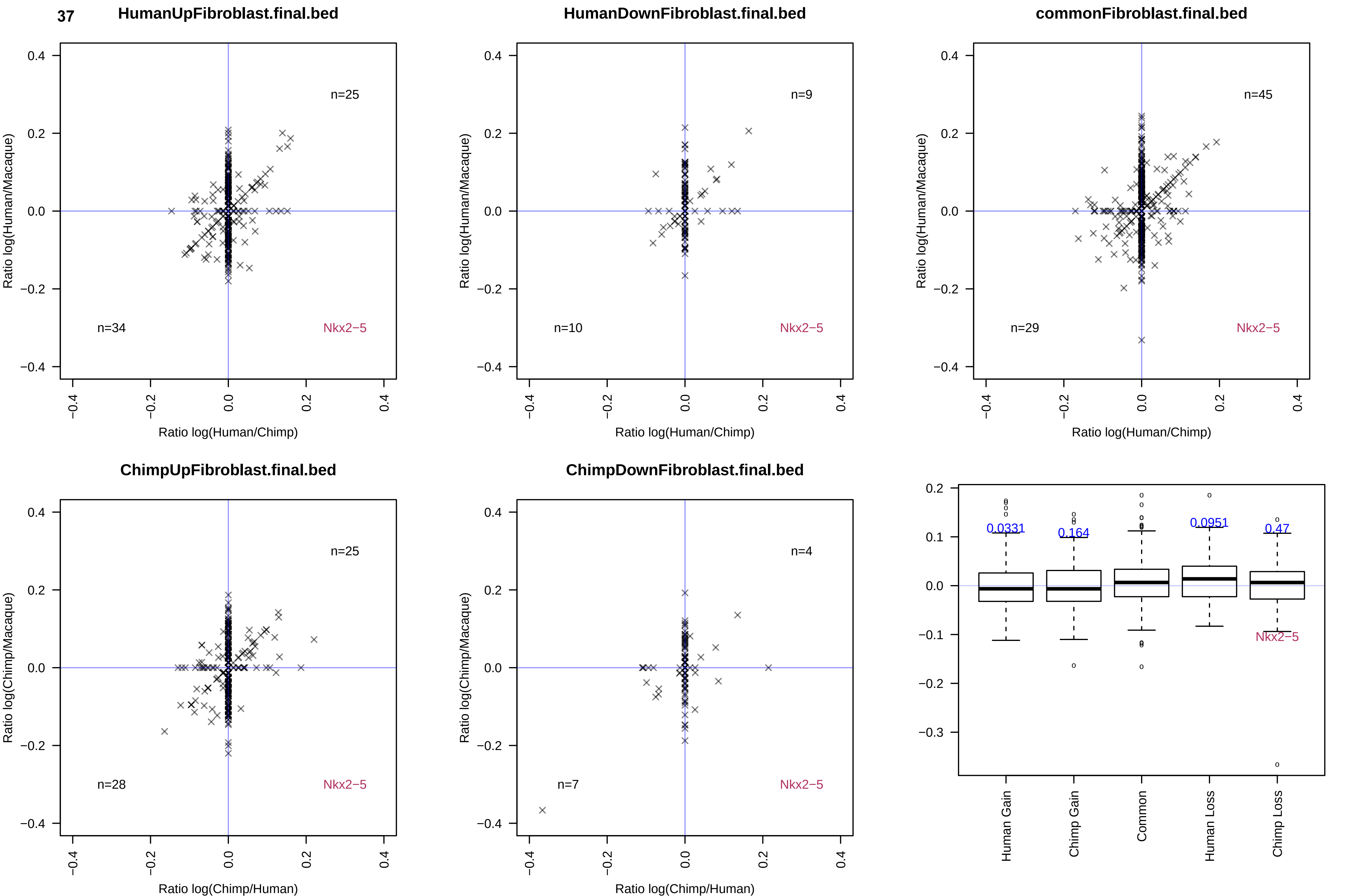
HumanUpFibroblast.final.bed
n=25
n=34
Nkx2−5
Ratio log(Human/Chimp)
Ratio log(Human/Macaque)
HumanDownFibroblast.final.bed
n=9
n=10
Nkx2−5
Ratio log(Human/Chimp)
Ratio log(Human/Macaque)
commonFibroblast.final.bed
n=45
n=29
Nkx2−5
Ratio log(Human/Chimp)
Ratio log(Human/Macaque)
ChimpUpFibroblast.final.bed
n=25
n=28
Nkx2−5
Ratio log(Chimp/Human)
Ratio log(Chimp/Macaque)
ChimpDownFibroblast.final.bed
n=4
n=7
Nkx2−5
Ratio log(Chimp/Human)
Ratio log(Chimp/Macaque)
0.0331
0.164
0.0951
0.47
Nkx2−5
Human Gain
Chimp Gain
Common
Human Loss
Chimp Loss

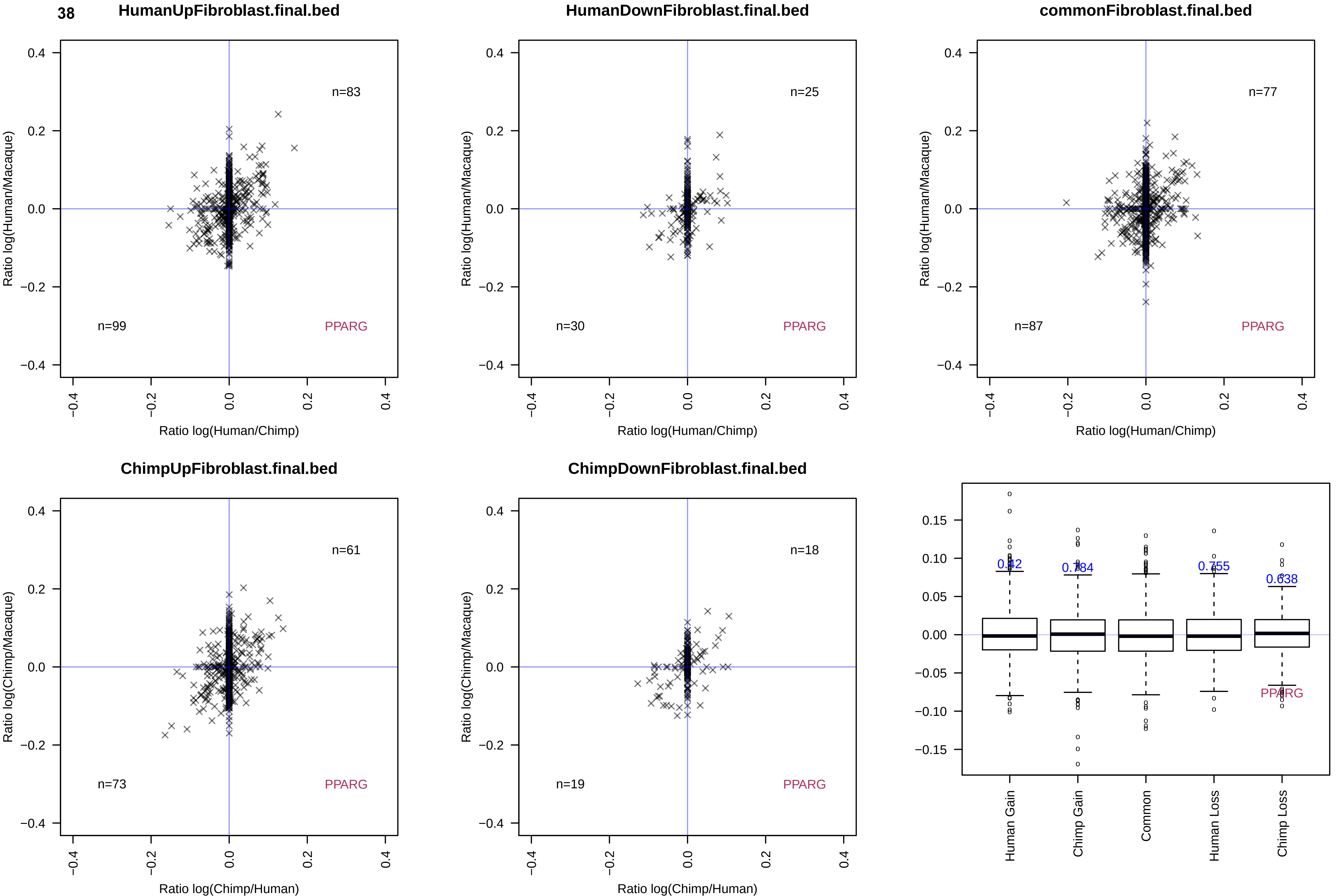
HumanUpFibroblast.final.bed
n=83
n=99
PPARG
Ratio log(Human/Macaque)
Ratio log(Human/Chimp)
HumanDownFibroblast.final.bed
n=25
n=30
PPARG
Ratio log(Human/Macaque)
Ratio log(Human/Chimp)
commonFibroblast.final.bed
n=77
n=87
PPARG
Ratio log(Human/Macaque)
Ratio log(Human/Chimp)
ChimpUpFibroblast.final.bed
n=61
n=73
PPARG
Ratio log(Chimp/Macaque)
Ratio log(Chimp/Human)
ChimpDownFibroblast.final.bed
n=18
n=19
PPARG
Ratio log(Chimp/Macaque)
Ratio log(Chimp/Human)
0.42
0.784
0.755
0.638
PPARG
Human Gain
Chimp Gain
Common
Human Loss
Chimp Loss

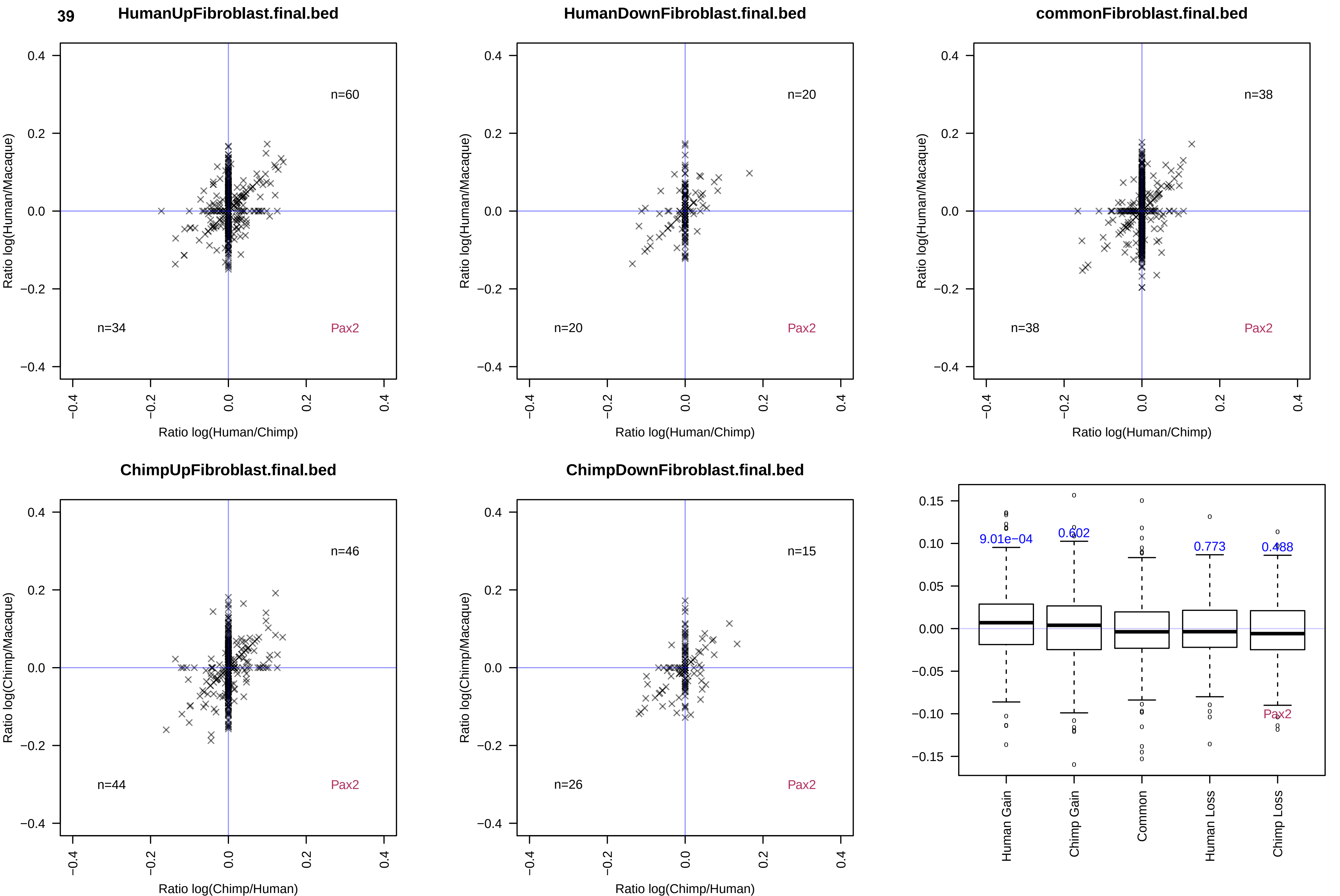
HumanUpFibroblast.final.bed
n=60
n=34
Pax2
Ratio log(Human/Chimp)
Ratio log(Human/Macaque)
HumanDownFibroblast.final.bed
n=20
n=20
Pax2
Ratio log(Human/Chimp)
Ratio log(Human/Macaque)
commonFibroblast.final.bed
n=38
n=38
Pax2
Ratio log(Human/Chimp)
Ratio log(Human/Macaque)
ChimpUpFibroblast.final.bed
n=46
n=44
Pax2
Ratio log(Chimp/Human)
Ratio log(Chimp/Macaque)
ChimpDownFibroblast.final.bed
n=15
n=26
Pax2
Ratio log(Chimp/Human)
Ratio log(Chimp/Macaque)
9.01e−04
0.602
0.773
0.488
Pax2
Human Gain
Chimp Gain
Common
Human Loss
Chimp Loss

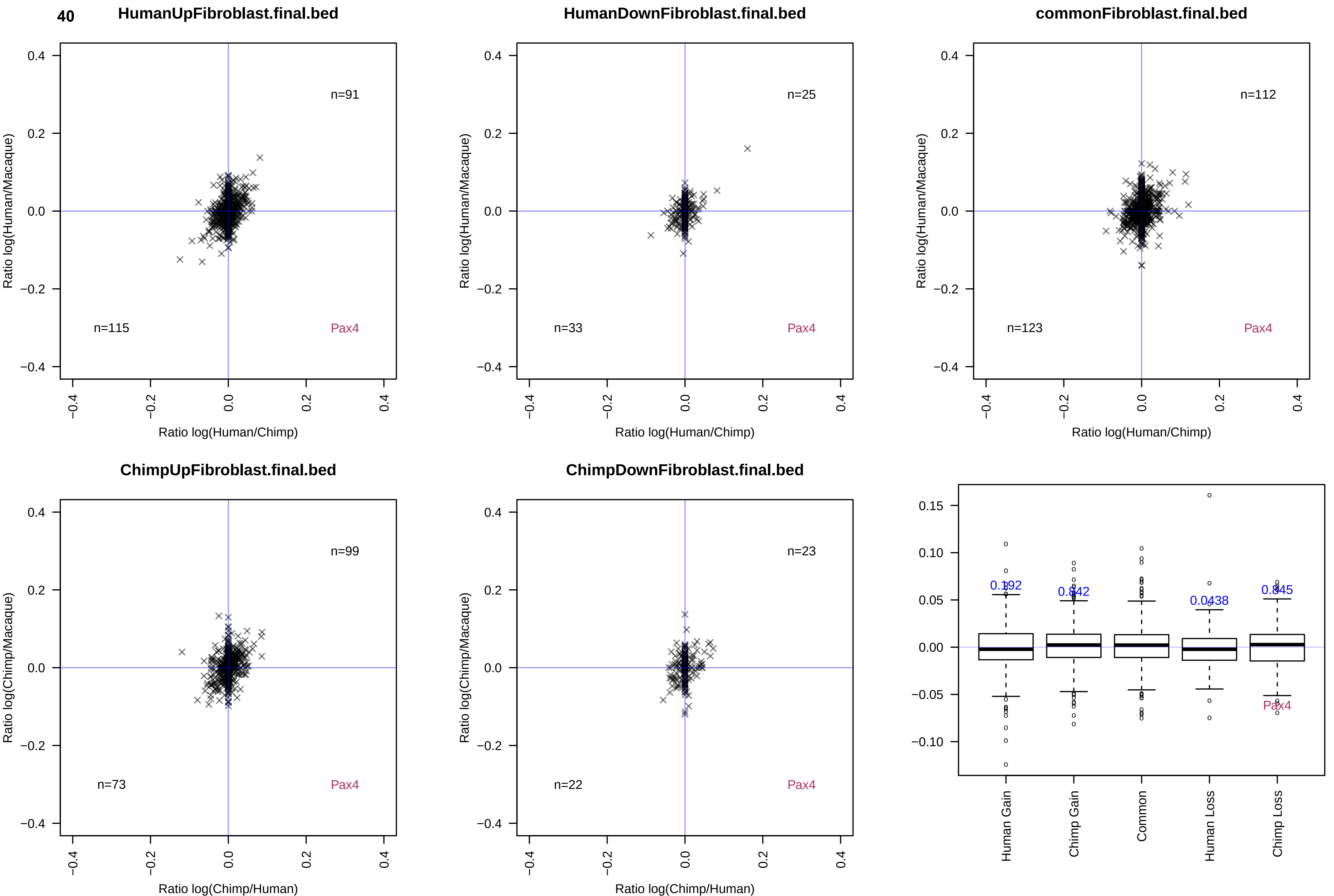
HumanUpFibroblast.final.bed
HumanDownFibroblast.final.bed
commonFibroblast.final.bed
ChimpUpFibroblast.final.bed
ChimpDownFibroblast.final.bed
Ratio log(Human/Macaque)
Ratio log(Human/Chimp)
Ratio log(Chimp/Macaque)
Ratio log(Chimp/Human)
n=91
n=115
n=25
n=33
n=112
n=123
n=99
n=73
n=23
n=22
Pax4
0.192
0.842
0.0438
0.845
Human Gain
Chimp Gain
Common
Human Loss
Chimp Loss

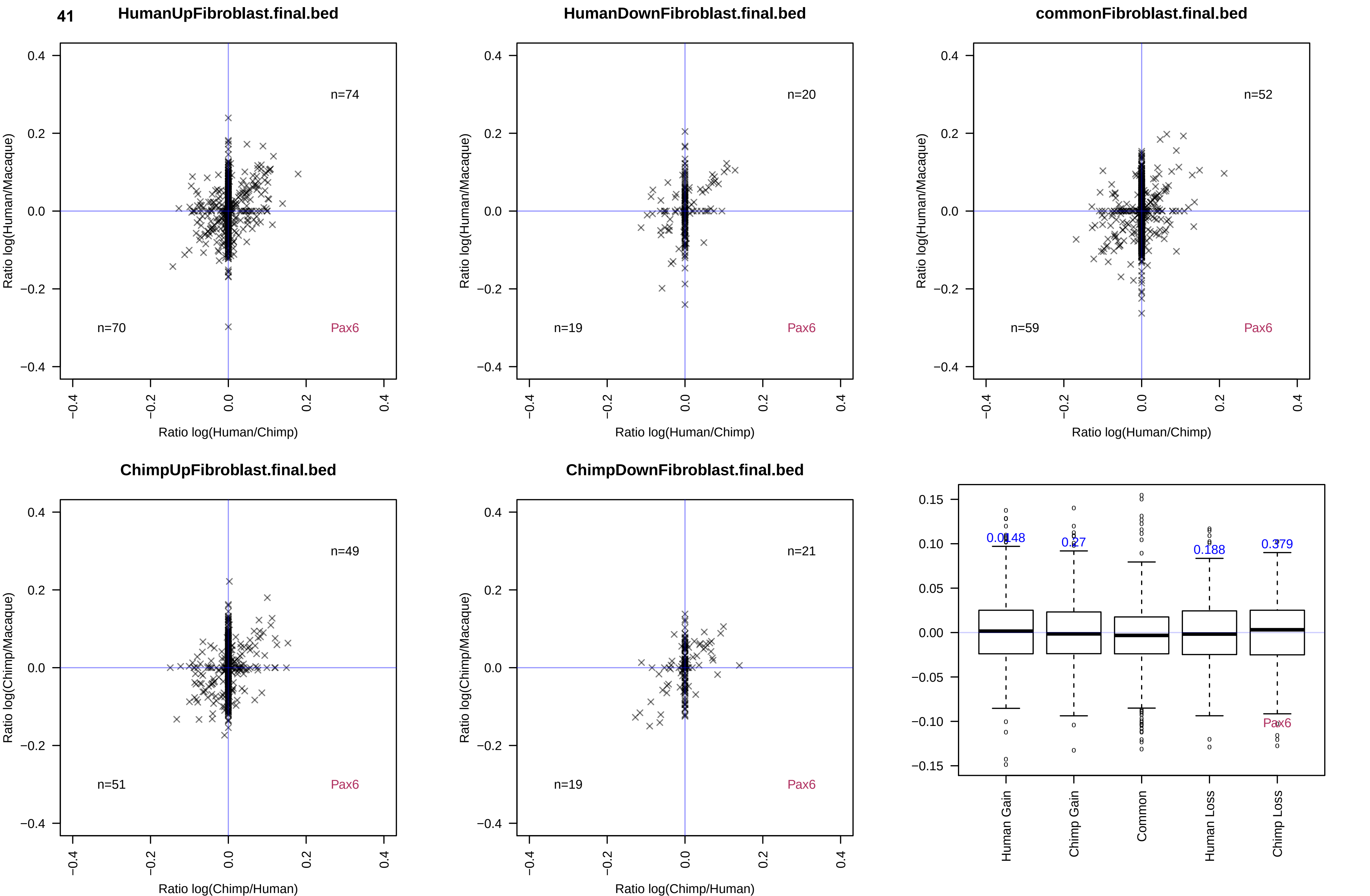
HumanUpFibroblast.final.bed
n=74
n=70
Pax6
HumanDownFibroblast.final.bed
n=20
n=19
Pax6
commonFibroblast.final.bed
n=52
n=59
Pax6
Ratio log(Human/Macaque)
Ratio log(Human/Chimp)
ChimpUpFibroblast.final.bed
n=49
n=51
Pax6
ChimpDownFibroblast.final.bed
n=21
n=19
Pax6
Ratio log(Chimp/Macaque)
Ratio log(Chimp/Human)
0.0148
0.27
0.188
0.379
Pax6
Human Gain
Chimp Gain
Common
Human Loss
Chimp Loss

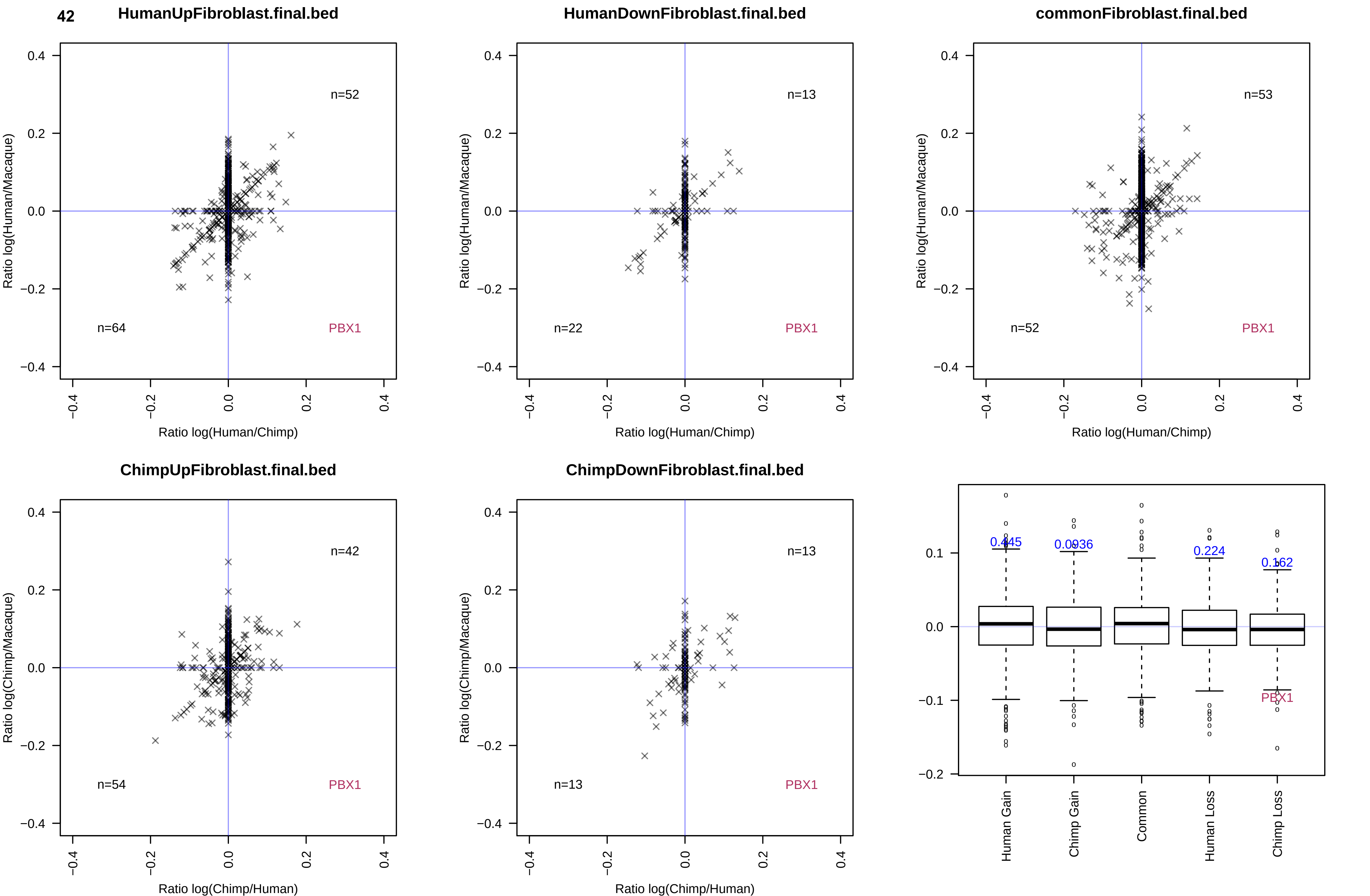
HumanUpFibroblast.final.bed
n=52
n=64
PBX1
Ratio log(Human/Macaque)
Ratio log(Human/Chimp)
HumanDownFibroblast.final.bed
n=13
n=22
PBX1
Ratio log(Human/Macaque)
Ratio log(Human/Chimp)
commonFibroblast.final.bed
n=53
n=52
PBX1
Ratio log(Human/Macaque)
Ratio log(Human/Chimp)
ChimpUpFibroblast.final.bed
n=42
n=54
PBX1
Ratio log(Chimp/Macaque)
Ratio log(Chimp/Human)
ChimpDownFibroblast.final.bed
n=13
n=13
PBX1
Ratio log(Chimp/Macaque)
Ratio log(Chimp/Human)
0.445
0.0936
0.224
0.162
PBX1
Human Gain
Chimp Gain
Common
Human Loss
Chimp Loss

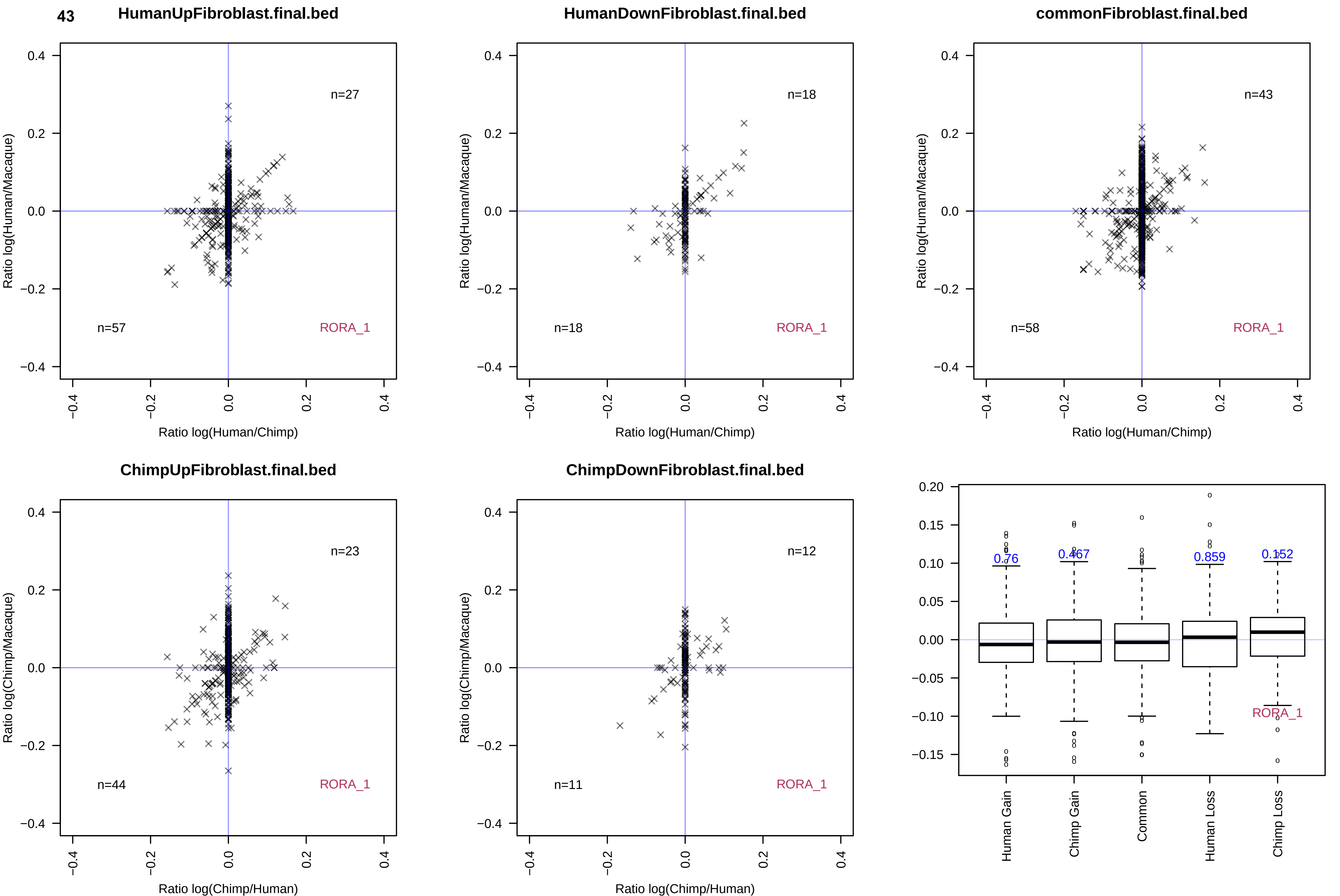
HumanUpFibroblast.final.bed
HumanDownFibroblast.final.bed
commonFibroblast.final.bed
ChimpUpFibroblast.final.bed
ChimpDownFibroblast.final.bed
Ratio log(Human/Macaque)
Ratio log(Human/Chimp)
Ratio log(Chimp/Macaque)
Ratio log(Chimp/Human)
n=27
n=57
n=18
n=18
n=43
n=58
n=23
n=44
n=12
n=11
RORA_1
0.76
0.467
0.859
0.152
Human Gain
Chimp Gain
Common
Human Loss
Chimp Loss

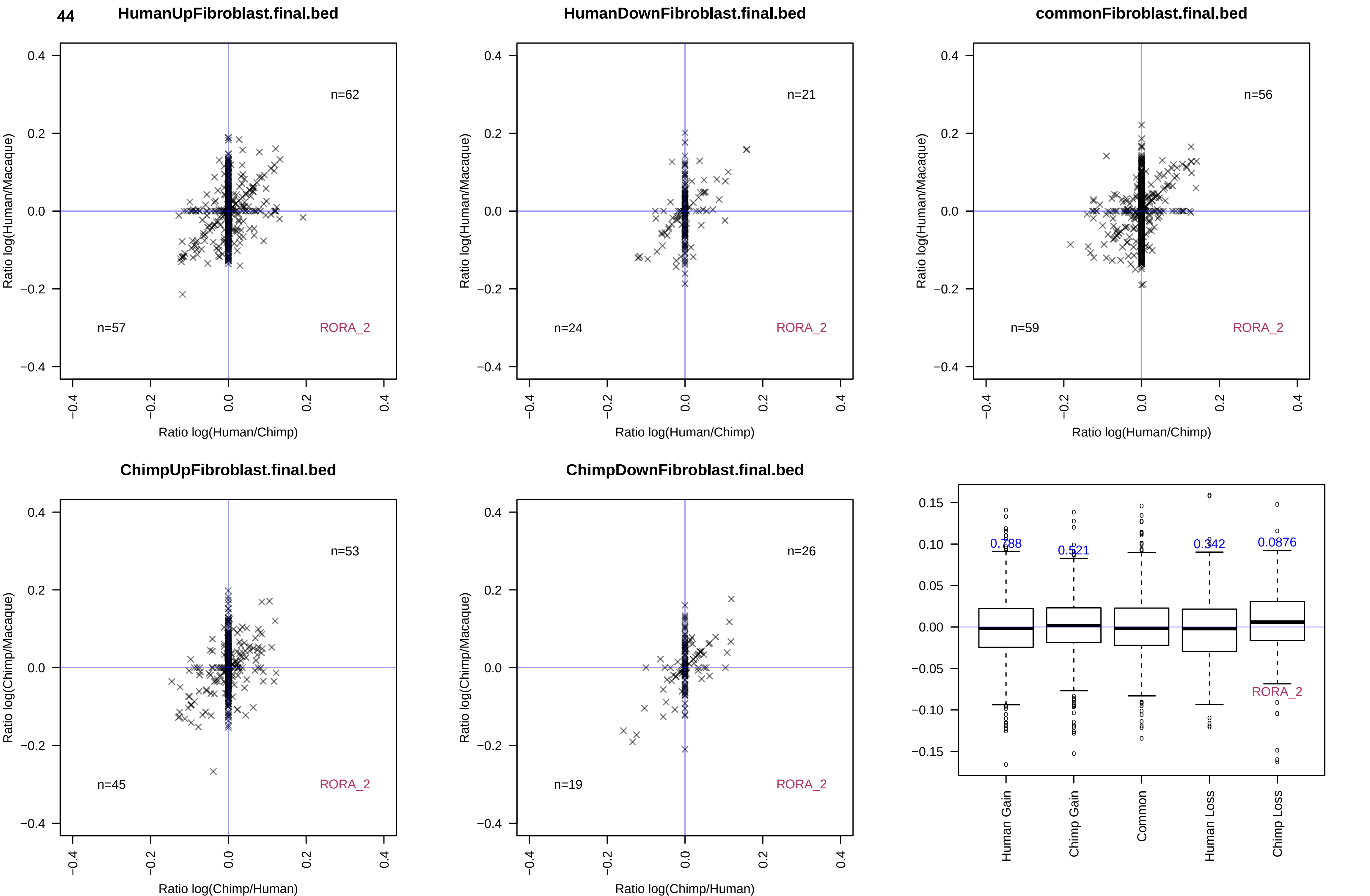
HumanUpFibroblast.final.bed
n=62
n=57
RORA_2
Ratio log(Human/Macaque)
Ratio log(Human/Chimp)
HumanDownFibroblast.final.bed
n=21
n=24
RORA_2
Ratio log(Human/Macaque)
Ratio log(Human/Chimp)
commonFibroblast.final.bed
n=56
n=59
RORA_2
Ratio log(Human/Macaque)
Ratio log(Human/Chimp)
ChimpUpFibroblast.final.bed
n=53
n=45
RORA_2
Ratio log(Chimp/Macaque)
Ratio log(Chimp/Human)
ChimpDownFibroblast.final.bed
n=26
n=19
RORA_2
Ratio log(Chimp/Macaque)
Ratio log(Chimp/Human)
0.788
0.521
0.342
0.0876
RORA_2
Human Gain
Chimp Gain
Common
Human Loss
Chimp Loss

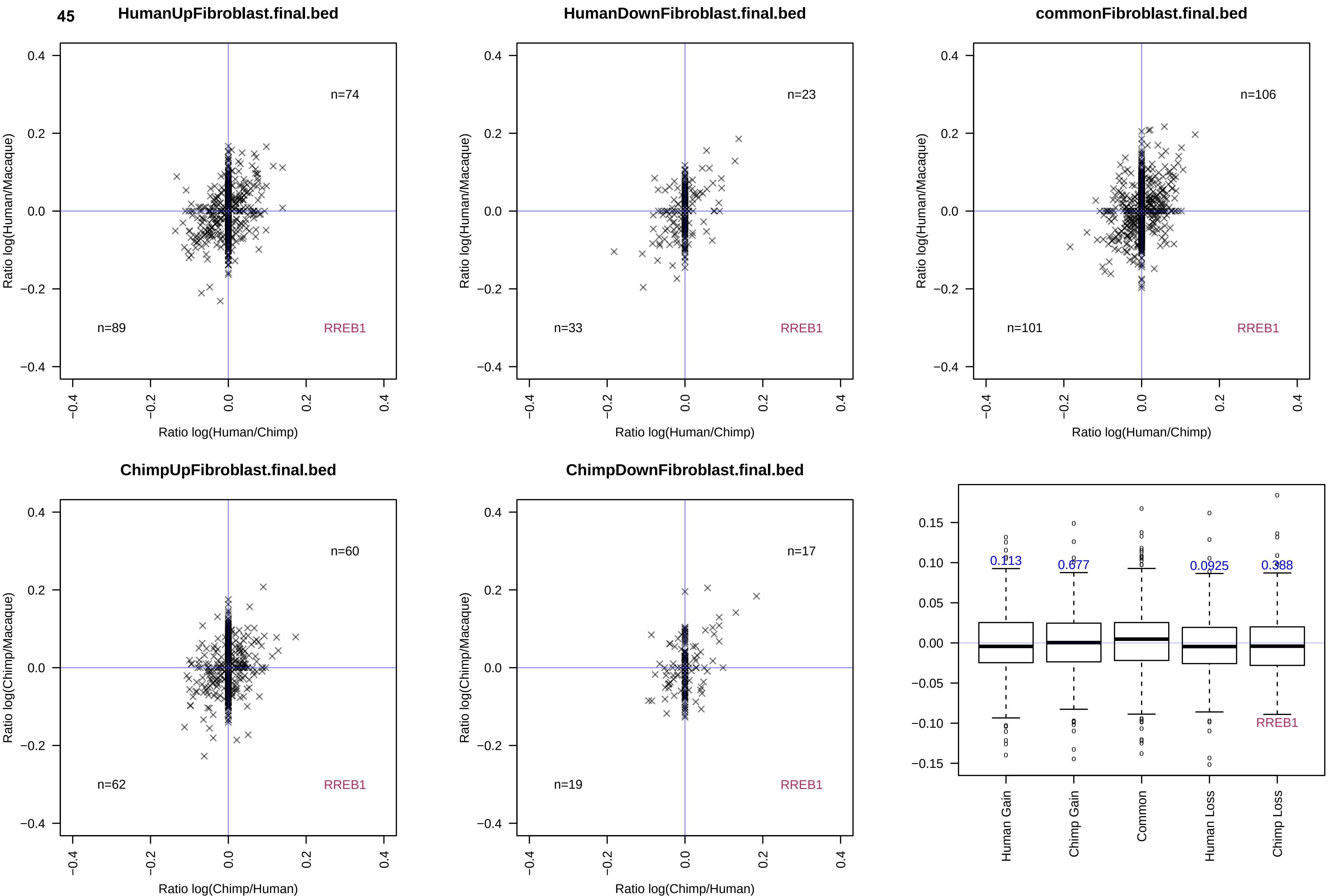
HumanUpFibroblast.final.bed
n=74
n=89
RREB1
Ratio log(Human/Macaque)
Ratio log(Human/Chimp)
HumanDownFibroblast.final.bed
n=23
n=33
RREB1
Ratio log(Human/Macaque)
Ratio log(Human/Chimp)
commonFibroblast.final.bed
n=106
n=101
RREB1
Ratio log(Human/Macaque)
Ratio log(Human/Chimp)
ChimpUpFibroblast.final.bed
n=60
n=62
RREB1
Ratio log(Chimp/Macaque)
Ratio log(Chimp/Human)
ChimpDownFibroblast.final.bed
n=17
n=19
RREB1
Ratio log(Chimp/Macaque)
Ratio log(Chimp/Human)
0.113
0.677
0.0925
0.388
RREB1
Human Gain
Chimp Gain
Common
Human Loss
Chimp Loss

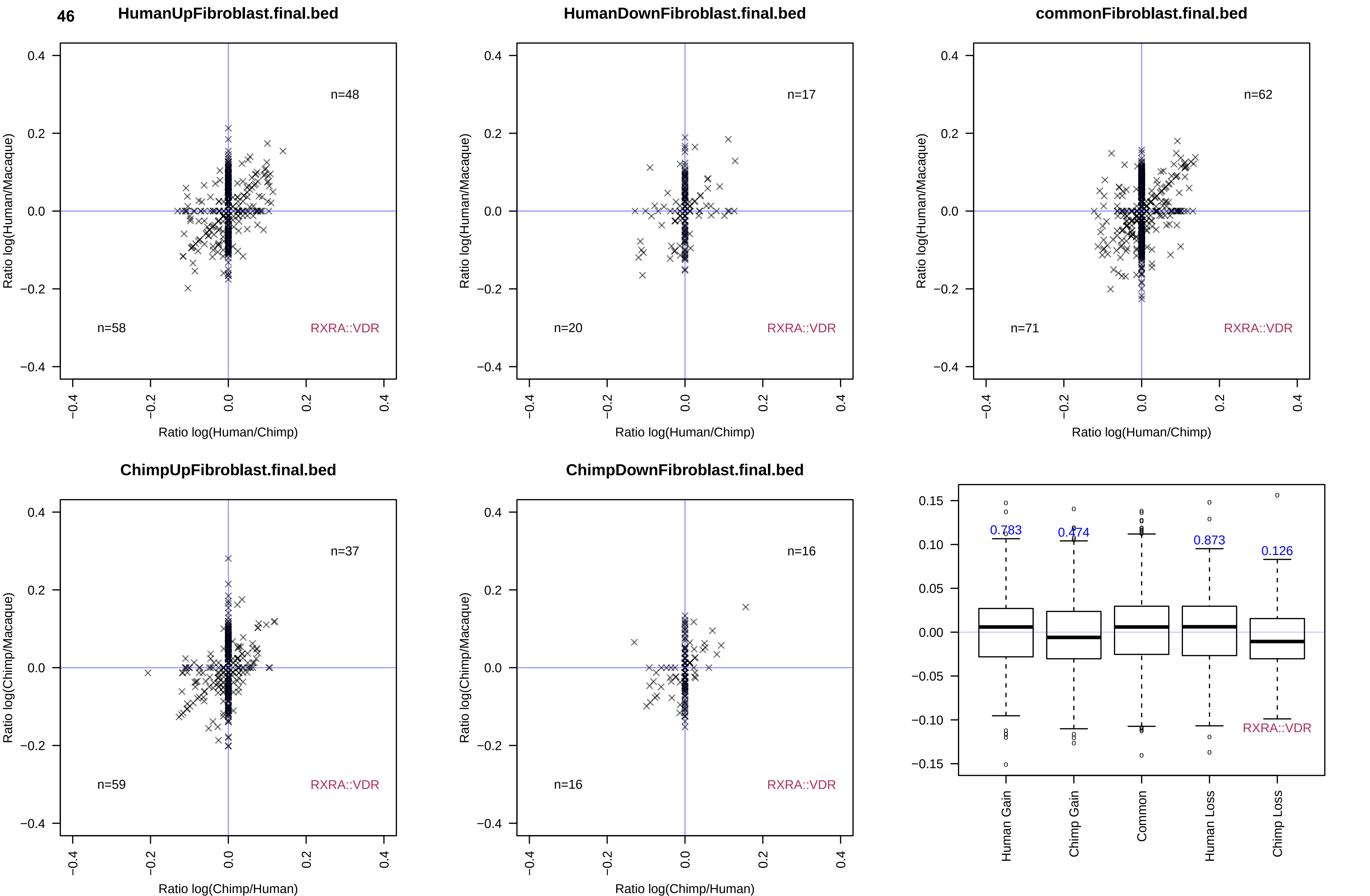
HumanUpFibroblast.final.bed
HumanDownFibroblast.final.bed
commonFibroblast.final.bed
ChimpUpFibroblast.final.bed
ChimpDownFibroblast.final.bed
n=48
n=58
n=17
n=20
n=62
n=71
n=37
n=59
n=16
n=16
RXRA::VDR
Ratio log(Human/Macaque)
Ratio log(Human/Chimp)
Ratio log(Chimp/Macaque)
Ratio log(Chimp/Human)
0.783
0.474
0.873
0.126
Human Gain
Chimp Gain
Common
Human Loss
Chimp Loss

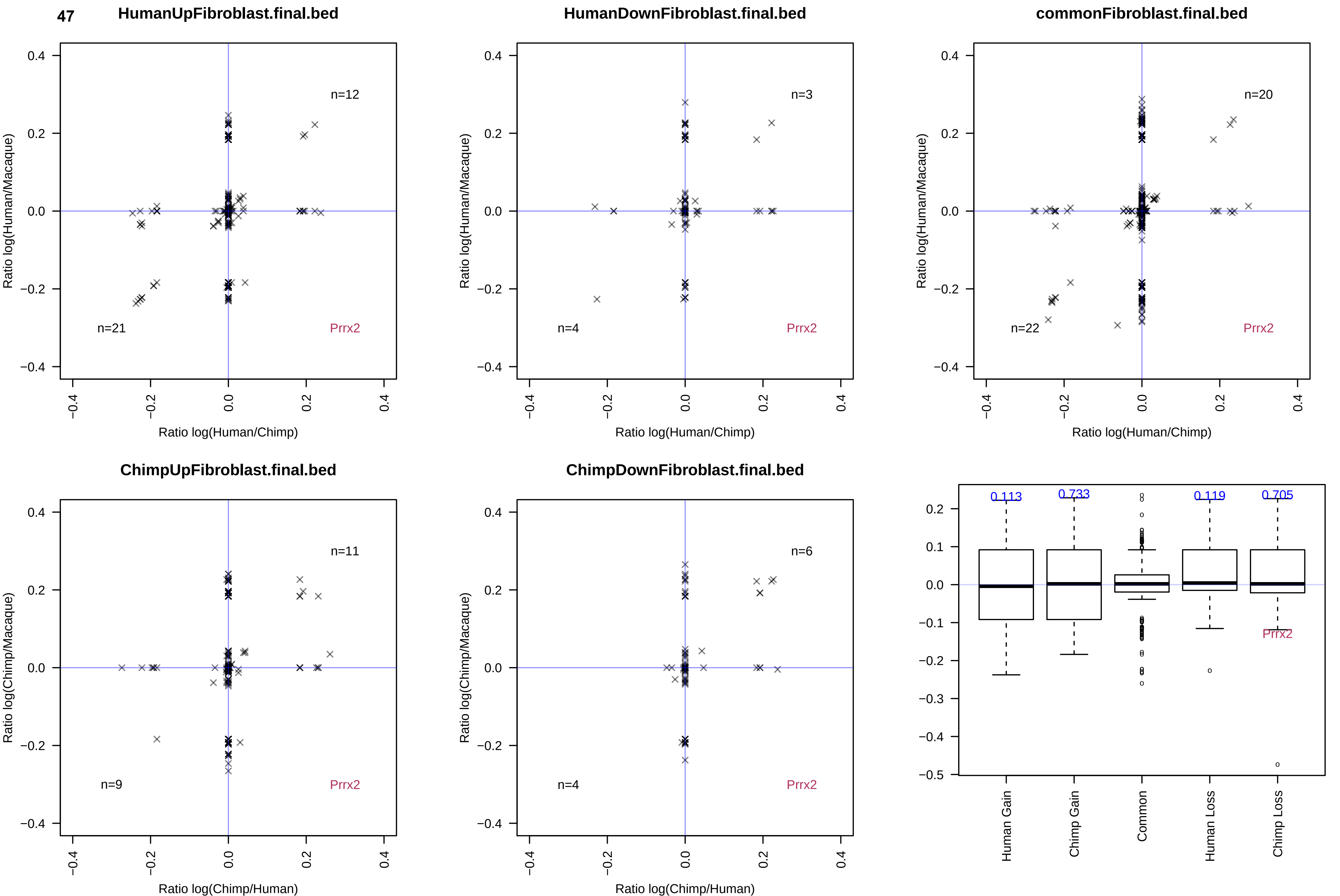
HumanUpFibroblast.final.bed
HumanDownFibroblast.final.bed
commonFibroblast.final.bed
ChimpUpFibroblast.final.bed
ChimpDownFibroblast.final.bed
Ratio log(Human/Macaque)
Ratio log(Human/Chimp)
Ratio log(Chimp/Macaque)
Ratio log(Chimp/Human)
n=12
n=21
n=3
n=4
n=20
n=22
n=11
n=9
n=6
n=4
Prrx2
0.113
0.733
0.119
0.705
Human Gain
Chimp Gain
Common
Human Loss
Chimp Loss

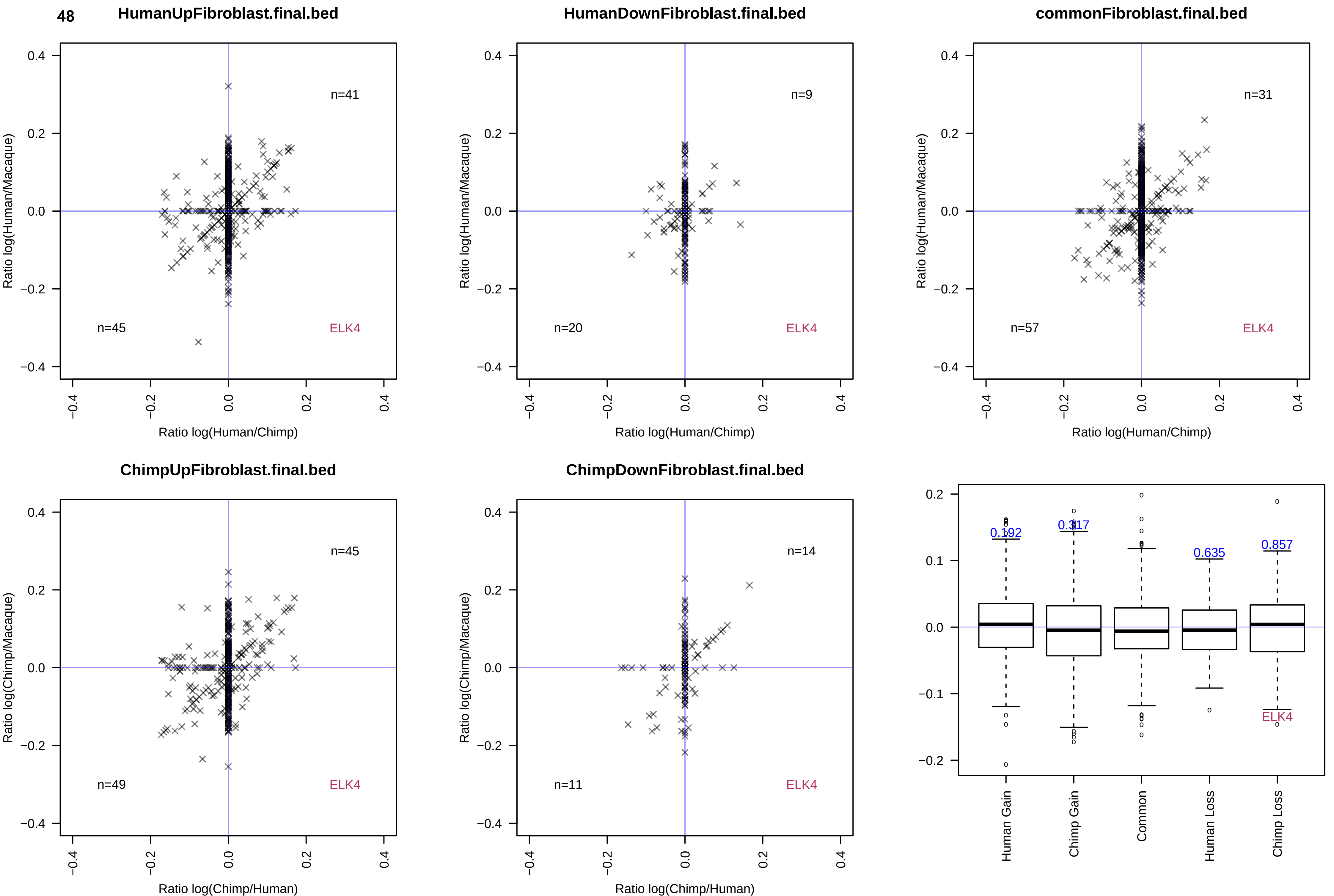
HumanUpFibroblast.final.bed
n=41
n=45
ELK4
Ratio log(Human/Macaque)
Ratio log(Human/Chimp)
HumanDownFibroblast.final.bed
n=9
n=20
ELK4
Ratio log(Human/Macaque)
Ratio log(Human/Chimp)
commonFibroblast.final.bed
n=31
n=57
ELK4
Ratio log(Human/Macaque)
Ratio log(Human/Chimp)
ChimpUpFibroblast.final.bed
n=45
n=49
ELK4
Ratio log(Chimp/Macaque)
Ratio log(Chimp/Human)
ChimpDownFibroblast.final.bed
n=14
n=11
ELK4
Ratio log(Chimp/Macaque)
Ratio log(Chimp/Human)
0.192
0.317
0.635
0.857
ELK4
Human Gain
Chimp Gain
Common
Human Loss
Chimp Loss

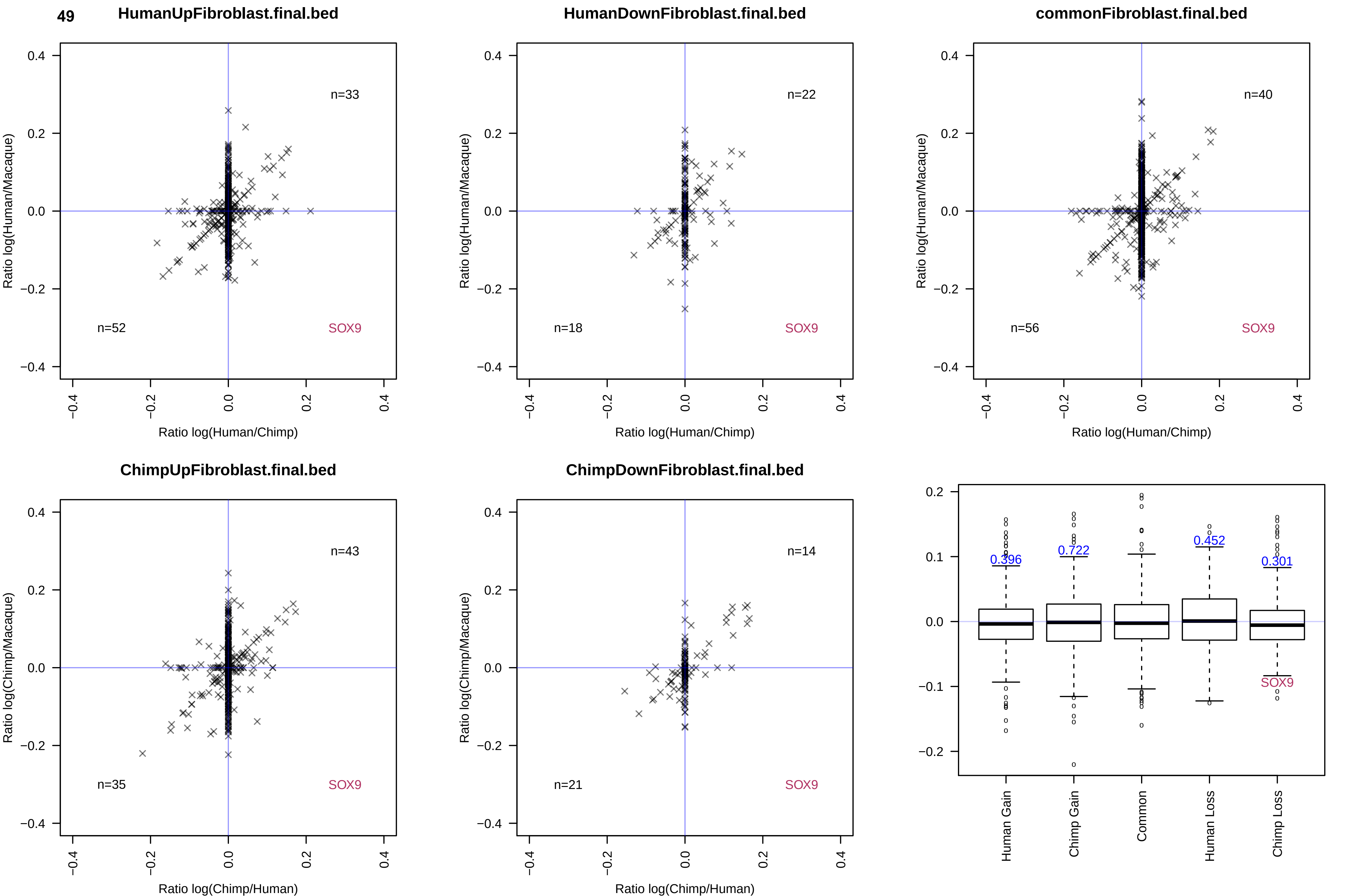
HumanUpFibroblast.final.bed
n=33
n=52
SOX9
Ratio log(Human/Macaque)
Ratio log(Human/Chimp)
HumanDownFibroblast.final.bed
n=22
n=18
SOX9
Ratio log(Human/Macaque)
Ratio log(Human/Chimp)
commonFibroblast.final.bed
n=40
n=56
SOX9
Ratio log(Human/Macaque)
Ratio log(Human/Chimp)
ChimpUpFibroblast.final.bed
n=43
n=35
SOX9
Ratio log(Chimp/Macaque)
Ratio log(Chimp/Human)
ChimpDownFibroblast.final.bed
n=14
n=21
SOX9
Ratio log(Chimp/Macaque)
Ratio log(Chimp/Human)
0.396
0.722
0.452
0.301
SOX9
Human Gain
Chimp Gain
Common
Human Loss
Chimp Loss

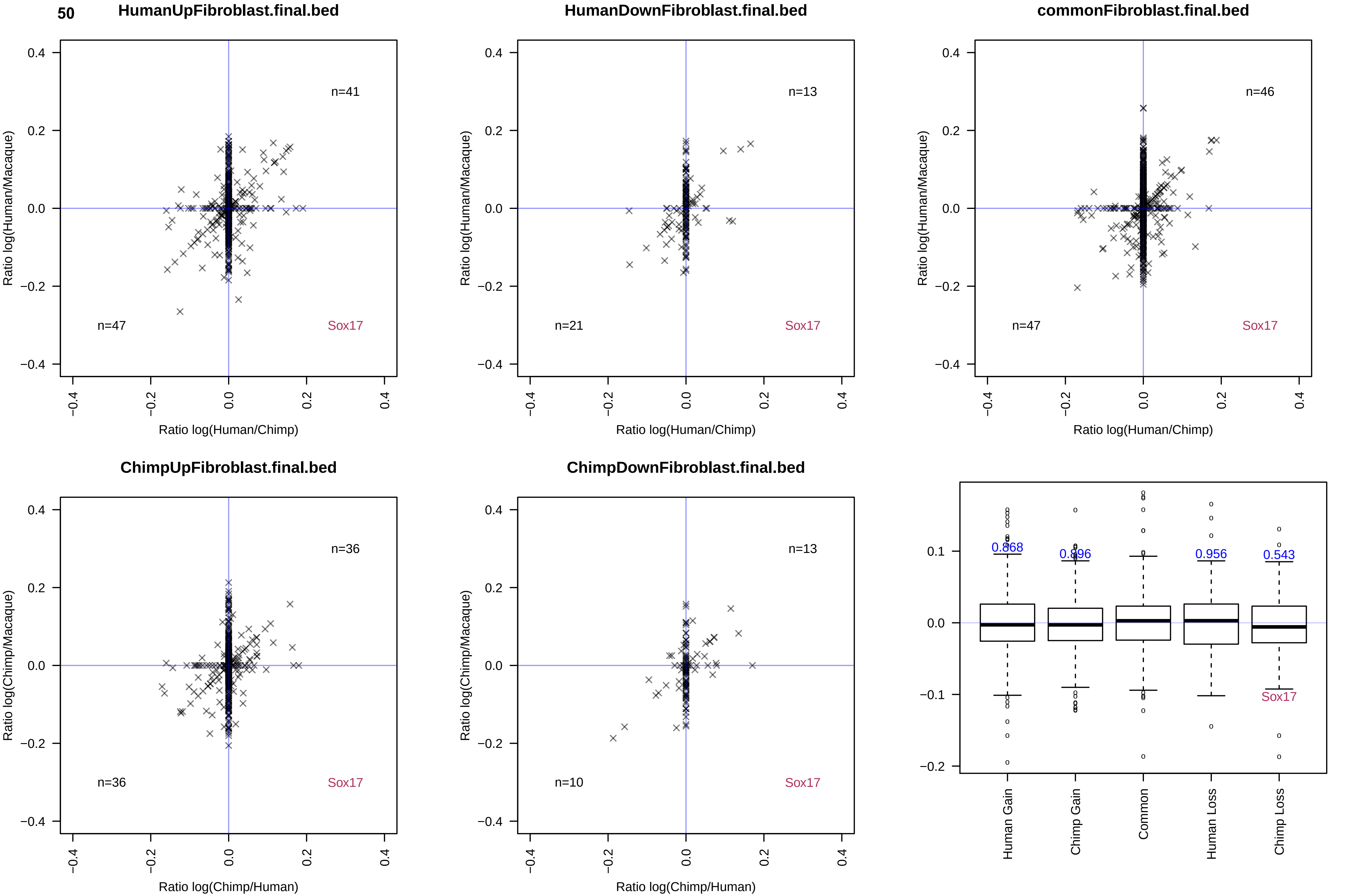
HumanUpFibroblast.final.bed
n=41
n=47
Sox17
Ratio log(Human/Macaque)
Ratio log(Human/Chimp)
HumanDownFibroblast.final.bed
n=13
n=21
Sox17
Ratio log(Human/Macaque)
Ratio log(Human/Chimp)
commonFibroblast.final.bed
n=46
n=47
Sox17
Ratio log(Human/Macaque)
Ratio log(Human/Chimp)
ChimpUpFibroblast.final.bed
n=36
n=36
Sox17
Ratio log(Chimp/Macaque)
Ratio log(Chimp/Human)
ChimpDownFibroblast.final.bed
n=13
n=10
Sox17
Ratio log(Chimp/Macaque)
Ratio log(Chimp/Human)
0.868
0.896
0.956
0.543
Sox17
Human Gain
Chimp Gain
Common
Human Loss
Chimp Loss

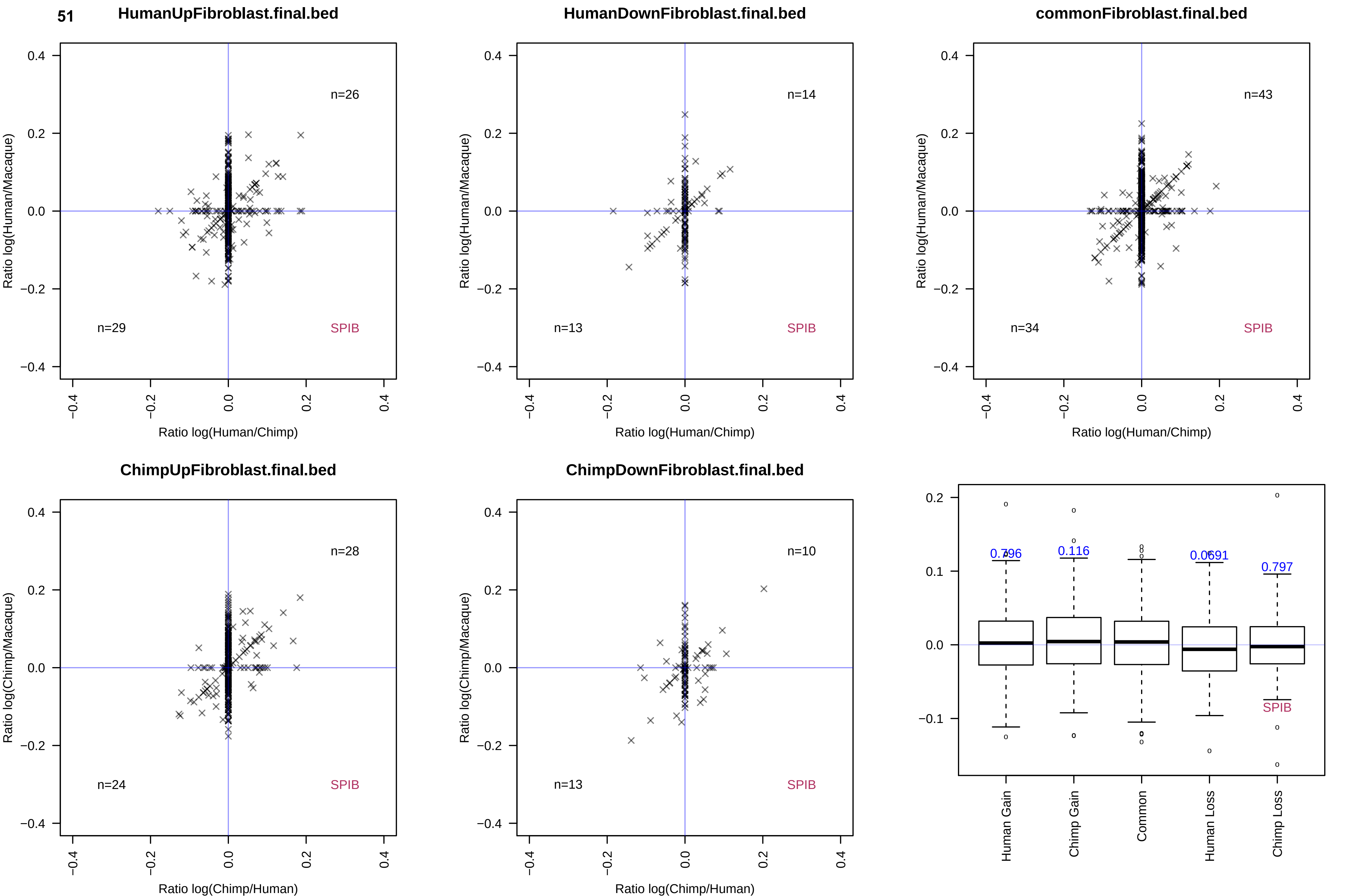
HumanUpFibroblast.final.bed
HumanDownFibroblast.final.bed
commonFibroblast.final.bed
ChimpUpFibroblast.final.bed
ChimpDownFibroblast.final.bed
Ratio log(Human/Macaque)
Ratio log(Human/Chimp)
Ratio log(Chimp/Macaque)
Ratio log(Chimp/Human)
n=26
n=29
n=14
n=13
n=43
n=34
n=28
n=24
n=10
n=13
SPIB
0.796
0.116
0.0691
0.797
Human Gain
Chimp Gain
Common
Human Loss
Chimp Loss

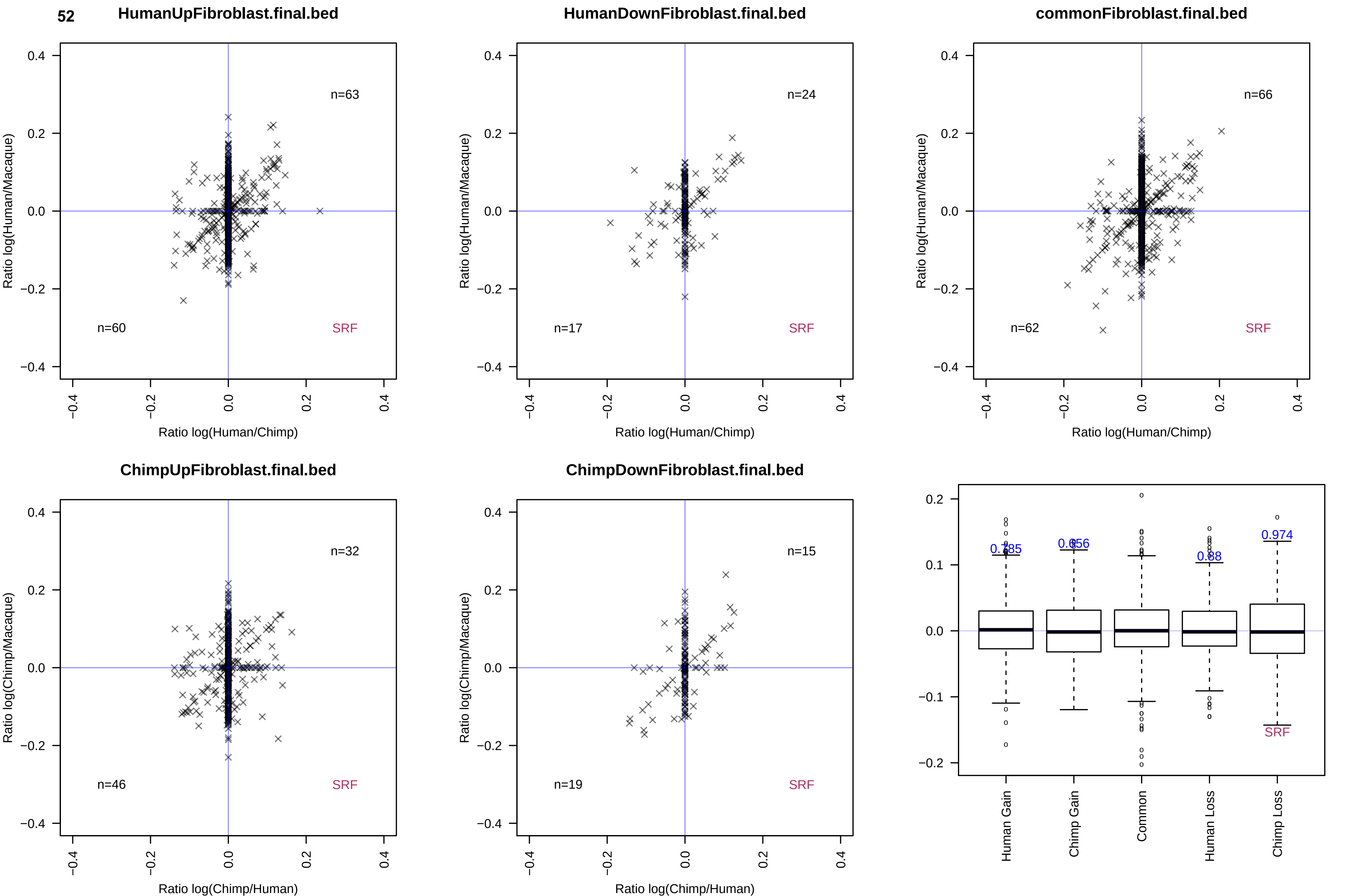

HumanUpFibroblast.final.bed
HumanDownFibroblast.final.bed
commonFibroblast.final.bed
ChimpUpFibroblast.final.bed
ChimpDownFibroblast.final.bed
n=63
n=60
n=24
n=17
n=66
n=62
n=32
n=46
n=15
n=19
SRF
Ratio log(Human/Macaque)
Ratio log(Human/Chimp)
Ratio log(Chimp/Macaque)
Ratio log(Chimp/Human)
0.785
0.656
0.88
0.974
Human Gain
Chimp Gain
Common
Human Loss
Chimp Loss

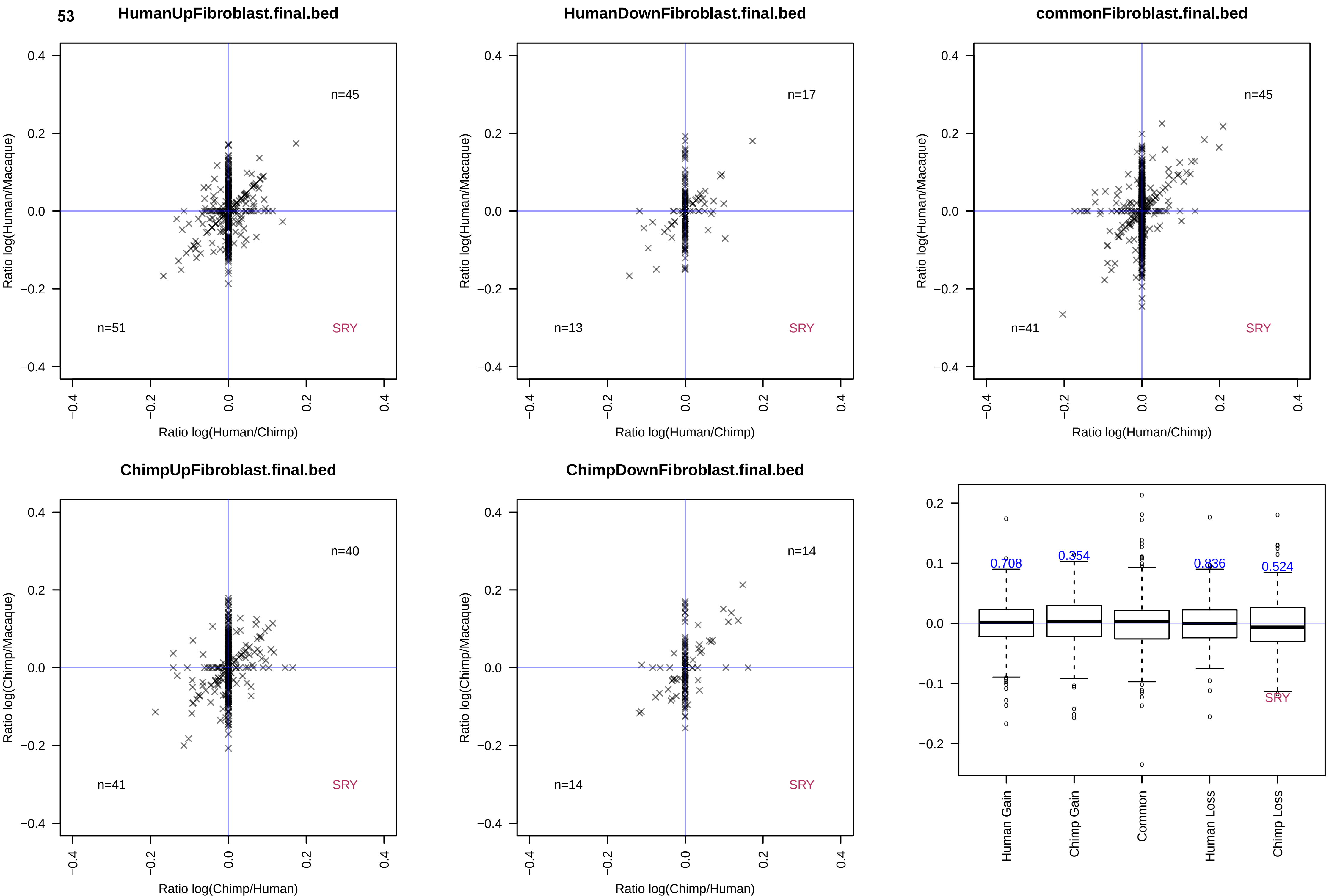
HumanUpFibroblast.final.bed
n=45
n=51
SRY
Ratio log(Human/Chimp)
Ratio log(Human/Macaque)
HumanDownFibroblast.final.bed
n=17
n=13
SRY
Ratio log(Human/Chimp)
Ratio log(Human/Macaque)
commonFibroblast.final.bed
n=45
n=41
SRY
Ratio log(Human/Chimp)
Ratio log(Human/Macaque)
ChimpUpFibroblast.final.bed
n=40
n=41
SRY
Ratio log(Chimp/Human)
Ratio log(Chimp/Macaque)
ChimpDownFibroblast.final.bed
n=14
n=14
SRY
Ratio log(Chimp/Human)
Ratio log(Chimp/Macaque)
0.708
0.354
0.836
0.524
SRY
Human Gain
Chimp Gain
Common
Human Loss
Chimp Loss

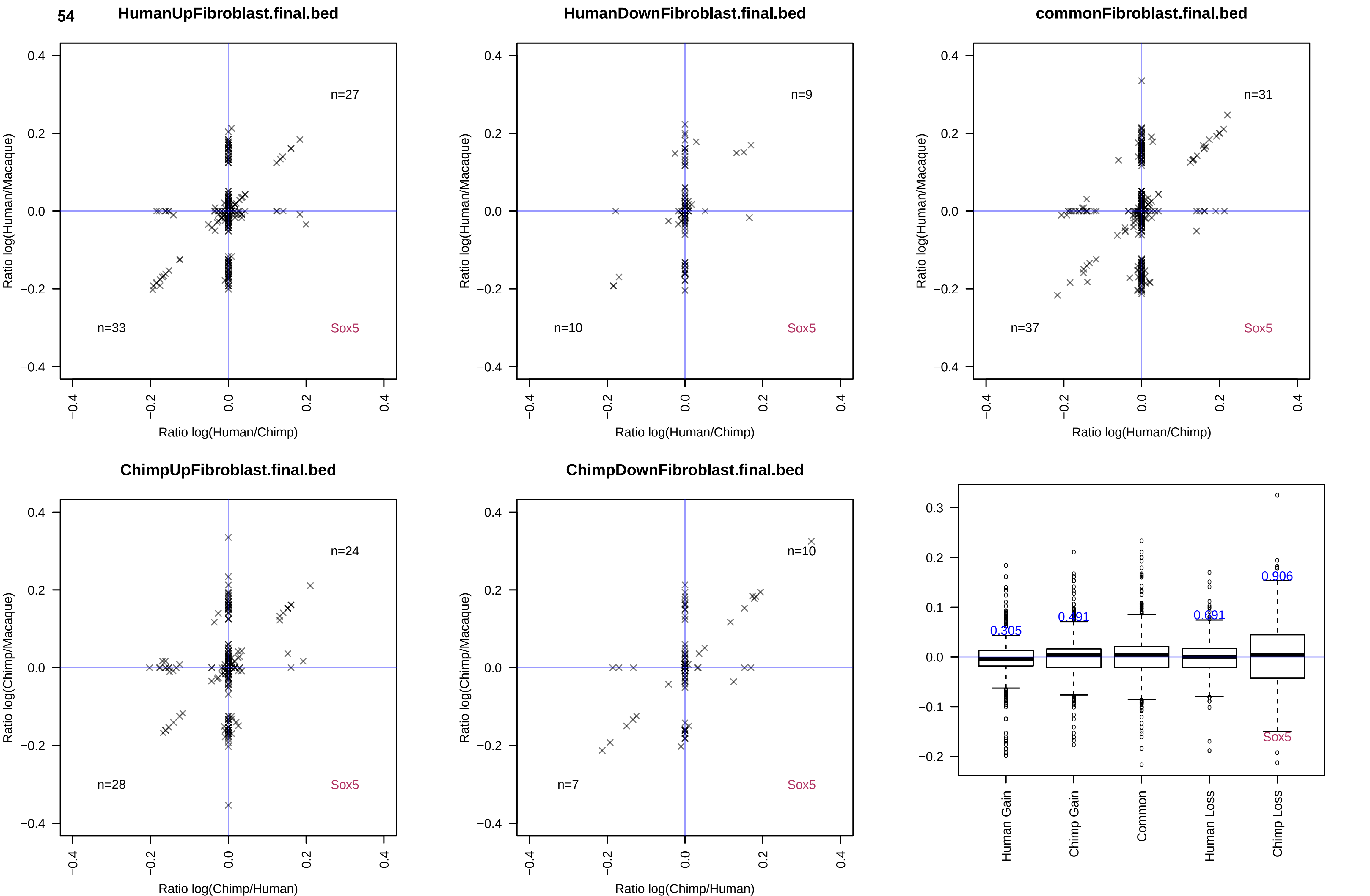
HumanUpFibroblast.final.bed
n=27
n=33
Sox5
HumanDownFibroblast.final.bed
n=9
n=10
Sox5
commonFibroblast.final.bed
n=31
n=37
Sox5
Ratio log(Human/Chimp)
Ratio log(Human/Macaque)
ChimpUpFibroblast.final.bed
n=24
n=28
Sox5
ChimpDownFibroblast.final.bed
n=10
n=7
Sox5
Ratio log(Chimp/Human)
Ratio log(Chimp/Macaque)
0.305
0.491
0.691
0.906
Sox5
Human Gain
Chimp Gain
Common
Human Loss
Chimp Loss

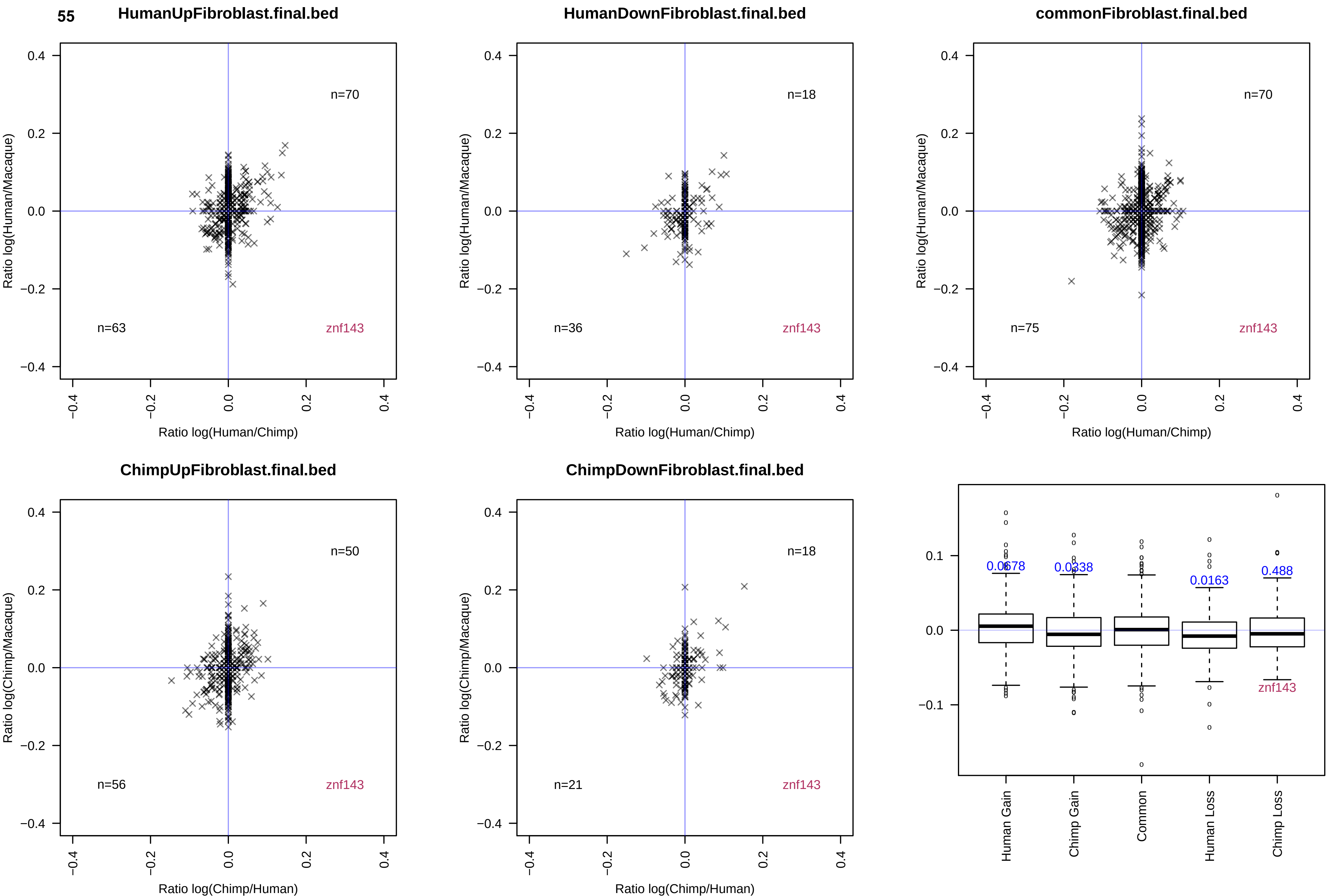
HumanUpFibroblast.final.bed
n=70
n=63
znf143
Ratio log(Human/Chimp)
Ratio log(Human/Macaque)
HumanDownFibroblast.final.bed
n=18
n=36
znf143
Ratio log(Human/Chimp)
Ratio log(Human/Macaque)
commonFibroblast.final.bed
n=70
n=75
znf143
Ratio log(Human/Chimp)
Ratio log(Human/Macaque)
ChimpUpFibroblast.final.bed
n=50
n=56
znf143
Ratio log(Chimp/Human)
Ratio log(Chimp/Macaque)
ChimpDownFibroblast.final.bed
n=18
n=21
znf143
Ratio log(Chimp/Human)
Ratio log(Chimp/Macaque)
0.0678
0.0338
0.0163
0.488
znf143
Human Gain
Chimp Gain
Common
Human Loss
Chimp Loss

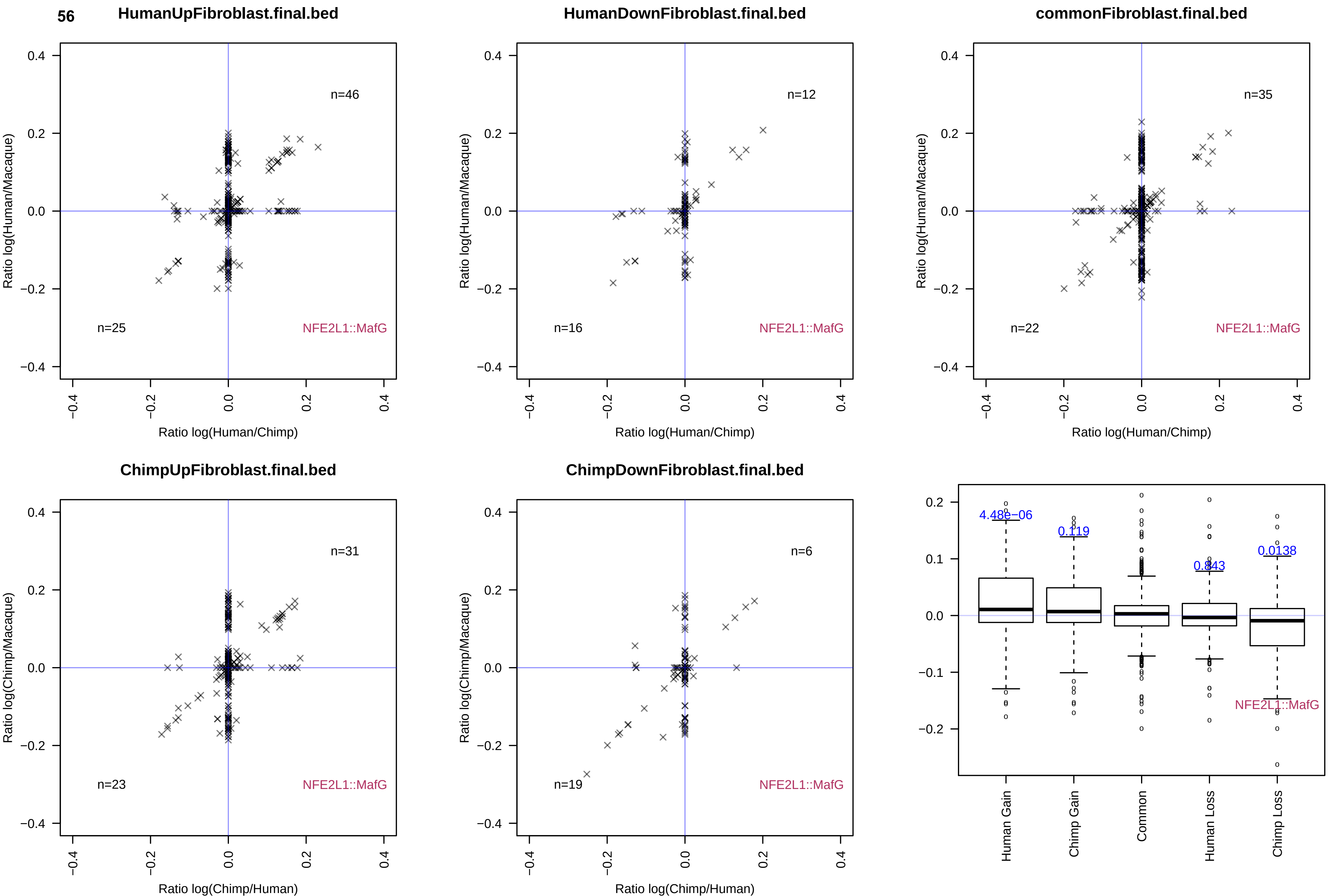
HumanUpFibroblast.final.bed
n=46
n=25
NFE2L1::MafG
Ratio log(Human/Chimp)
Ratio log(Human/Macaque)
HumanDownFibroblast.final.bed
n=12
n=16
NFE2L1::MafG
Ratio log(Human/Chimp)
Ratio log(Human/Macaque)
commonFibroblast.final.bed
n=35
n=22
NFE2L1::MafG
Ratio log(Human/Chimp)
Ratio log(Human/Macaque)
ChimpUpFibroblast.final.bed
n=31
n=23
NFE2L1::MafG
Ratio log(Chimp/Human)
Ratio log(Chimp/Macaque)
ChimpDownFibroblast.final.bed
n=6
n=19
NFE2L1::MafG
Ratio log(Chimp/Human)
Ratio log(Chimp/Macaque)
4.48e−06
0.119
0.843
0.0138
NFE2L1::MafG
Human Gain
Chimp Gain
Common
Human Loss
Chimp Loss

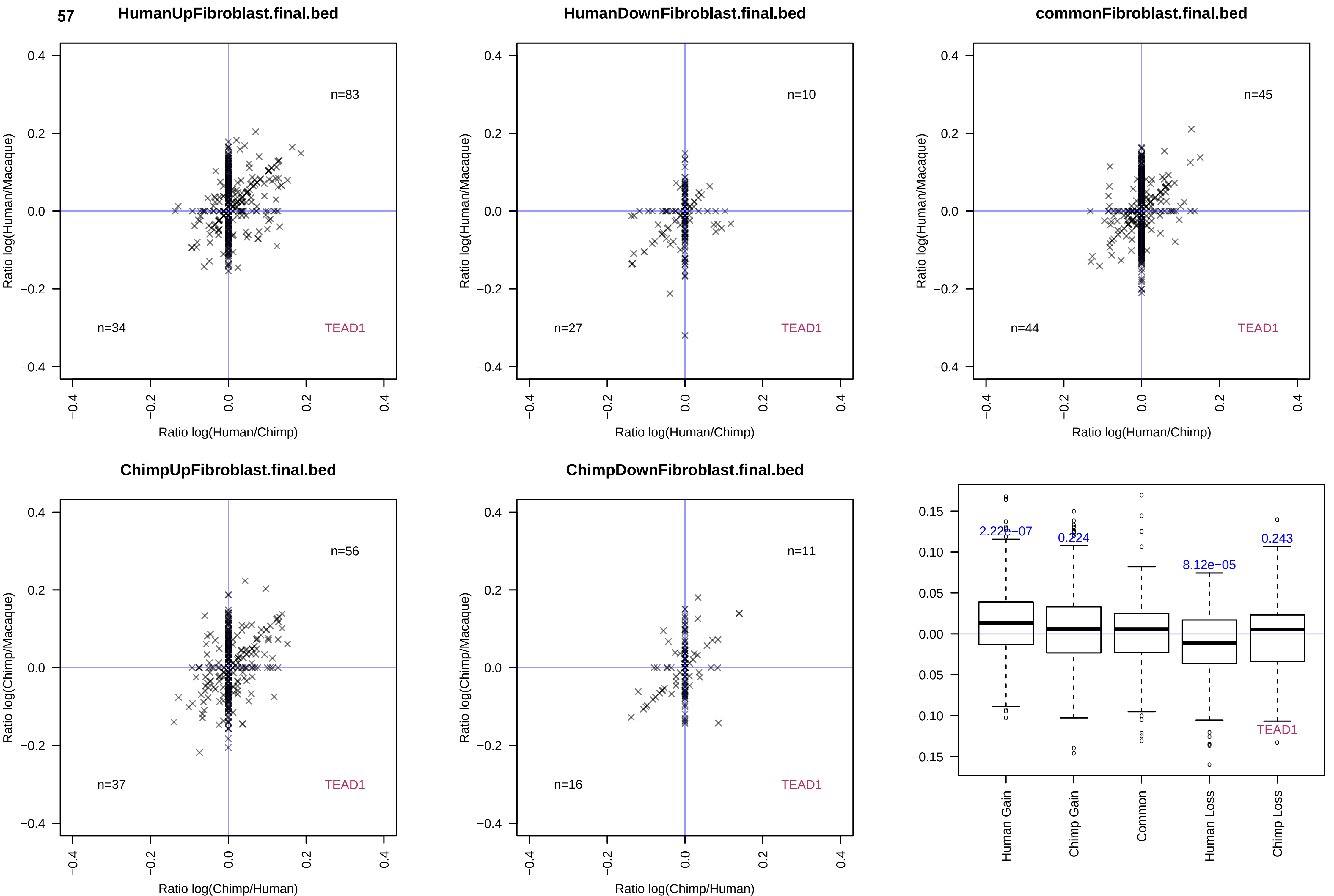
HumanUpFibroblast.final.bed
n=83
n=34
TEAD1
HumanDownFibroblast.final.bed
n=10
n=27
TEAD1
commonFibroblast.final.bed
n=45
n=44
TEAD1
Ratio log(Human/Macaque)
Ratio log(Human/Chimp)
ChimpUpFibroblast.final.bed
n=56
n=37
TEAD1
ChimpDownFibroblast.final.bed
n=11
n=16
TEAD1
Ratio log(Chimp/Macaque)
Ratio log(Chimp/Human)
2.22e−07
0.224
8.12e−05
0.243
TEAD1
Human Gain
Chimp Gain
Common
Human Loss
Chimp Loss

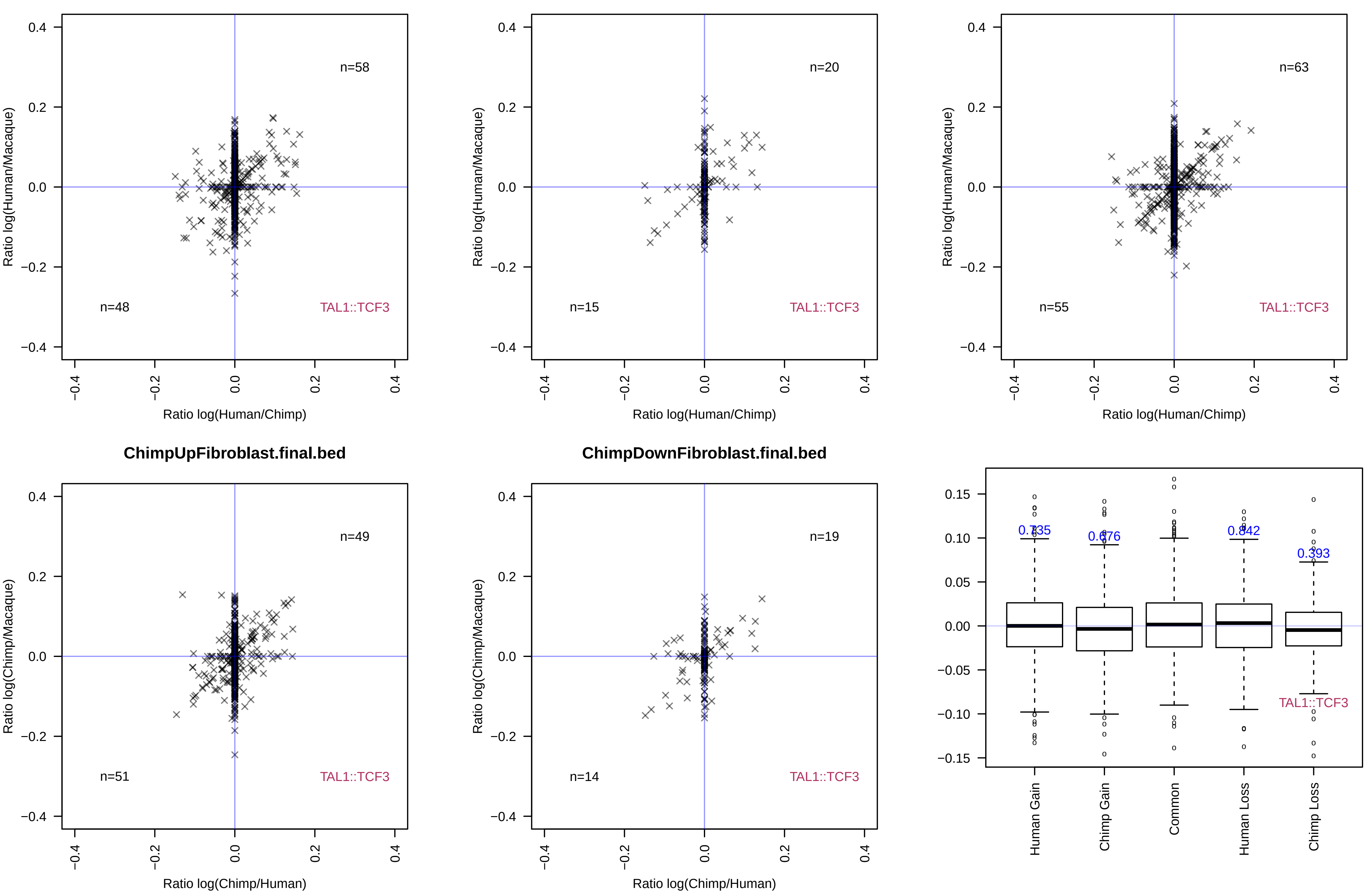

HumanUpFibroblast.final.bed
HumanDownFibroblast.final.bed
commonFibroblast.final.bed
ChimpUpFibroblast.final.bed
ChimpDownFibroblast.final.bed
Ratio log(Human/Macaque)
Ratio log(Human/Chimp)
Ratio log(Chimp/Macaque)
Ratio log(Chimp/Human)
n=58
n=48
n=20
n=15
n=63
n=55
n=49
n=51
n=19
n=14
TAL1::TCF3
0.735
0.676
0.842
0.393
Human Gain
Chimp Gain
Common
Human Loss
Chimp Loss

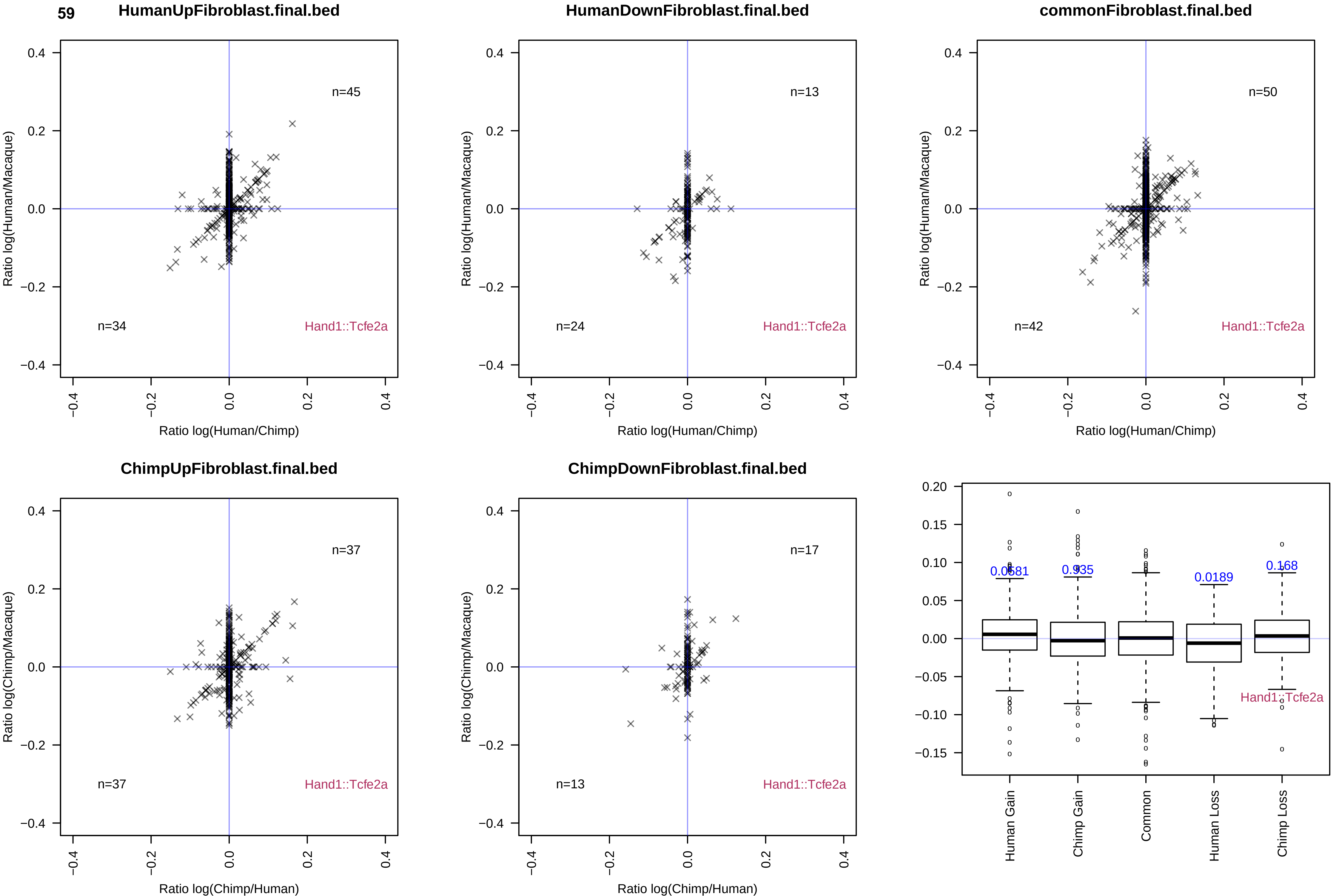
HumanUpFibroblast.final.bed
n=45
n=34
Hand1::Tcfe2a
Ratio log(Human/Macaque)
Ratio log(Human/Chimp)
HumanDownFibroblast.final.bed
n=13
n=24
Hand1::Tcfe2a
Ratio log(Human/Macaque)
Ratio log(Human/Chimp)
commonFibroblast.final.bed
n=50
n=42
Hand1::Tcfe2a
Ratio log(Human/Macaque)
Ratio log(Human/Chimp)
ChimpUpFibroblast.final.bed
n=37
n=37
Hand1::Tcfe2a
Ratio log(Chimp/Macaque)
Ratio log(Chimp/Human)
ChimpDownFibroblast.final.bed
n=17
n=13
Hand1::Tcfe2a
Ratio log(Chimp/Macaque)
Ratio log(Chimp/Human)
0.0581
0.935
0.0189
0.168
Hand1::Tcfe2a
Human Gain
Chimp Gain
Common
Human Loss
Chimp Loss

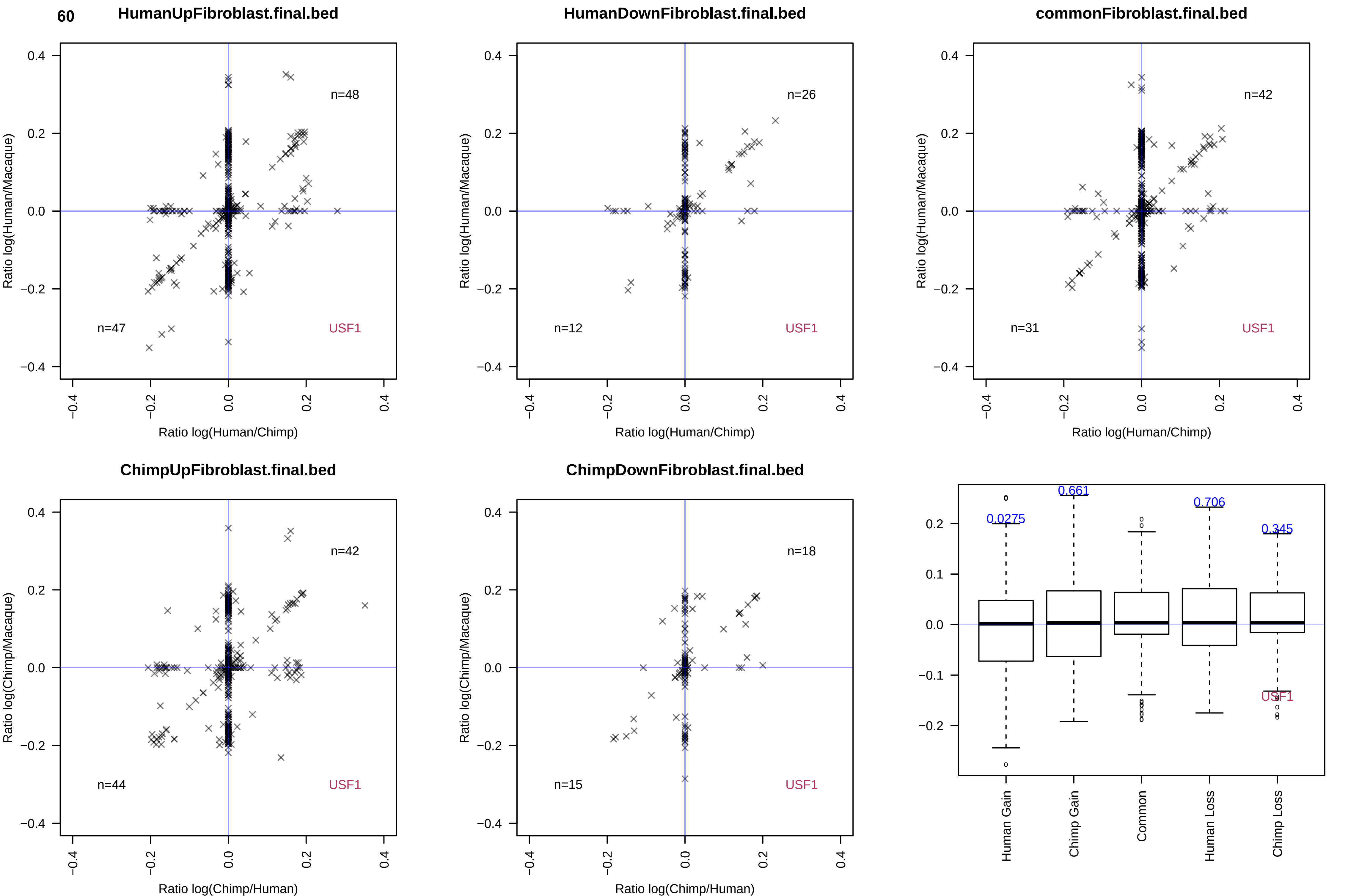
HumanUpFibroblast.final.bed
n=48
n=47
USF1
Ratio log(Human/Macaque)
Ratio log(Human/Chimp)
HumanDownFibroblast.final.bed
n=26
n=12
USF1
Ratio log(Human/Macaque)
Ratio log(Human/Chimp)
commonFibroblast.final.bed
n=42
n=31
USF1
Ratio log(Human/Macaque)
Ratio log(Human/Chimp)
ChimpUpFibroblast.final.bed
n=42
n=44
USF1
Ratio log(Chimp/Macaque)
Ratio log(Chimp/Human)
ChimpDownFibroblast.final.bed
n=18
n=15
USF1
Ratio log(Chimp/Macaque)
Ratio log(Chimp/Human)
0.0275
0.661
0.706
0.345
USF1
Human Gain
Chimp Gain
Common
Human Loss
Chimp Loss

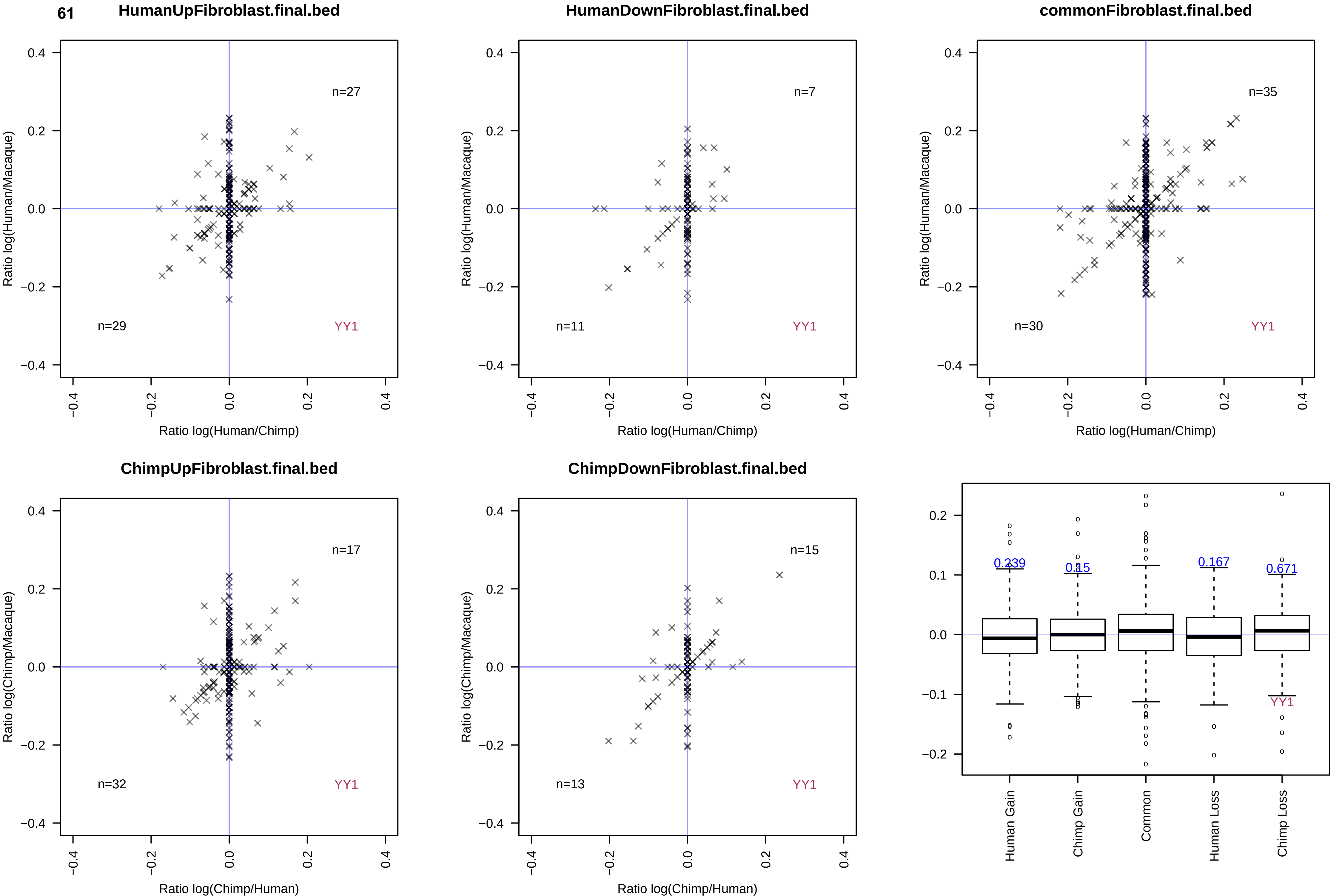
HumanUpFibroblast.final.bed
HumanDownFibroblast.final.bed
commonFibroblast.final.bed
ChimpUpFibroblast.final.bed
ChimpDownFibroblast.final.bed
n=27
n=29
n=7
n=11
n=35
n=30
n=17
n=32
n=15
n=13
YY1
Ratio log(Human/Macaque)
Ratio log(Human/Chimp)
Ratio log(Chimp/Macaque)
Ratio log(Chimp/Human)
0.239
0.15
0.167
0.671
Human Gain
Chimp Gain
Common
Human Loss
Chimp Loss

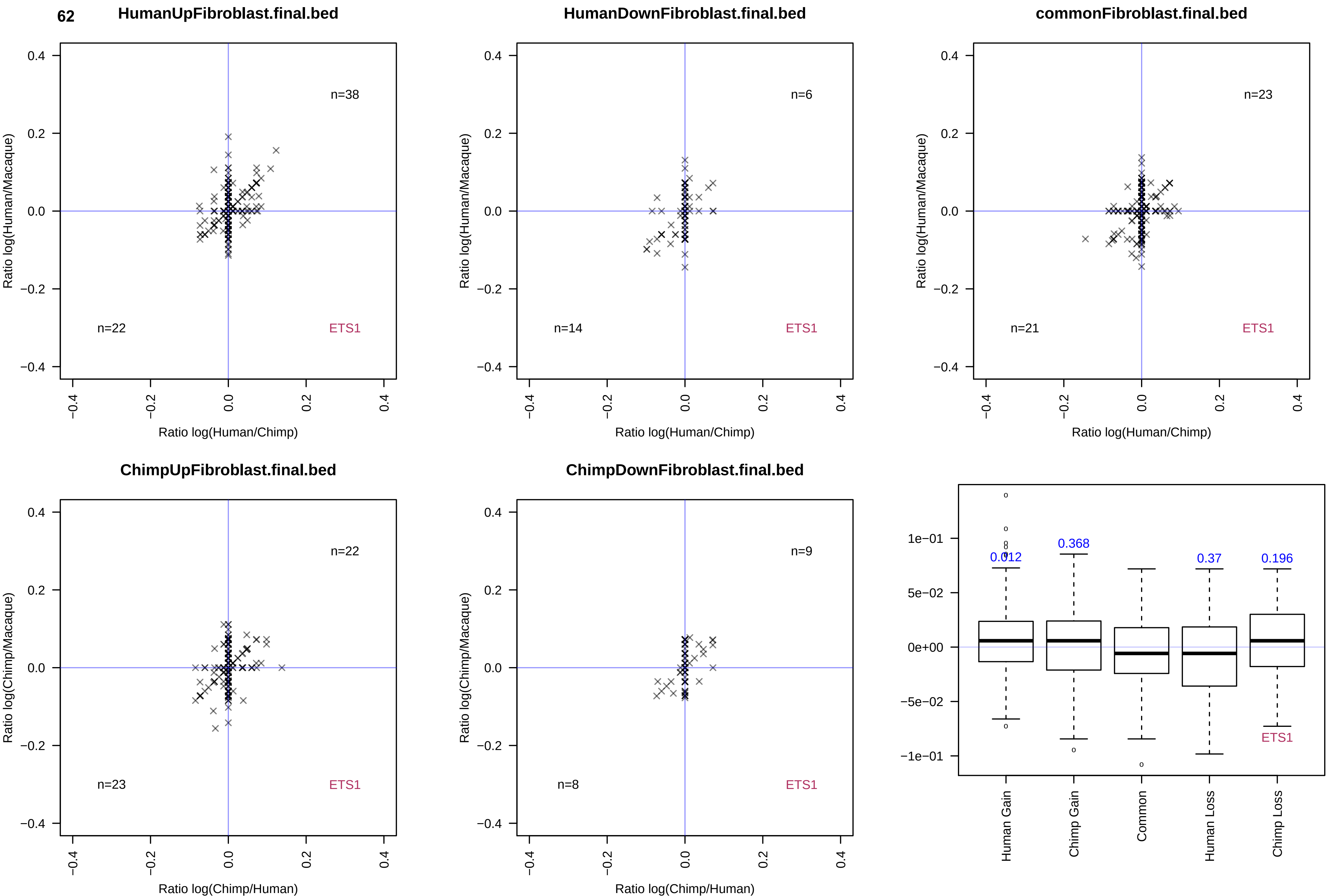

HumanUpFibroblast.final.bed
n=38
n=22
ETS1
HumanDownFibroblast.final.bed
n=6
n=14
ETS1
commonFibroblast.final.bed
n=23
n=21
ETS1
Ratio log(Human/Macaque)
Ratio log(Human/Chimp)
ChimpUpFibroblast.final.bed
n=22
n=23
ETS1
ChimpDownFibroblast.final.bed
n=9
n=8
ETS1
Ratio log(Chimp/Macaque)
Ratio log(Chimp/Human)
0.4
0.2
0.0
−0.2
−0.4
0.012
0.368
0.37
0.196
ETS1
1e−01
5e−02
0e+00
−5e−02
−1e−01
Human Gain
Chimp Gain
Common
Human Loss
Chimp Loss

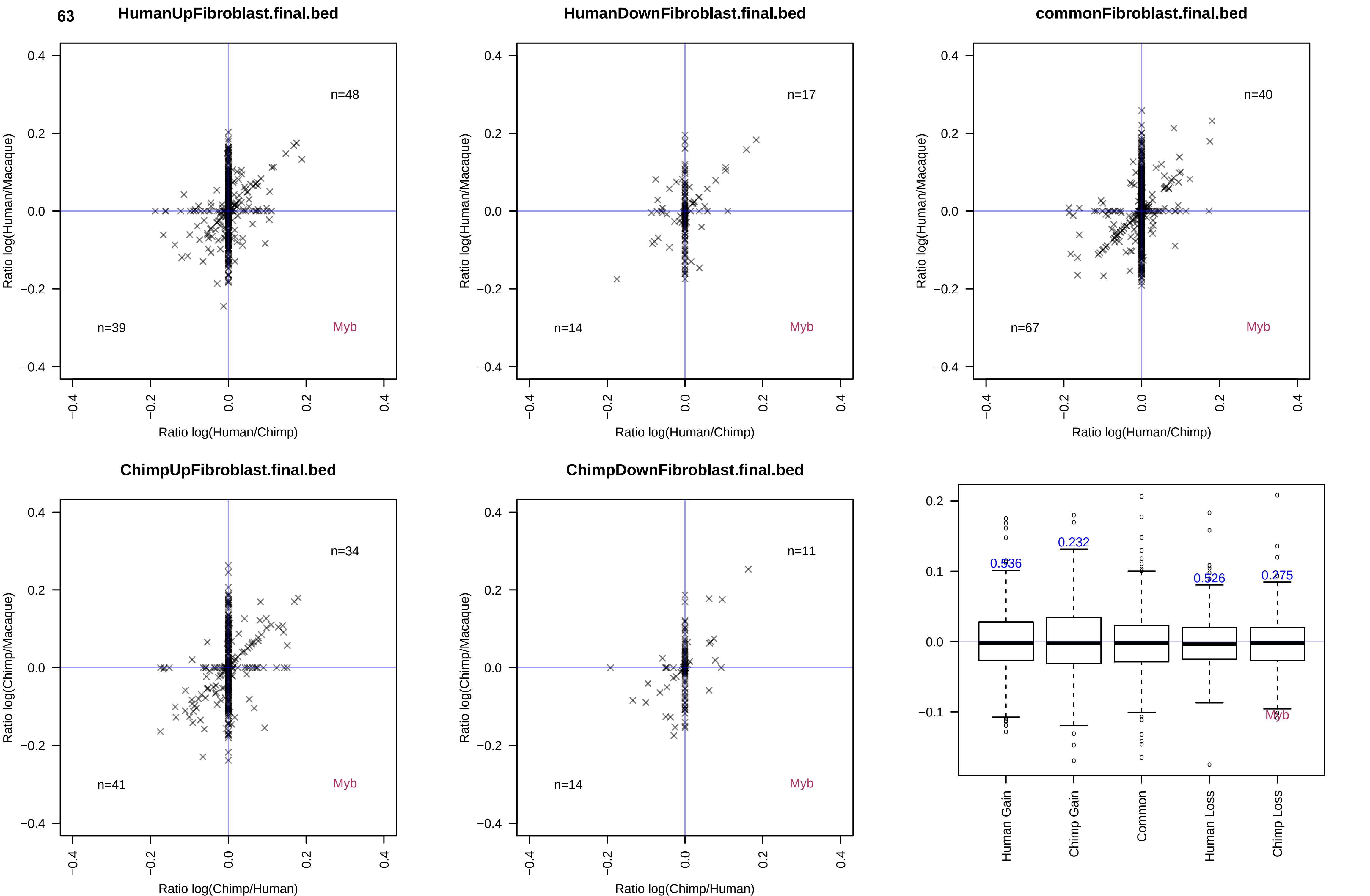
HumanUpFibroblast.final.bed
HumanDownFibroblast.final.bed
commonFibroblast.final.bed
ChimpUpFibroblast.final.bed
ChimpDownFibroblast.final.bed
n=48
n=39
n=17
n=14
n=40
n=67
n=34
n=41
n=11
n=14
Myb
Ratio log(Human/Macaque)
Ratio log(Human/Chimp)
Ratio log(Chimp/Macaque)
Ratio log(Chimp/Human)
0.536
0.232
0.526
0.275
Human Gain
Chimp Gain
Common
Human Loss
Chimp Loss

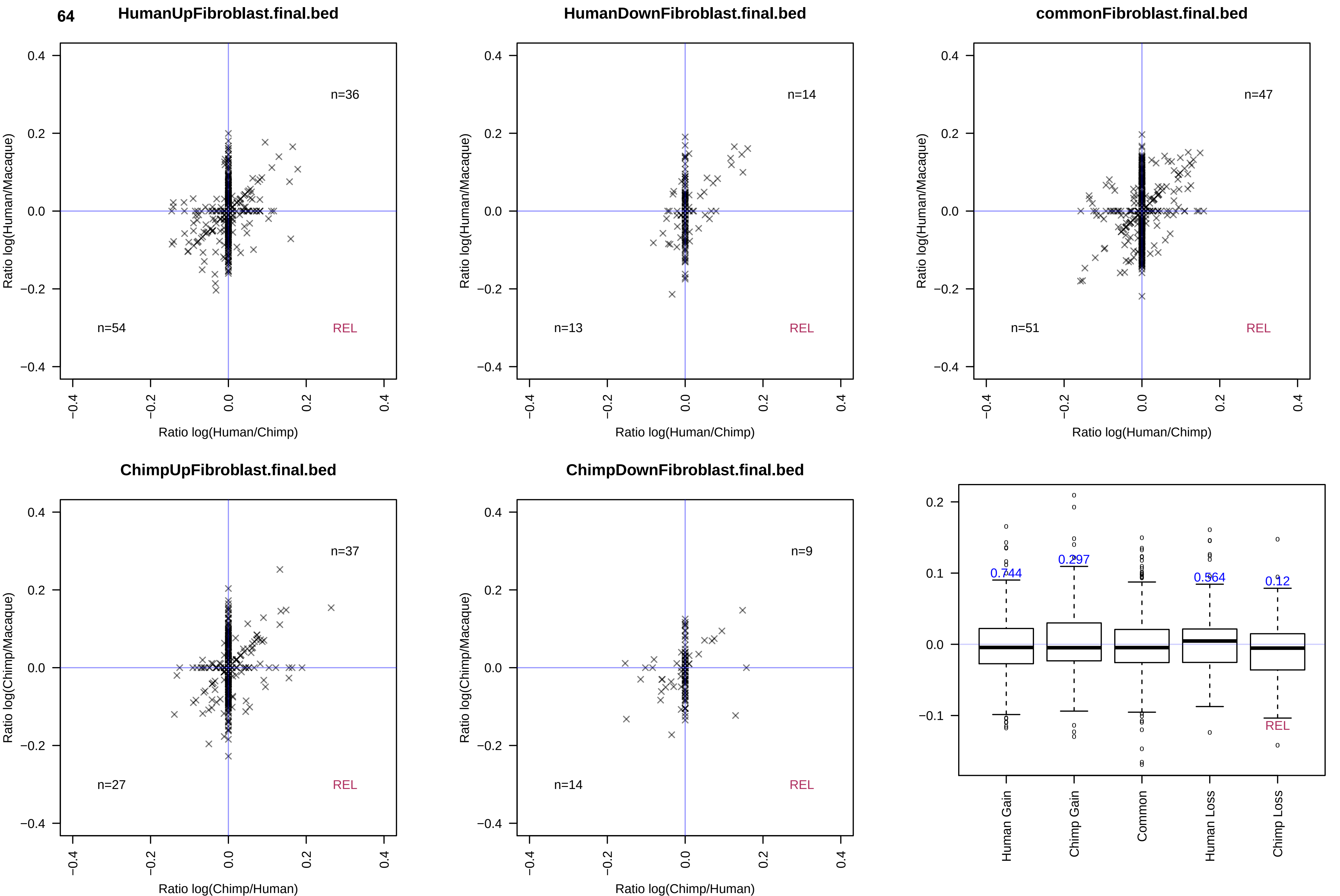
HumanUpFibroblast.final.bed
n=36
n=54
REL
Ratio log(Human/Macaque)
Ratio log(Human/Chimp)
HumanDownFibroblast.final.bed
n=14
n=13
REL
Ratio log(Human/Macaque)
Ratio log(Human/Chimp)
commonFibroblast.final.bed
n=47
n=51
REL
Ratio log(Human/Macaque)
Ratio log(Human/Chimp)
ChimpUpFibroblast.final.bed
n=37
n=27
REL
Ratio log(Chimp/Macaque)
Ratio log(Chimp/Human)
ChimpDownFibroblast.final.bed
n=9
n=14
REL
Ratio log(Chimp/Macaque)
Ratio log(Chimp/Human)
0.744
0.297
0.564
0.12
REL
Human Gain
Chimp Gain
Common
Human Loss
Chimp Loss

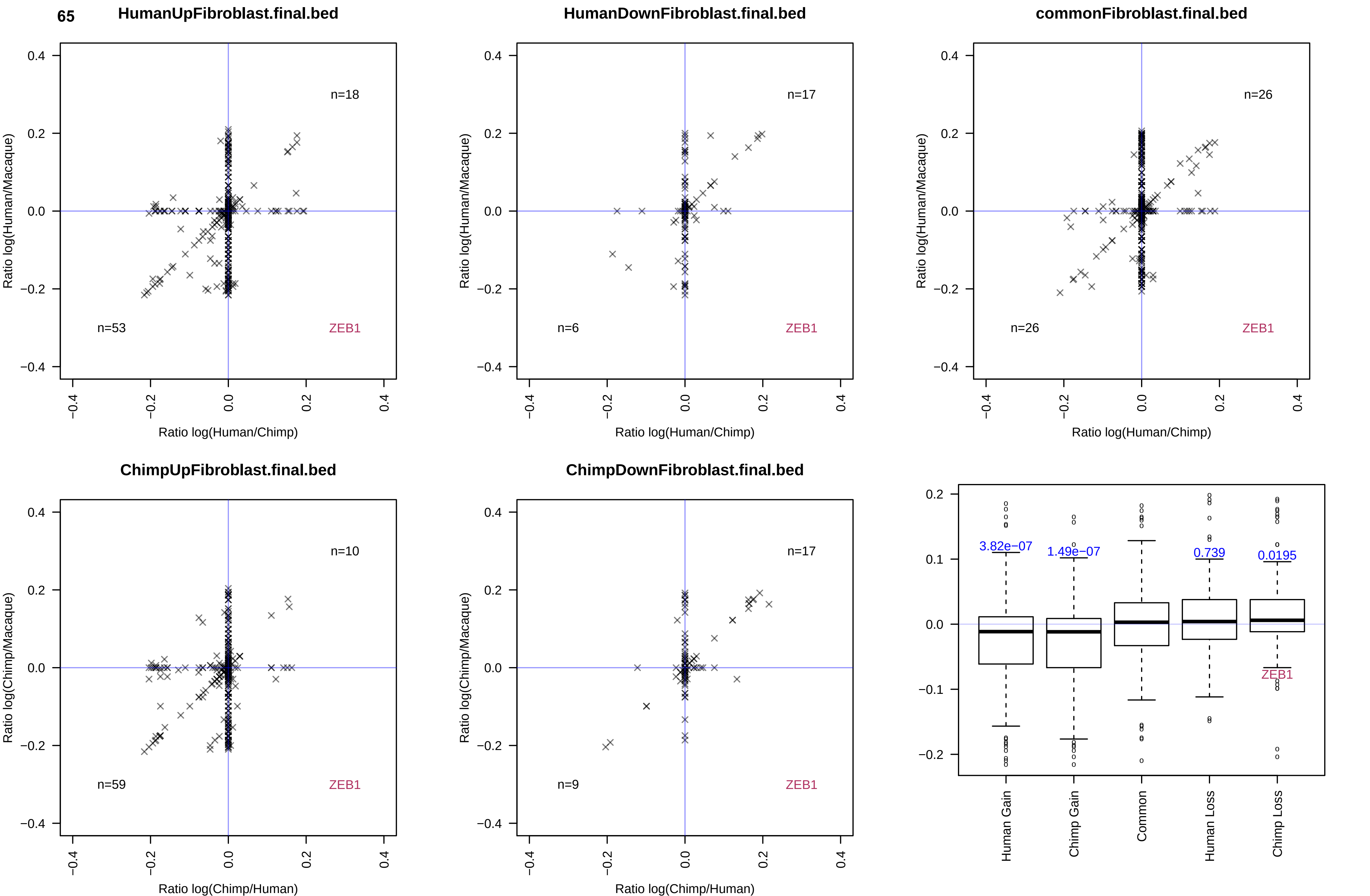
HumanUpFibroblast.final.bed
n=18
n=53
ZEB1
Ratio log(Human/Macaque)
Ratio log(Human/Chimp)
HumanDownFibroblast.final.bed
n=17
n=6
ZEB1
Ratio log(Human/Macaque)
Ratio log(Human/Chimp)
commonFibroblast.final.bed
n=26
n=26
ZEB1
Ratio log(Human/Macaque)
Ratio log(Human/Chimp)
ChimpUpFibroblast.final.bed
n=10
n=59
ZEB1
Ratio log(Chimp/Macaque)
Ratio log(Chimp/Human)
ChimpDownFibroblast.final.bed
n=17
n=9
ZEB1
Ratio log(Chimp/Macaque)
Ratio log(Chimp/Human)
3.82e−07
1.49e−07
0.739
0.0195
ZEB1
Human Gain
Chimp Gain
Common
Human Loss
Chimp Loss

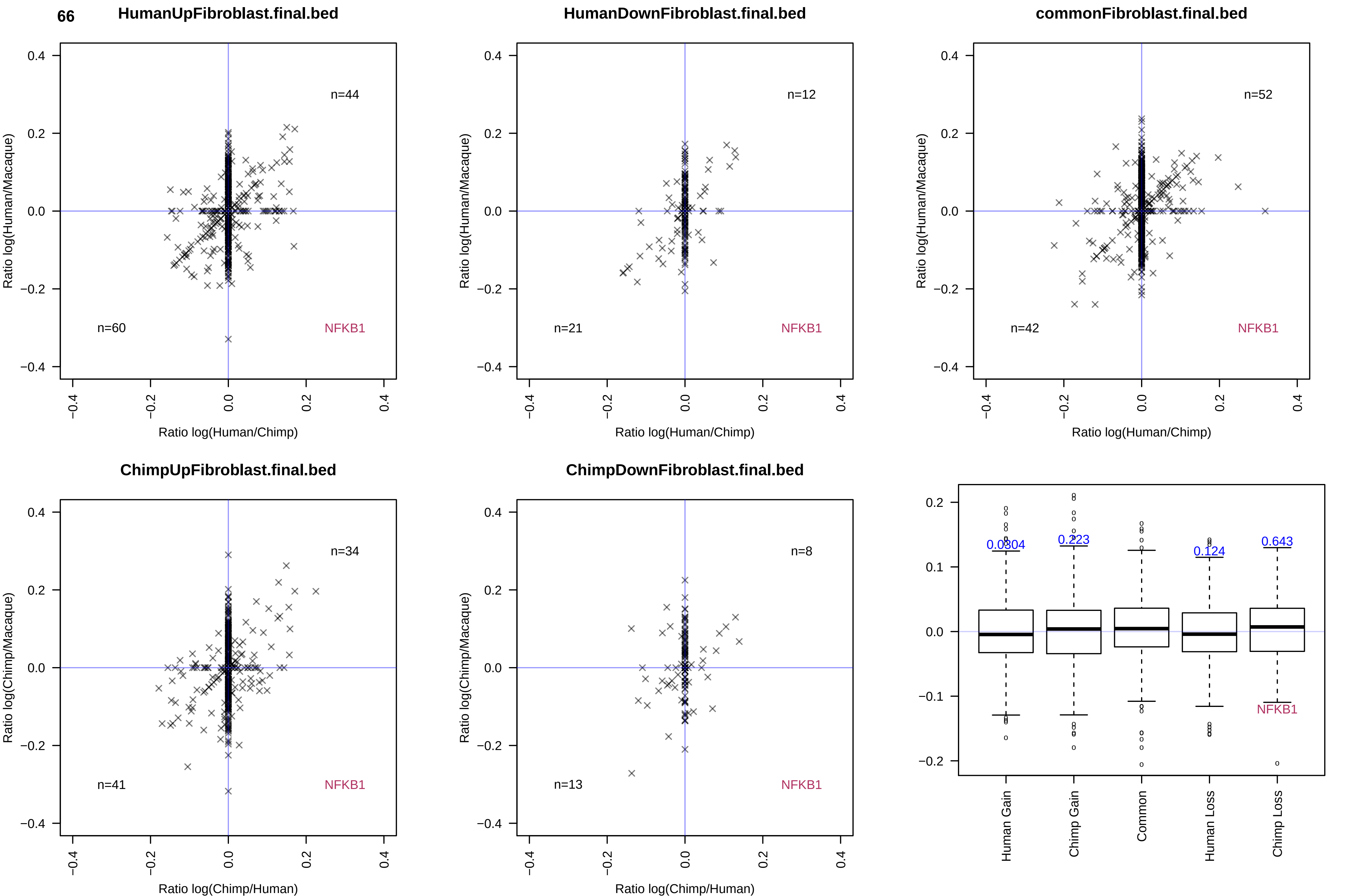
HumanUpFibroblast.final.bed
n=44
n=60
NFKB1
Ratio log(Human/Macaque)
Ratio log(Human/Chimp)
HumanDownFibroblast.final.bed
n=12
n=21
NFKB1
Ratio log(Human/Macaque)
Ratio log(Human/Chimp)
commonFibroblast.final.bed
n=52
n=42
NFKB1
Ratio log(Human/Macaque)
Ratio log(Human/Chimp)
ChimpUpFibroblast.final.bed
n=34
n=41
NFKB1
Ratio log(Chimp/Macaque)
Ratio log(Chimp/Human)
ChimpDownFibroblast.final.bed
n=8
n=13
NFKB1
Ratio log(Chimp/Macaque)
Ratio log(Chimp/Human)
0.0304
0.223
0.124
0.643
NFKB1
Human Gain
Chimp Gain
Common
Human Loss
Chimp Loss

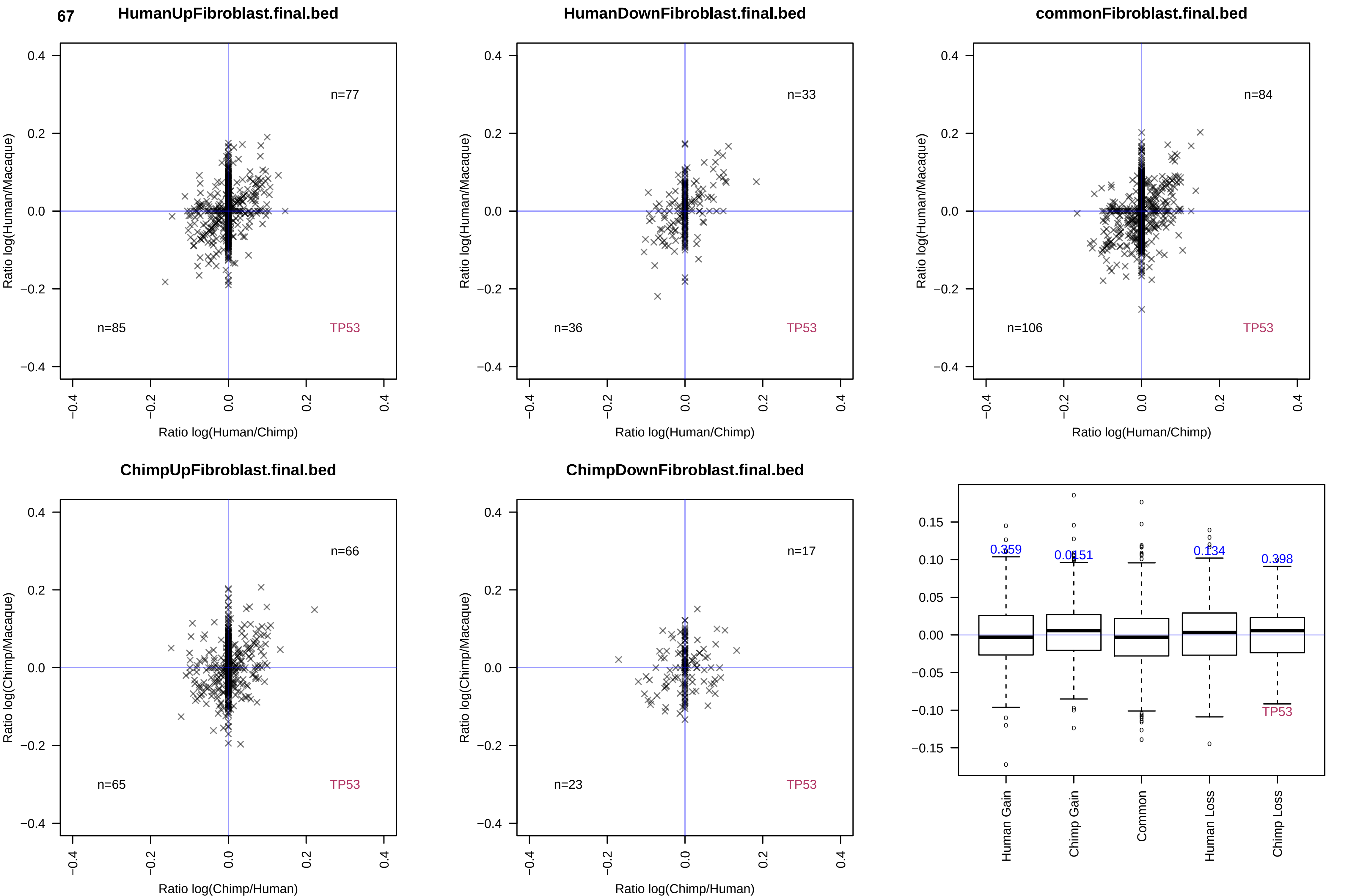
HumanUpFibroblast.final.bed
n=77
n=85
TP53
Ratio log(Human/Chimp)
Ratio log(Human/Macaque)
HumanDownFibroblast.final.bed
n=33
n=36
TP53
Ratio log(Human/Chimp)
Ratio log(Human/Macaque)
commonFibroblast.final.bed
n=84
n=106
TP53
Ratio log(Human/Chimp)
Ratio log(Human/Macaque)
ChimpUpFibroblast.final.bed
n=66
n=65
TP53
Ratio log(Chimp/Human)
Ratio log(Chimp/Macaque)
ChimpDownFibroblast.final.bed
n=17
n=23
TP53
Ratio log(Chimp/Human)
Ratio log(Chimp/Macaque)
0.359
0.0151
0.134
0.398
TP53
Human Gain
Chimp Gain
Common
Human Loss
Chimp Loss

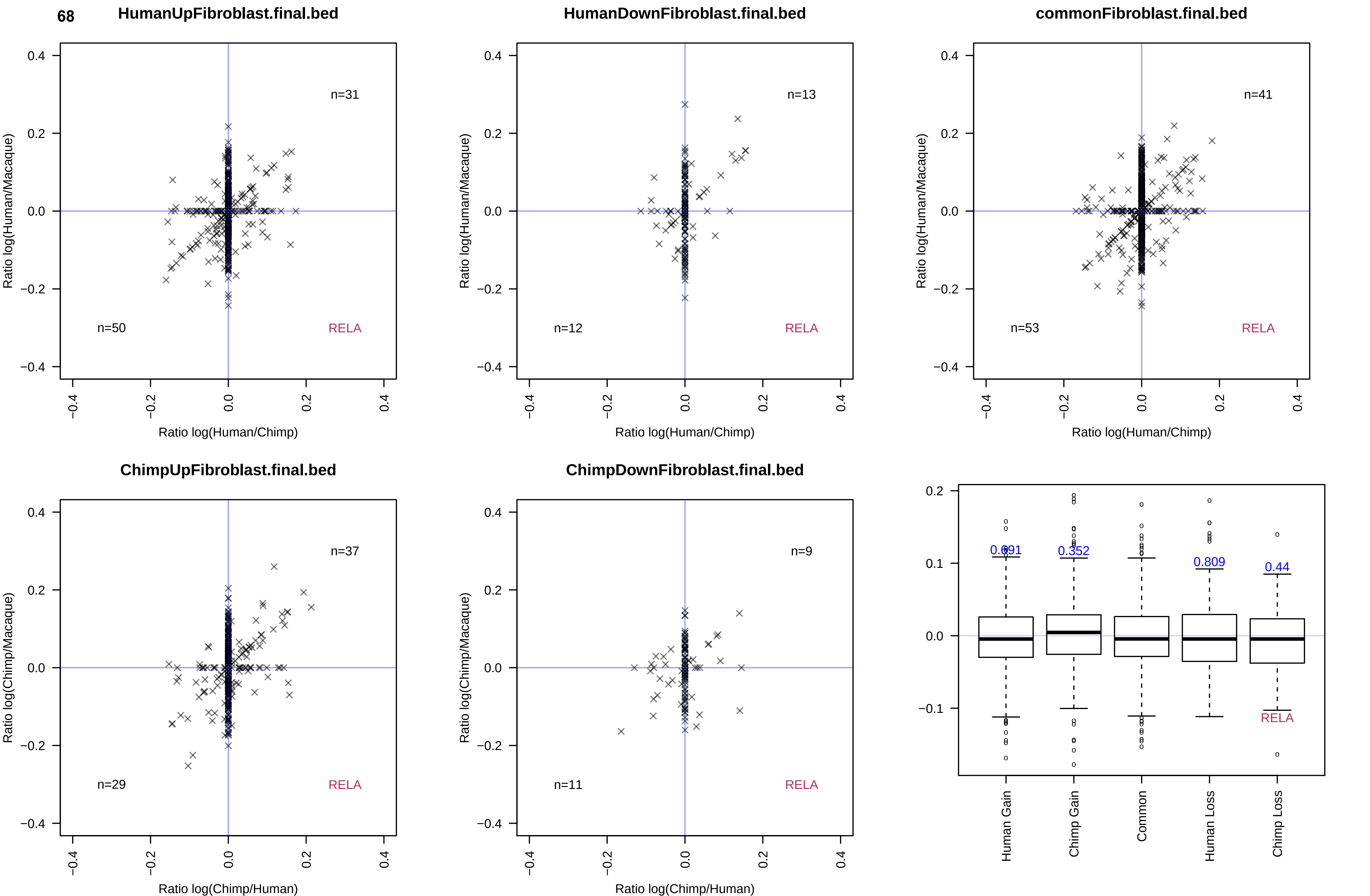
HumanUpFibroblast.final.bed
HumanDownFibroblast.final.bed
commonFibroblast.final.bed
ChimpUpFibroblast.final.bed
ChimpDownFibroblast.final.bed
Ratio log(Human/Macaque)
Ratio log(Human/Chimp)
Ratio log(Chimp/Macaque)
Ratio log(Chimp/Human)
n=31
n=50
n=13
n=12
n=41
n=53
n=37
n=29
n=9
n=11
RELA
0.691
0.352
0.809
0.44
Human Gain
Chimp Gain
Common
Human Loss
Chimp Loss

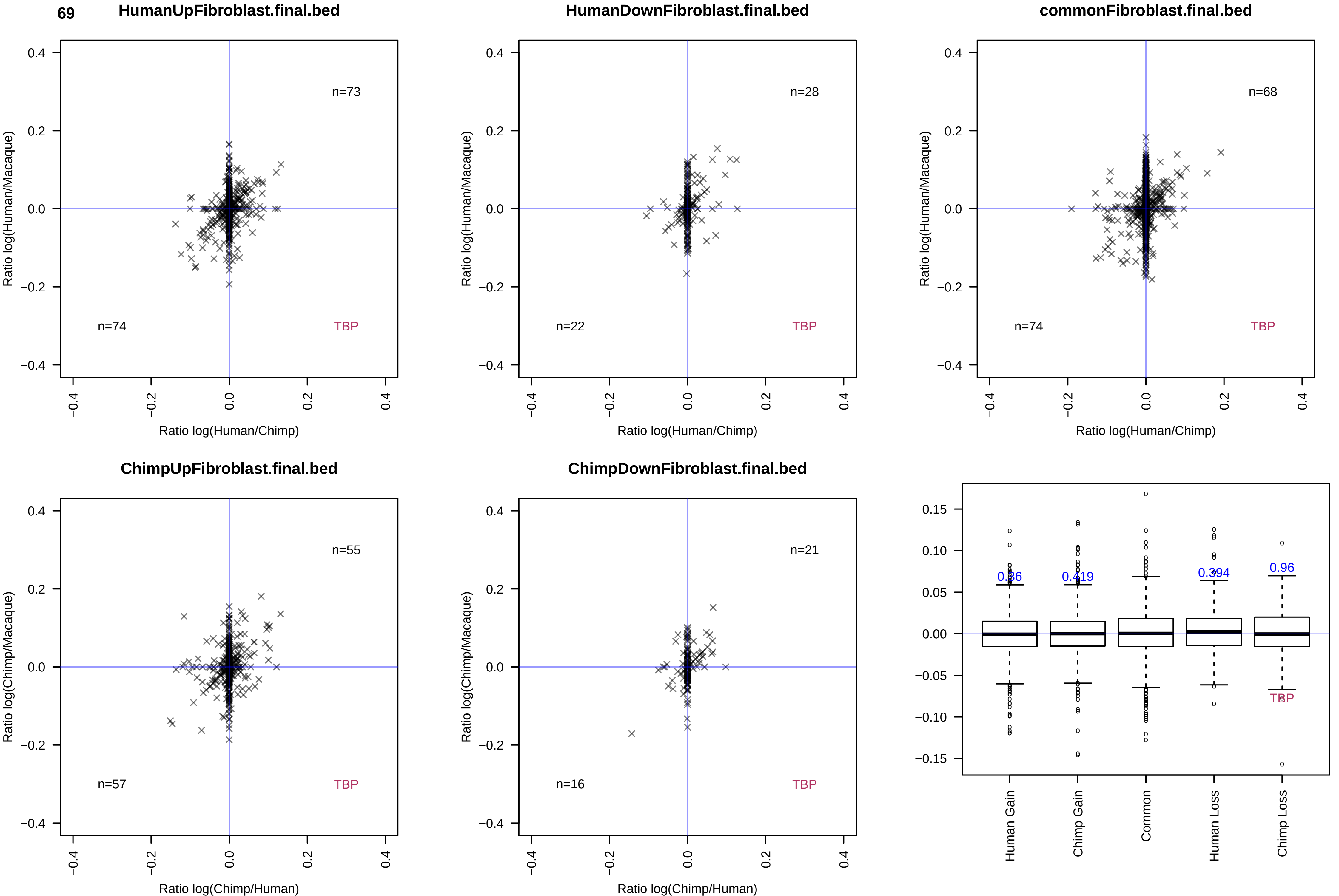
HumanUpFibroblast.final.bed
Ratio log(Human/Macaque)
Ratio log(Human/Chimp)
n=73
n=74
TBP
HumanDownFibroblast.final.bed
Ratio log(Human/Macaque)
Ratio log(Human/Chimp)
n=28
n=22
TBP
commonFibroblast.final.bed
Ratio log(Human/Macaque)
Ratio log(Human/Chimp)
n=68
n=74
TBP
ChimpUpFibroblast.final.bed
Ratio log(Chimp/Macaque)
Ratio log(Chimp/Human)
n=55
n=57
TBP
ChimpDownFibroblast.final.bed
Ratio log(Chimp/Macaque)
Ratio log(Chimp/Human)
n=21
n=16
TBP
0.36
0.419
0.394
0.96
TBP
Human Gain
Chimp Gain
Common
Human Loss
Chimp Loss

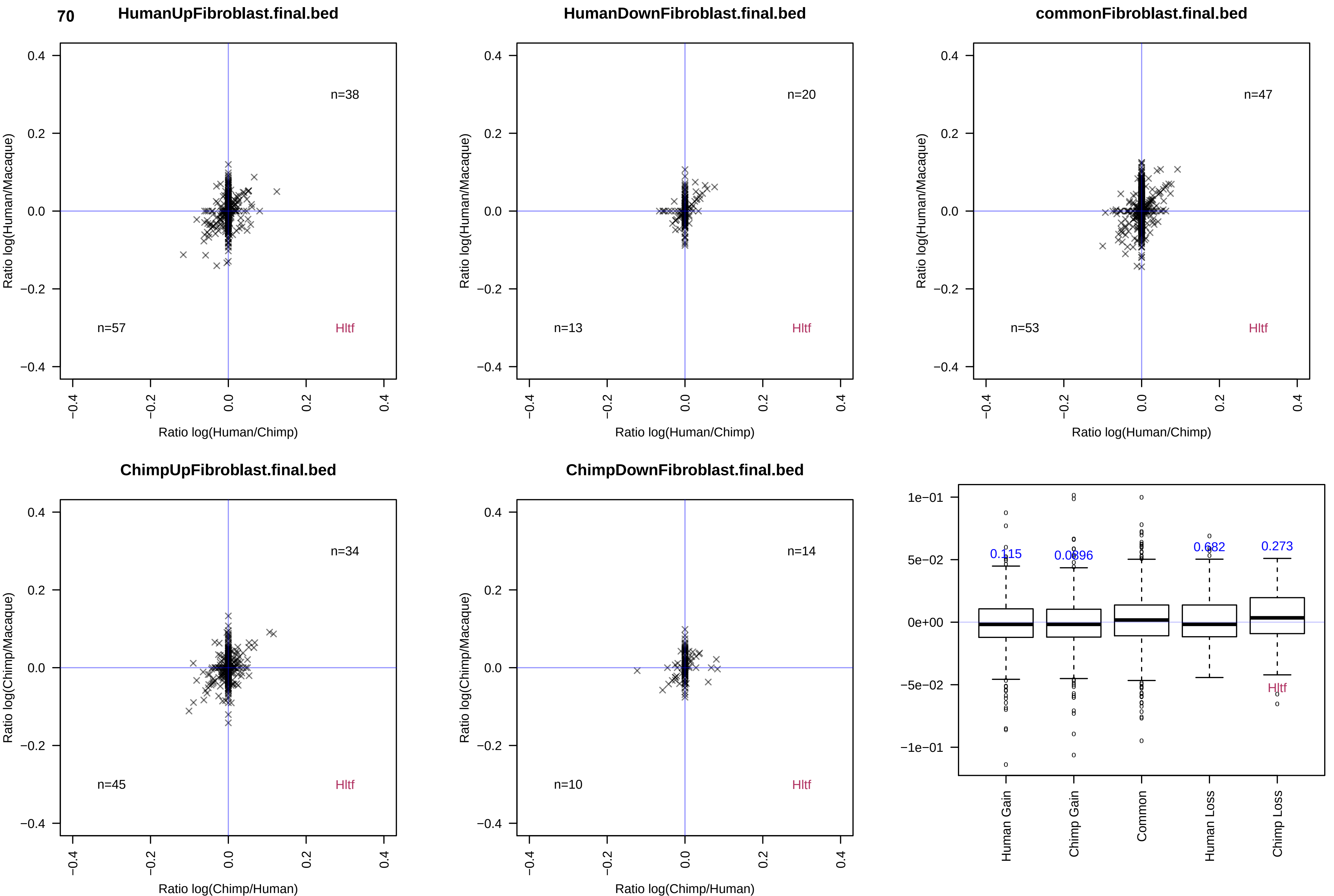
HumanUpFibroblast.final.bed
n=38
n=57
Hltf
Ratio log(Human/Macaque)
Ratio log(Human/Chimp)
HumanDownFibroblast.final.bed
n=20
n=13
Hltf
Ratio log(Human/Macaque)
Ratio log(Human/Chimp)
commonFibroblast.final.bed
n=47
n=53
Hltf
Ratio log(Human/Macaque)
Ratio log(Human/Chimp)
ChimpUpFibroblast.final.bed
n=34
n=45
Hltf
Ratio log(Chimp/Macaque)
Ratio log(Chimp/Human)
ChimpDownFibroblast.final.bed
n=14
n=10
Hltf
Ratio log(Chimp/Macaque)
Ratio log(Chimp/Human)
0.115
0.0896
0.682
0.273
Hltf
Human Gain
Chimp Gain
Common
Human Loss
Chimp Loss

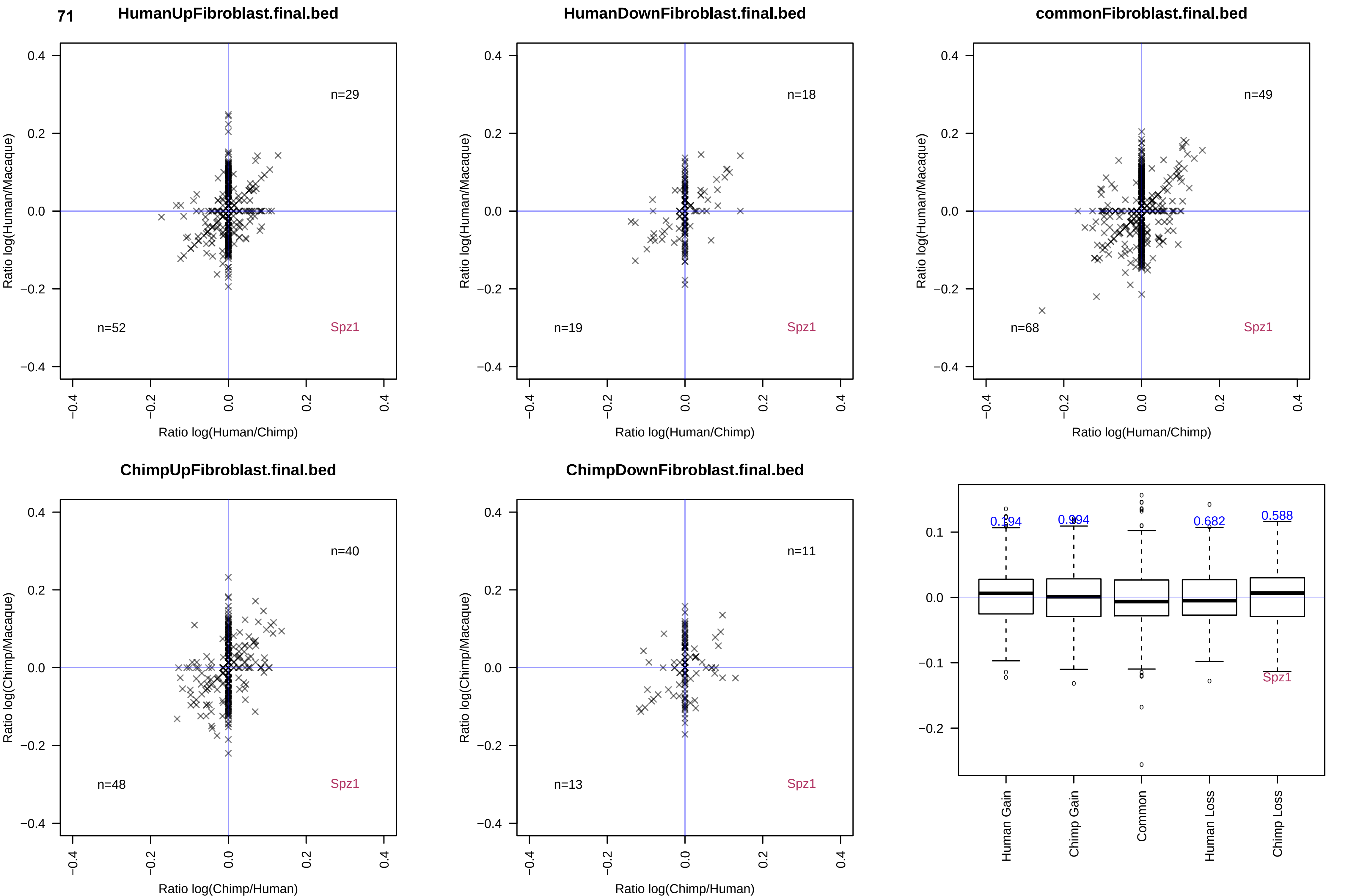
HumanUpFibroblast.final.bed
n=29
n=52
Spz1
Ratio log(Human/Chimp)
Ratio log(Human/Macaque)
HumanDownFibroblast.final.bed
n=18
n=19
Spz1
Ratio log(Human/Chimp)
Ratio log(Human/Macaque)
commonFibroblast.final.bed
n=49
n=68
Spz1
Ratio log(Human/Chimp)
Ratio log(Human/Macaque)
ChimpUpFibroblast.final.bed
n=40
n=48
Spz1
Ratio log(Chimp/Human)
Ratio log(Chimp/Macaque)
ChimpDownFibroblast.final.bed
n=11
n=13
Spz1
Ratio log(Chimp/Human)
Ratio log(Chimp/Macaque)
0.194
0.994
0.682
0.588
Spz1
Human Gain
Chimp Gain
Common
Human Loss
Chimp Loss

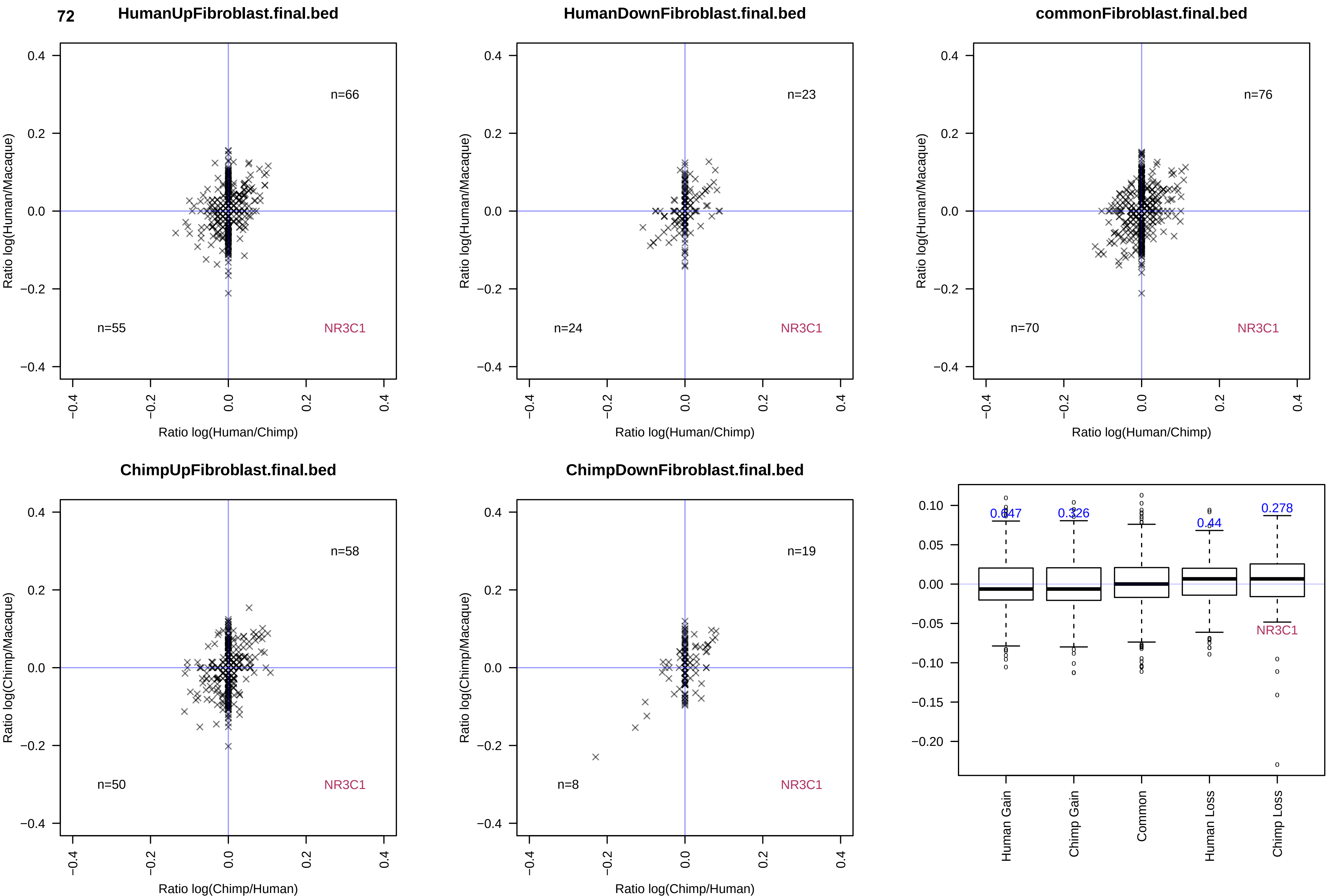
HumanUpFibroblast.final.bed
n=66
n=55
NR3C1
Ratio log(Human/Macaque)
Ratio log(Human/Chimp)
HumanDownFibroblast.final.bed
n=23
n=24
NR3C1
Ratio log(Human/Macaque)
Ratio log(Human/Chimp)
commonFibroblast.final.bed
n=76
n=70
NR3C1
Ratio log(Human/Macaque)
Ratio log(Human/Chimp)
ChimpUpFibroblast.final.bed
n=58
n=50
NR3C1
Ratio log(Chimp/Macaque)
Ratio log(Chimp/Human)
ChimpDownFibroblast.final.bed
n=19
n=8
NR3C1
Ratio log(Chimp/Macaque)
Ratio log(Chimp/Human)
0.647
0.326
0.44
0.278
NR3C1
Human Gain
Chimp Gain
Common
Human Loss
Chimp Loss

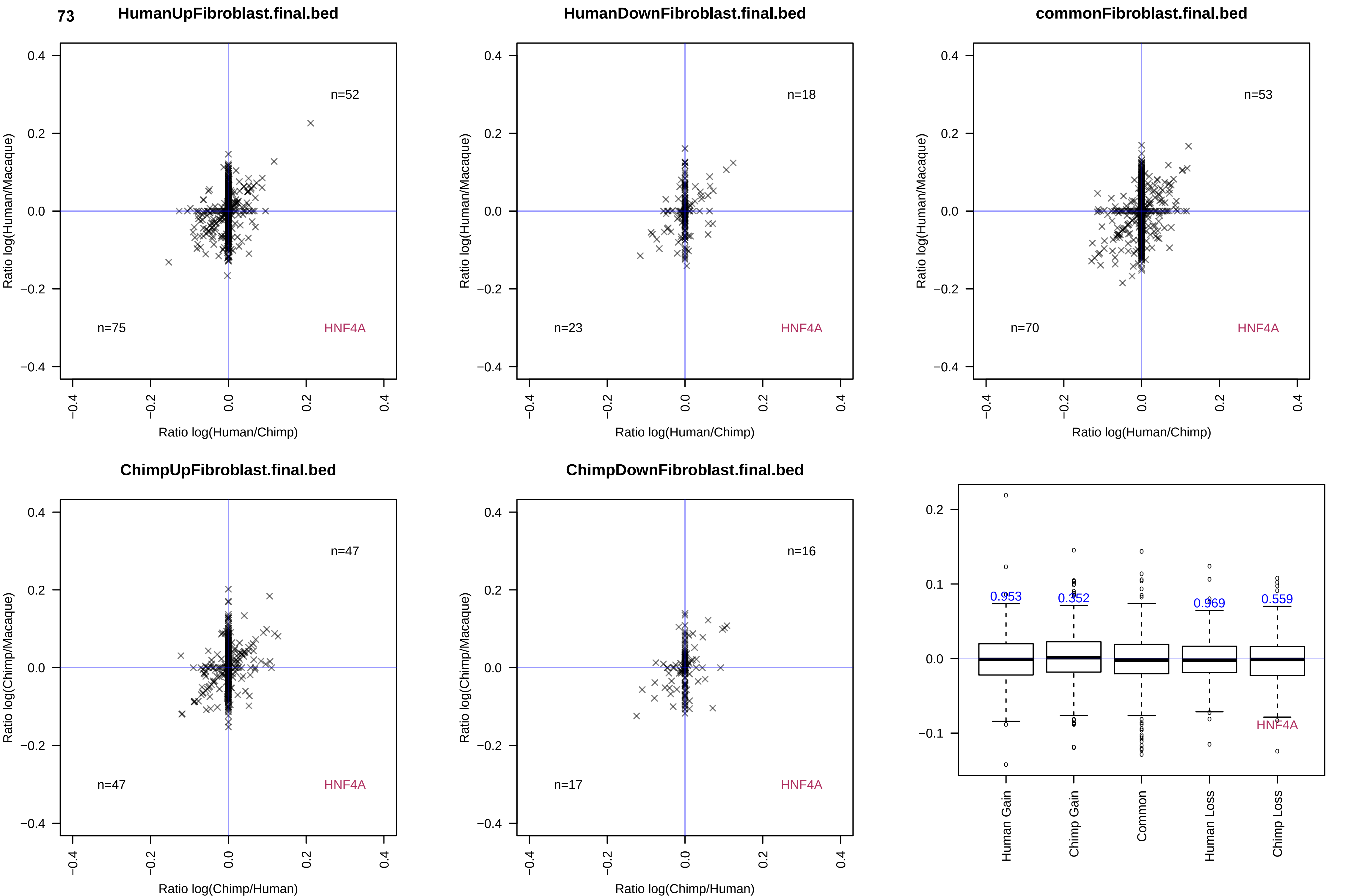
HumanUpFibroblast.final.bed
n=52
n=75
HNF4A
Ratio log(Human/Macaque)
Ratio log(Human/Chimp)
HumanDownFibroblast.final.bed
n=18
n=23
HNF4A
Ratio log(Human/Macaque)
Ratio log(Human/Chimp)
commonFibroblast.final.bed
n=53
n=70
HNF4A
Ratio log(Human/Macaque)
Ratio log(Human/Chimp)
ChimpUpFibroblast.final.bed
n=47
n=47
HNF4A
Ratio log(Chimp/Macaque)
Ratio log(Chimp/Human)
ChimpDownFibroblast.final.bed
n=16
n=17
HNF4A
Ratio log(Chimp/Macaque)
Ratio log(Chimp/Human)
0.953
0.352
0.969
0.559
HNF4A
Human Gain
Chimp Gain
Common
Human Loss
Chimp Loss

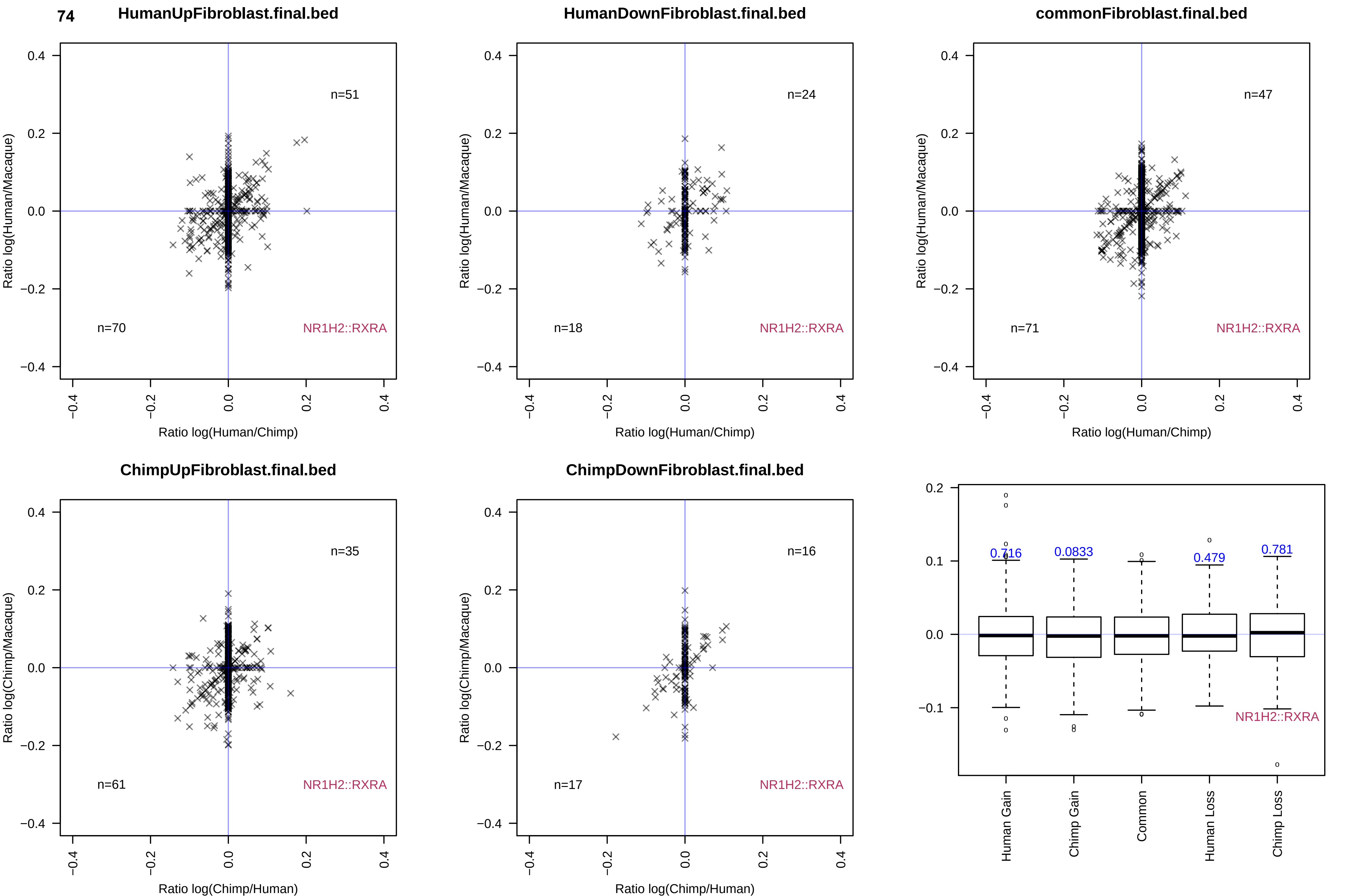
HumanUpFibroblast.final.bed
n=51
n=70
NR1H2::RXRA
Ratio log(Human/Macaque)
Ratio log(Human/Chimp)
HumanDownFibroblast.final.bed
n=24
n=18
NR1H2::RXRA
Ratio log(Human/Macaque)
Ratio log(Human/Chimp)
commonFibroblast.final.bed
n=47
n=71
NR1H2::RXRA
Ratio log(Human/Macaque)
Ratio log(Human/Chimp)
ChimpUpFibroblast.final.bed
n=35
n=61
NR1H2::RXRA
Ratio log(Chimp/Macaque)
Ratio log(Chimp/Human)
ChimpDownFibroblast.final.bed
n=16
n=17
NR1H2::RXRA
Ratio log(Chimp/Macaque)
Ratio log(Chimp/Human)
0.716
0.0833
0.479
0.781
NR1H2::RXRA
Human Gain
Chimp Gain
Common
Human Loss
Chimp Loss

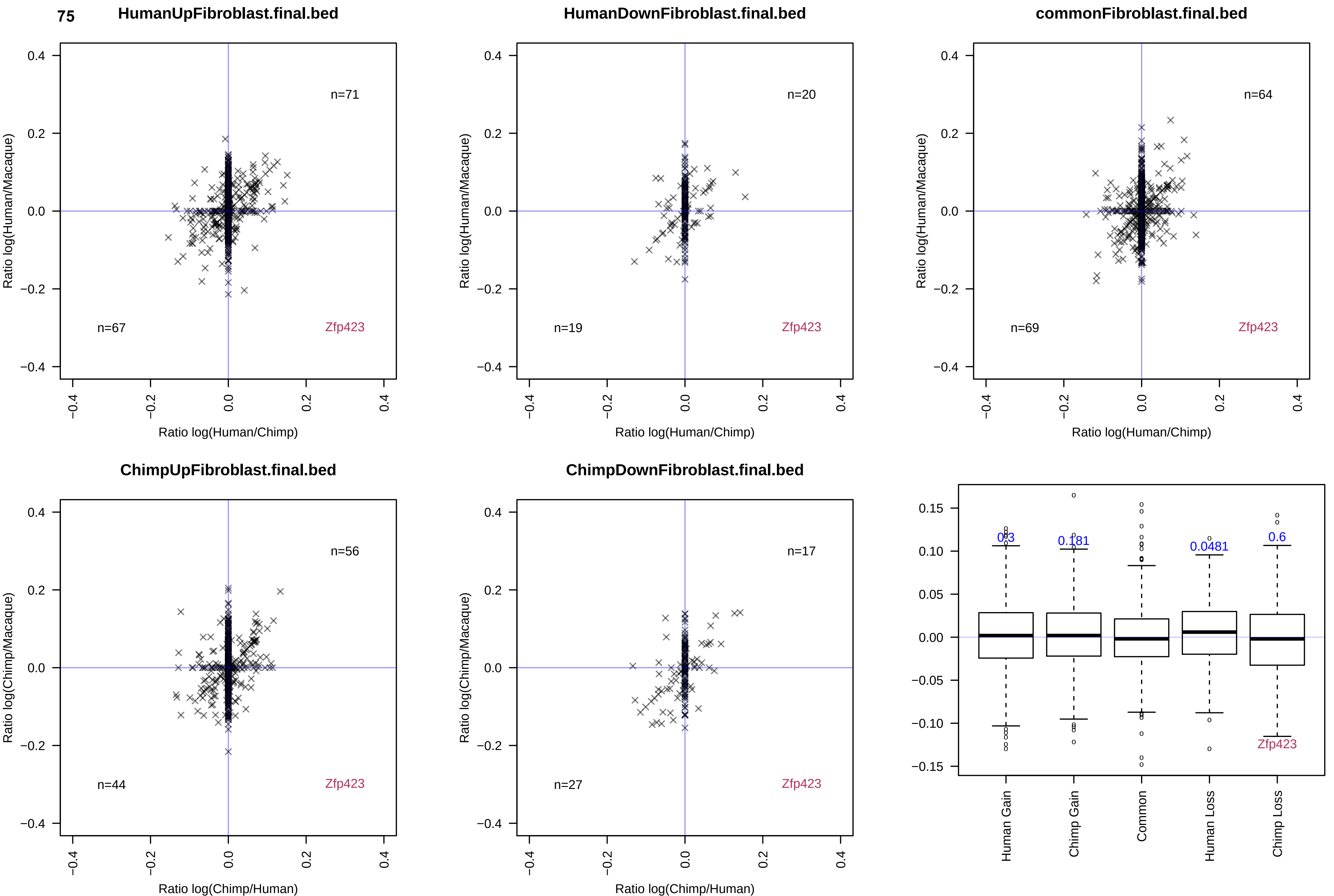
HumanUpFibroblast.final.bed
n=71
n=67
Zfp423
Ratio log(Human/Macaque)
Ratio log(Human/Chimp)
HumanDownFibroblast.final.bed
n=20
n=19
Zfp423
Ratio log(Human/Macaque)
Ratio log(Human/Chimp)
commonFibroblast.final.bed
n=64
n=69
Zfp423
Ratio log(Human/Macaque)
Ratio log(Human/Chimp)
ChimpUpFibroblast.final.bed
n=56
n=44
Zfp423
Ratio log(Chimp/Macaque)
Ratio log(Chimp/Human)
ChimpDownFibroblast.final.bed
n=17
n=27
Zfp423
Ratio log(Chimp/Macaque)
Ratio log(Chimp/Human)
0.3
0.181
0.0481
0.6
Zfp423
Human Gain
Chimp Gain
Common
Human Loss
Chimp Loss

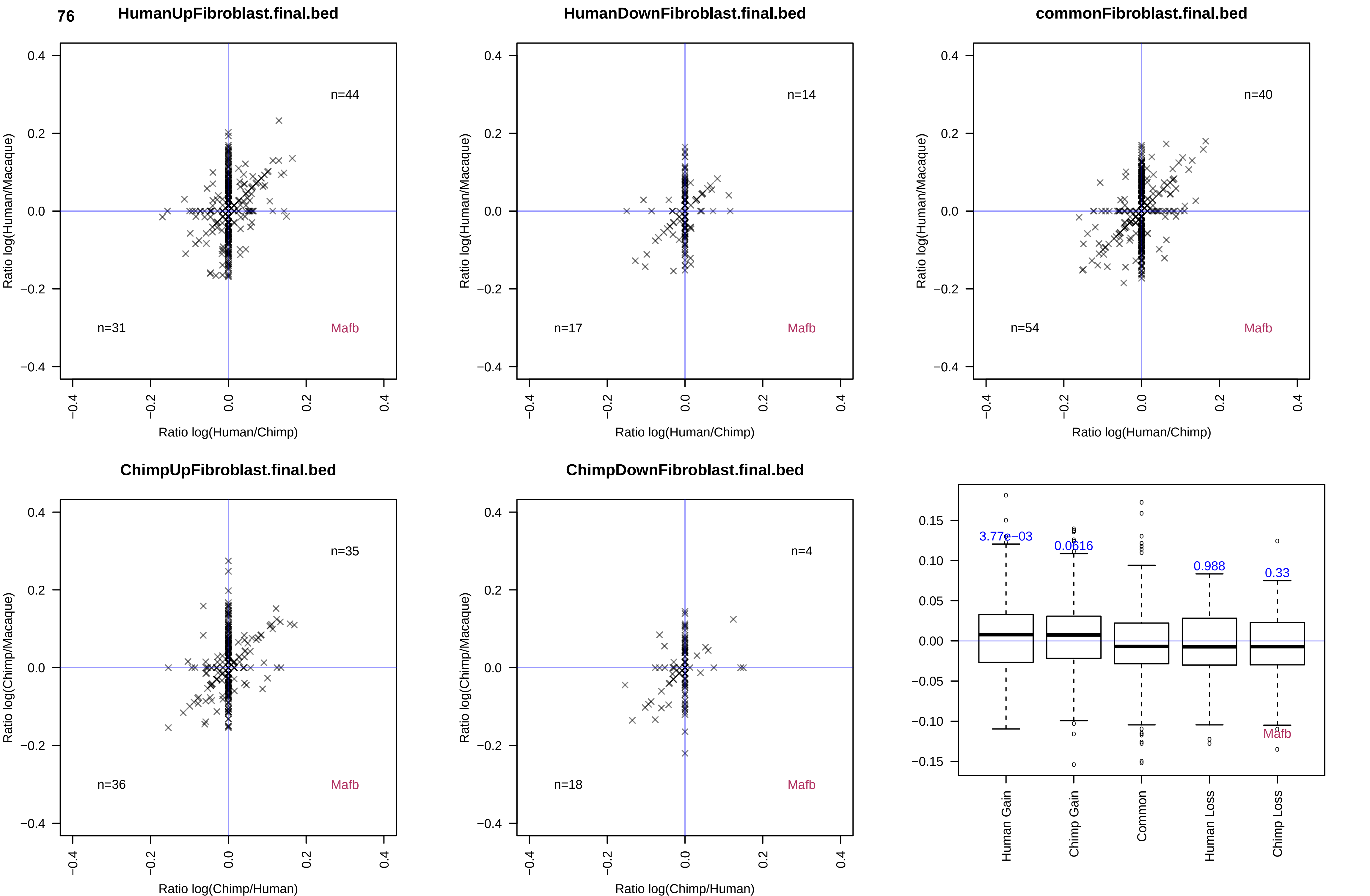
HumanUpFibroblast.final.bed
n=44
n=31
Mafb
Ratio log(Human/Chimp)
Ratio log(Human/Macaque)
HumanDownFibroblast.final.bed
n=14
n=17
Mafb
Ratio log(Human/Chimp)
Ratio log(Human/Macaque)
commonFibroblast.final.bed
n=40
n=54
Mafb
Ratio log(Human/Chimp)
Ratio log(Human/Macaque)
ChimpUpFibroblast.final.bed
n=35
n=36
Mafb
Ratio log(Chimp/Human)
Ratio log(Chimp/Macaque)
ChimpDownFibroblast.final.bed
n=4
n=18
Mafb
Ratio log(Chimp/Human)
Ratio log(Chimp/Macaque)
3.77e−03
0.0616
0.988
0.33
Mafb
Human Gain
Chimp Gain
Common
Human Loss
Chimp Loss

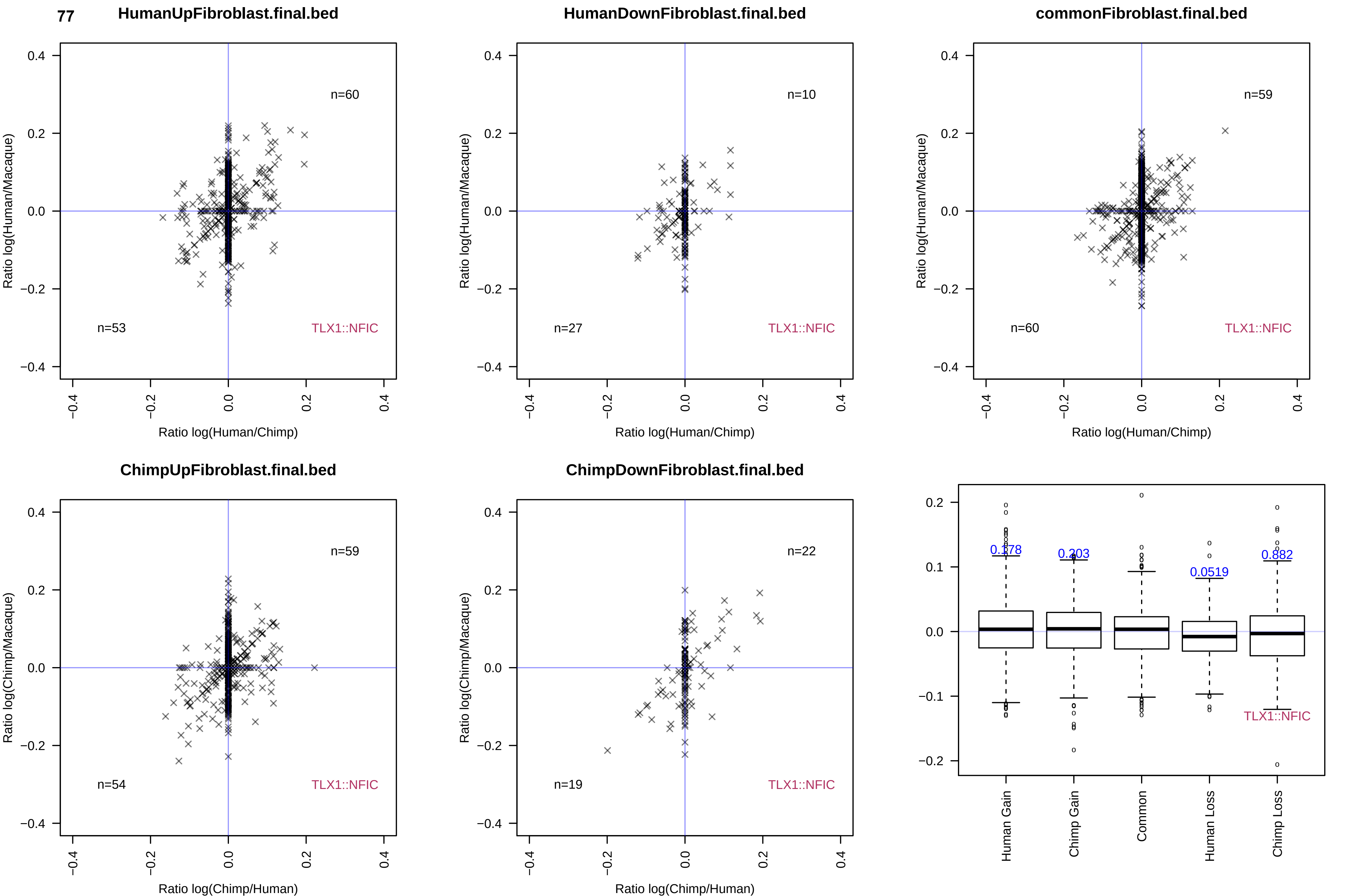
HumanUpFibroblast.final.bed
n=60
n=53
TLX1::NFIC
Ratio log(Human/Macaque)
Ratio log(Human/Chimp)
HumanDownFibroblast.final.bed
n=10
n=27
TLX1::NFIC
Ratio log(Human/Macaque)
Ratio log(Human/Chimp)
commonFibroblast.final.bed
n=59
n=60
TLX1::NFIC
Ratio log(Human/Macaque)
Ratio log(Human/Chimp)
ChimpUpFibroblast.final.bed
n=59
n=54
TLX1::NFIC
Ratio log(Chimp/Macaque)
Ratio log(Chimp/Human)
ChimpDownFibroblast.final.bed
n=22
n=19
TLX1::NFIC
Ratio log(Chimp/Macaque)
Ratio log(Chimp/Human)
0.178
0.203
0.0519
0.882
TLX1::NFIC
Human Gain
Chimp Gain
Common
Human Loss
Chimp Loss

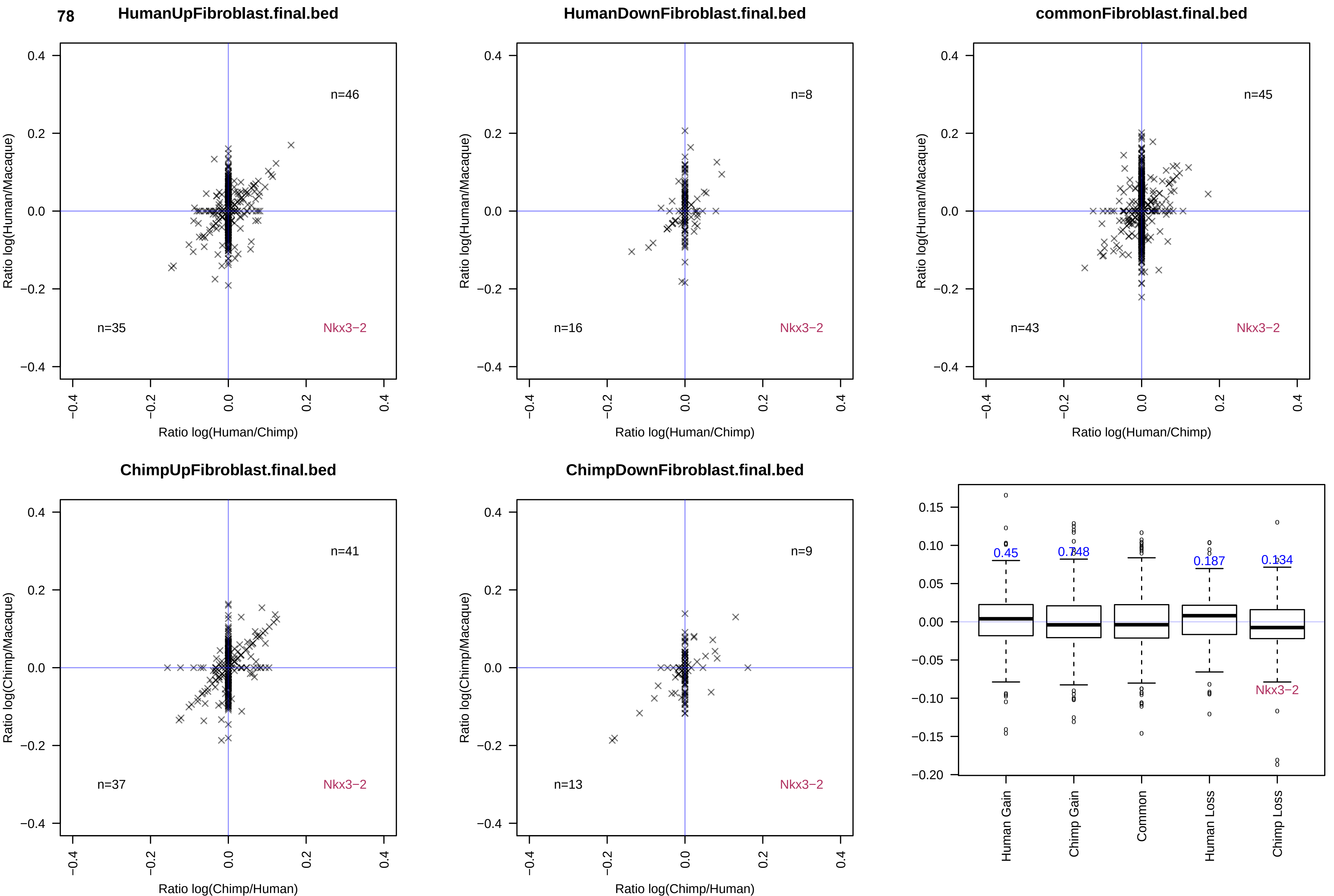
HumanUpFibroblast.final.bed
HumanDownFibroblast.final.bed
commonFibroblast.final.bed
ChimpUpFibroblast.final.bed
ChimpDownFibroblast.final.bed
Ratio log(Human/Macaque)
Ratio log(Human/Chimp)
Ratio log(Chimp/Macaque)
Ratio log(Chimp/Human)
n=46
n=35
n=8
n=16
n=45
n=43
n=41
n=37
n=9
n=13
Nkx3−2
0.45
0.748
0.187
0.134
Human Gain
Chimp Gain
Common
Human Loss
Chimp Loss

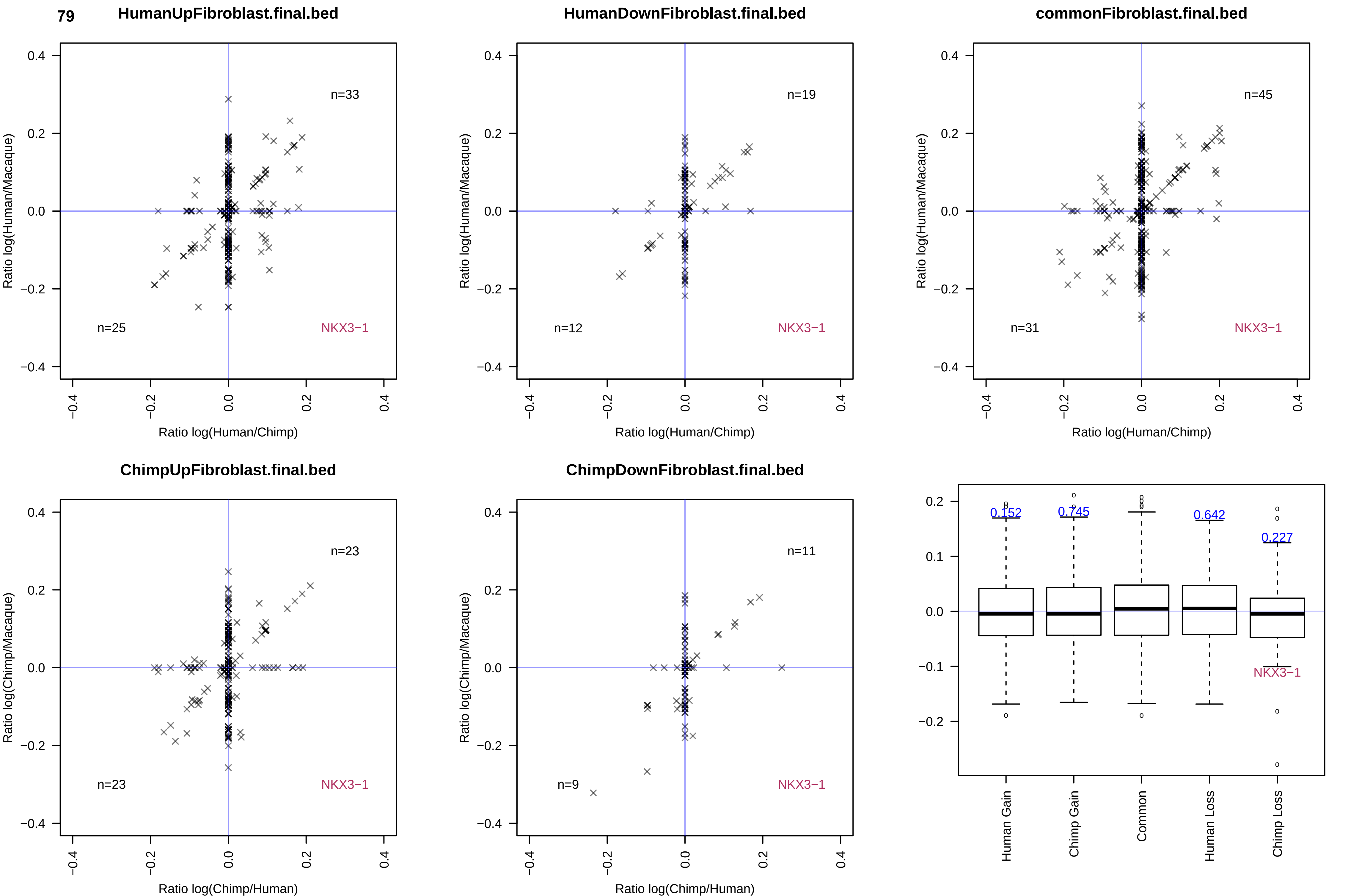
HumanUpFibroblast.final.bed
n=33
n=25
NKX3−1
Ratio log(Human/Macaque)
Ratio log(Human/Chimp)
HumanDownFibroblast.final.bed
n=19
n=12
NKX3−1
Ratio log(Human/Macaque)
Ratio log(Human/Chimp)
commonFibroblast.final.bed
n=45
n=31
NKX3−1
Ratio log(Human/Macaque)
Ratio log(Human/Chimp)
ChimpUpFibroblast.final.bed
n=23
n=23
NKX3−1
Ratio log(Chimp/Macaque)
Ratio log(Chimp/Human)
ChimpDownFibroblast.final.bed
n=11
n=9
NKX3−1
Ratio log(Chimp/Macaque)
Ratio log(Chimp/Human)
0.152
0.745
0.642
0.227
NKX3−1
Human Gain
Chimp Gain
Common
Human Loss
Chimp Loss

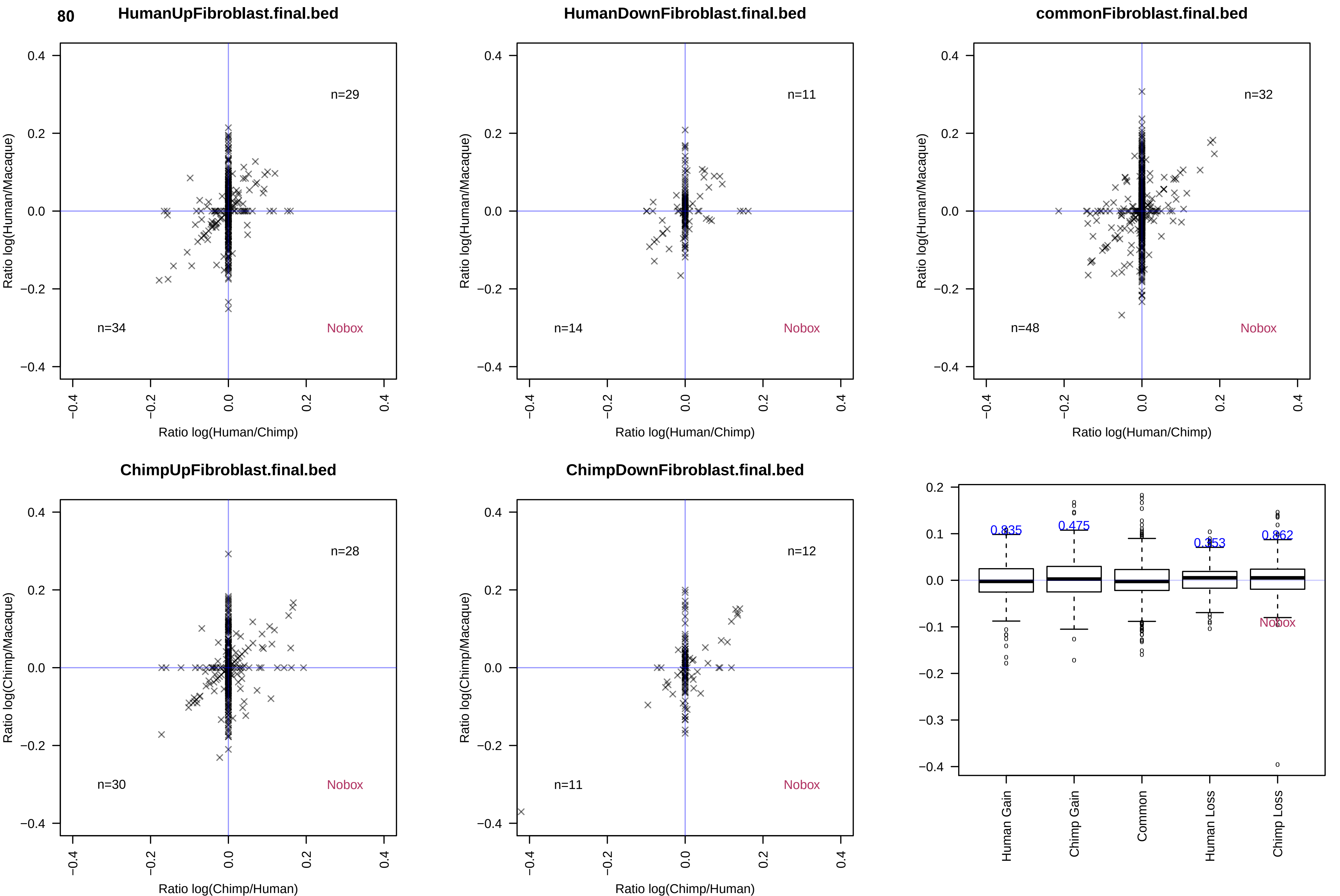
HumanUpFibroblast.final.bed
HumanDownFibroblast.final.bed
commonFibroblast.final.bed
ChimpUpFibroblast.final.bed
ChimpDownFibroblast.final.bed
Ratio log(Human/Macaque)
Ratio log(Human/Chimp)
Ratio log(Chimp/Macaque)
Ratio log(Chimp/Human)
n=29
n=34
n=11
n=14
n=32
n=48
n=28
n=30
n=12
n=11
Nobox
0.835
0.475
0.353
0.862
Human Gain
Chimp Gain
Common
Human Loss
Chimp Loss

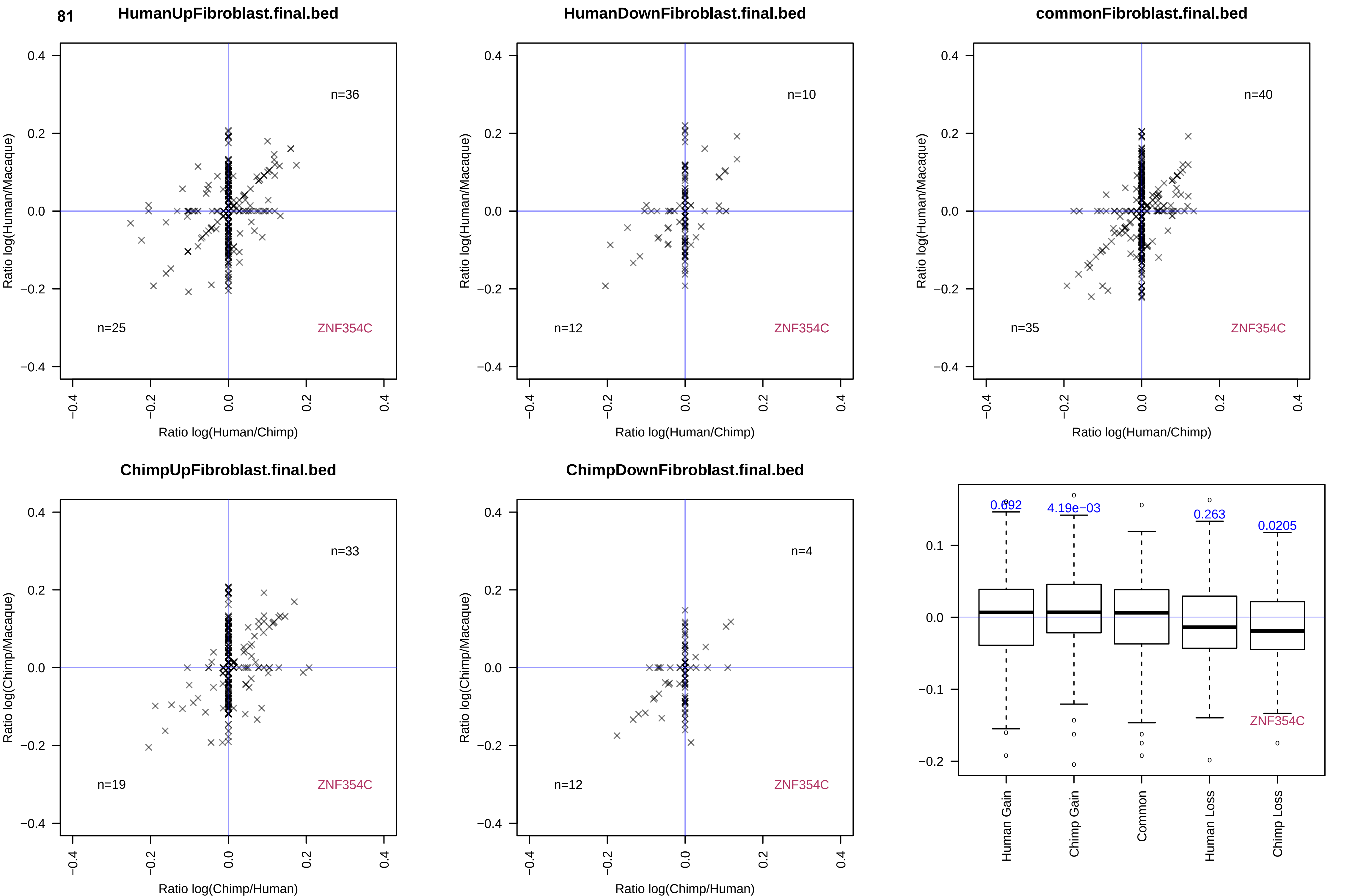
HumanUpFibroblast.final.bed
HumanDownFibroblast.final.bed
commonFibroblast.final.bed
ChimpUpFibroblast.final.bed
ChimpDownFibroblast.final.bed
Ratio log(Human/Macaque)
Ratio log(Human/Chimp)
Ratio log(Chimp/Macaque)
Ratio log(Chimp/Human)
n=36
n=25
n=10
n=12
n=40
n=35
n=33
n=19
n=4
n=12
ZNF354C
0.692
4.19e−03
0.263
0.0205
Human Gain
Chimp Gain
Common
Human Loss
Chimp Loss

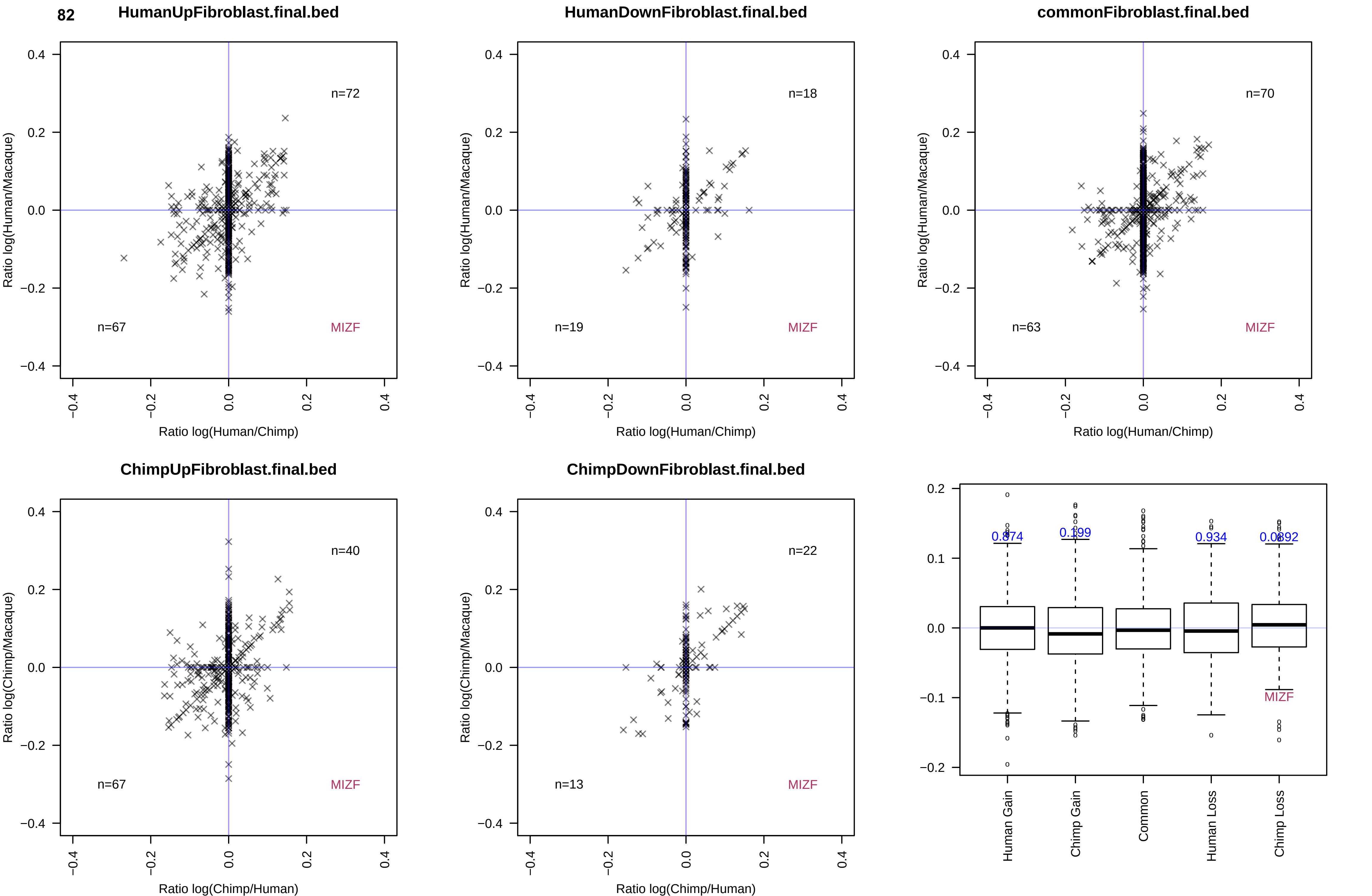
HumanUpFibroblast.final.bed
n=72
n=67
MIZF
Ratio log(Human/Macaque)
Ratio log(Human/Chimp)
HumanDownFibroblast.final.bed
n=18
n=19
MIZF
Ratio log(Human/Macaque)
Ratio log(Human/Chimp)
commonFibroblast.final.bed
n=70
n=63
MIZF
Ratio log(Human/Macaque)
Ratio log(Human/Chimp)
ChimpUpFibroblast.final.bed
n=40
n=67
MIZF
Ratio log(Chimp/Macaque)
Ratio log(Chimp/Human)
ChimpDownFibroblast.final.bed
n=22
n=13
MIZF
Ratio log(Chimp/Macaque)
Ratio log(Chimp/Human)
0.874
0.199
0.934
0.0892
MIZF
Human Gain
Chimp Gain
Common
Human Loss
Chimp Loss

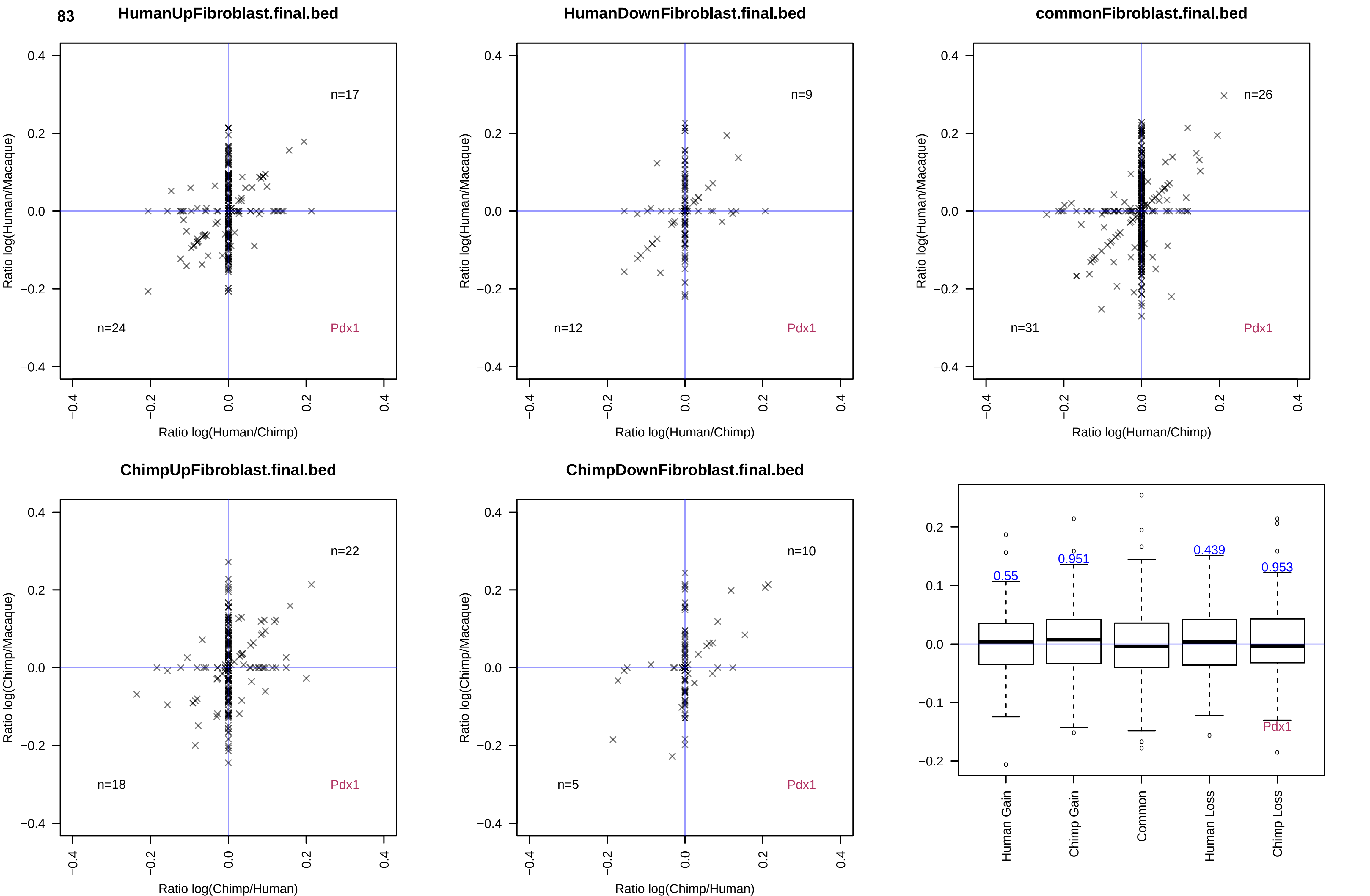
HumanUpFibroblast.final.bed
Ratio log(Human/Macaque)
Ratio log(Human/Chimp)
n=17
n=24
Pdx1
HumanDownFibroblast.final.bed
Ratio log(Human/Macaque)
Ratio log(Human/Chimp)
n=9
n=12
Pdx1
commonFibroblast.final.bed
Ratio log(Human/Macaque)
Ratio log(Human/Chimp)
n=26
n=31
Pdx1
ChimpUpFibroblast.final.bed
Ratio log(Chimp/Macaque)
Ratio log(Chimp/Human)
n=22
n=18
Pdx1
ChimpDownFibroblast.final.bed
Ratio log(Chimp/Macaque)
Ratio log(Chimp/Human)
n=10
n=5
Pdx1
0.55
0.951
0.439
0.953
Pdx1
Human Gain
Chimp Gain
Common
Human Loss
Chimp Loss

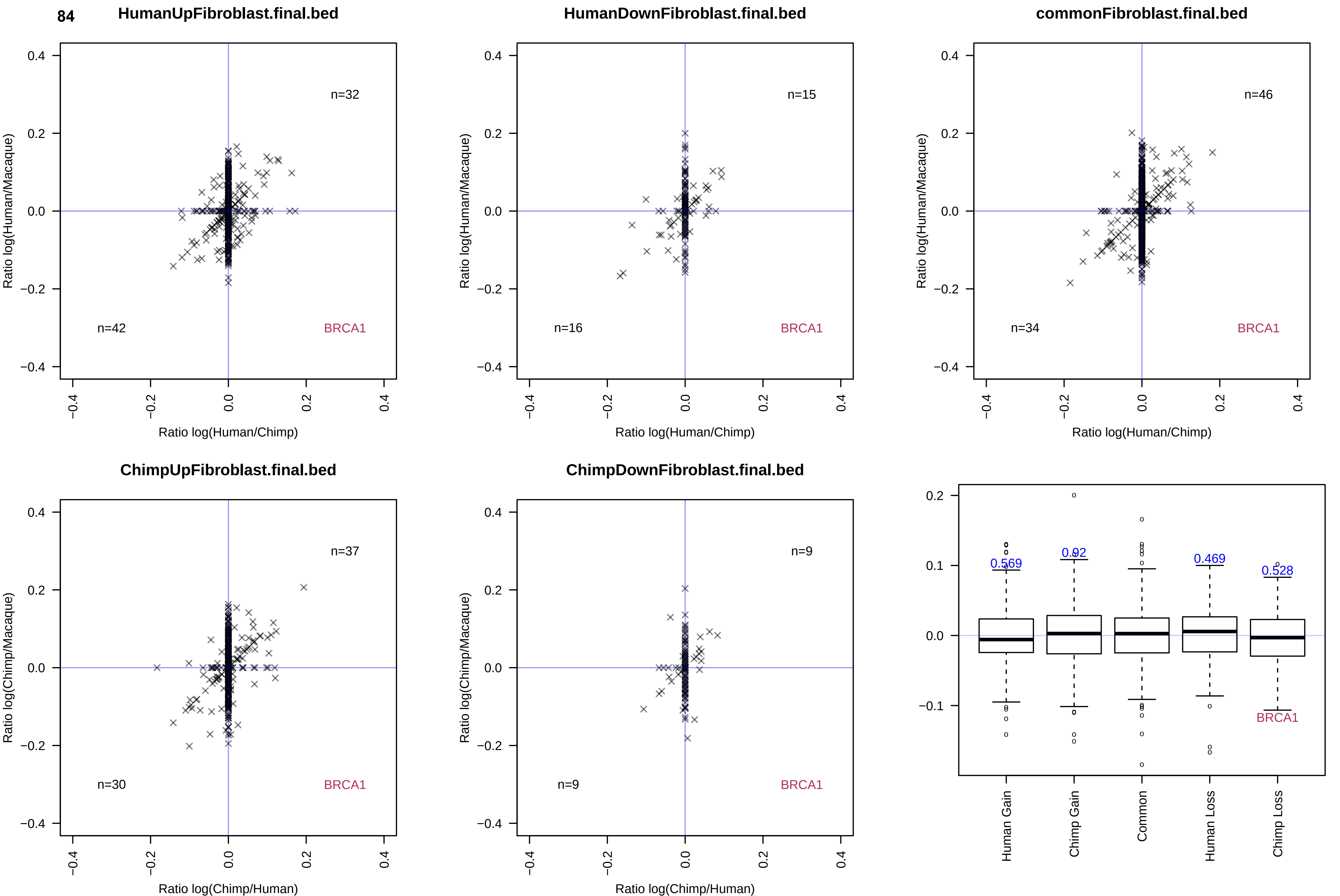
HumanUpFibroblast.final.bed
n=32
n=42
BRCA1
Ratio log(Human/Macaque)
Ratio log(Human/Chimp)
HumanDownFibroblast.final.bed
n=15
n=16
BRCA1
Ratio log(Human/Macaque)
Ratio log(Human/Chimp)
commonFibroblast.final.bed
n=46
n=34
BRCA1
Ratio log(Human/Macaque)
Ratio log(Human/Chimp)
ChimpUpFibroblast.final.bed
n=37
n=30
BRCA1
Ratio log(Chimp/Macaque)
Ratio log(Chimp/Human)
ChimpDownFibroblast.final.bed
n=9
n=9
BRCA1
Ratio log(Chimp/Macaque)
Ratio log(Chimp/Human)
0.569
0.92
0.469
0.528
BRCA1
Human Gain
Chimp Gain
Common
Human Loss
Chimp Loss

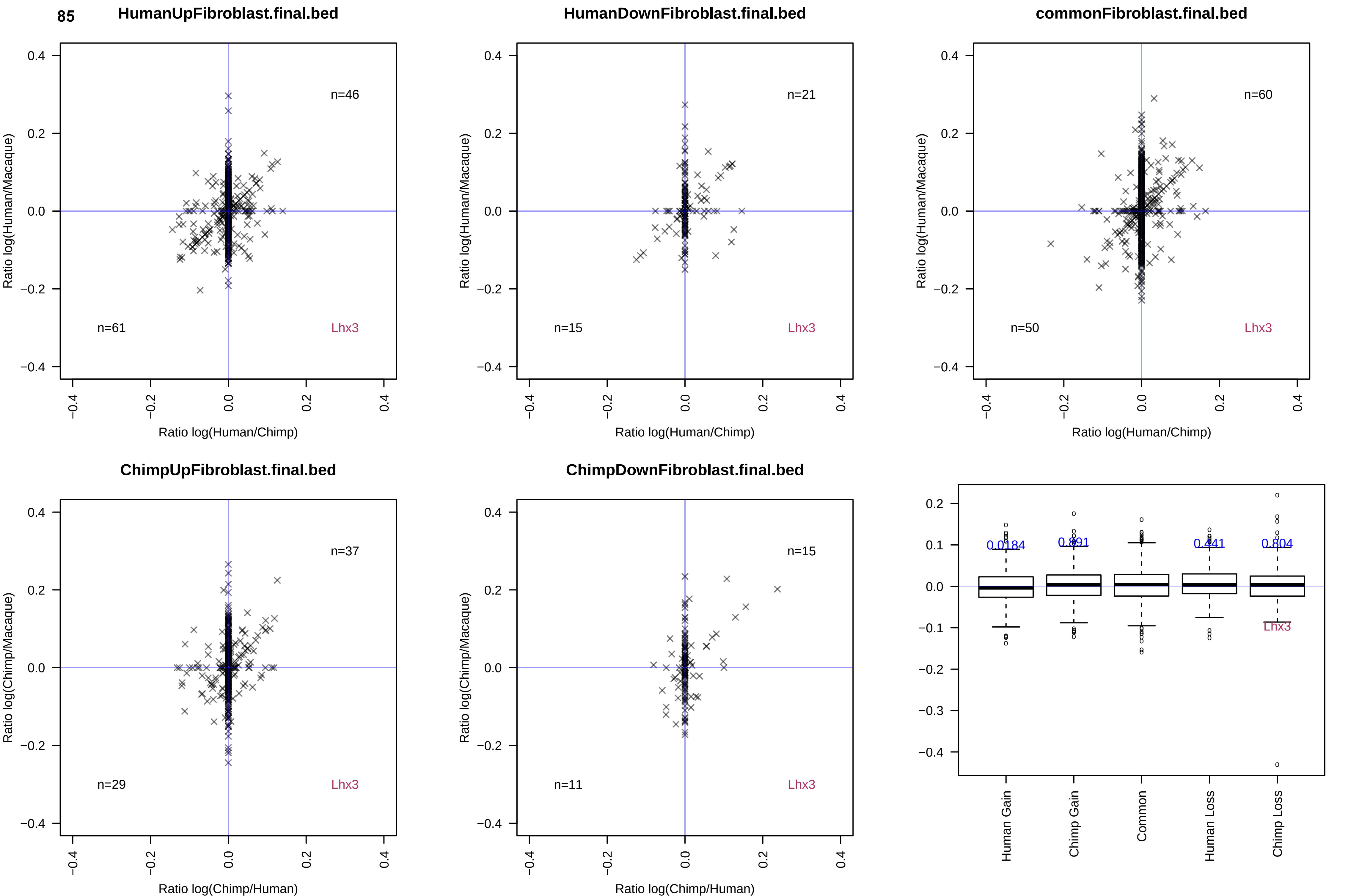
HumanUpFibroblast.final.bed
HumanDownFibroblast.final.bed
commonFibroblast.final.bed
ChimpUpFibroblast.final.bed
ChimpDownFibroblast.final.bed
n=46
n=61
n=21
n=15
n=60
n=50
n=37
n=29
n=15
n=11
Lhx3
Ratio log(Human/Macaque)
Ratio log(Human/Chimp)
Ratio log(Chimp/Macaque)
Ratio log(Chimp/Human)
0.0184
0.891
0.441
0.804
Human Gain
Chimp Gain
Common
Human Loss
Chimp Loss

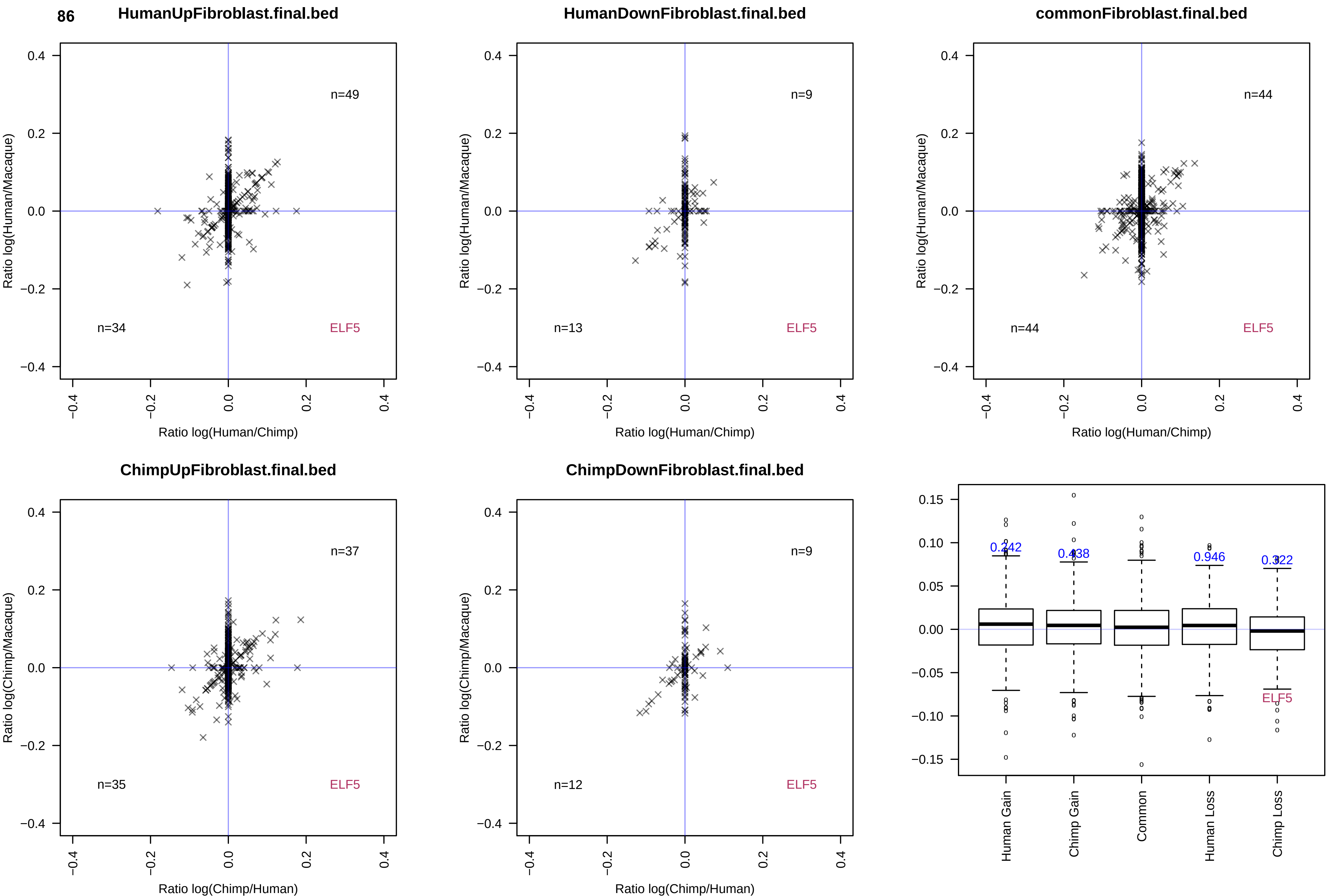
HumanUpFibroblast.final.bed
n=49
n=34
ELF5
Ratio log(Human/Chimp)
Ratio log(Human/Macaque)
HumanDownFibroblast.final.bed
n=9
n=13
ELF5
Ratio log(Human/Chimp)
Ratio log(Human/Macaque)
commonFibroblast.final.bed
n=44
n=44
ELF5
Ratio log(Human/Chimp)
Ratio log(Human/Macaque)
ChimpUpFibroblast.final.bed
n=37
n=35
ELF5
Ratio log(Chimp/Human)
Ratio log(Chimp/Macaque)
ChimpDownFibroblast.final.bed
n=9
n=12
ELF5
Ratio log(Chimp/Human)
Ratio log(Chimp/Macaque)
0.242
0.438
0.946
0.322
ELF5
Human Gain
Chimp Gain
Common
Human Loss
Chimp Loss

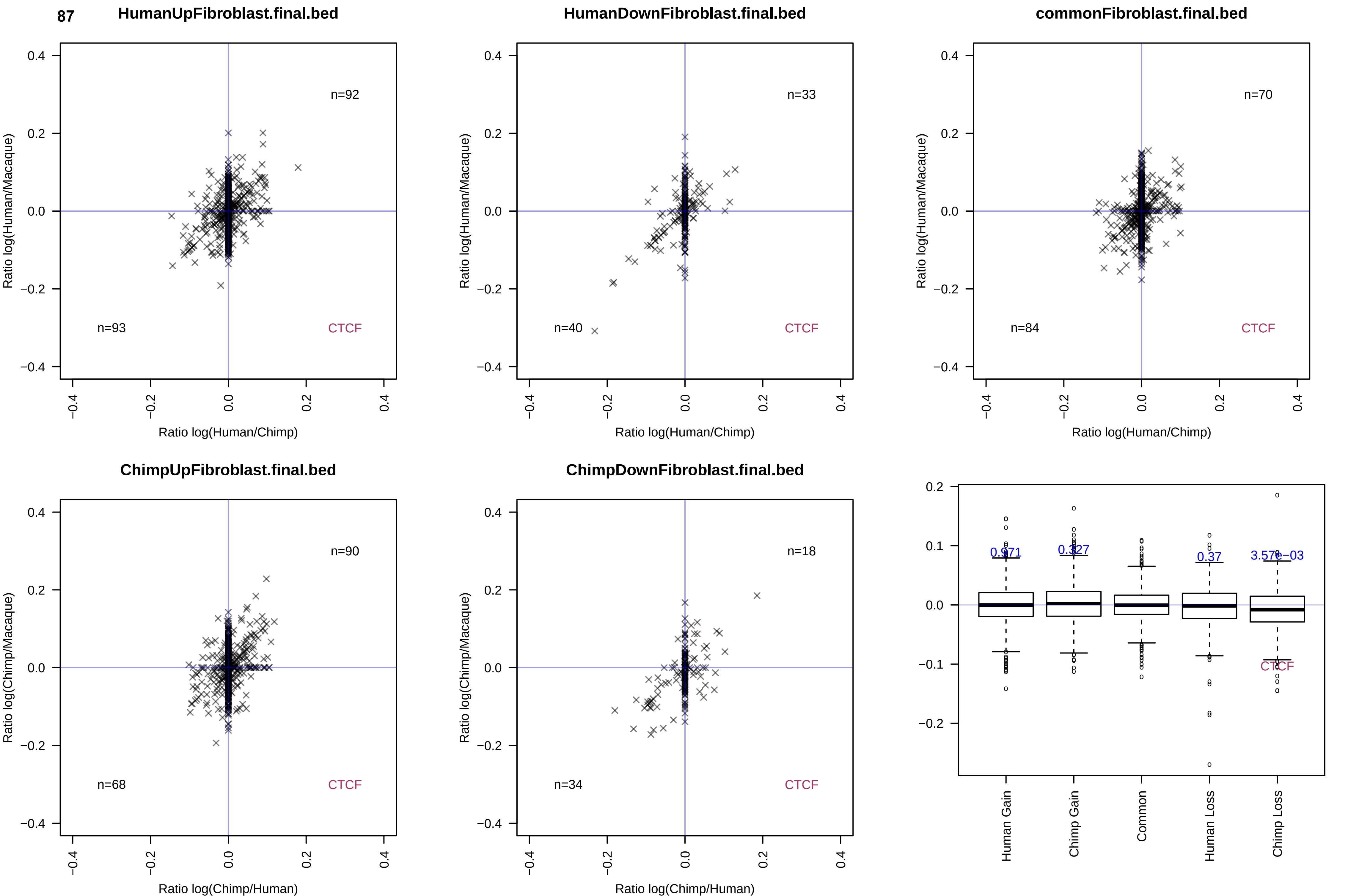
HumanUpFibroblast.final.bed
n=92
n=93
CTCF
Ratio log(Human/Macaque)
Ratio log(Human/Chimp)
HumanDownFibroblast.final.bed
n=33
n=40
CTCF
Ratio log(Human/Macaque)
Ratio log(Human/Chimp)
commonFibroblast.final.bed
n=70
n=84
CTCF
Ratio log(Human/Macaque)
Ratio log(Human/Chimp)
ChimpUpFibroblast.final.bed
n=90
n=68
CTCF
Ratio log(Chimp/Macaque)
Ratio log(Chimp/Human)
ChimpDownFibroblast.final.bed
n=18
n=34
CTCF
Ratio log(Chimp/Macaque)
Ratio log(Chimp/Human)
0.971
0.327
0.37
3.57e−03
CTCF
Human Gain
Chimp Gain
Common
Human Loss
Chimp Loss

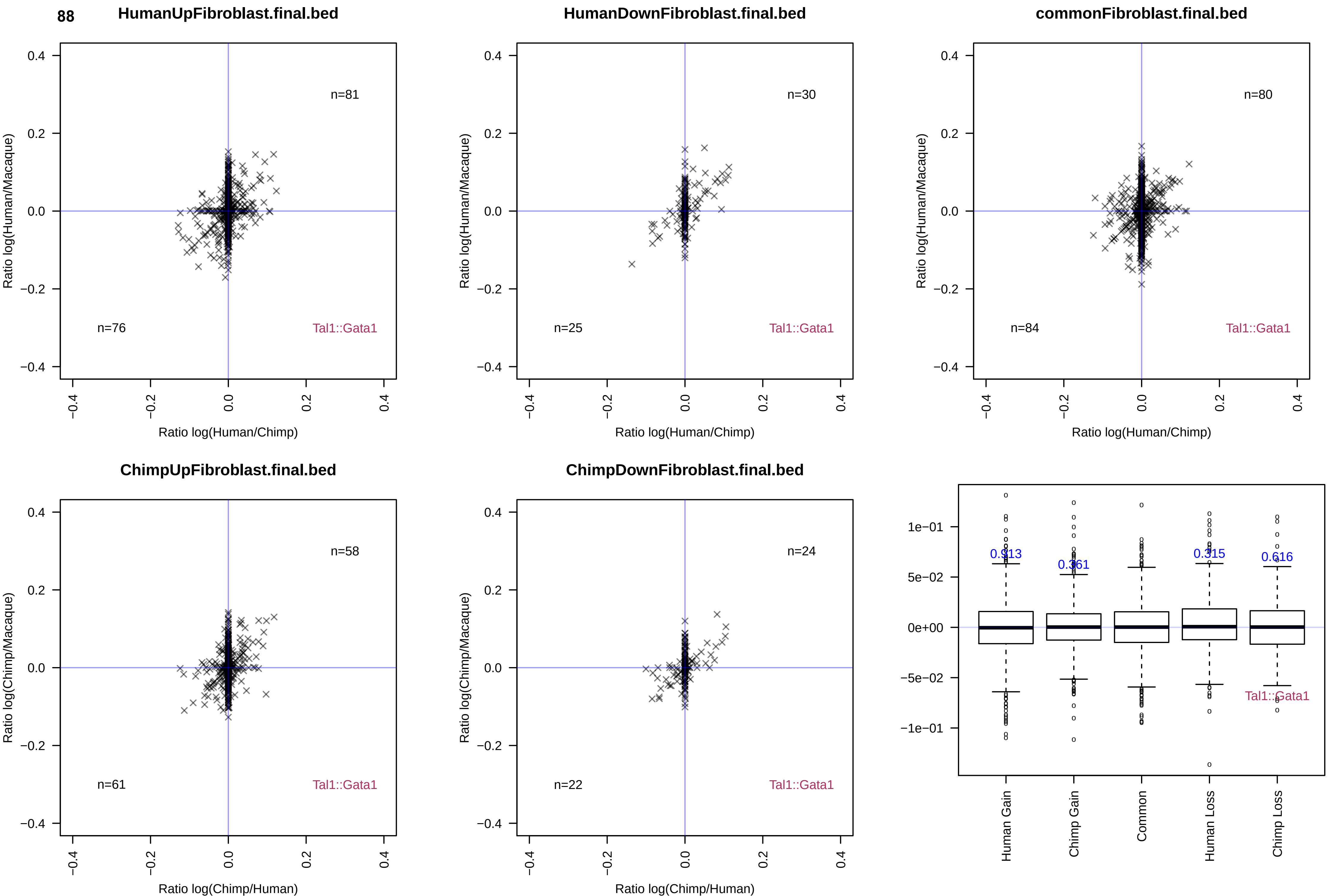
HumanUpFibroblast.final.bed
n=81
n=76
Tal1::Gata1
Ratio log(Human/Chimp)
Ratio log(Human/Macaque)
HumanDownFibroblast.final.bed
n=30
n=25
Tal1::Gata1
Ratio log(Human/Chimp)
Ratio log(Human/Macaque)
commonFibroblast.final.bed
n=80
n=84
Tal1::Gata1
Ratio log(Human/Chimp)
Ratio log(Human/Macaque)
ChimpUpFibroblast.final.bed
n=58
n=61
Tal1::Gata1
Ratio log(Chimp/Human)
Ratio log(Chimp/Macaque)
ChimpDownFibroblast.final.bed
n=24
n=22
Tal1::Gata1
Ratio log(Chimp/Human)
Ratio log(Chimp/Macaque)
−0.4
−0.2
0.0
0.2
0.4
1e−01
5e−02
0e+00
−5e−02
−1e−01
0.913
0.361
0.315
0.616
Tal1::Gata1
Human Gain
Chimp Gain
Common
Human Loss
Chimp Loss

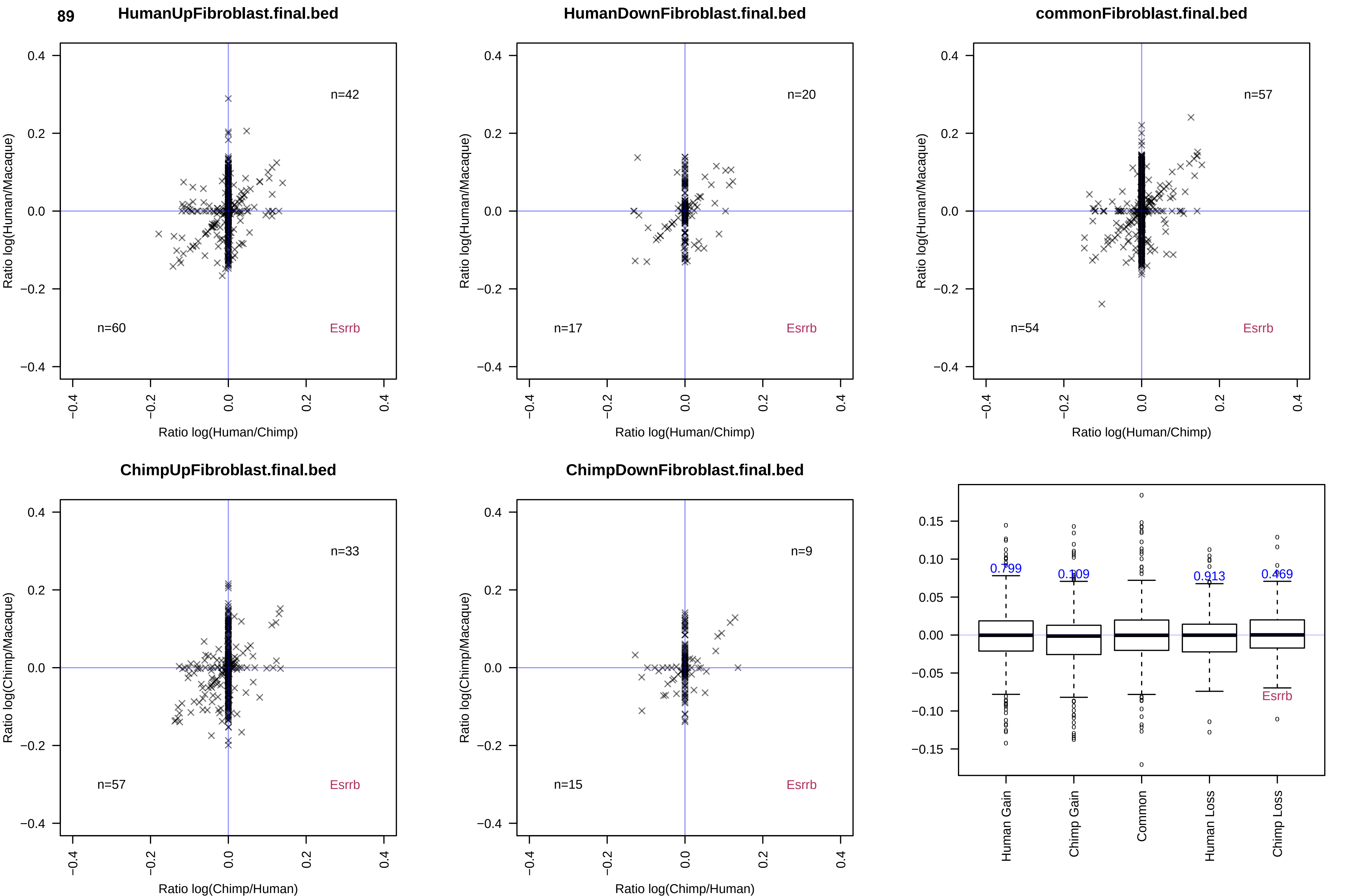
HumanUpFibroblast.final.bed
HumanDownFibroblast.final.bed
commonFibroblast.final.bed
ChimpUpFibroblast.final.bed
ChimpDownFibroblast.final.bed
Ratio log(Human/Macaque)
Ratio log(Human/Chimp)
Ratio log(Chimp/Macaque)
Ratio log(Chimp/Human)
n=42
n=60
n=20
n=17
n=57
n=54
n=33
n=57
n=9
n=15
Esrrb
0.799
0.109
0.913
0.469
Human Gain
Chimp Gain
Common
Human Loss
Chimp Loss

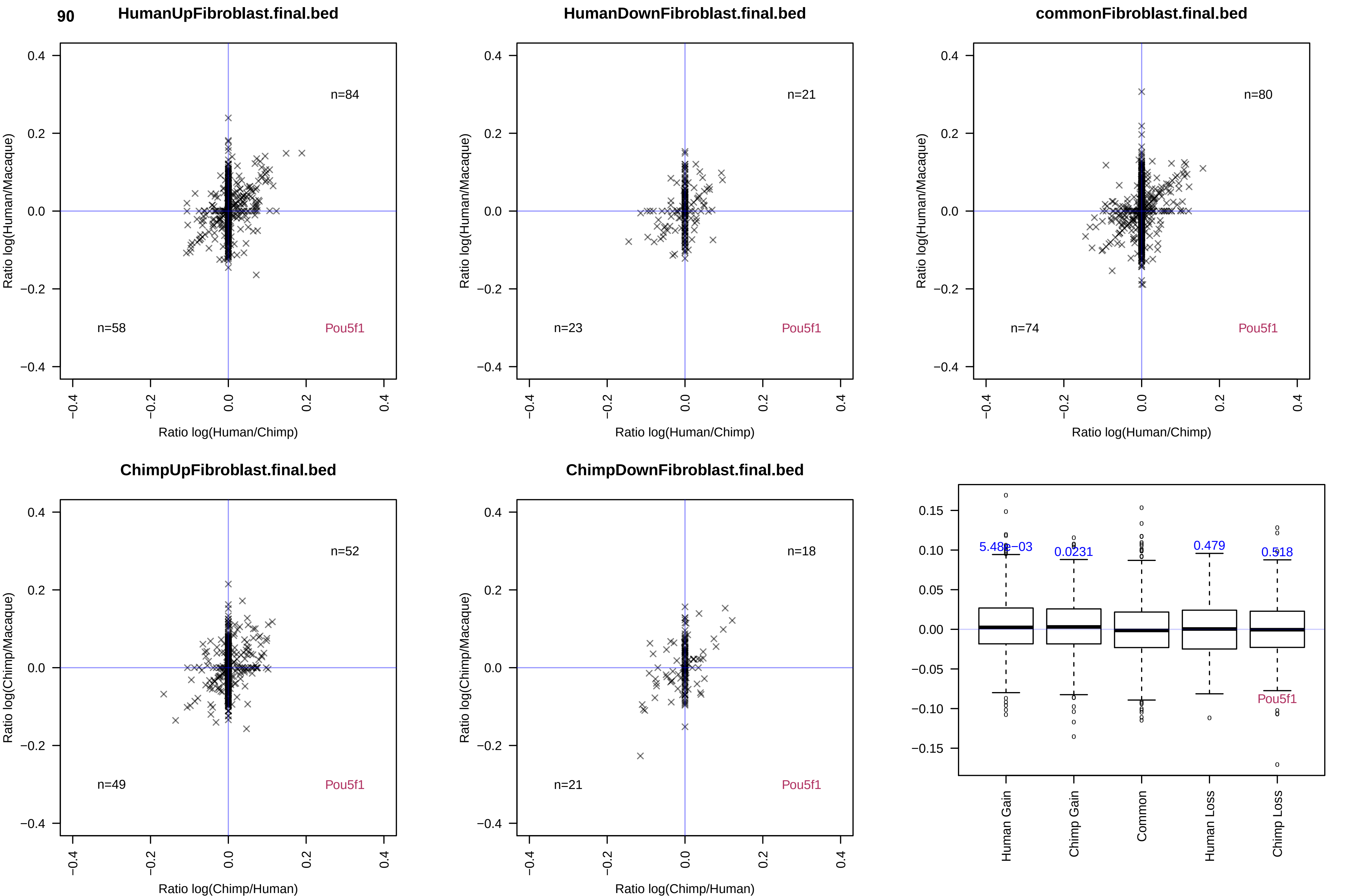
HumanUpFibroblast.final.bed
n=84
n=58
Pou5f1
Ratio log(Human/Macaque)
Ratio log(Human/Chimp)
HumanDownFibroblast.final.bed
n=21
n=23
Pou5f1
Ratio log(Human/Macaque)
Ratio log(Human/Chimp)
commonFibroblast.final.bed
n=80
n=74
Pou5f1
Ratio log(Human/Macaque)
Ratio log(Human/Chimp)
ChimpUpFibroblast.final.bed
n=52
n=49
Pou5f1
Ratio log(Chimp/Macaque)
Ratio log(Chimp/Human)
ChimpDownFibroblast.final.bed
n=18
n=21
Pou5f1
Ratio log(Chimp/Macaque)
Ratio log(Chimp/Human)
5.48e−03
0.0231
0.479
0.518
Pou5f1
Human Gain
Chimp Gain
Common
Human Loss
Chimp Loss

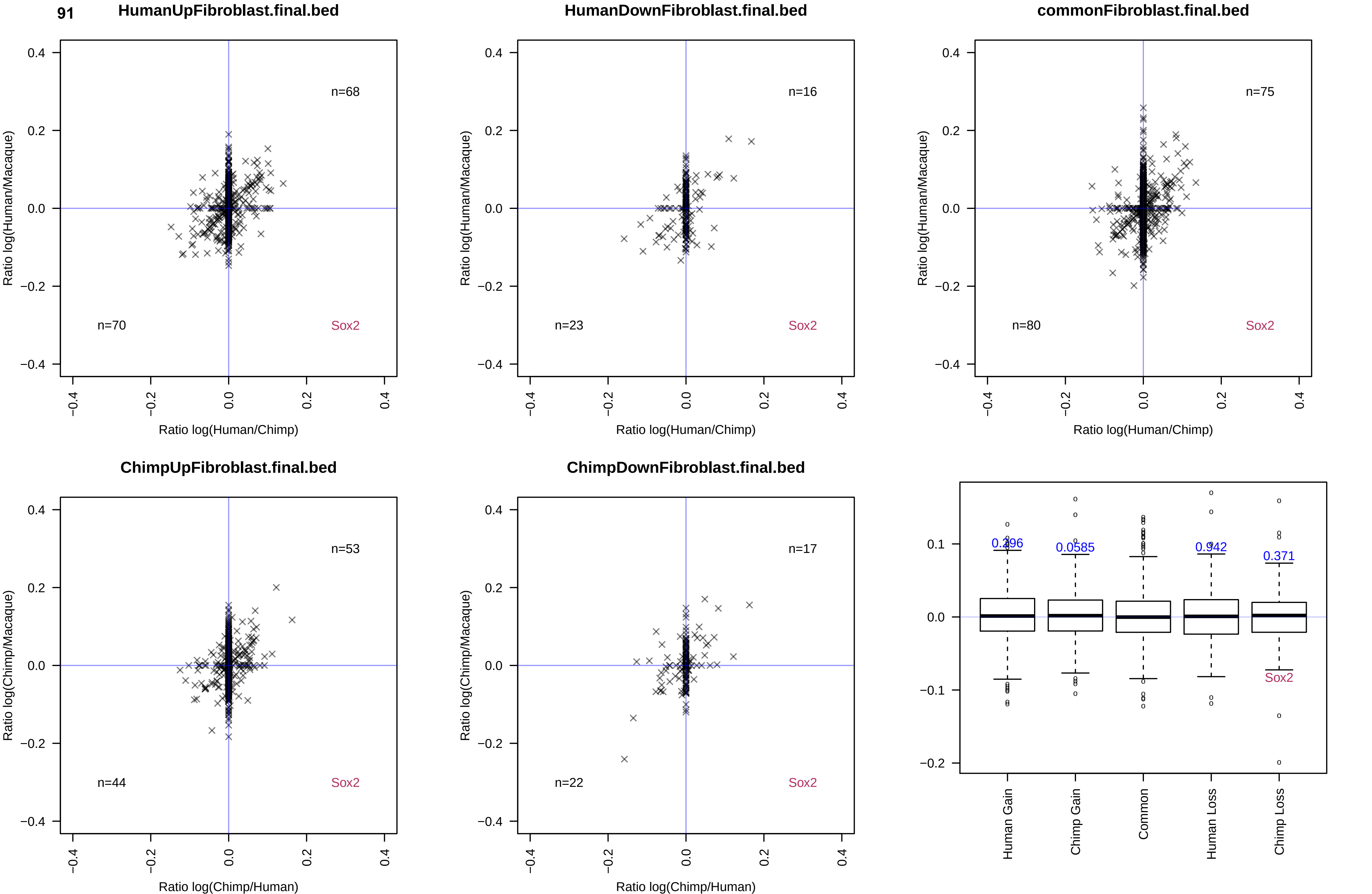
HumanUpFibroblast.final.bed
n=68
n=70
Sox2
Ratio log(Human/Macaque)
Ratio log(Human/Chimp)
HumanDownFibroblast.final.bed
n=16
n=23
Sox2
Ratio log(Human/Macaque)
Ratio log(Human/Chimp)
commonFibroblast.final.bed
n=75
n=80
Sox2
Ratio log(Human/Macaque)
Ratio log(Human/Chimp)
ChimpUpFibroblast.final.bed
n=53
n=44
Sox2
Ratio log(Chimp/Macaque)
Ratio log(Chimp/Human)
ChimpDownFibroblast.final.bed
n=17
n=22
Sox2
Ratio log(Chimp/Macaque)
Ratio log(Chimp/Human)
0.296
0.0585
0.942
0.371
Sox2
Human Gain
Chimp Gain
Common
Human Loss
Chimp Loss

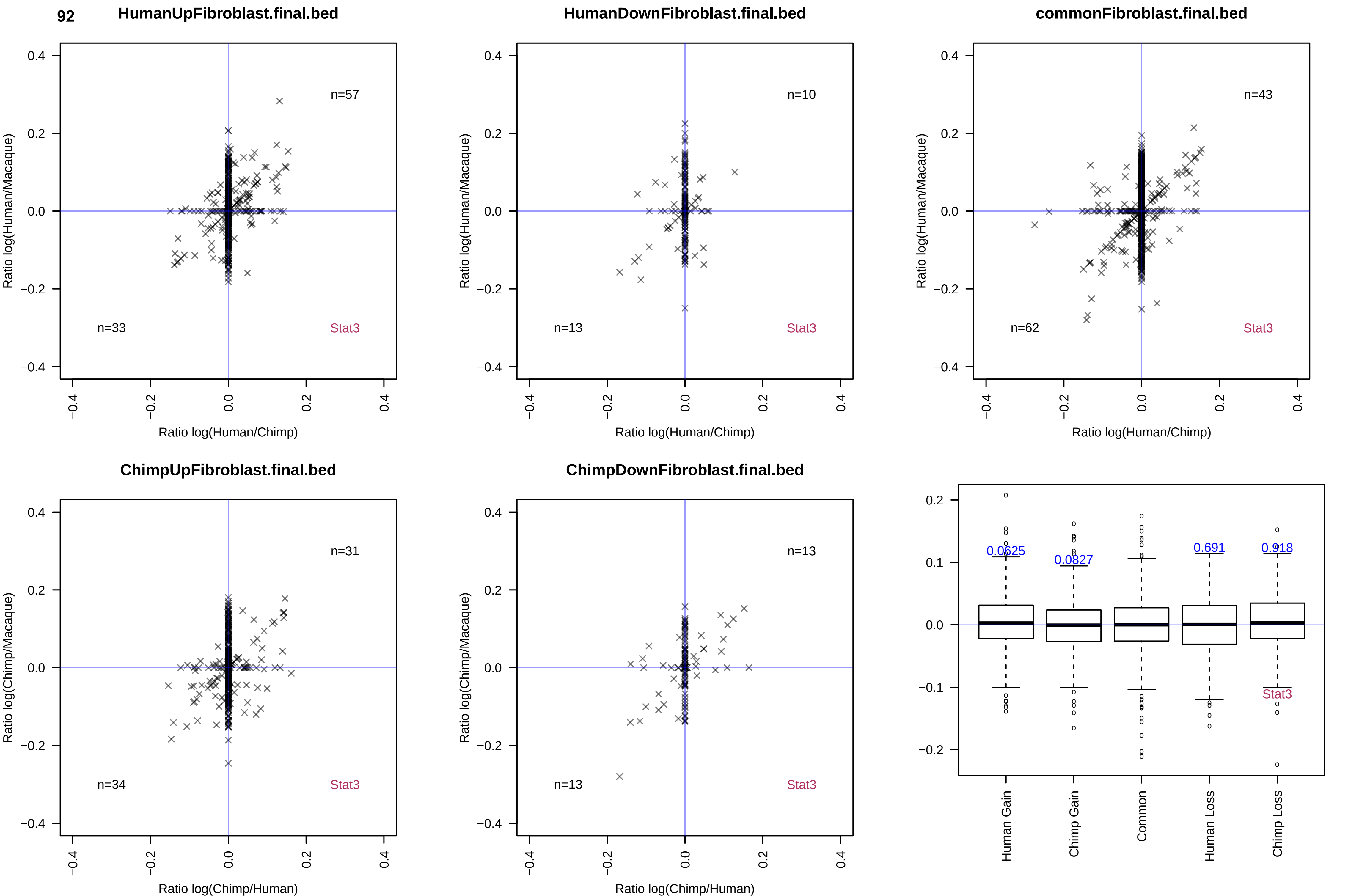
HumanUpFibroblast.final.bed
n=57
n=33
Stat3
Ratio log(Human/Macaque)
Ratio log(Human/Chimp)
HumanDownFibroblast.final.bed
n=10
n=13
Stat3
Ratio log(Human/Macaque)
Ratio log(Human/Chimp)
commonFibroblast.final.bed
n=43
n=62
Stat3
Ratio log(Human/Macaque)
Ratio log(Human/Chimp)
ChimpUpFibroblast.final.bed
n=31
n=34
Stat3
Ratio log(Chimp/Macaque)
Ratio log(Chimp/Human)
ChimpDownFibroblast.final.bed
n=13
n=13
Stat3
Ratio log(Chimp/Macaque)
Ratio log(Chimp/Human)
0.0625
0.0827
0.691
0.918
Stat3
Human Gain
Chimp Gain
Common
Human Loss
Chimp Loss

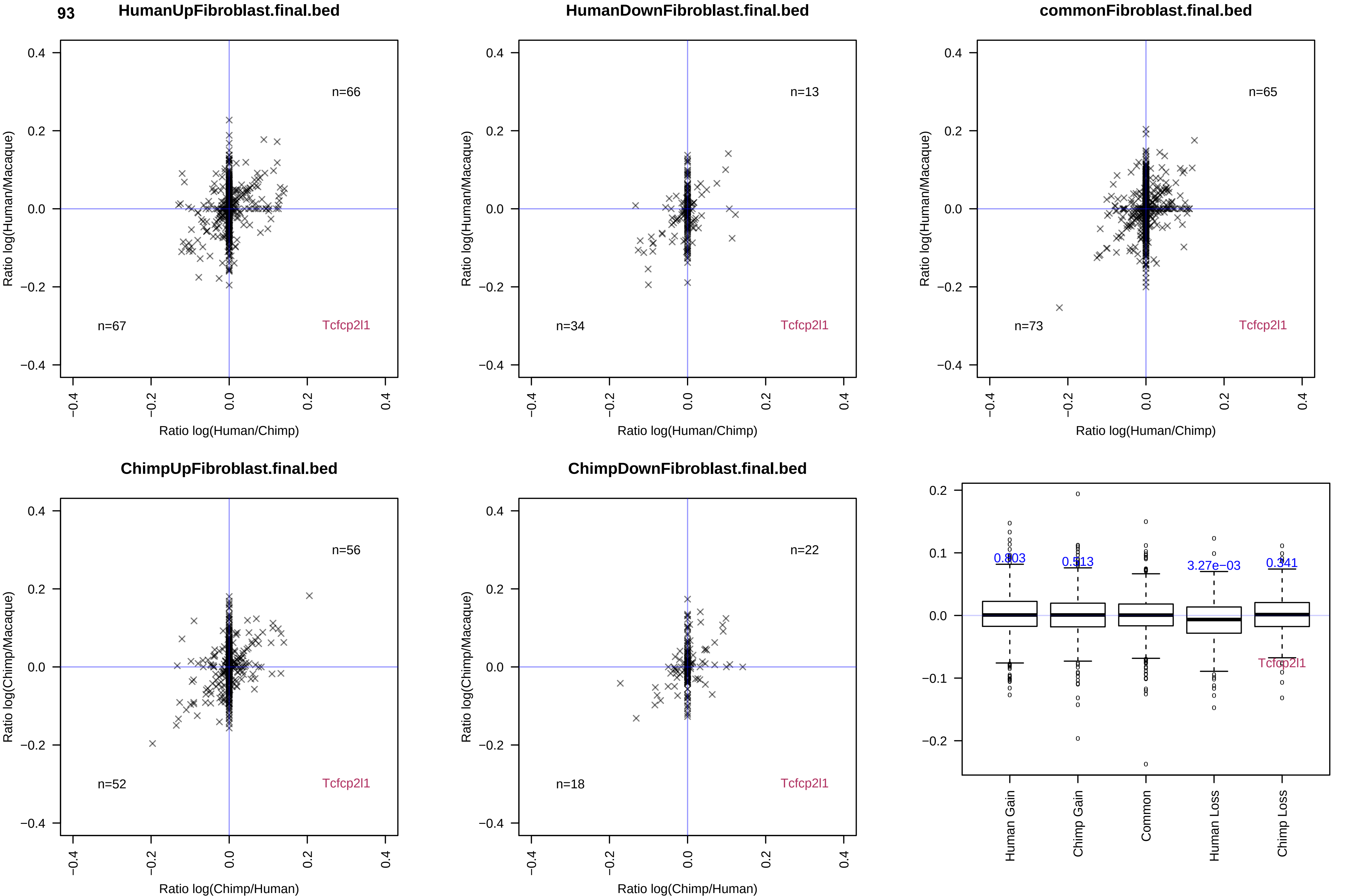
HumanUpFibroblast.final.bed
Ratio log(Human/Macaque)
Ratio log(Human/Chimp)
n=66
n=67
Tcfcp2l1
HumanDownFibroblast.final.bed
Ratio log(Human/Macaque)
Ratio log(Human/Chimp)
n=13
n=34
Tcfcp2l1
commonFibroblast.final.bed
Ratio log(Human/Macaque)
Ratio log(Human/Chimp)
n=65
n=73
Tcfcp2l1
ChimpUpFibroblast.final.bed
Ratio log(Chimp/Macaque)
Ratio log(Chimp/Human)
n=56
n=52
Tcfcp2l1
ChimpDownFibroblast.final.bed
Ratio log(Chimp/Macaque)
Ratio log(Chimp/Human)
n=22
n=18
Tcfcp2l1
0.803
0.513
3.27e−03
0.341
Tcfcp2l1
Human Gain
Chimp Gain
Common
Human Loss
Chimp Loss

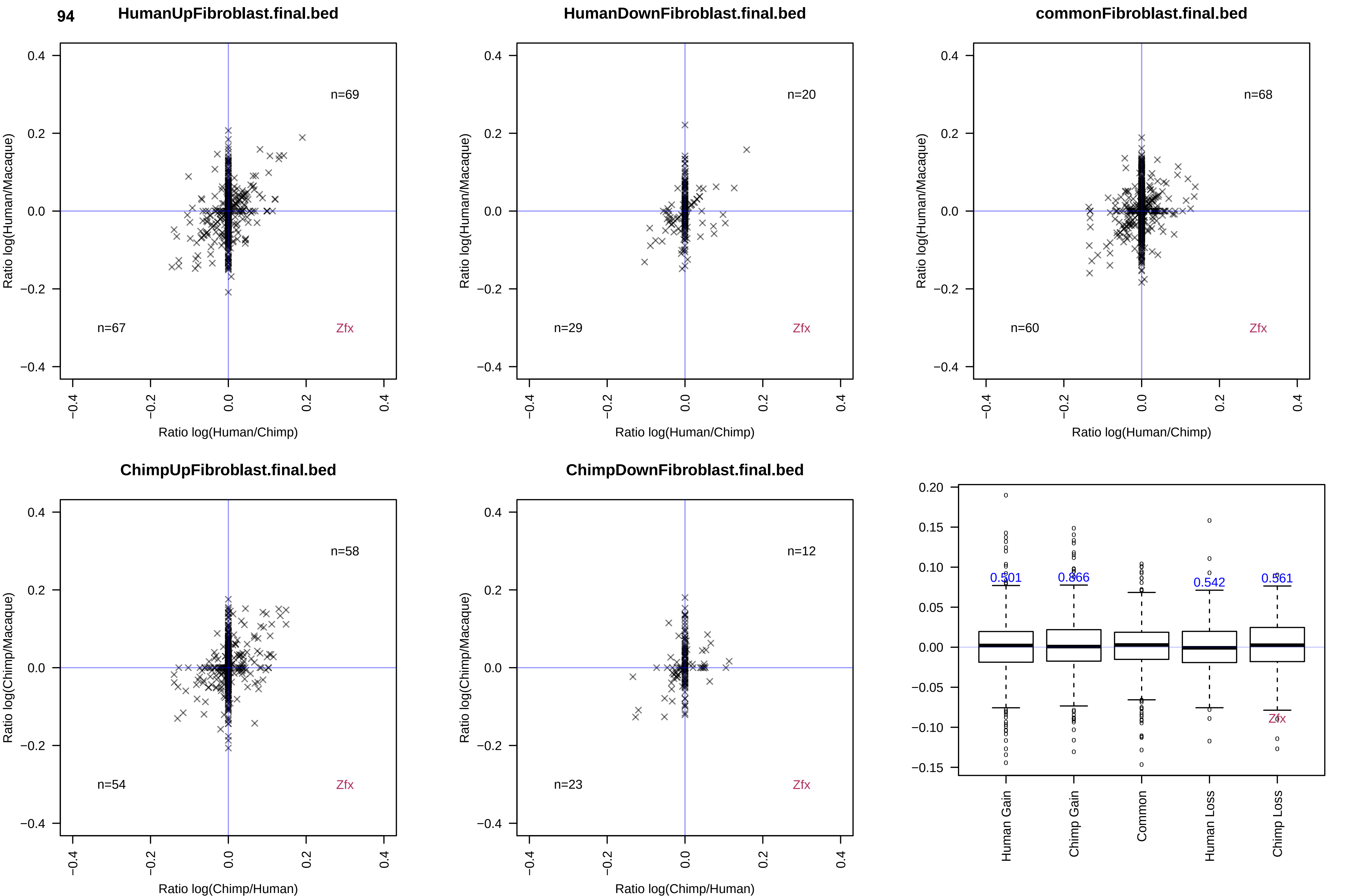
HumanUpFibroblast.final.bed
n=69
n=67
Zfx
Ratio log(Human/Macaque)
Ratio log(Human/Chimp)
HumanDownFibroblast.final.bed
n=20
n=29
Zfx
Ratio log(Human/Macaque)
Ratio log(Human/Chimp)
commonFibroblast.final.bed
n=68
n=60
Zfx
Ratio log(Human/Macaque)
Ratio log(Human/Chimp)
ChimpUpFibroblast.final.bed
n=58
n=54
Zfx
Ratio log(Chimp/Macaque)
Ratio log(Chimp/Human)
ChimpDownFibroblast.final.bed
n=12
n=23
Zfx
Ratio log(Chimp/Macaque)
Ratio log(Chimp/Human)
0.501
0.866
0.542
0.561
Zfx
Human Gain
Chimp Gain
Common
Human Loss
Chimp Loss

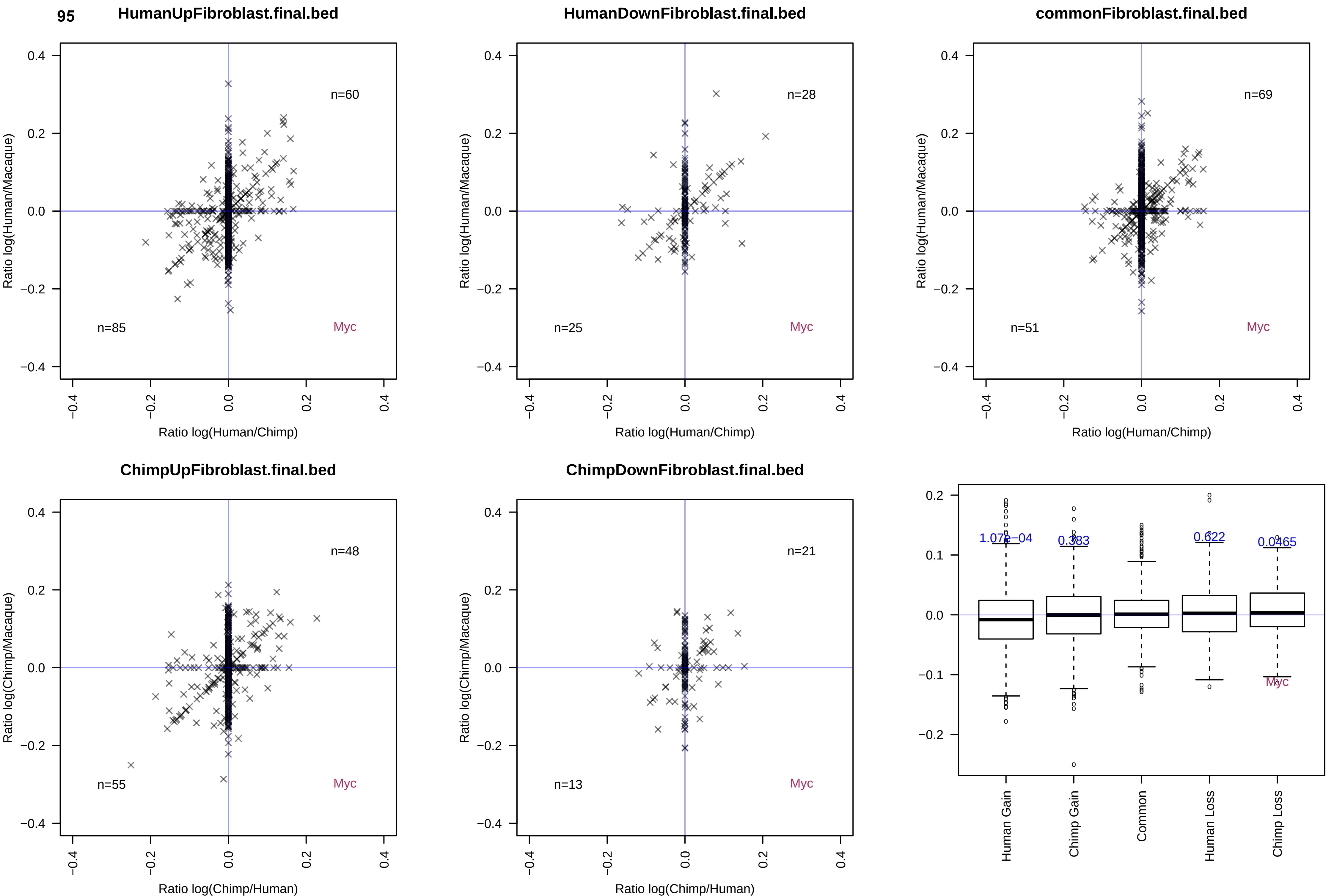

HumanUpFibroblast.final.bed
n=60
n=85
Myc
Ratio log(Human/Macaque)
Ratio log(Human/Chimp)
HumanDownFibroblast.final.bed
n=28
n=25
Myc
Ratio log(Human/Macaque)
Ratio log(Human/Chimp)
commonFibroblast.final.bed
n=69
n=51
Myc
Ratio log(Human/Macaque)
Ratio log(Human/Chimp)
ChimpUpFibroblast.final.bed
n=48
n=55
Myc
Ratio log(Chimp/Macaque)
Ratio log(Chimp/Human)
ChimpDownFibroblast.final.bed
n=21
n=13
Myc
Ratio log(Chimp/Macaque)
Ratio log(Chimp/Human)
1.07e−04
0.383
0.622
0.0465
Myc
Human Gain
Chimp Gain
Common
Human Loss
Chimp Loss

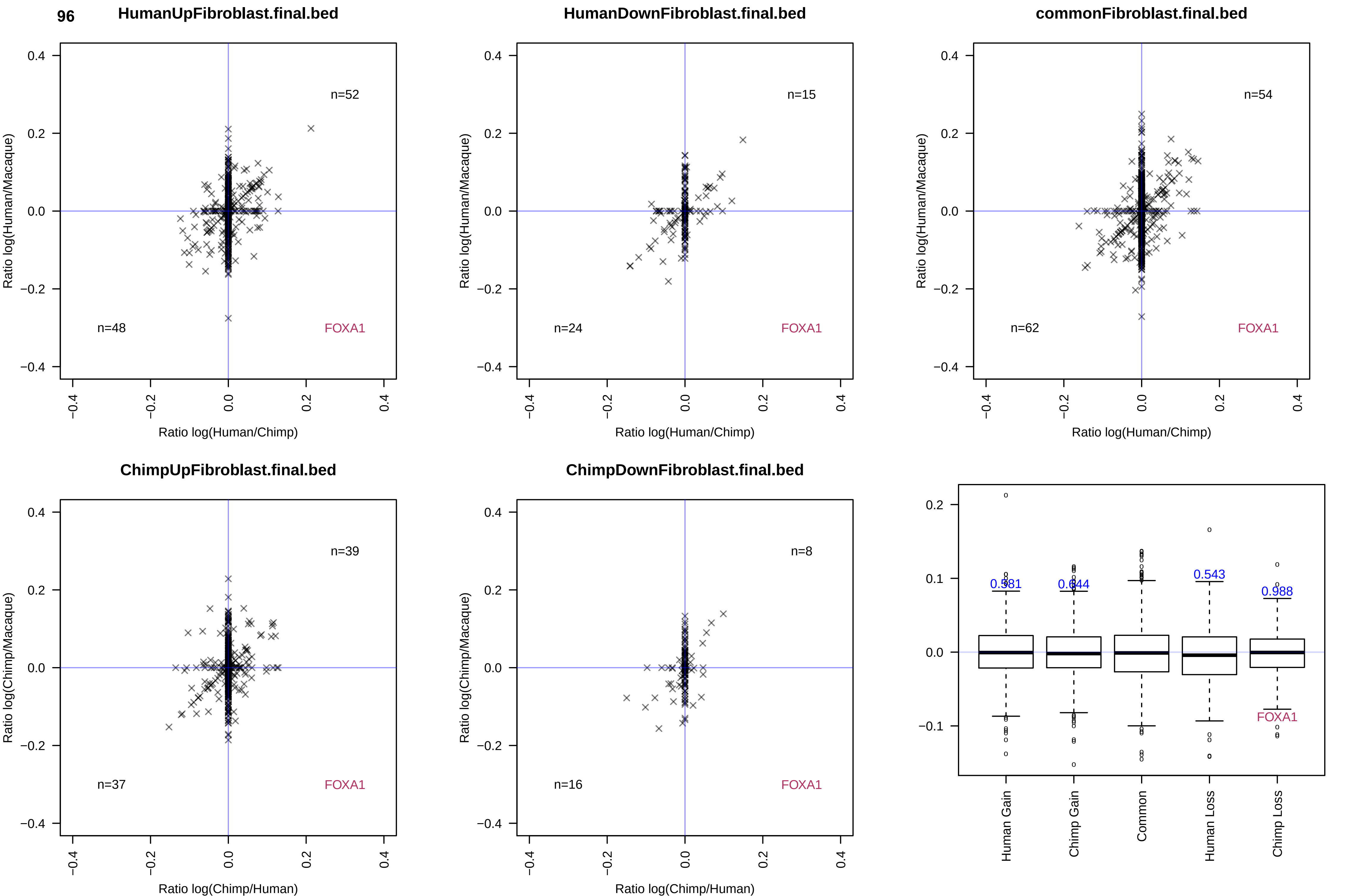
HumanUpFibroblast.final.bed
n=52
n=48
FOXA1
Ratio log(Human/Macaque)
Ratio log(Human/Chimp)
HumanDownFibroblast.final.bed
n=15
n=24
FOXA1
Ratio log(Human/Macaque)
Ratio log(Human/Chimp)
commonFibroblast.final.bed
n=54
n=62
FOXA1
Ratio log(Human/Macaque)
Ratio log(Human/Chimp)
ChimpUpFibroblast.final.bed
n=39
n=37
FOXA1
Ratio log(Chimp/Macaque)
Ratio log(Chimp/Human)
ChimpDownFibroblast.final.bed
n=8
n=16
FOXA1
Ratio log(Chimp/Macaque)
Ratio log(Chimp/Human)
0.581
0.644
0.543
0.988
FOXA1
Human Gain
Chimp Gain
Common
Human Loss
Chimp Loss

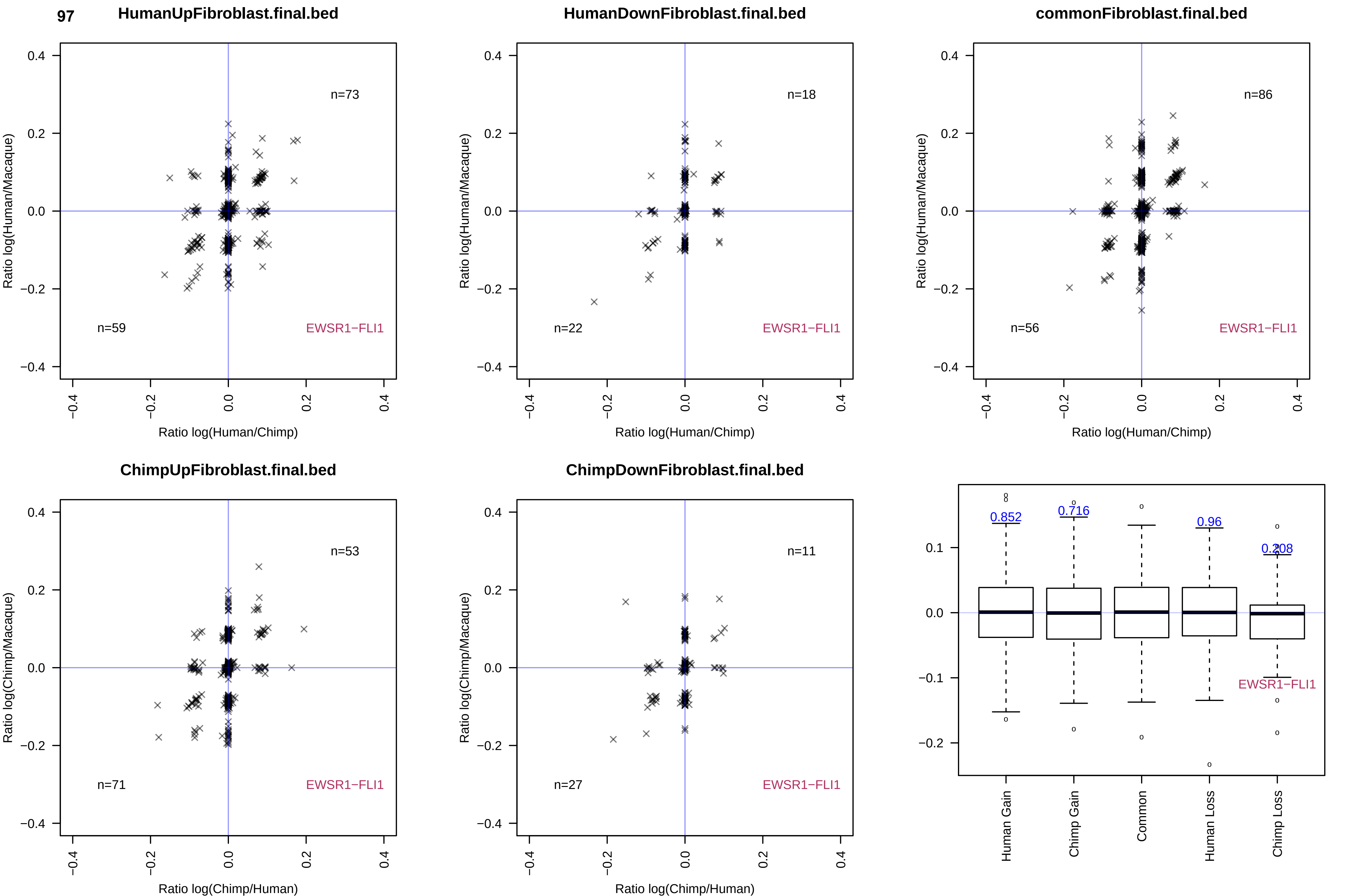
HumanUpFibroblast.final.bed
HumanDownFibroblast.final.bed
commonFibroblast.final.bed
ChimpUpFibroblast.final.bed
ChimpDownFibroblast.final.bed
Ratio log(Human/Macaque)
Ratio log(Human/Chimp)
Ratio log(Chimp/Macaque)
Ratio log(Chimp/Human)
n=73
n=59
n=18
n=22
n=86
n=56
n=53
n=71
n=11
n=27
EWSR1−FLI1
0.852
0.716
0.96
0.208
Human Gain
Chimp Gain
Common
Human Loss
Chimp Loss

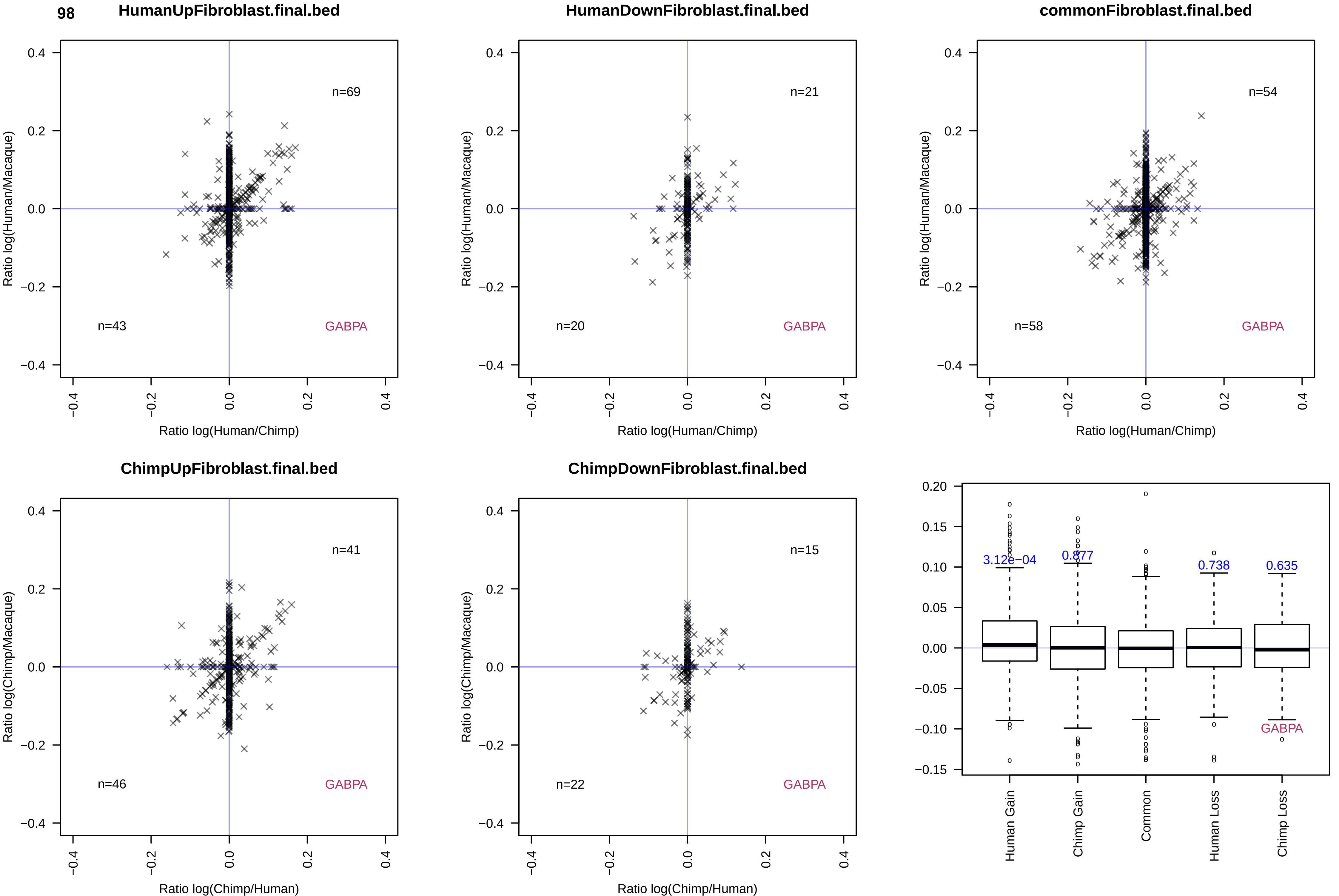
HumanUpFibroblast.final.bed
n=69
n=43
GABPA
Ratio log(Human/Chimp)
Ratio log(Human/Macaque)
HumanDownFibroblast.final.bed
n=21
n=20
GABPA
Ratio log(Human/Chimp)
Ratio log(Human/Macaque)
commonFibroblast.final.bed
n=54
n=58
GABPA
Ratio log(Human/Chimp)
Ratio log(Human/Macaque)
ChimpUpFibroblast.final.bed
n=41
n=46
GABPA
Ratio log(Chimp/Human)
Ratio log(Chimp/Macaque)
ChimpDownFibroblast.final.bed
n=15
n=22
GABPA
Ratio log(Chimp/Human)
Ratio log(Chimp/Macaque)
3.12e−04
0.877
0.738
0.635
GABPA
Human Gain
Chimp Gain
Common
Human Loss
Chimp Loss

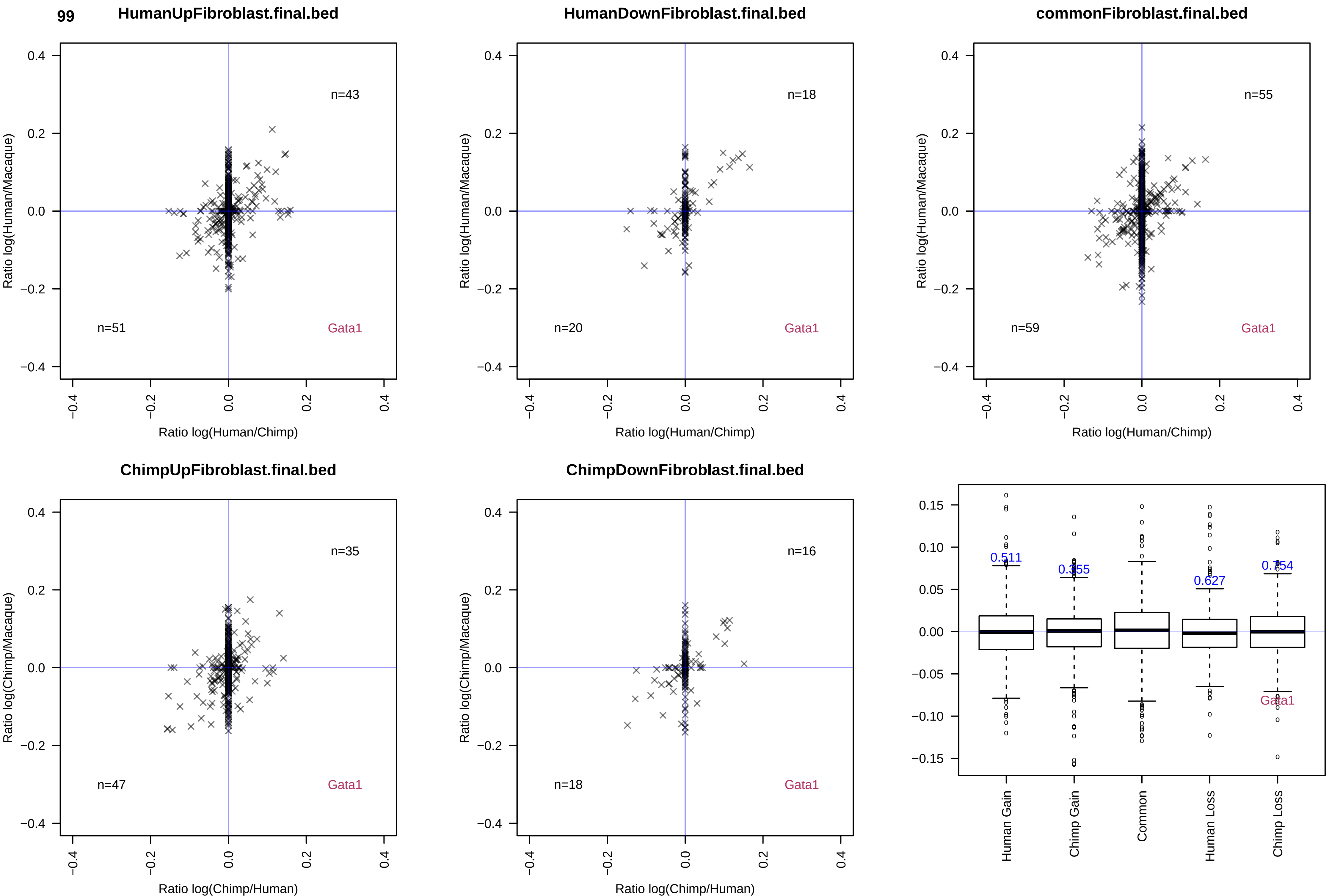

HumanUpFibroblast.final.bed
n=43
n=51
Gata1
Ratio log(Human/Macaque)
Ratio log(Human/Chimp)
HumanDownFibroblast.final.bed
n=18
n=20
Gata1
Ratio log(Human/Macaque)
Ratio log(Human/Chimp)
commonFibroblast.final.bed
n=55
n=59
Gata1
Ratio log(Human/Macaque)
Ratio log(Human/Chimp)
ChimpUpFibroblast.final.bed
n=35
n=47
Gata1
Ratio log(Chimp/Macaque)
Ratio log(Chimp/Human)
ChimpDownFibroblast.final.bed
n=16
n=18
Gata1
Ratio log(Chimp/Macaque)
Ratio log(Chimp/Human)
0.511
0.355
0.627
0.754
Gata1
Human Gain
Chimp Gain
Common
Human Loss
Chimp Loss

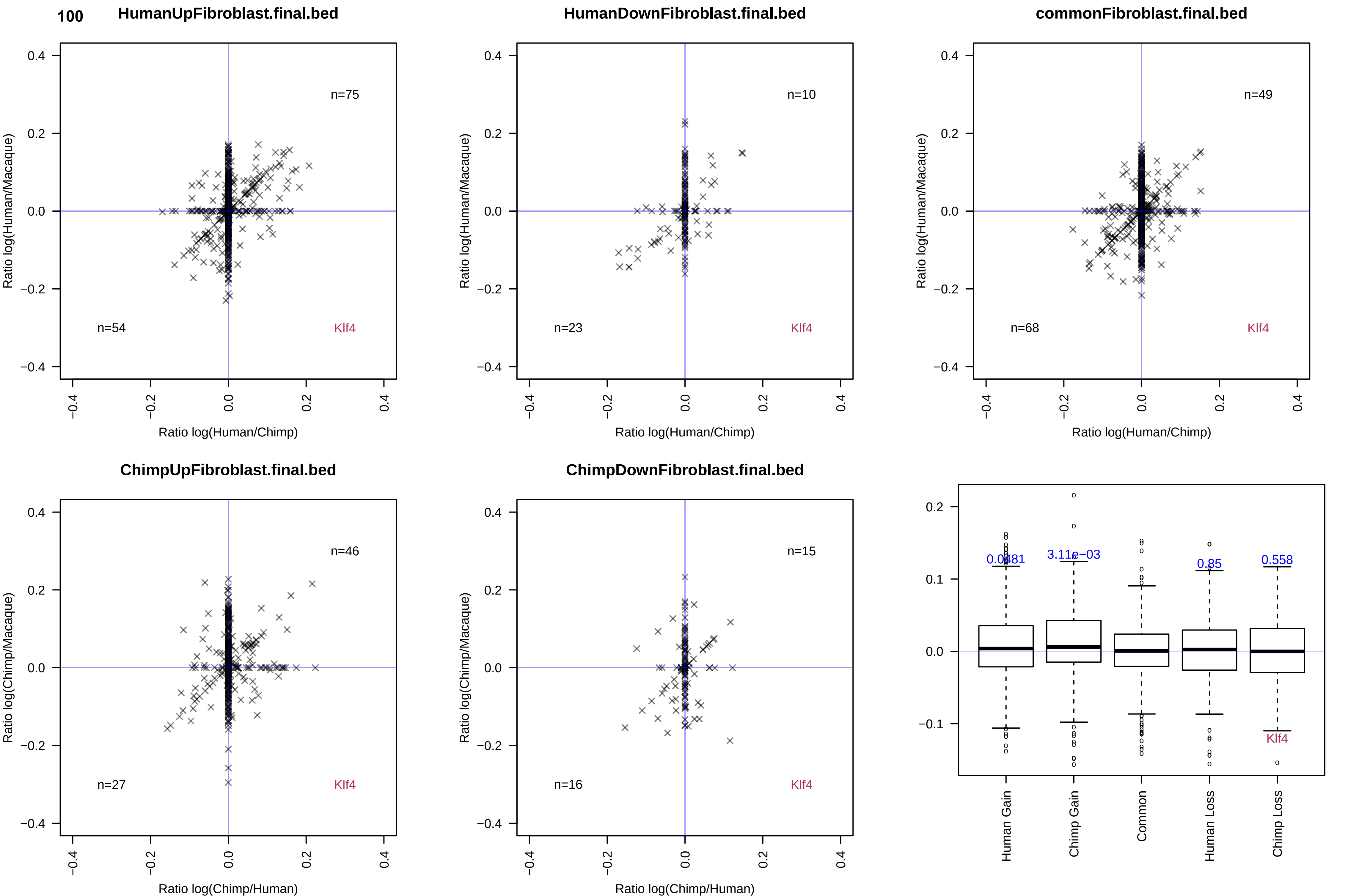
HumanUpFibroblast.final.bed
HumanDownFibroblast.final.bed
commonFibroblast.final.bed
ChimpUpFibroblast.final.bed
ChimpDownFibroblast.final.bed
n=75
n=54
n=10
n=23
n=49
n=68
n=46
n=27
n=15
n=16
Klf4
Ratio log(Human/Macaque)
Ratio log(Human/Chimp)
Ratio log(Chimp/Macaque)
Ratio log(Chimp/Human)
0.4
0.2
0.0
−0.2
−0.4
0.1
−0.1
0.0481
3.11e−03
0.85
0.558
Human Gain
Chimp Gain
Common
Human Loss
Chimp Loss

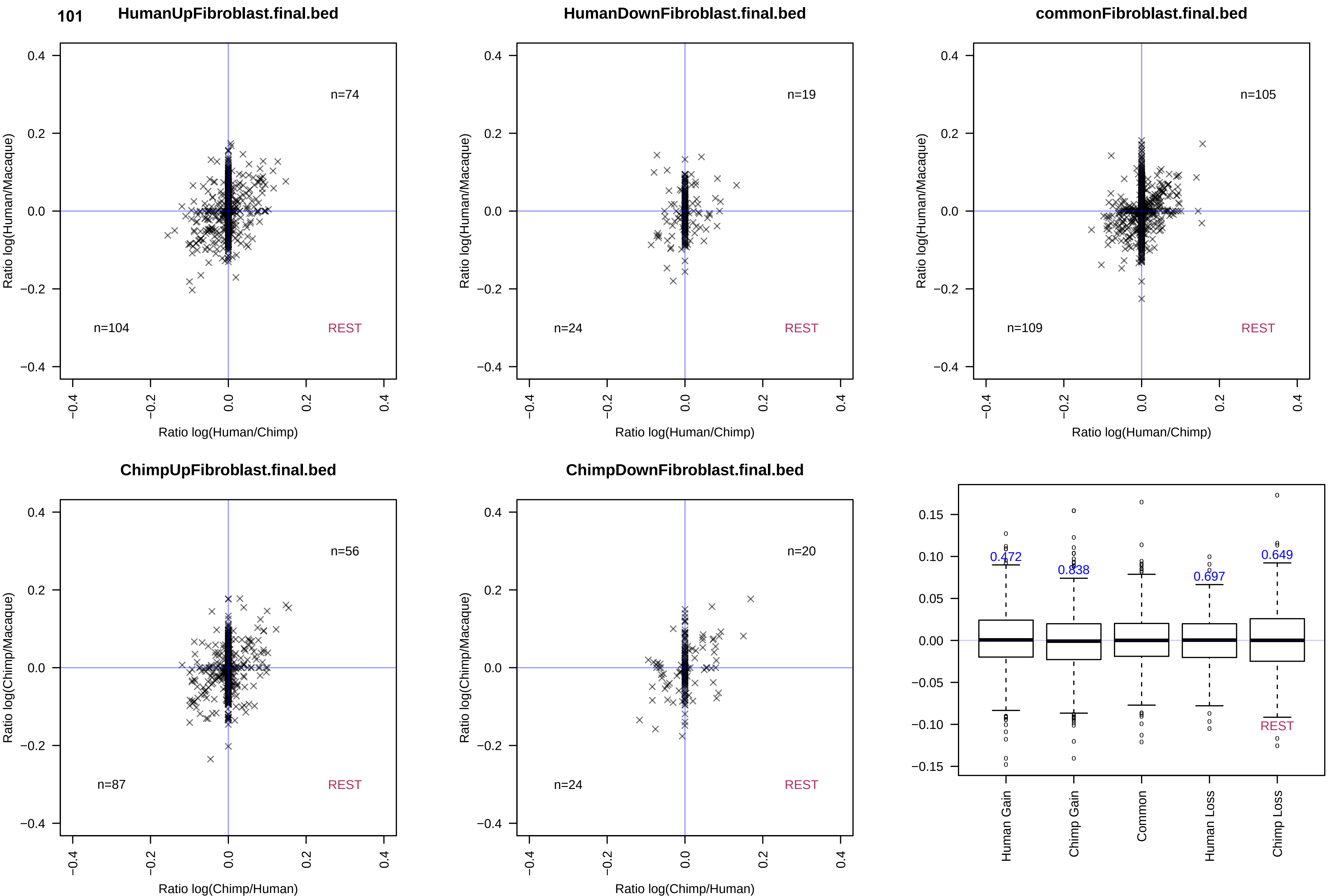
HumanUpFibroblast.final.bed
n=74
n=104
REST
HumanDownFibroblast.final.bed
n=19
n=24
REST
commonFibroblast.final.bed
n=105
n=109
REST
ChimpUpFibroblast.final.bed
n=56
n=87
REST
ChimpDownFibroblast.final.bed
n=20
n=24
REST
Ratio log(Human/Macaque)
Ratio log(Human/Chimp)
Ratio log(Chimp/Macaque)
Ratio log(Chimp/Human)
0.472
0.838
0.697
0.649
REST
Human Gain
Chimp Gain
Common
Human Loss
Chimp Loss

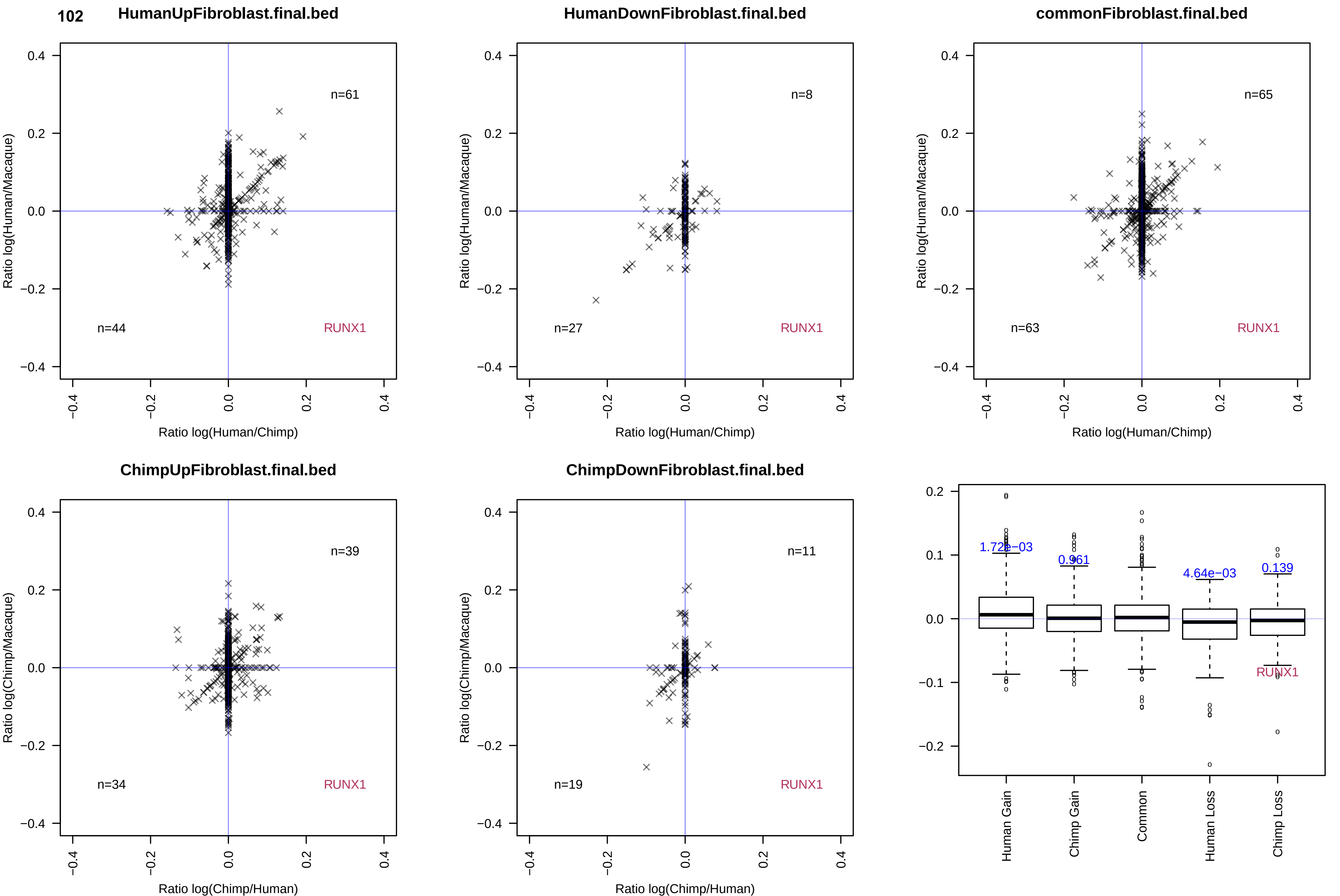
HumanUpFibroblast.final.bed
n=61
n=44
RUNX1
Ratio log(Human/Macaque)
Ratio log(Human/Chimp)
HumanDownFibroblast.final.bed
n=8
n=27
RUNX1
commonFibroblast.final.bed
n=65
n=63
RUNX1
ChimpUpFibroblast.final.bed
n=39
n=34
RUNX1
Ratio log(Chimp/Macaque)
Ratio log(Chimp/Human)
ChimpDownFibroblast.final.bed
n=11
n=19
RUNX1
1.72e−03
0.961
4.64e−03
0.139
RUNX1
Human Gain
Chimp Gain
Common
Human Loss
Chimp Loss

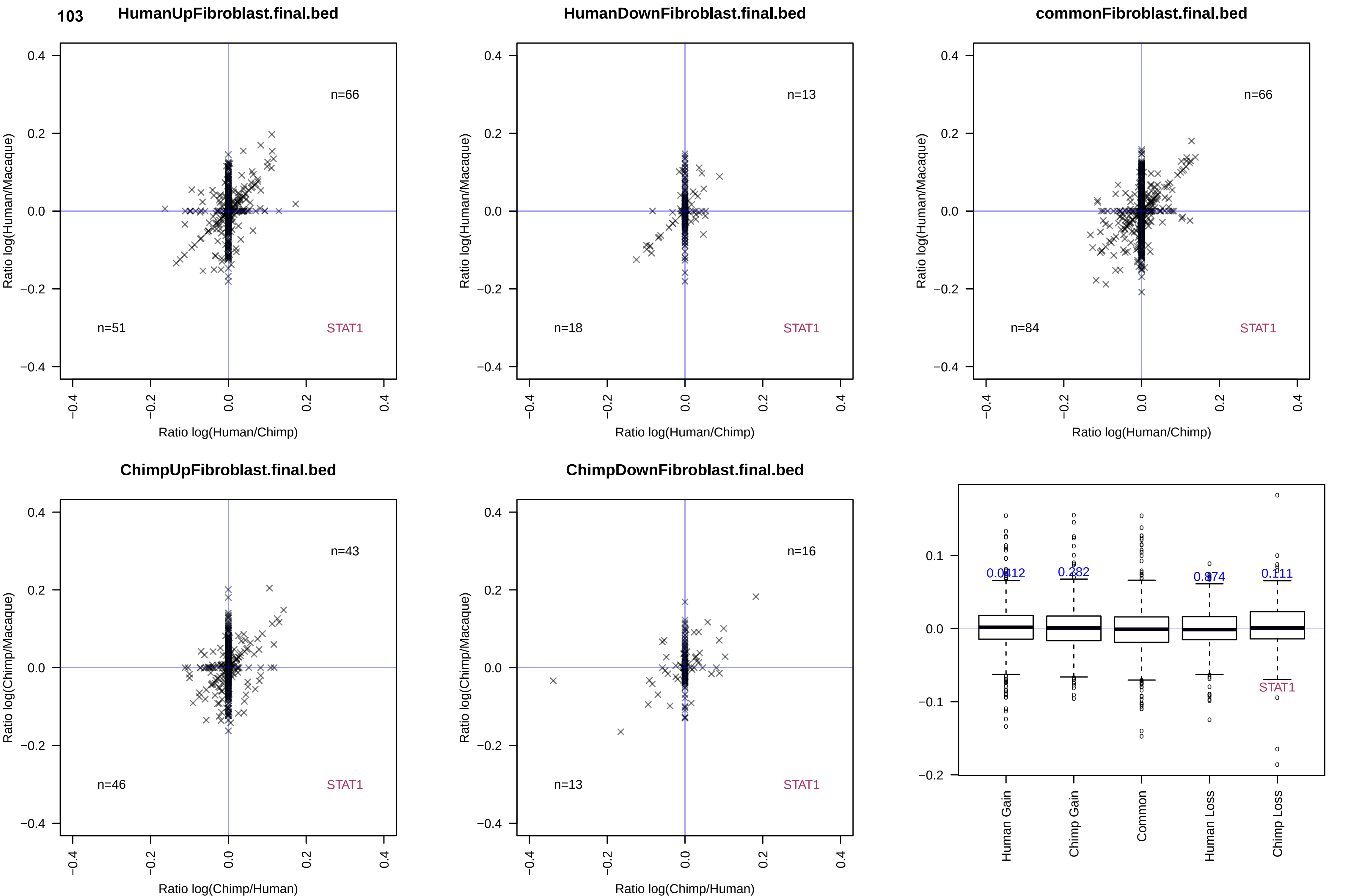
HumanUpFibroblast.final.bed
n=66
n=51
STAT1
HumanDownFibroblast.final.bed
n=13
n=18
STAT1
commonFibroblast.final.bed
n=66
n=84
STAT1
Ratio log(Human/Macaque)
Ratio log(Human/Chimp)
ChimpUpFibroblast.final.bed
n=43
n=46
STAT1
ChimpDownFibroblast.final.bed
n=16
n=13
STAT1
Ratio log(Chimp/Macaque)
Ratio log(Chimp/Human)
0.0412
0.282
0.874
0.111
STAT1
Human Gain
Chimp Gain
Common
Human Loss
Chimp Loss

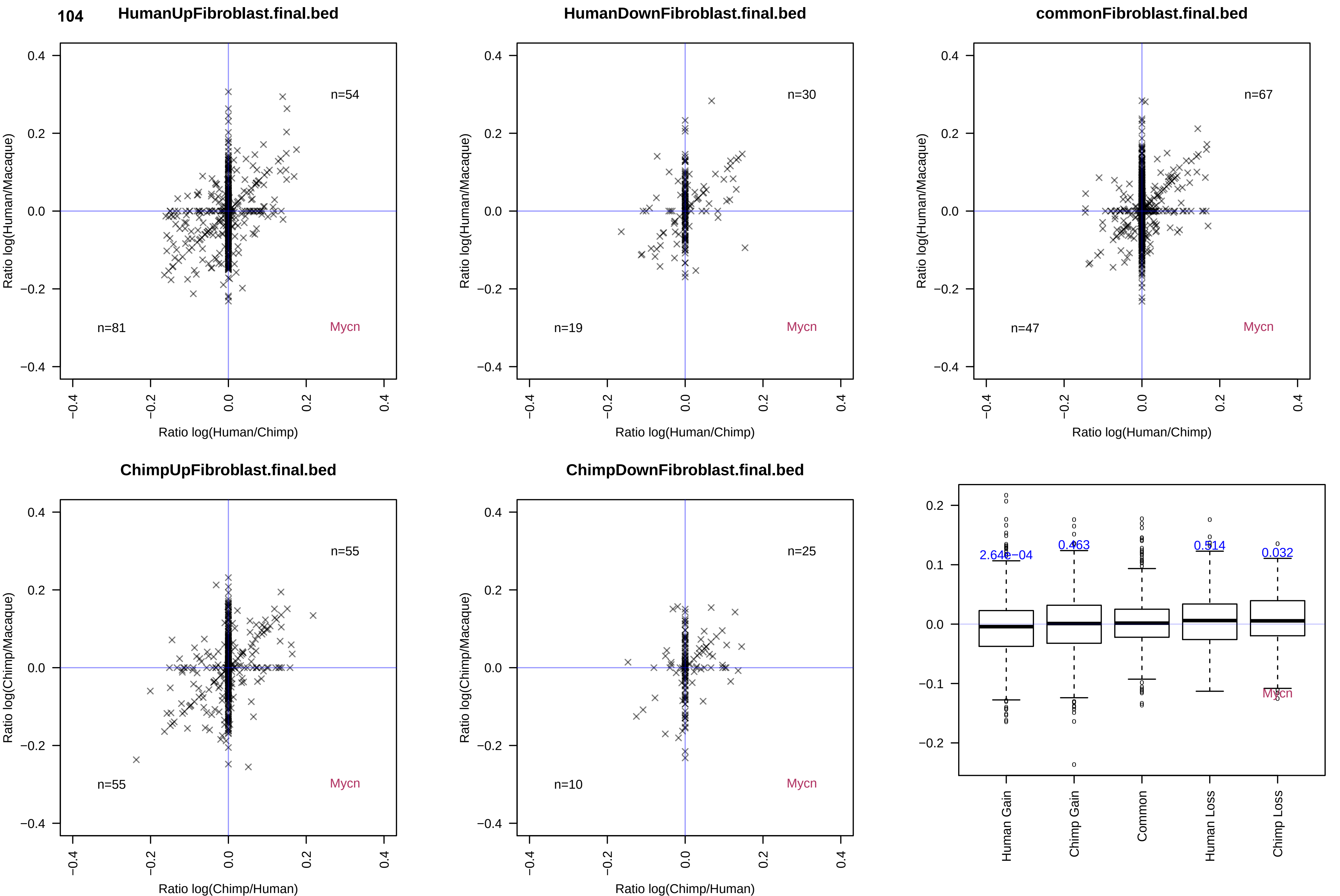
HumanUpFibroblast.final.bed
HumanDownFibroblast.final.bed
commonFibroblast.final.bed
ChimpUpFibroblast.final.bed
ChimpDownFibroblast.final.bed
Ratio log(Human/Macaque)
Ratio log(Human/Chimp)
Ratio log(Chimp/Macaque)
Ratio log(Chimp/Human)
n=54
n=81
n=30
n=19
n=67
n=47
n=55
n=55
n=25
n=10
Mycn
2.64e−04
0.463
0.514
0.032
Human Gain
Chimp Gain
Common
Human Loss
Chimp Loss

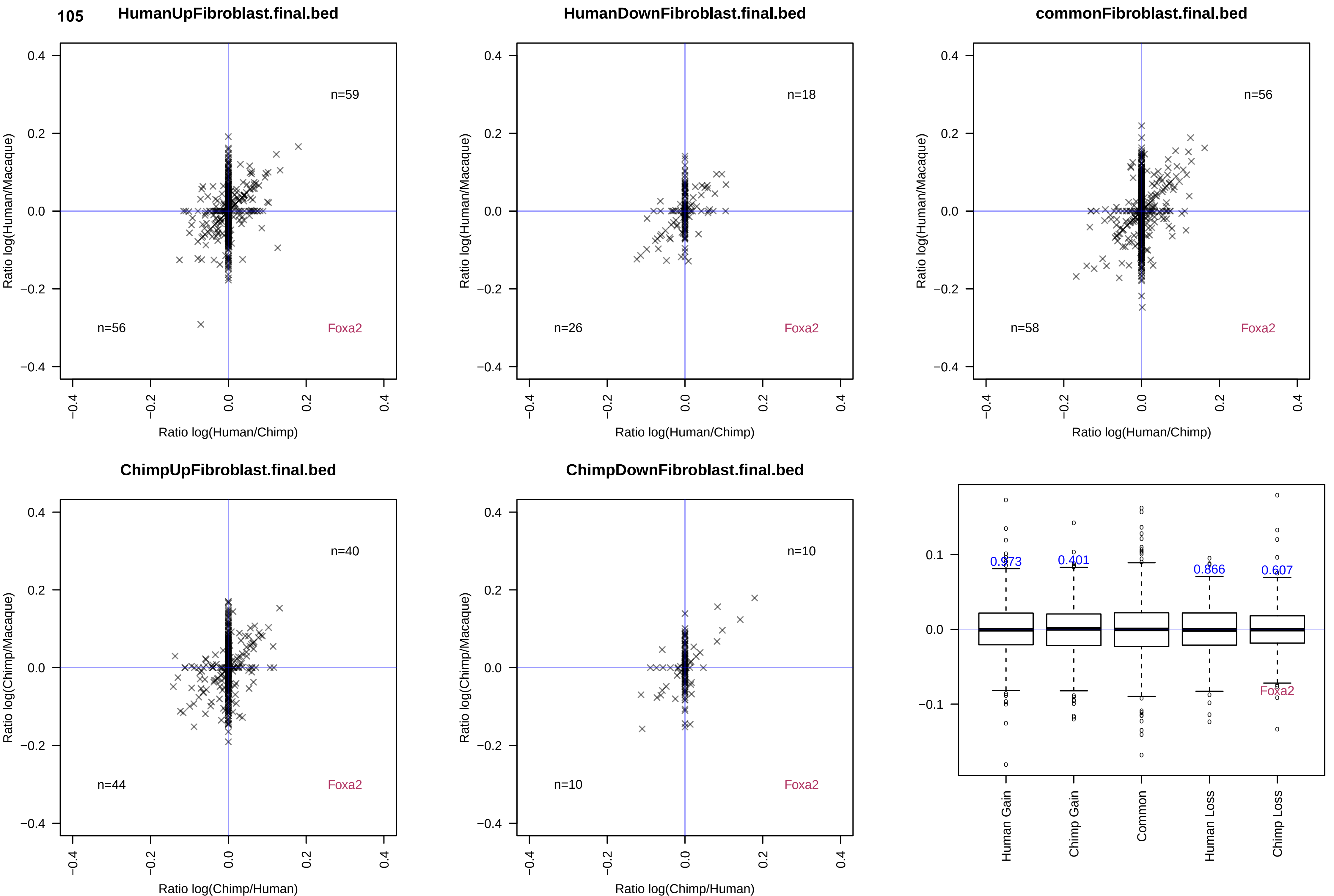
HumanUpFibroblast.final.bed
n=59
n=56
Foxa2
HumanDownFibroblast.final.bed
n=18
n=26
Foxa2
commonFibroblast.final.bed
n=56
n=58
Foxa2
Ratio log(Human/Macaque)
Ratio log(Human/Chimp)
ChimpUpFibroblast.final.bed
n=40
n=44
Foxa2
ChimpDownFibroblast.final.bed
n=10
n=10
Foxa2
Ratio log(Chimp/Macaque)
Ratio log(Chimp/Human)
0.973
0.401
0.866
0.607
Foxa2
Human Gain
Chimp Gain
Common
Human Loss
Chimp Loss

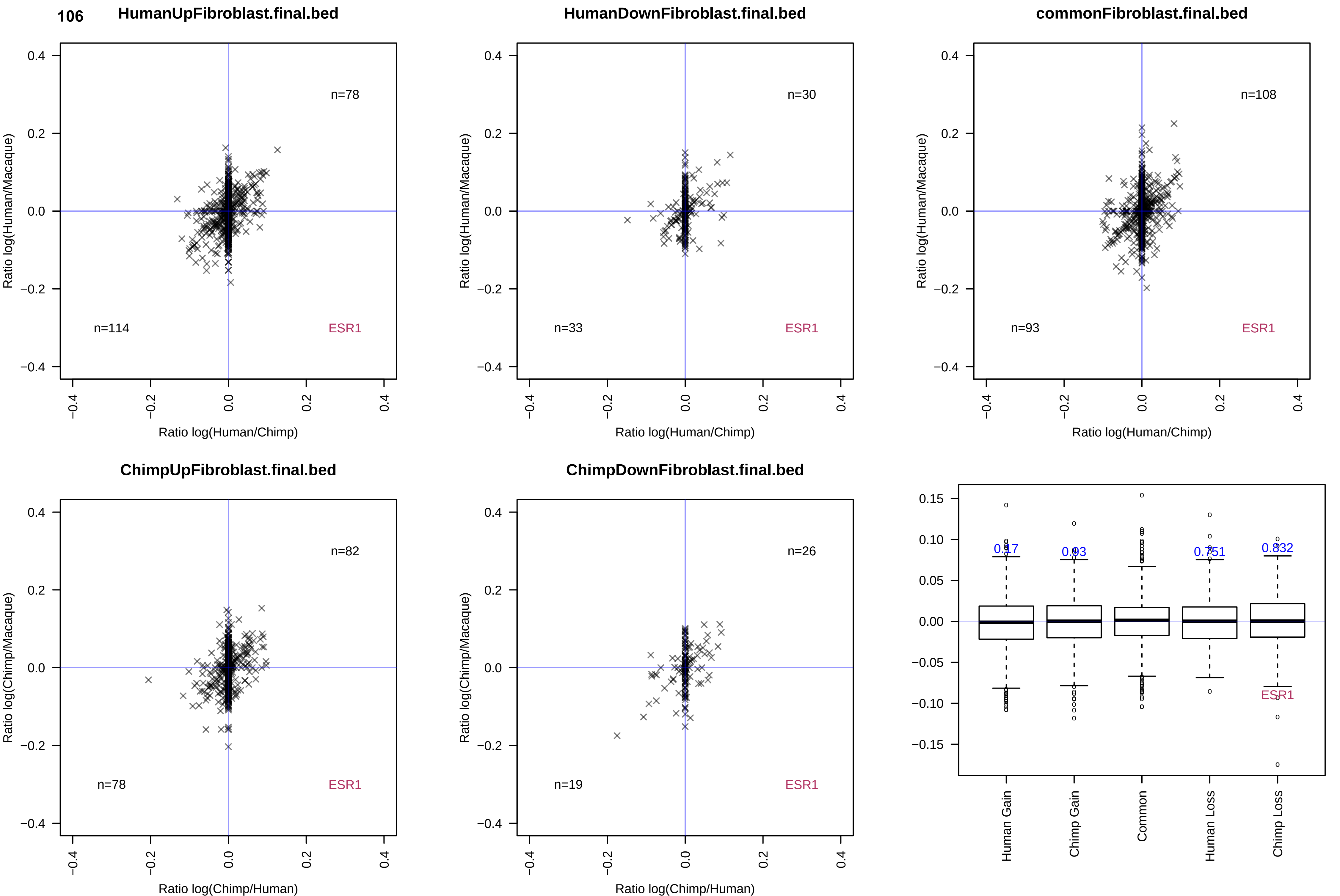
HumanUpFibroblast.final.bed
n=78
n=114
ESR1
Ratio log(Human/Macaque)
Ratio log(Human/Chimp)
HumanDownFibroblast.final.bed
n=30
n=33
ESR1
Ratio log(Human/Macaque)
Ratio log(Human/Chimp)
commonFibroblast.final.bed
n=108
n=93
ESR1
Ratio log(Human/Macaque)
Ratio log(Human/Chimp)
ChimpUpFibroblast.final.bed
n=82
n=78
ESR1
Ratio log(Chimp/Macaque)
Ratio log(Chimp/Human)
ChimpDownFibroblast.final.bed
n=26
n=19
ESR1
Ratio log(Chimp/Macaque)
Ratio log(Chimp/Human)
0.17
0.93
0.751
0.832
ESR1
Human Gain
Chimp Gain
Common
Human Loss
Chimp Loss

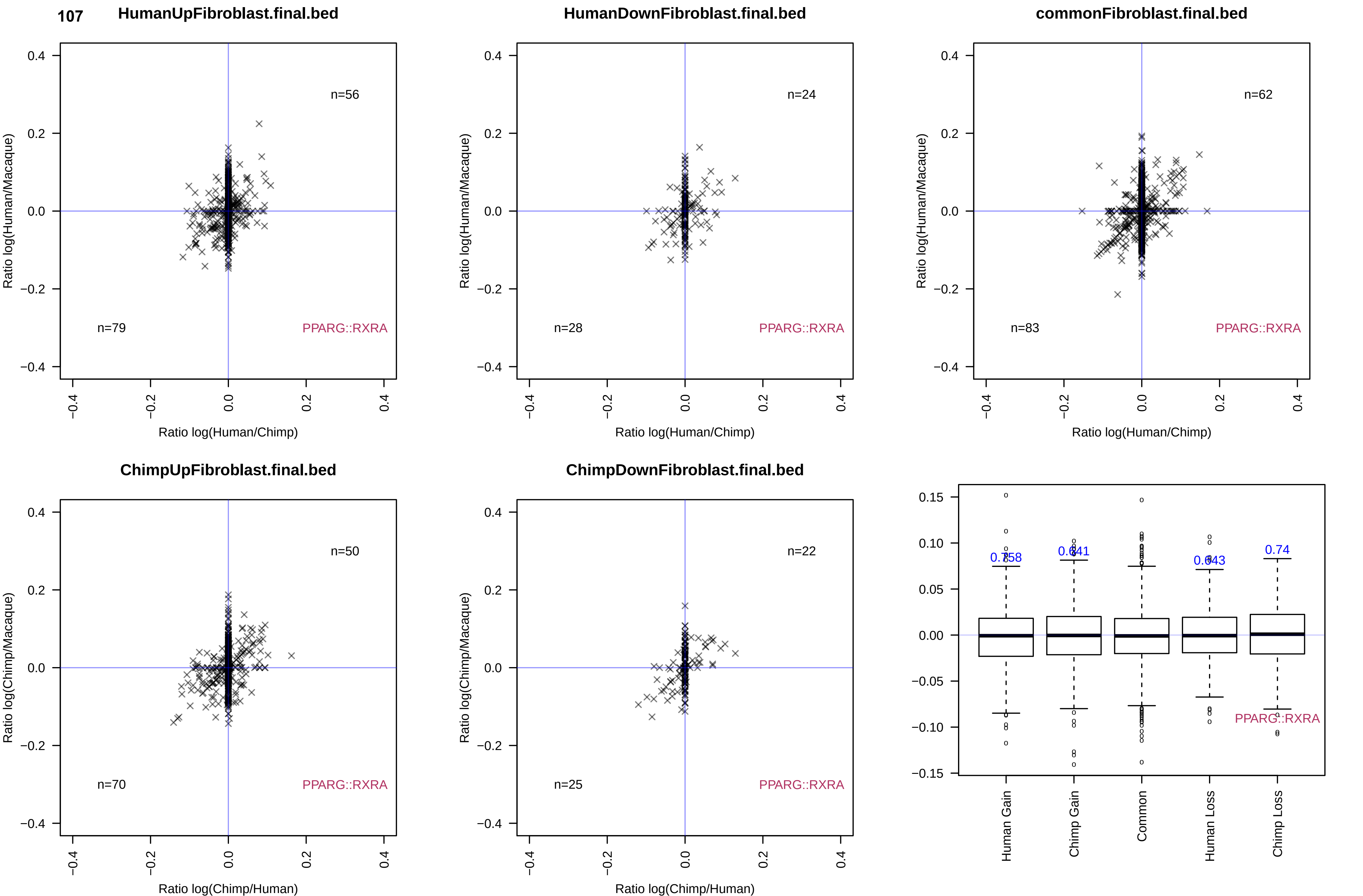
HumanUpFibroblast.final.bed
n=56
n=79
PPARG::RXRA
HumanDownFibroblast.final.bed
n=24
n=28
PPARG::RXRA
commonFibroblast.final.bed
n=62
n=83
PPARG::RXRA
Ratio log(Human/Macaque)
Ratio log(Human/Chimp)
ChimpUpFibroblast.final.bed
n=50
n=70
PPARG::RXRA
ChimpDownFibroblast.final.bed
n=22
n=25
PPARG::RXRA
Ratio log(Chimp/Macaque)
Ratio log(Chimp/Human)
0.758
0.641
0.643
0.74
PPARG::RXRA
Human Gain
Chimp Gain
Common
Human Loss
Chimp Loss

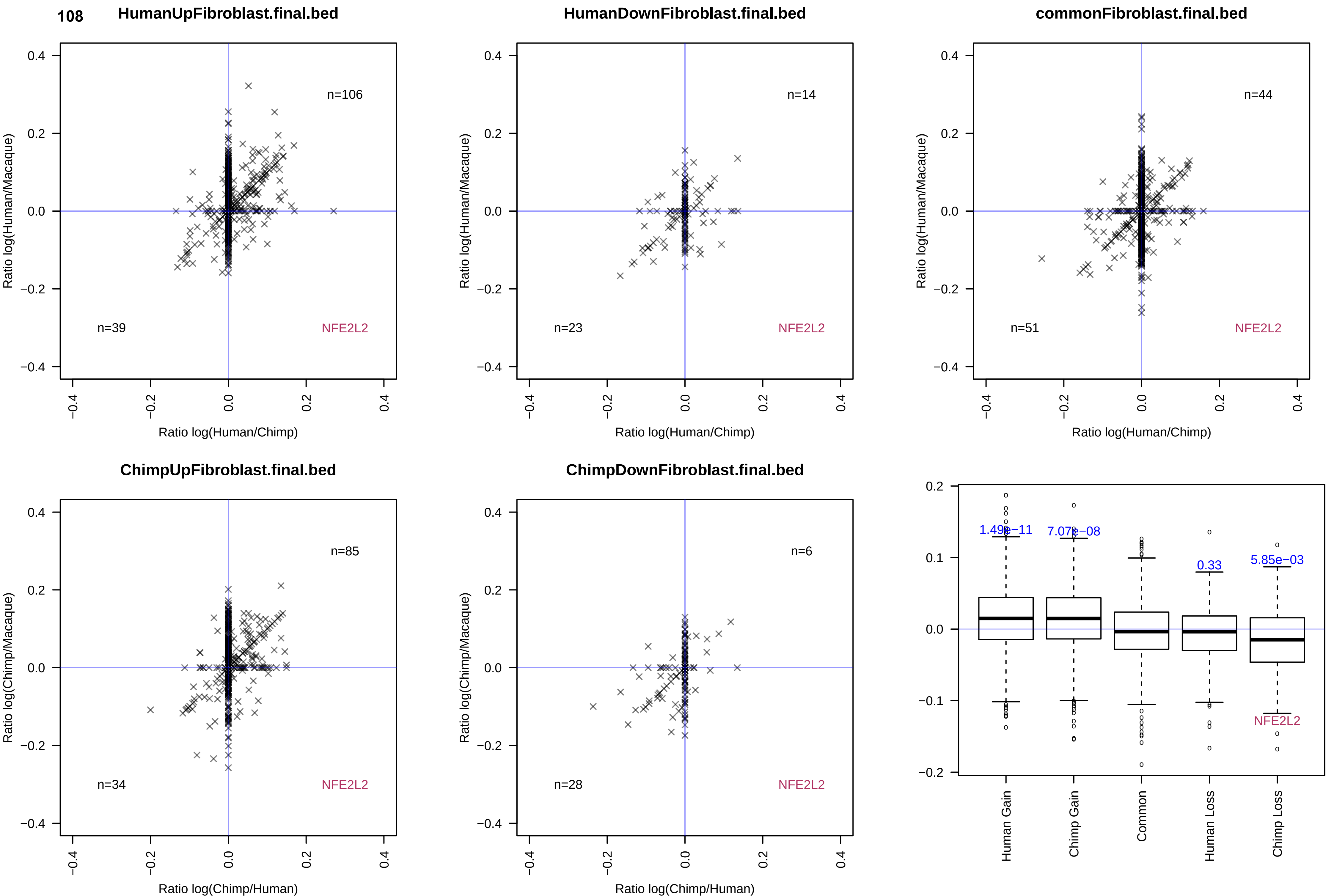
HumanUpFibroblast.final.bed
HumanDownFibroblast.final.bed
commonFibroblast.final.bed
ChimpUpFibroblast.final.bed
ChimpDownFibroblast.final.bed
n=106
n=39
n=14
n=23
n=44
n=51
n=85
n=34
n=6
n=28
NFE2L2
Ratio log(Human/Macaque)
Ratio log(Human/Chimp)
Ratio log(Chimp/Macaque)
Ratio log(Chimp/Human)
1.49e−11
7.07e−08
0.33
5.85e−03
Human Gain
Chimp Gain
Common
Human Loss
Chimp Loss

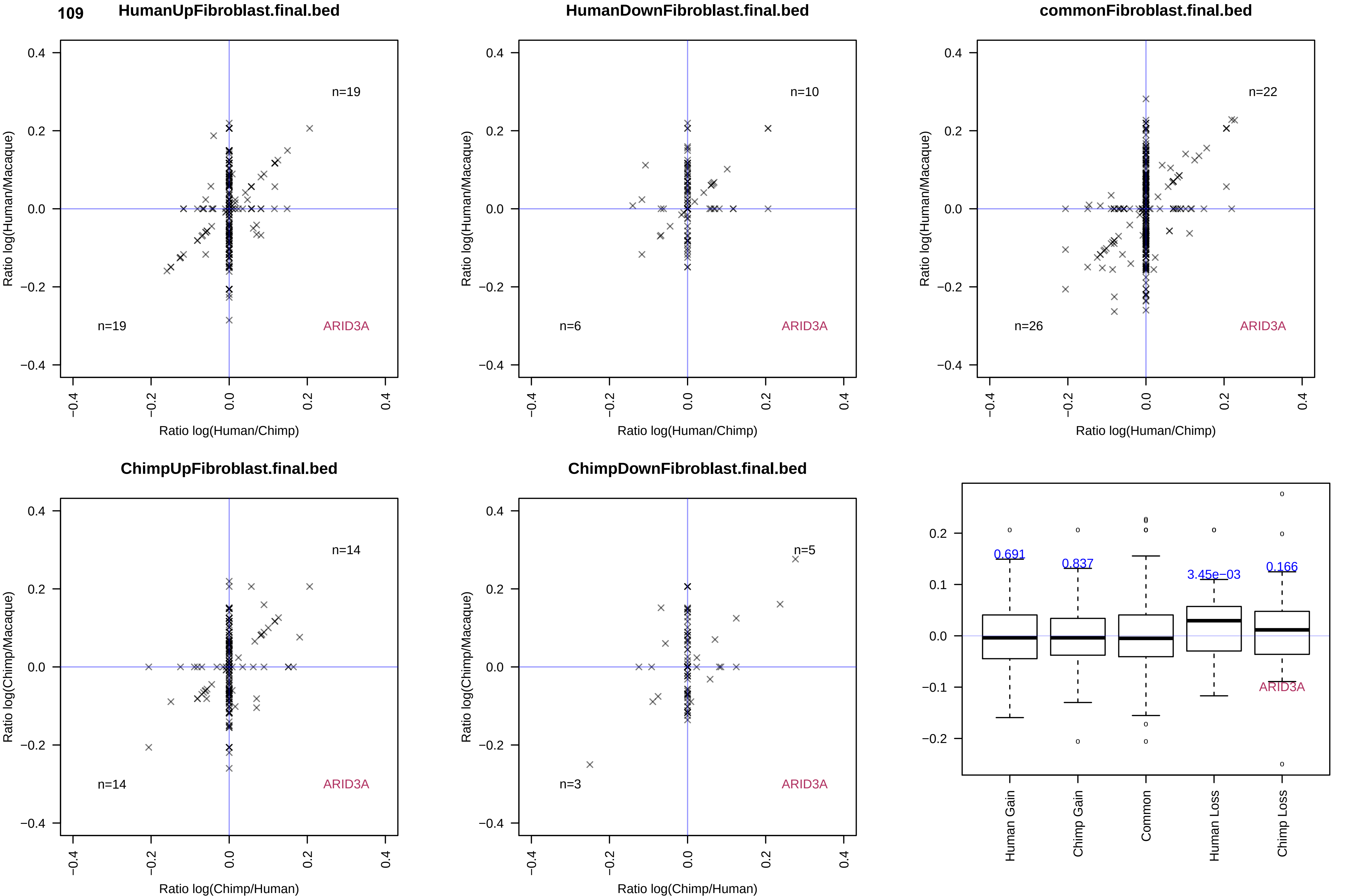
HumanUpFibroblast.final.bed
n=19
n=19
ARID3A
Ratio log(Human/Chimp)
Ratio log(Human/Macaque)
HumanDownFibroblast.final.bed
n=10
n=6
ARID3A
Ratio log(Human/Chimp)
Ratio log(Human/Macaque)
commonFibroblast.final.bed
n=22
n=26
ARID3A
Ratio log(Human/Chimp)
Ratio log(Human/Macaque)
ChimpUpFibroblast.final.bed
n=14
n=14
ARID3A
Ratio log(Chimp/Human)
Ratio log(Chimp/Macaque)
ChimpDownFibroblast.final.bed
n=5
n=3
ARID3A
Ratio log(Chimp/Human)
Ratio log(Chimp/Macaque)
0.691
0.837
3.45e−03
0.166
ARID3A
Human Gain
Chimp Gain
Common
Human Loss
Chimp Loss

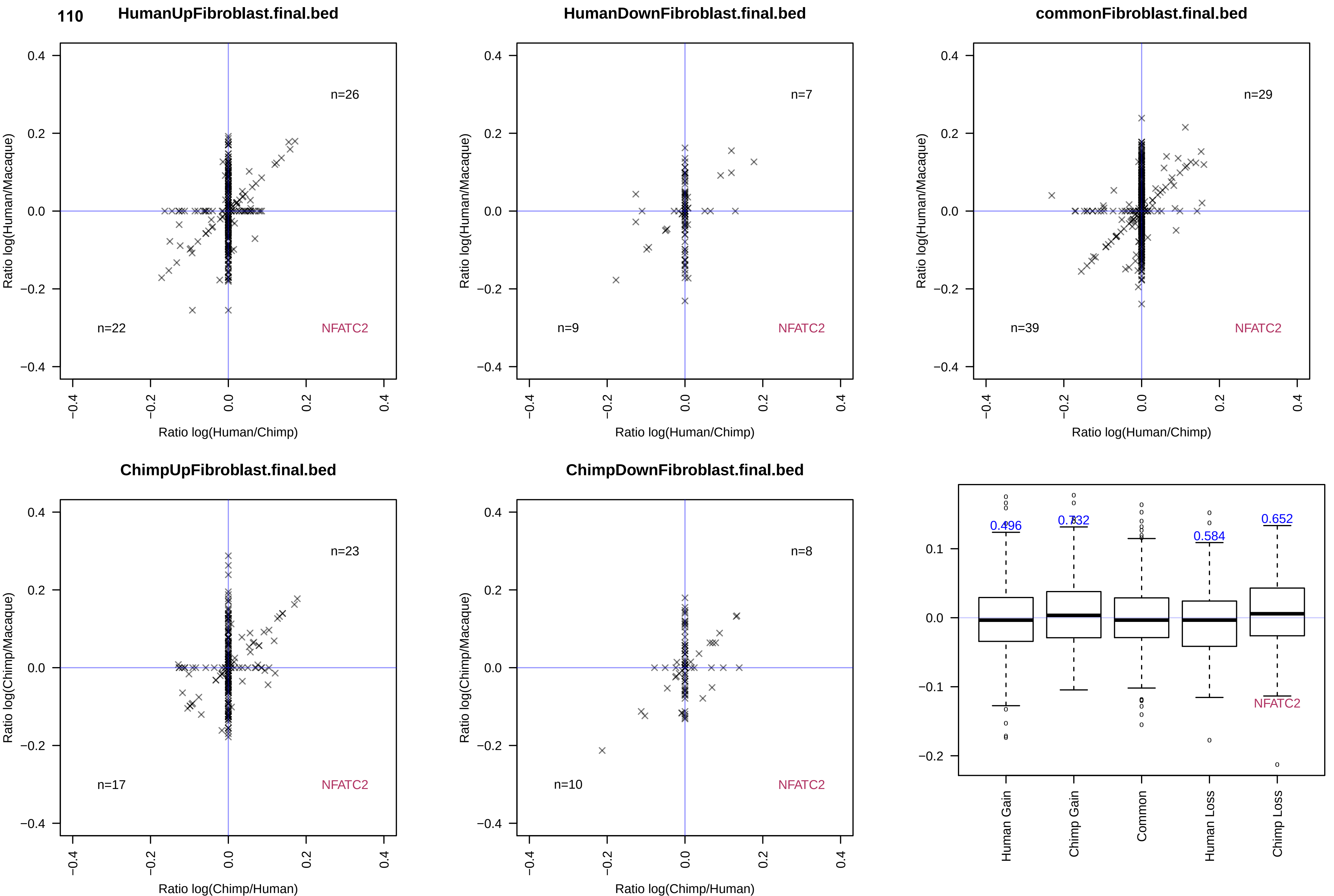
HumanUpFibroblast.final.bed
HumanDownFibroblast.final.bed
commonFibroblast.final.bed
ChimpUpFibroblast.final.bed
ChimpDownFibroblast.final.bed
Ratio log(Human/Macaque)
Ratio log(Human/Chimp)
Ratio log(Chimp/Macaque)
Ratio log(Chimp/Human)
n=26
n=22
n=7
n=9
n=29
n=39
n=23
n=17
n=8
n=10
NFATC2
0.496
0.732
0.584
0.652
Human Gain
Chimp Gain
Common
Human Loss
Chimp Loss

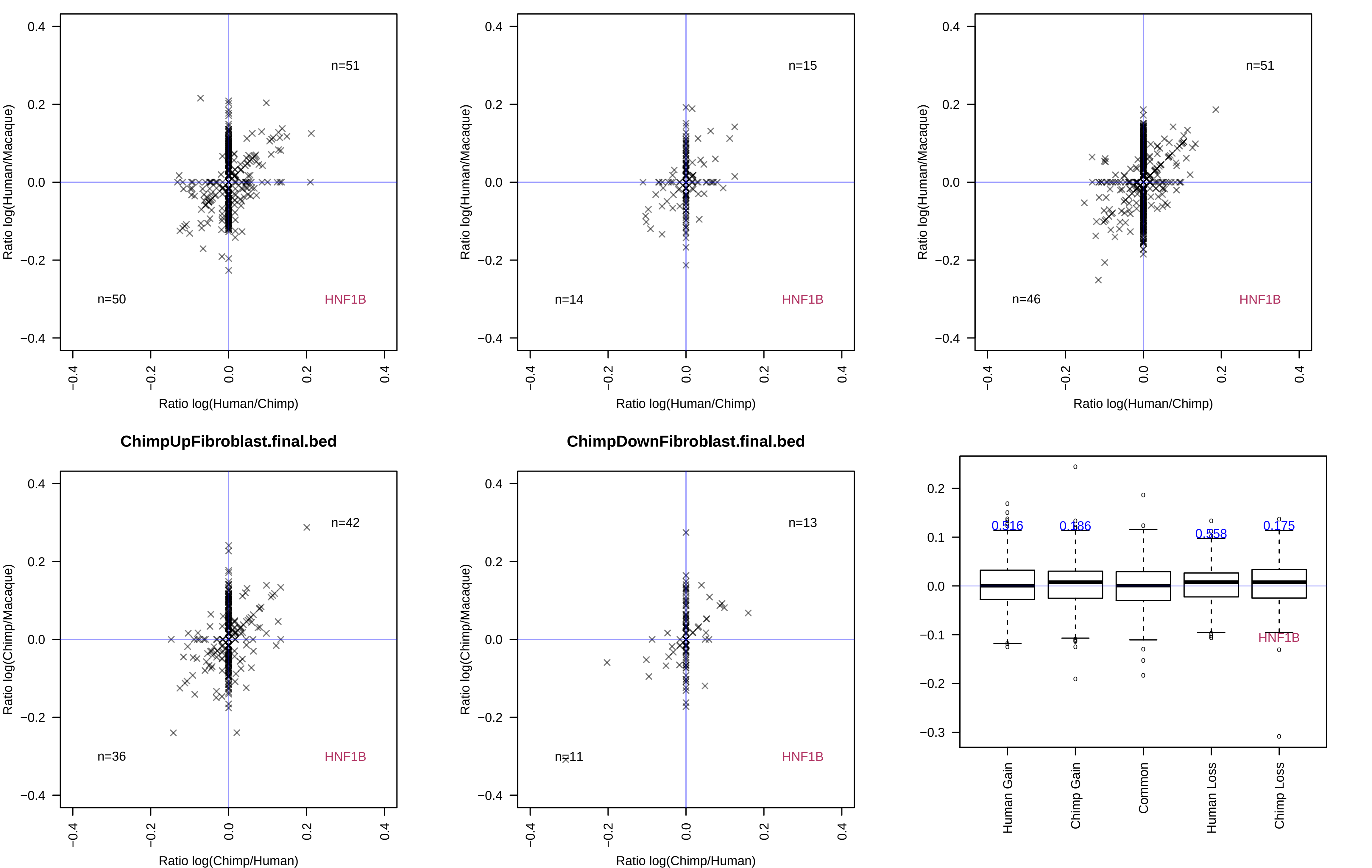
HumanUpFibroblast.final.bed
n=51
n=50
HNF1B
HumanDownFibroblast.final.bed
n=15
n=14
HNF1B
commonFibroblast.final.bed
n=51
n=46
HNF1B
Ratio log(Human/Macaque)
Ratio log(Human/Chimp)
ChimpUpFibroblast.final.bed
n=42
n=36
HNF1B
ChimpDownFibroblast.final.bed
n=13
n=11
HNF1B
Ratio log(Chimp/Macaque)
Ratio log(Chimp/Human)
0.516
0.186
0.558
0.175
HNF1B
Human Gain
Chimp Gain
Common
Human Loss
Chimp Loss

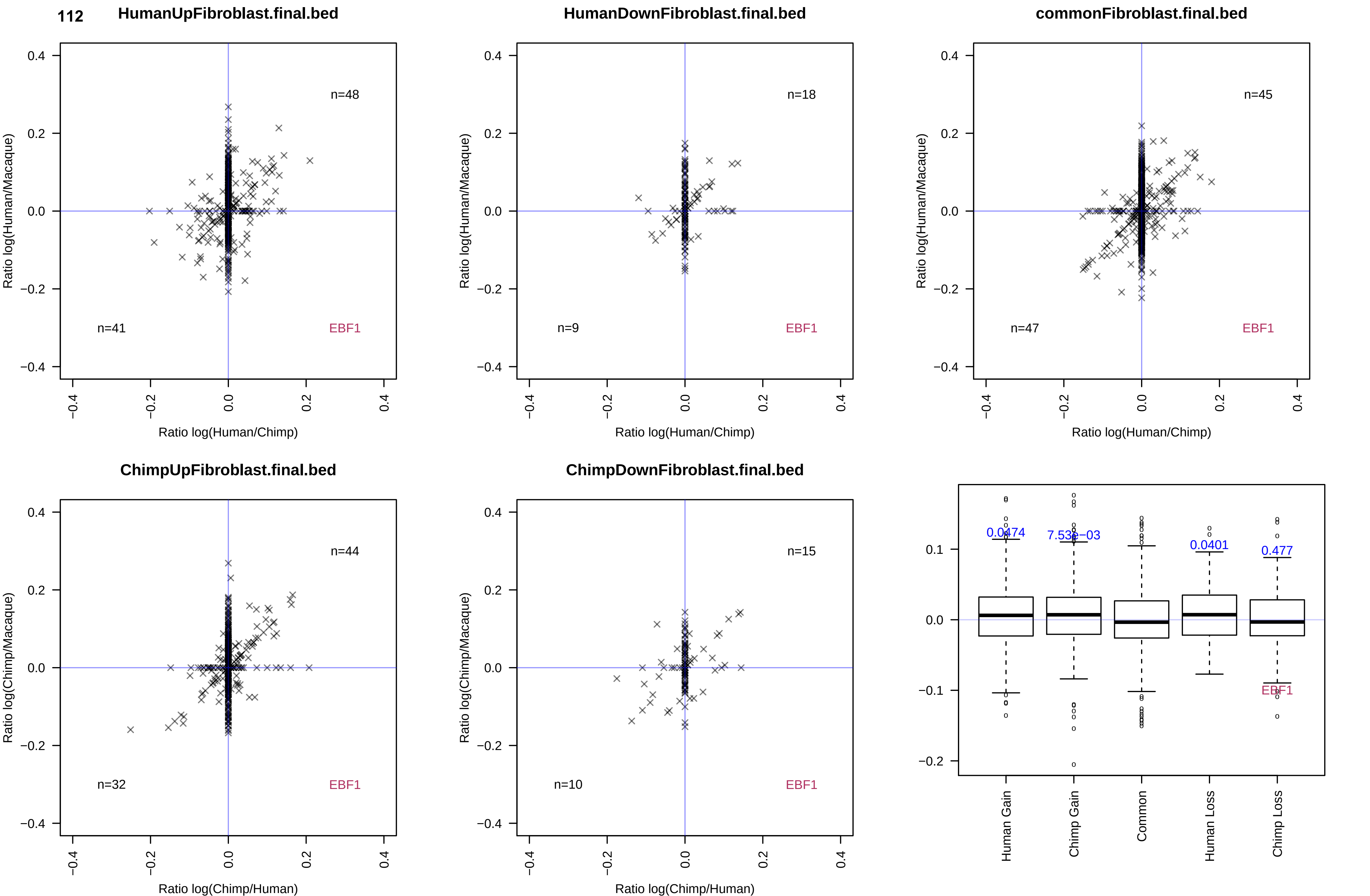
HumanUpFibroblast.final.bed
n=48
n=41
EBF1
HumanDownFibroblast.final.bed
n=18
n=9
EBF1
commonFibroblast.final.bed
n=45
n=47
EBF1
Ratio log(Human/Macaque)
Ratio log(Human/Chimp)
ChimpUpFibroblast.final.bed
n=44
n=32
EBF1
ChimpDownFibroblast.final.bed
n=15
n=10
EBF1
Ratio log(Chimp/Macaque)
Ratio log(Chimp/Human)
0.0474
7.53e−03
0.0401
0.477
EBF1
Human Gain
Chimp Gain
Common
Human Loss
Chimp Loss

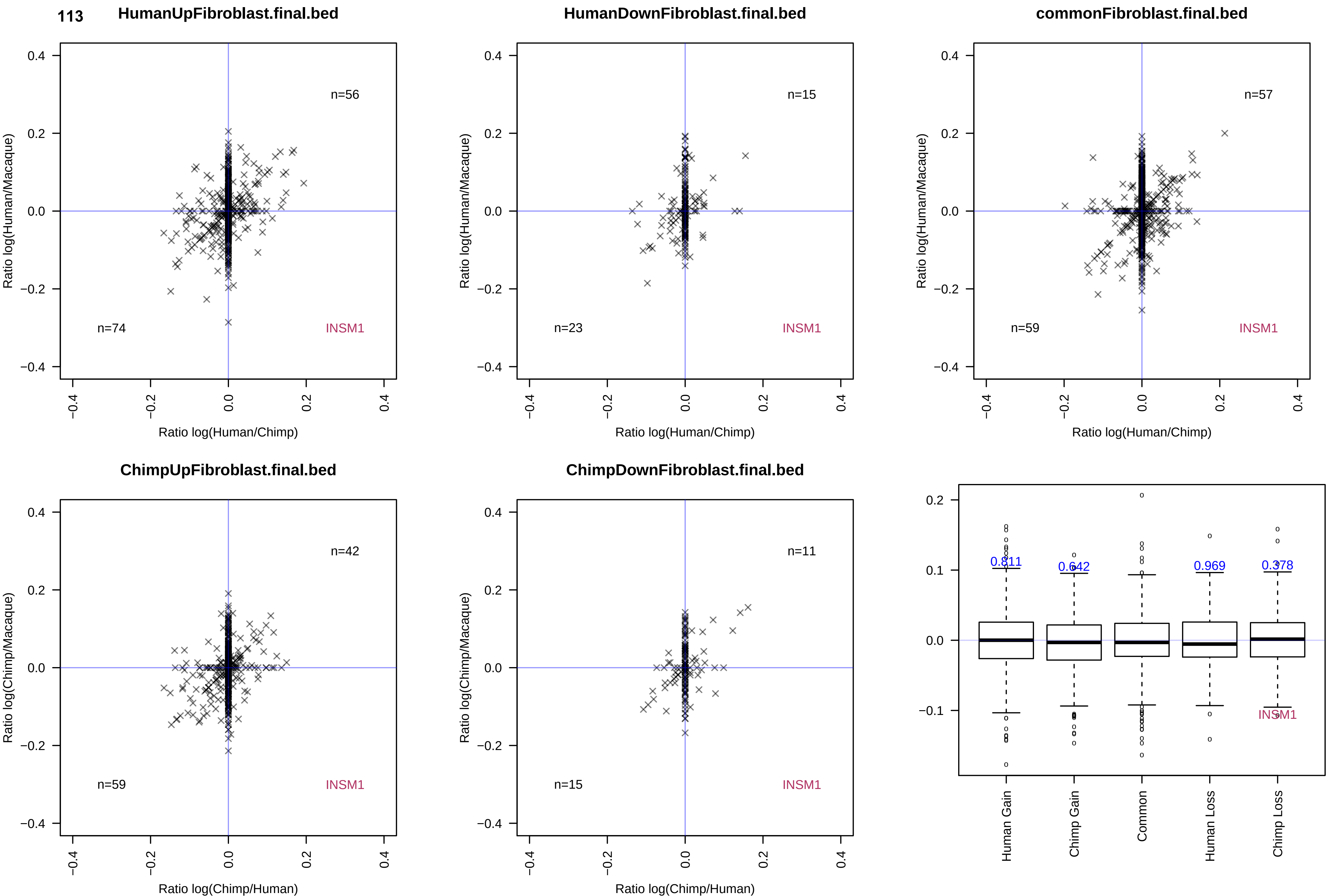
HumanUpFibroblast.final.bed
HumanDownFibroblast.final.bed
commonFibroblast.final.bed
ChimpUpFibroblast.final.bed
ChimpDownFibroblast.final.bed
Ratio log(Human/Macaque)
Ratio log(Human/Chimp)
Ratio log(Chimp/Macaque)
Ratio log(Chimp/Human)
n=56
n=74
n=15
n=23
n=57
n=59
n=42
n=59
n=11
n=15
INSM1
0.811
0.642
0.969
0.378
Human Gain
Chimp Gain
Common
Human Loss
Chimp Loss

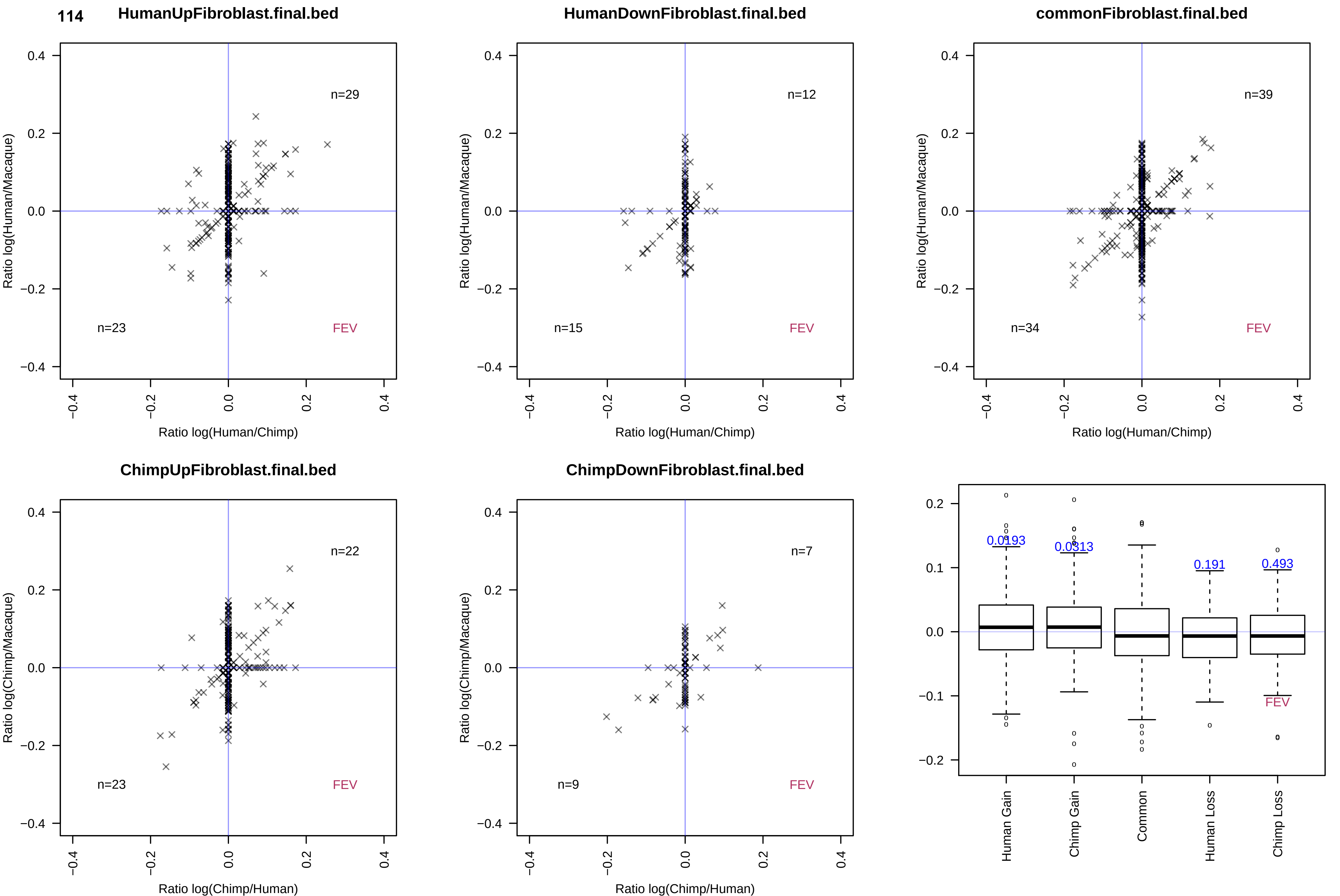
HumanUpFibroblast.final.bed
n=29
n=23
FEV
Ratio log(Human/Chimp)
Ratio log(Human/Macaque)
HumanDownFibroblast.final.bed
n=12
n=15
FEV
Ratio log(Human/Chimp)
Ratio log(Human/Macaque)
commonFibroblast.final.bed
n=39
n=34
FEV
Ratio log(Human/Chimp)
Ratio log(Human/Macaque)
ChimpUpFibroblast.final.bed
n=22
n=23
FEV
Ratio log(Chimp/Human)
Ratio log(Chimp/Macaque)
ChimpDownFibroblast.final.bed
n=7
n=9
FEV
Ratio log(Chimp/Human)
Ratio log(Chimp/Macaque)
0.0193
0.0313
0.191
0.493
FEV
Human Gain
Chimp Gain
Common
Human Loss
Chimp Loss

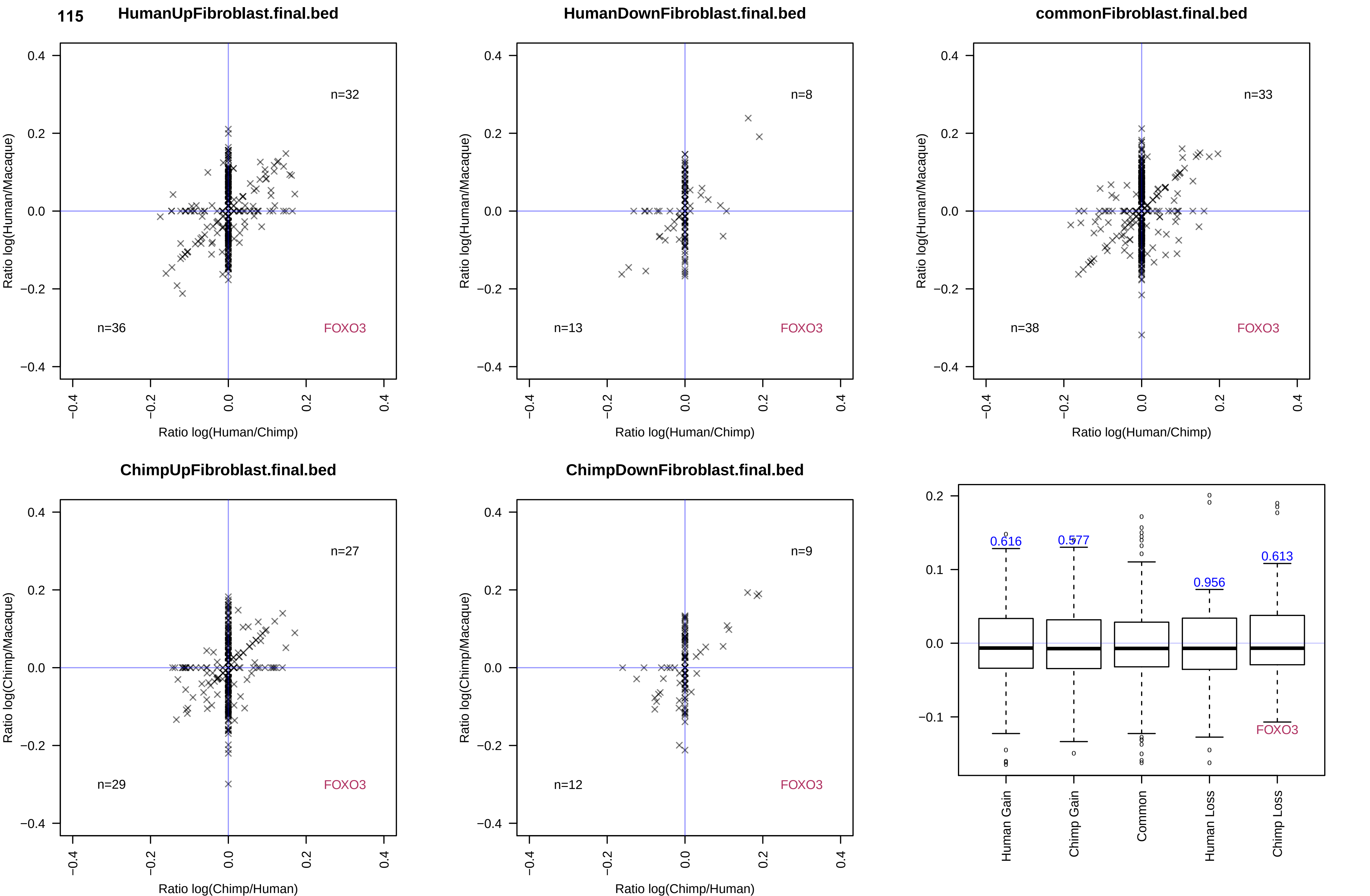
HumanUpFibroblast.final.bed
n=32
n=36
FOXO3
HumanDownFibroblast.final.bed
n=8
n=13
FOXO3
commonFibroblast.final.bed
n=33
n=38
FOXO3
Ratio log(Human/Macaque)
Ratio log(Human/Chimp)
ChimpUpFibroblast.final.bed
n=27
n=29
FOXO3
ChimpDownFibroblast.final.bed
n=9
n=12
FOXO3
Ratio log(Chimp/Macaque)
Ratio log(Chimp/Human)
0.616
0.577
0.956
0.613
FOXO3
Human Gain
Chimp Gain
Common
Human Loss
Chimp Loss

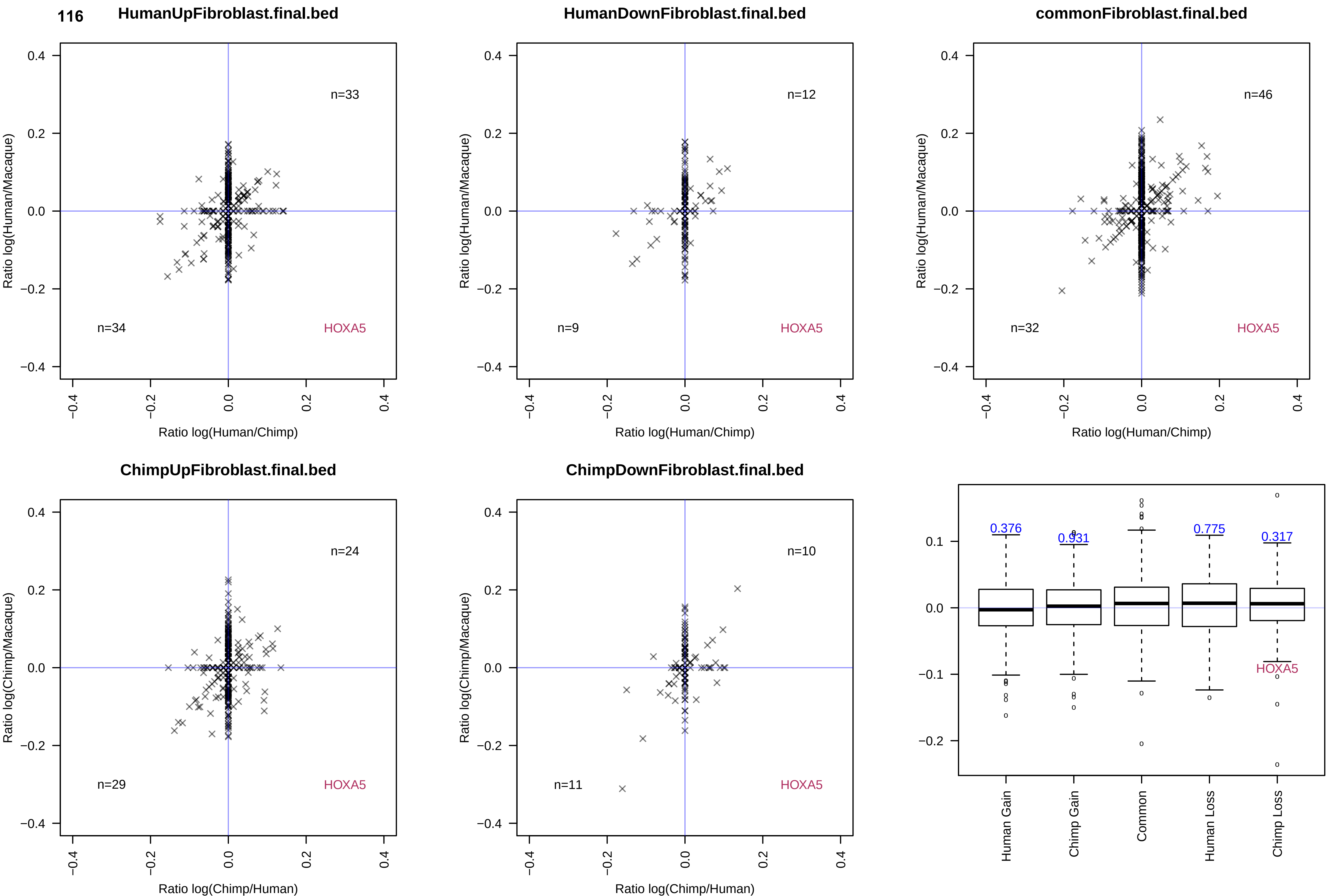
HumanUpFibroblast.final.bed
n=33
n=34
HOXA5
Ratio log(Human/Macaque)
Ratio log(Human/Chimp)
HumanDownFibroblast.final.bed
n=12
n=9
HOXA5
Ratio log(Human/Macaque)
Ratio log(Human/Chimp)
commonFibroblast.final.bed
n=46
n=32
HOXA5
Ratio log(Human/Macaque)
Ratio log(Human/Chimp)
ChimpUpFibroblast.final.bed
n=24
n=29
HOXA5
Ratio log(Chimp/Macaque)
Ratio log(Chimp/Human)
ChimpDownFibroblast.final.bed
n=10
n=11
HOXA5
Ratio log(Chimp/Macaque)
Ratio log(Chimp/Human)
0.376
0.931
0.775
0.317
HOXA5
Human Gain
Chimp Gain
Common
Human Loss
Chimp Loss

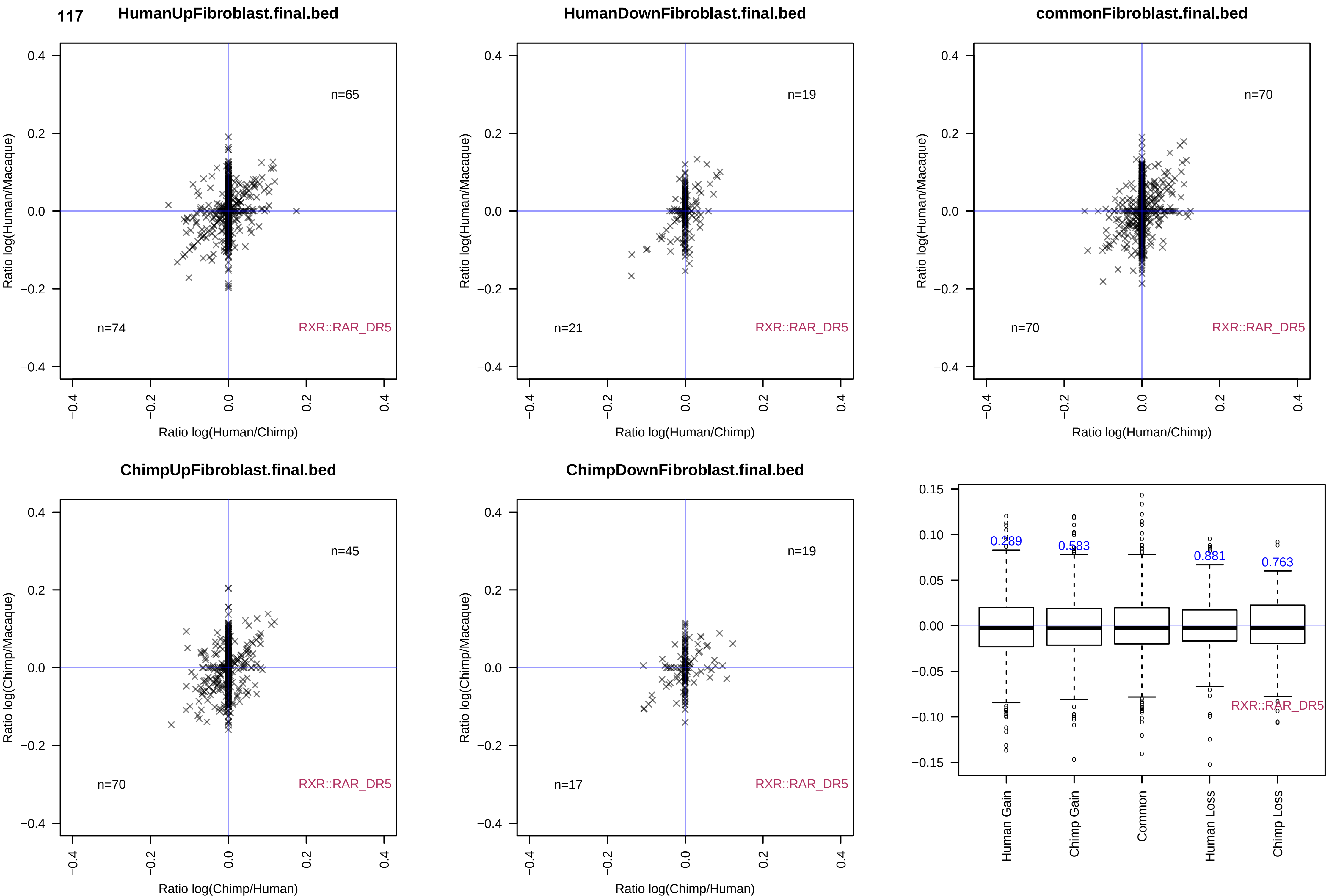
HumanUpFibroblast.final.bed
HumanDownFibroblast.final.bed
commonFibroblast.final.bed
ChimpUpFibroblast.final.bed
ChimpDownFibroblast.final.bed
Ratio log(Human/Macaque)
Ratio log(Human/Chimp)
Ratio log(Chimp/Macaque)
Ratio log(Chimp/Human)
n=65
n=74
n=19
n=21
n=70
n=70
n=45
n=70
n=19
n=17
RXR::RAR_DR5
0.289
0.583
0.881
0.763
Human Gain
Chimp Gain
Common
Human Loss
Chimp Loss

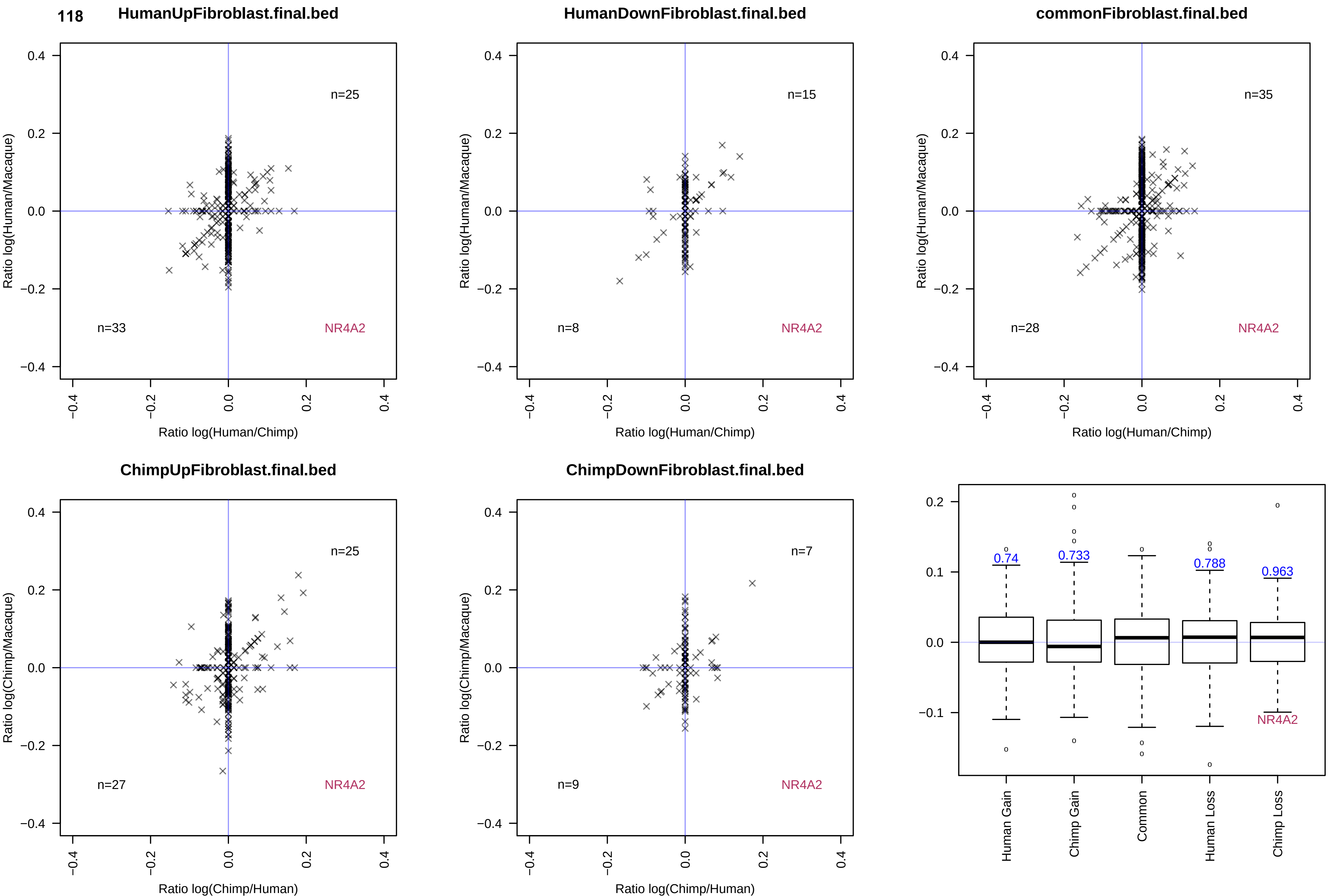
HumanUpFibroblast.final.bed
n=25
n=33
NR4A2
Ratio log(Human/Macaque)
Ratio log(Human/Chimp)
HumanDownFibroblast.final.bed
n=15
n=8
NR4A2
Ratio log(Human/Macaque)
Ratio log(Human/Chimp)
commonFibroblast.final.bed
n=35
n=28
NR4A2
Ratio log(Human/Macaque)
Ratio log(Human/Chimp)
ChimpUpFibroblast.final.bed
n=25
n=27
NR4A2
Ratio log(Chimp/Macaque)
Ratio log(Chimp/Human)
ChimpDownFibroblast.final.bed
n=7
n=9
NR4A2
Ratio log(Chimp/Macaque)
Ratio log(Chimp/Human)
0.74
0.733
0.788
0.963
NR4A2
Human Gain
Chimp Gain
Common
Human Loss
Chimp Loss

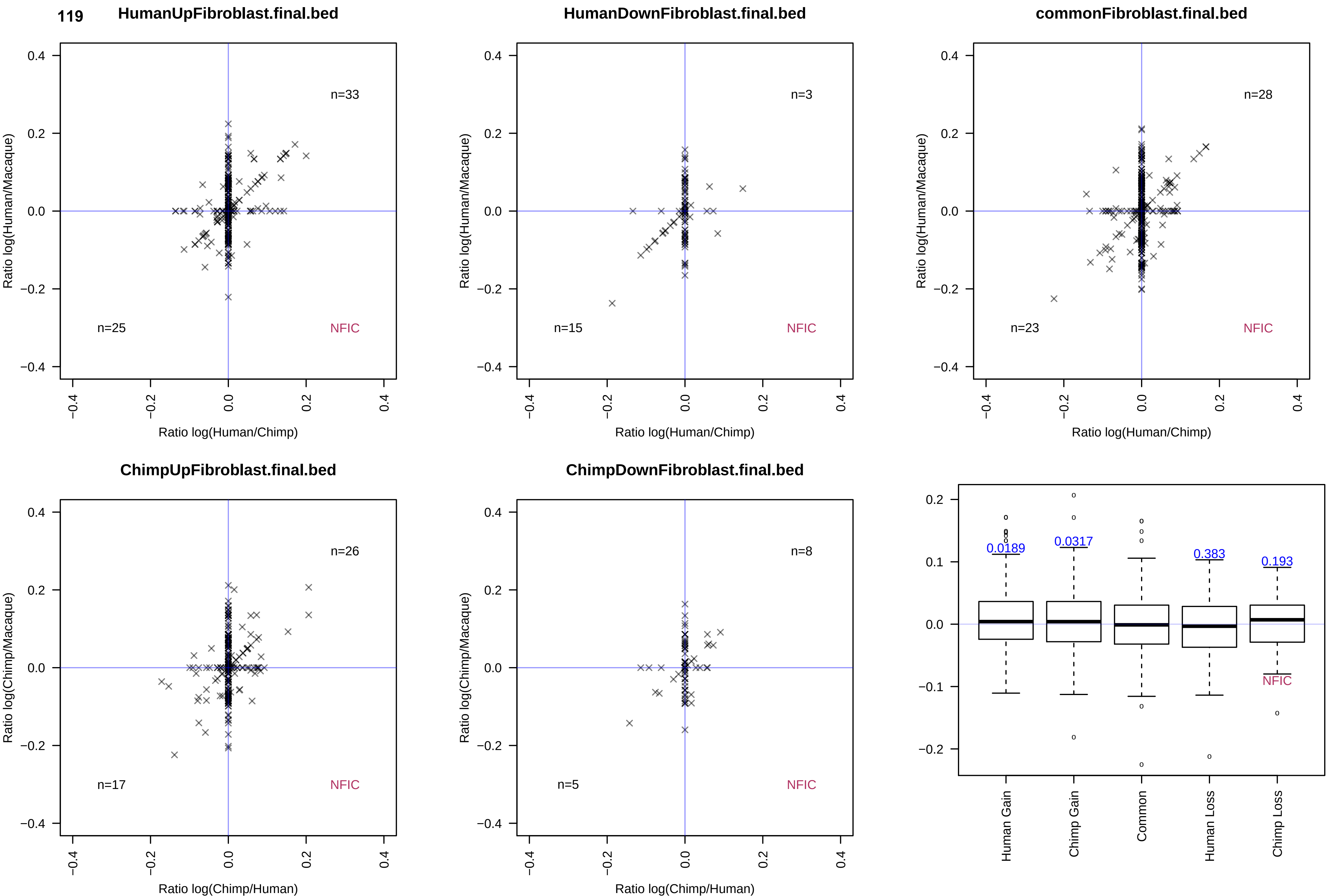
HumanUpFibroblast.final.bed
n=33
n=25
NFIC
Ratio log(Human/Macaque)
Ratio log(Human/Chimp)
HumanDownFibroblast.final.bed
n=3
n=15
NFIC
Ratio log(Human/Macaque)
Ratio log(Human/Chimp)
commonFibroblast.final.bed
n=28
n=23
NFIC
Ratio log(Human/Macaque)
Ratio log(Human/Chimp)
ChimpUpFibroblast.final.bed
n=26
n=17
NFIC
Ratio log(Chimp/Macaque)
Ratio log(Chimp/Human)
ChimpDownFibroblast.final.bed
n=8
n=5
NFIC
Ratio log(Chimp/Macaque)
Ratio log(Chimp/Human)
0.0189
0.0317
0.383
0.193
NFIC
Human Gain
Chimp Gain
Common
Human Loss
Chimp Loss

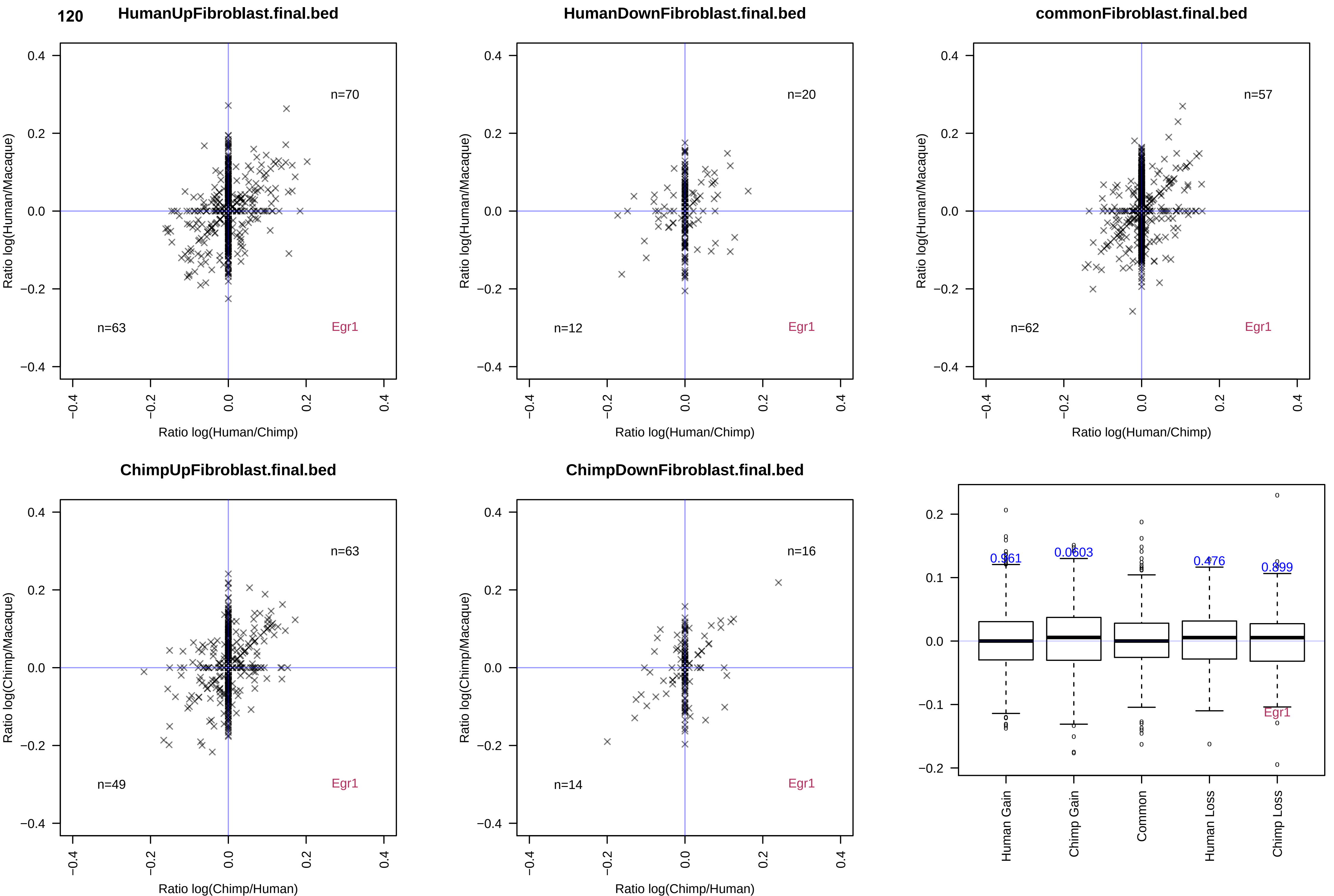
HumanUpFibroblast.final.bed
HumanDownFibroblast.final.bed
commonFibroblast.final.bed
ChimpUpFibroblast.final.bed
ChimpDownFibroblast.final.bed
Ratio log(Human/Macaque)
Ratio log(Human/Chimp)
Ratio log(Chimp/Macaque)
Ratio log(Chimp/Human)
n=70
n=63
n=20
n=12
n=57
n=62
n=63
n=49
n=16
n=14
Egr1
0.961
0.0603
0.476
0.899
Human Gain
Chimp Gain
Common
Human Loss
Chimp Loss

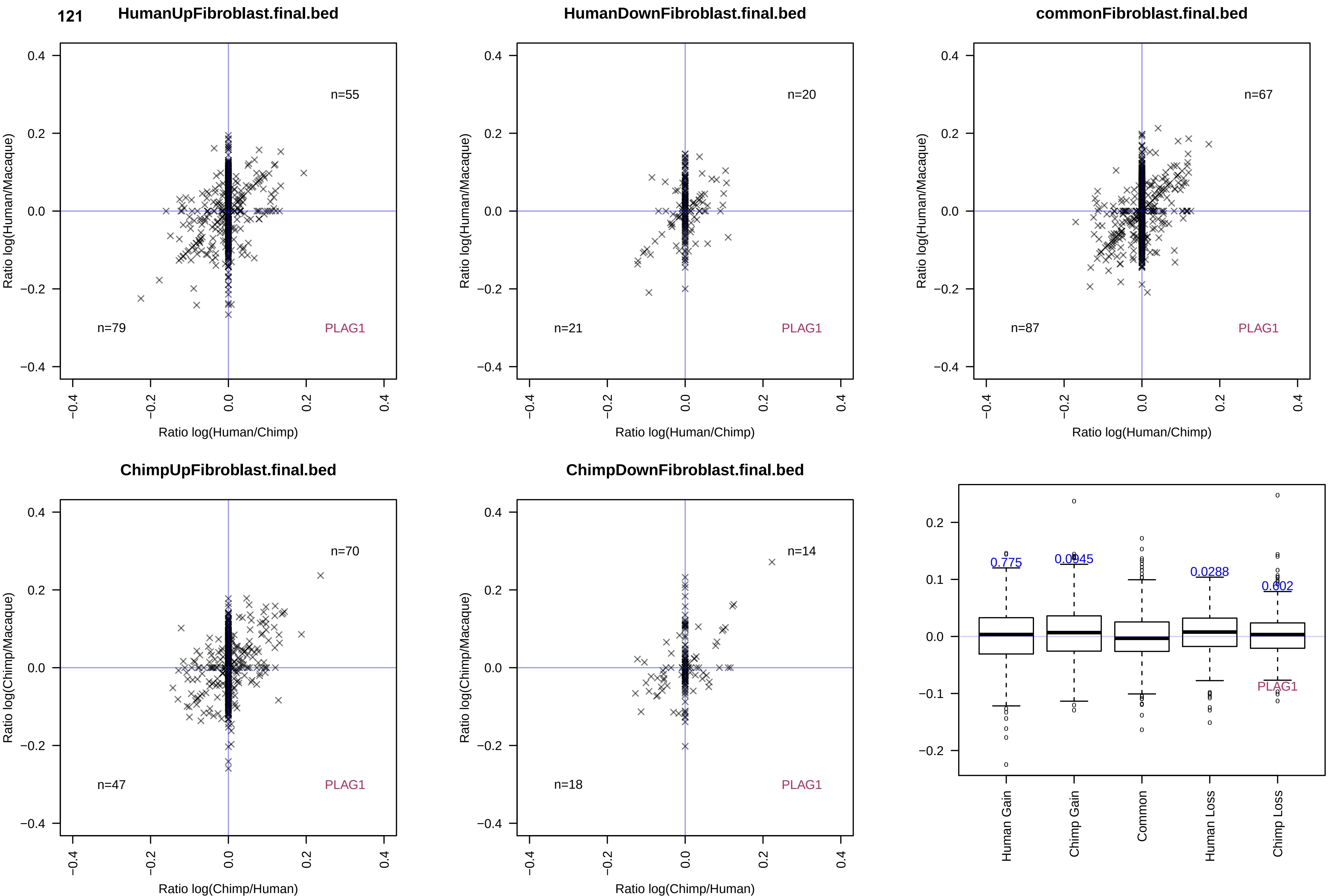
HumanUpFibroblast.final.bed
n=55
n=79
PLAG1
Ratio log(Human/Macaque)
Ratio log(Human/Chimp)
HumanDownFibroblast.final.bed
n=20
n=21
PLAG1
Ratio log(Human/Macaque)
Ratio log(Human/Chimp)
commonFibroblast.final.bed
n=67
n=87
PLAG1
Ratio log(Human/Macaque)
Ratio log(Human/Chimp)
ChimpUpFibroblast.final.bed
n=70
n=47
PLAG1
Ratio log(Chimp/Macaque)
Ratio log(Chimp/Human)
ChimpDownFibroblast.final.bed
n=14
n=18
PLAG1
Ratio log(Chimp/Macaque)
Ratio log(Chimp/Human)
0.775
0.0945
0.0288
0.602
PLAG1
Human Gain
Chimp Gain
Common
Human Loss
Chimp Loss

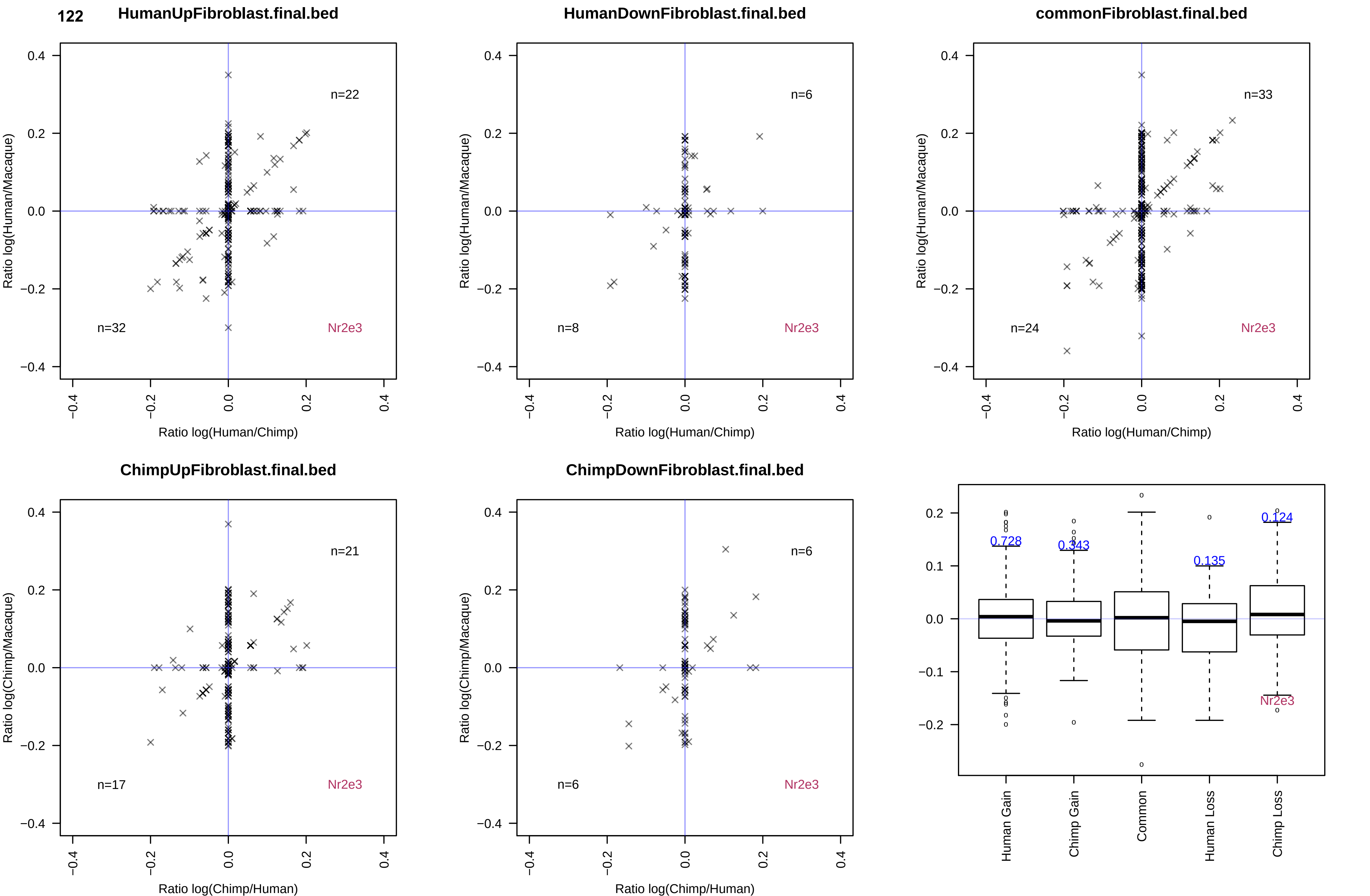
HumanUpFibroblast.final.bed
n=22
n=32
Nr2e3
Ratio log(Human/Macaque)
Ratio log(Human/Chimp)
HumanDownFibroblast.final.bed
n=6
n=8
Nr2e3
Ratio log(Human/Macaque)
Ratio log(Human/Chimp)
commonFibroblast.final.bed
n=33
n=24
Nr2e3
Ratio log(Human/Macaque)
Ratio log(Human/Chimp)
ChimpUpFibroblast.final.bed
n=21
n=17
Nr2e3
Ratio log(Chimp/Macaque)
Ratio log(Chimp/Human)
ChimpDownFibroblast.final.bed
n=6
n=6
Nr2e3
Ratio log(Chimp/Macaque)
Ratio log(Chimp/Human)
0.728
0.343
0.135
0.124
Nr2e3
Human Gain
Chimp Gain
Common
Human Loss
Chimp Loss

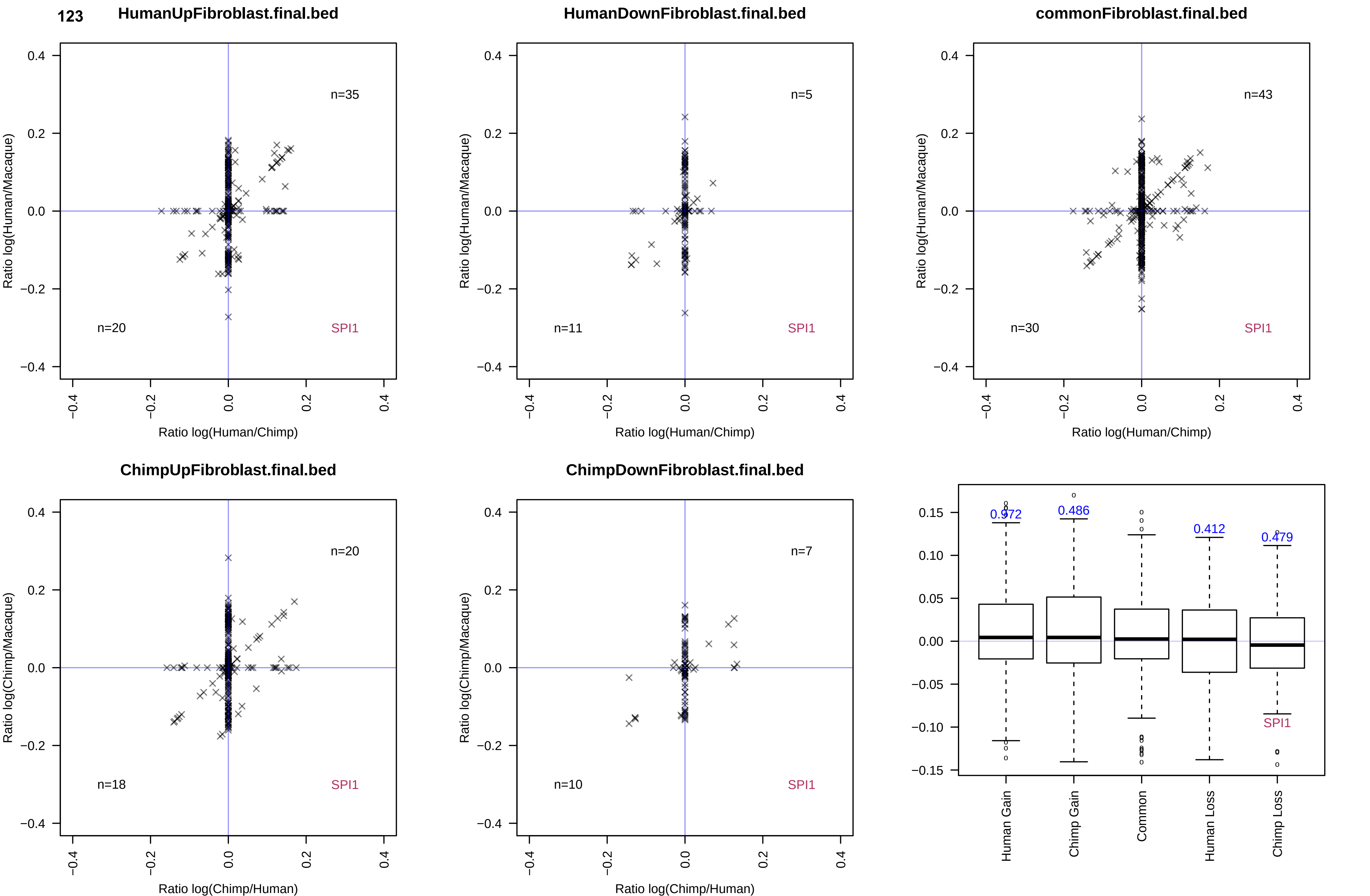
HumanUpFibroblast.final.bed
n=35
n=20
SPI1
Ratio log(Human/Macaque)
Ratio log(Human/Chimp)
HumanDownFibroblast.final.bed
n=5
n=11
SPI1
Ratio log(Human/Macaque)
Ratio log(Human/Chimp)
commonFibroblast.final.bed
n=43
n=30
SPI1
Ratio log(Human/Macaque)
Ratio log(Human/Chimp)
ChimpUpFibroblast.final.bed
n=20
n=18
SPI1
Ratio log(Chimp/Macaque)
Ratio log(Chimp/Human)
ChimpDownFibroblast.final.bed
n=7
n=10
SPI1
Ratio log(Chimp/Macaque)
Ratio log(Chimp/Human)
0.972
0.486
0.412
0.479
SPI1
Human Gain
Chimp Gain
Common
Human Loss
Chimp Loss

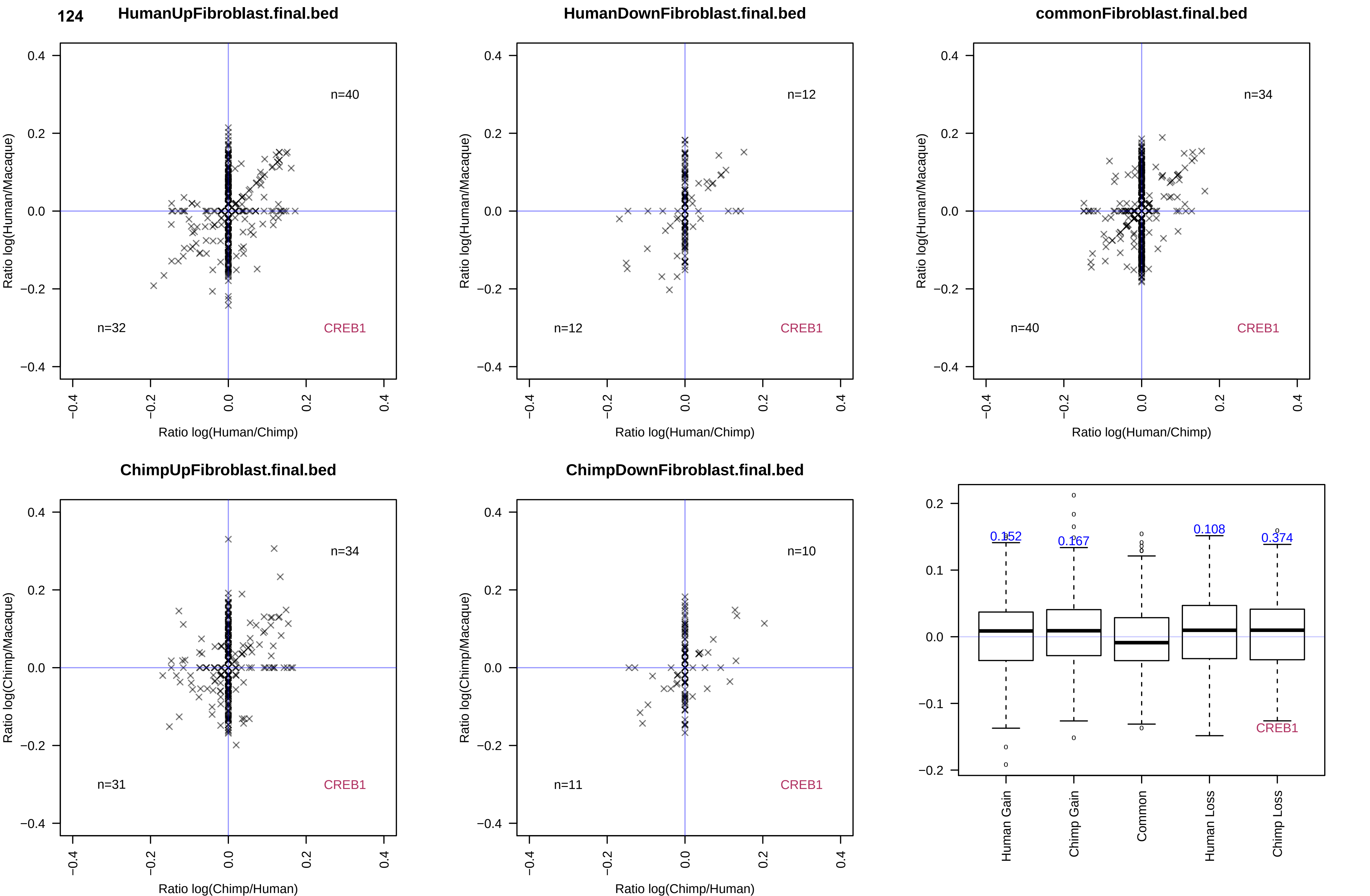
HumanUpFibroblast.final.bed
n=40
n=32
CREB1
Ratio log(Human/Chimp)
Ratio log(Human/Macaque)
HumanDownFibroblast.final.bed
n=12
n=12
CREB1
Ratio log(Human/Chimp)
Ratio log(Human/Macaque)
commonFibroblast.final.bed
n=34
n=40
CREB1
Ratio log(Human/Chimp)
Ratio log(Human/Macaque)
ChimpUpFibroblast.final.bed
n=34
n=31
CREB1
Ratio log(Chimp/Human)
Ratio log(Chimp/Macaque)
ChimpDownFibroblast.final.bed
n=10
n=11
CREB1
Ratio log(Chimp/Human)
Ratio log(Chimp/Macaque)
0.152
0.167
0.108
0.374
CREB1
Human Gain
Chimp Gain
Common
Human Loss
Chimp Loss

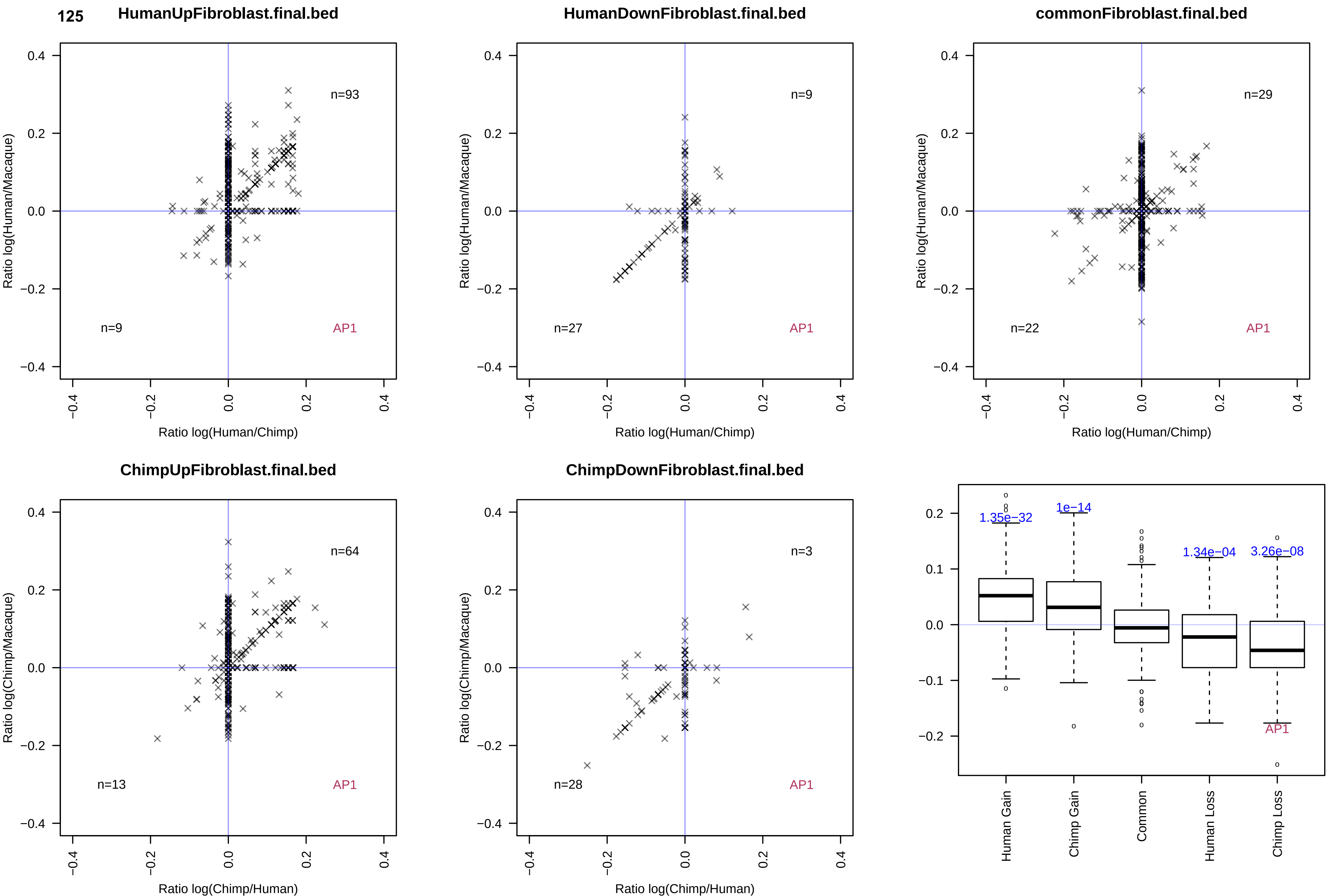
HumanUpFibroblast.final.bed
n=93
n=9
AP1
Ratio log(Human/Macaque)
Ratio log(Human/Chimp)
HumanDownFibroblast.final.bed
n=9
n=27
AP1
Ratio log(Human/Macaque)
Ratio log(Human/Chimp)
commonFibroblast.final.bed
n=29
n=22
AP1
Ratio log(Human/Macaque)
Ratio log(Human/Chimp)
ChimpUpFibroblast.final.bed
n=64
n=13
AP1
Ratio log(Chimp/Macaque)
Ratio log(Chimp/Human)
ChimpDownFibroblast.final.bed
n=3
n=28
AP1
Ratio log(Chimp/Macaque)
Ratio log(Chimp/Human)
1.35e−32
1e−14
1.34e−04
3.26e−08
AP1
Human Gain
Chimp Gain
Common
Human Loss
Chimp Loss

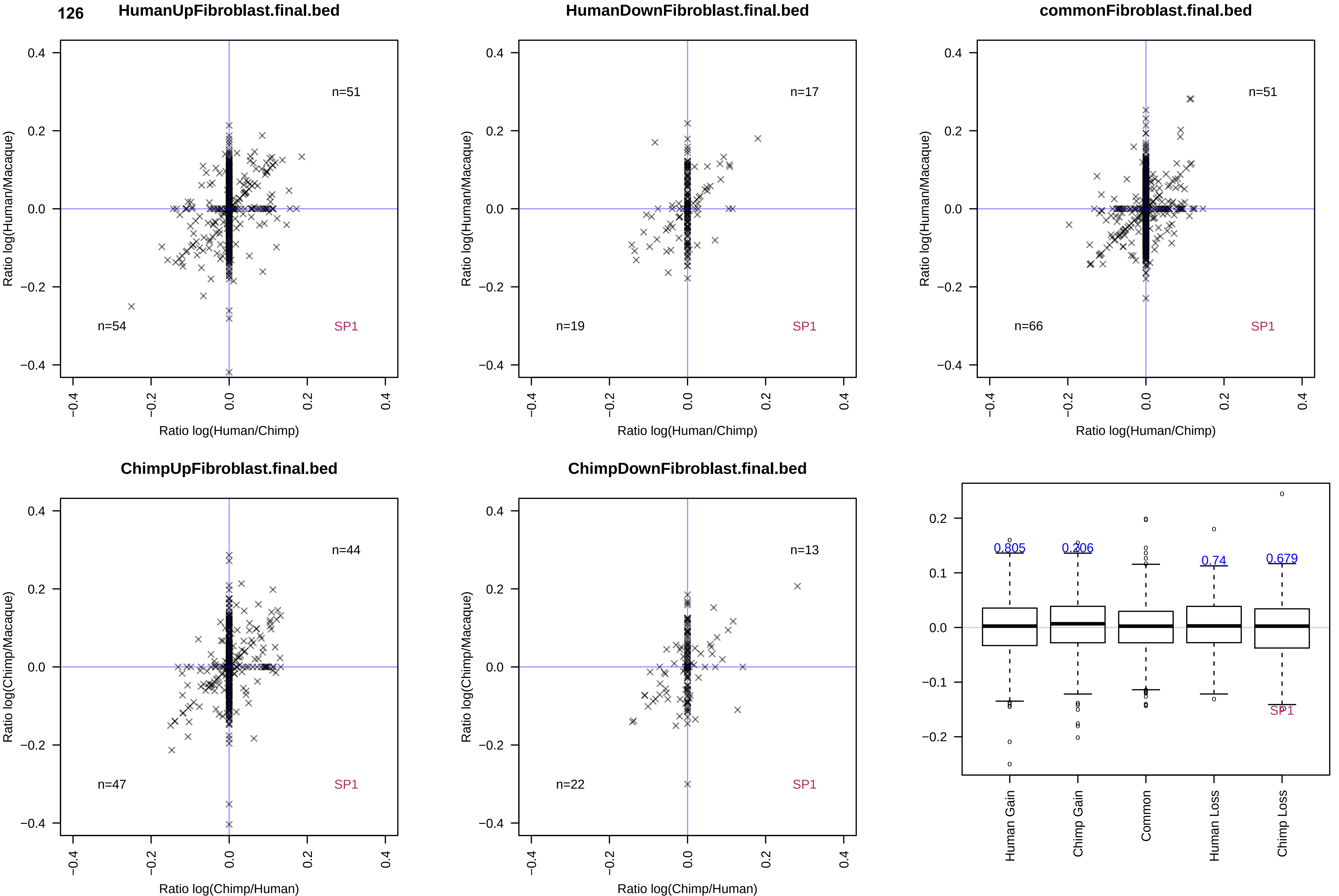
HumanUpFibroblast.final.bed
n=51
n=54
SP1
Ratio log(Human/Macaque)
Ratio log(Human/Chimp)
HumanDownFibroblast.final.bed
n=17
n=19
SP1
Ratio log(Human/Macaque)
Ratio log(Human/Chimp)
commonFibroblast.final.bed
n=51
n=66
SP1
Ratio log(Human/Macaque)
Ratio log(Human/Chimp)
ChimpUpFibroblast.final.bed
n=44
n=47
SP1
Ratio log(Chimp/Macaque)
Ratio log(Chimp/Human)
ChimpDownFibroblast.final.bed
n=13
n=22
SP1
Ratio log(Chimp/Macaque)
Ratio log(Chimp/Human)
0.805
0.206
0.74
0.679
SP1
Human Gain
Chimp Gain
Common
Human Loss
Chimp Loss

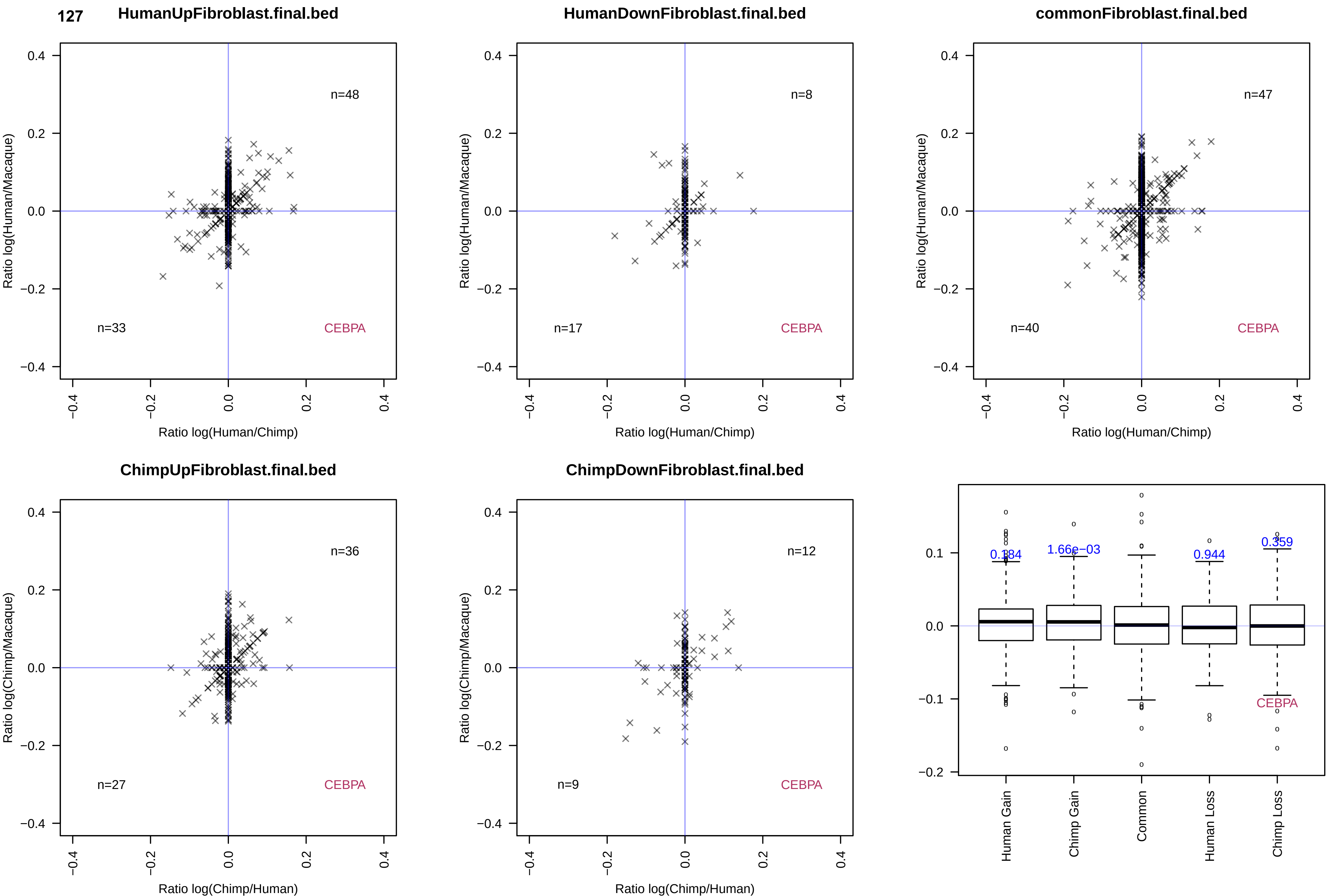
HumanUpFibroblast.final.bed
HumanDownFibroblast.final.bed
commonFibroblast.final.bed
ChimpUpFibroblast.final.bed
ChimpDownFibroblast.final.bed
Ratio log(Human/Macaque)
Ratio log(Human/Chimp)
Ratio log(Chimp/Macaque)
Ratio log(Chimp/Human)
n=48
n=33
n=8
n=17
n=47
n=40
n=36
n=27
n=12
n=9
CEBPA
0.184
1.66e−03
0.944
0.359
Human Gain
Chimp Gain
Common
Human Loss
Chimp Loss

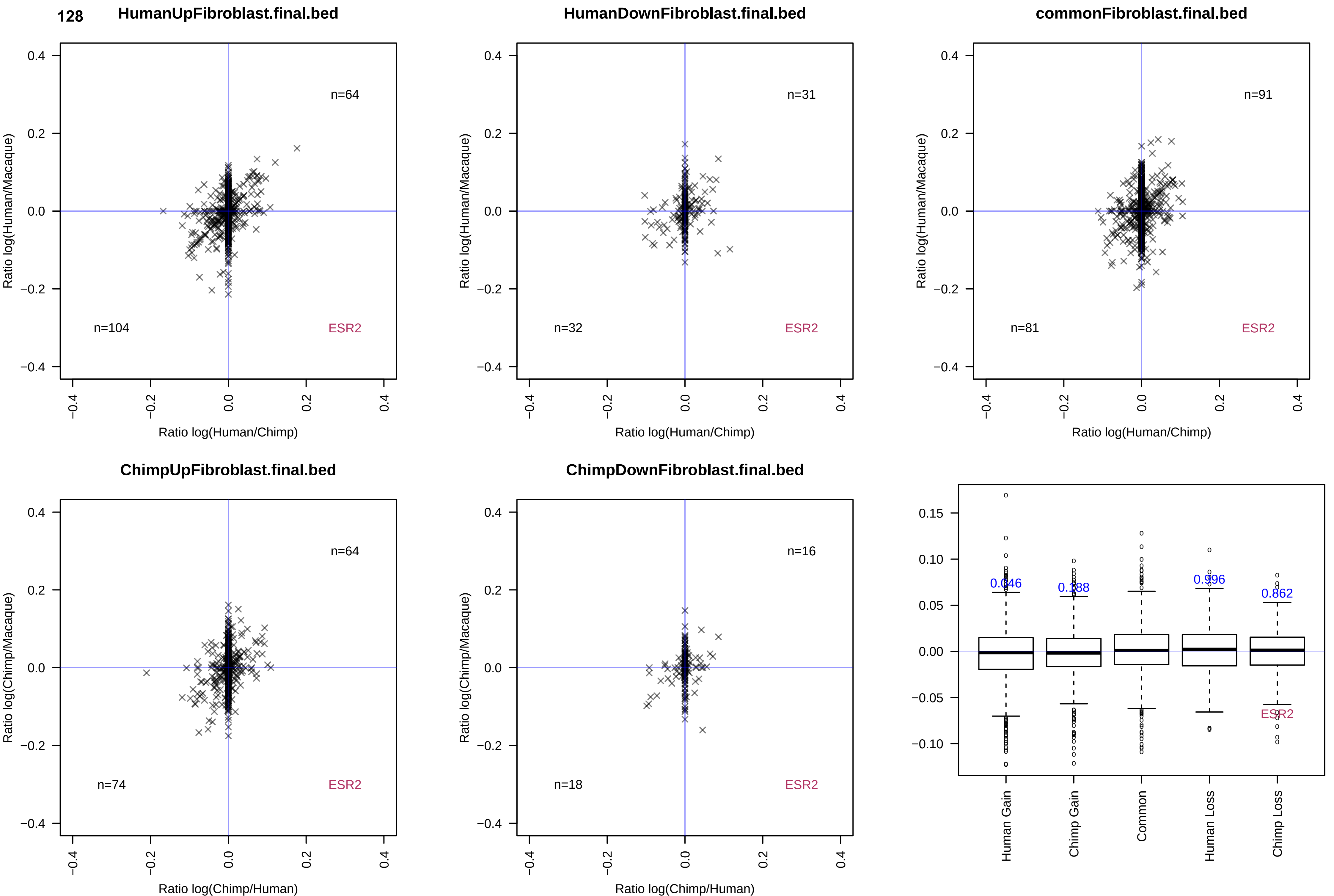
HumanUpFibroblast.final.bed
n=64
n=104
ESR2
Ratio log(Human/Macaque)
Ratio log(Human/Chimp)
HumanDownFibroblast.final.bed
n=31
n=32
ESR2
Ratio log(Human/Macaque)
Ratio log(Human/Chimp)
commonFibroblast.final.bed
n=91
n=81
ESR2
Ratio log(Human/Macaque)
Ratio log(Human/Chimp)
ChimpUpFibroblast.final.bed
n=64
n=74
ESR2
Ratio log(Chimp/Macaque)
Ratio log(Chimp/Human)
ChimpDownFibroblast.final.bed
n=16
n=18
ESR2
Ratio log(Chimp/Macaque)
Ratio log(Chimp/Human)
0.046
0.188
0.996
0.862
ESR2
Human Gain
Chimp Gain
Common
Human Loss
Chimp Loss

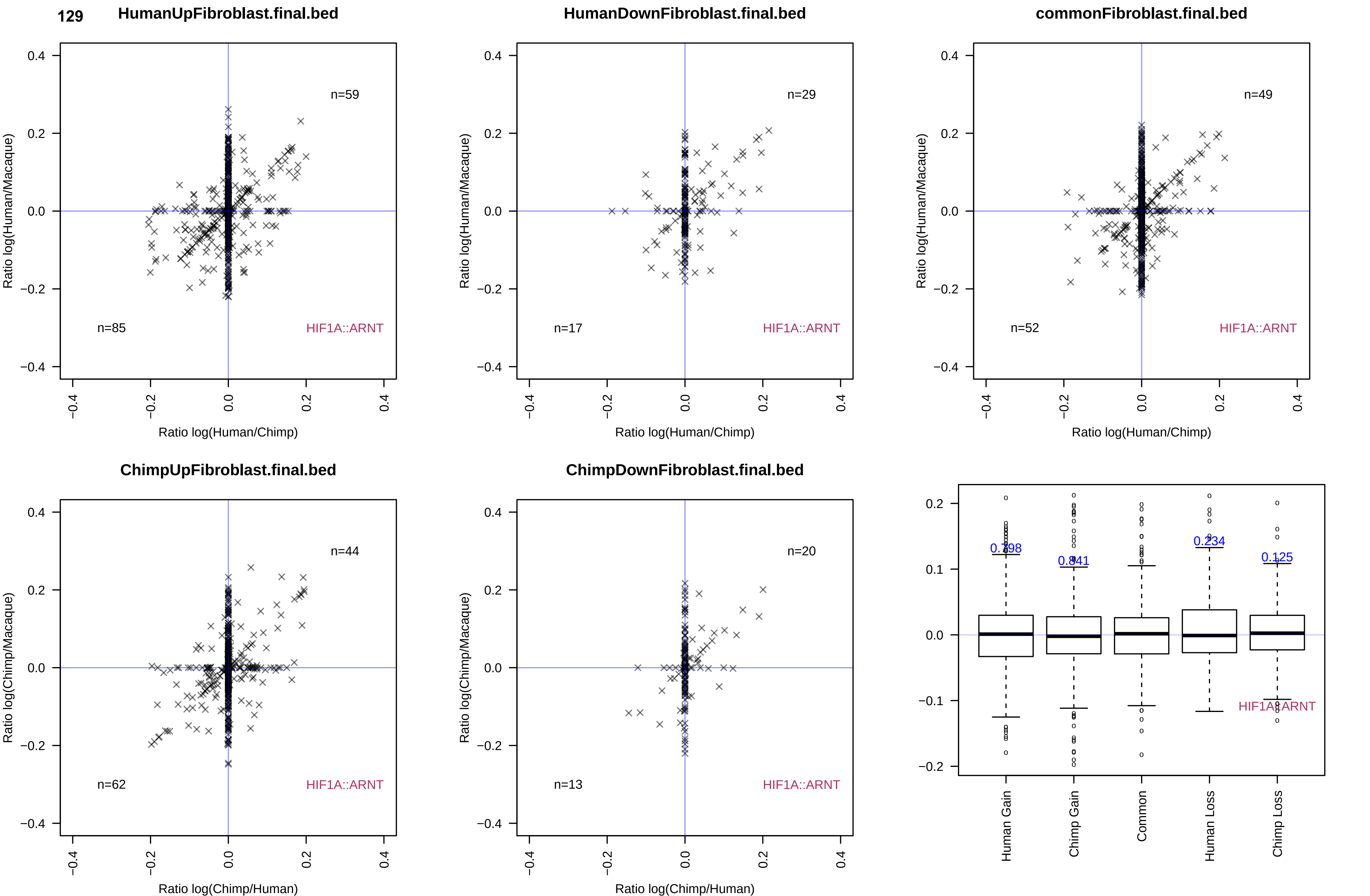
HumanUpFibroblast.final.bed
n=59
n=85
HIF1A::ARNT
Ratio log(Human/Macaque)
Ratio log(Human/Chimp)
HumanDownFibroblast.final.bed
n=29
n=17
HIF1A::ARNT
Ratio log(Human/Macaque)
Ratio log(Human/Chimp)
commonFibroblast.final.bed
n=49
n=52
HIF1A::ARNT
Ratio log(Human/Macaque)
Ratio log(Human/Chimp)
ChimpUpFibroblast.final.bed
n=44
n=62
HIF1A::ARNT
Ratio log(Chimp/Macaque)
Ratio log(Chimp/Human)
ChimpDownFibroblast.final.bed
n=20
n=13
HIF1A::ARNT
Ratio log(Chimp/Macaque)
Ratio log(Chimp/Human)
0.798
0.841
0.234
0.125
HIF1A::ARNT
Human Gain
Chimp Gain
Common
Human Loss
Chimp Loss

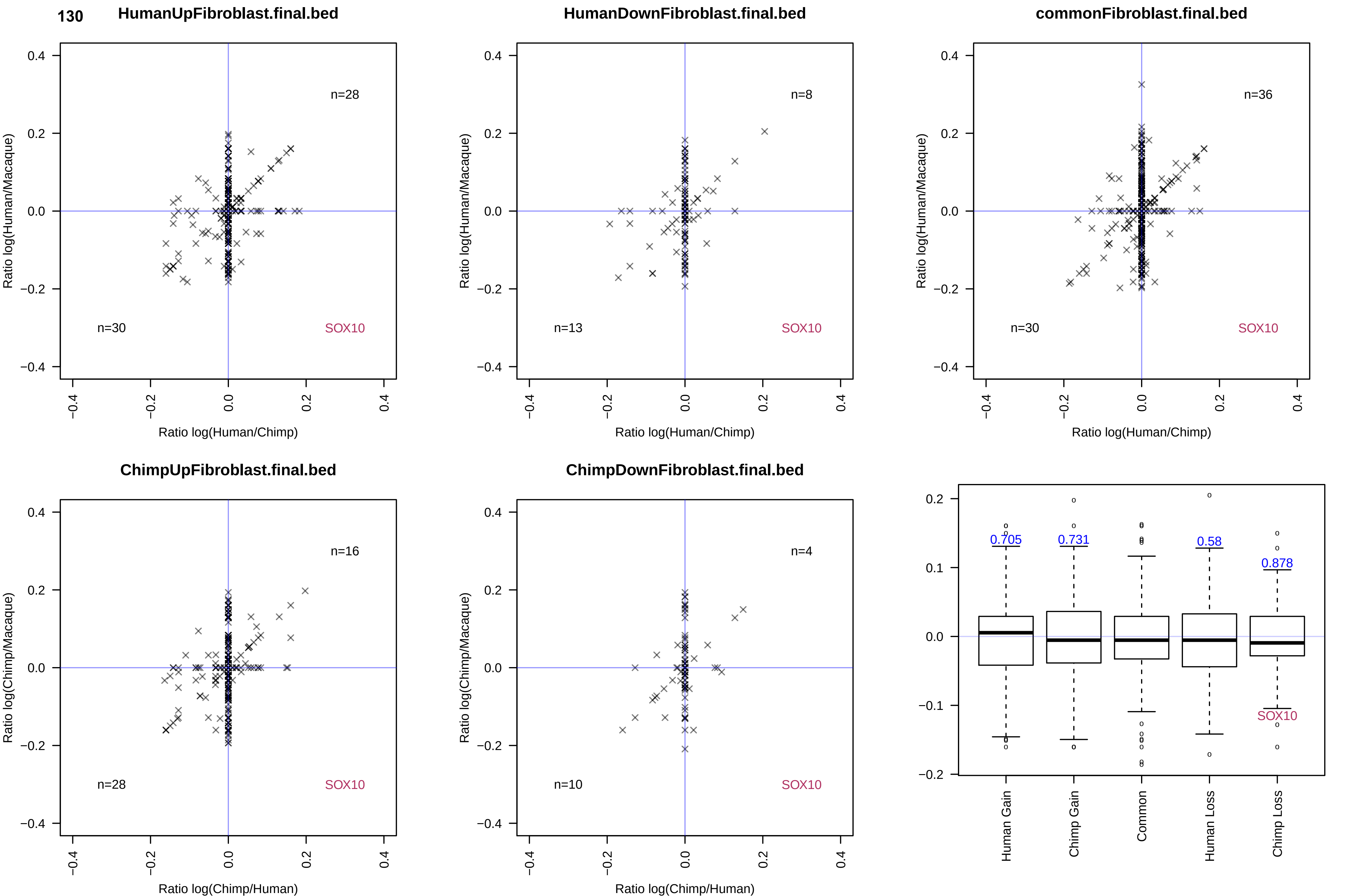
HumanUpFibroblast.final.bed
n=28
n=30
SOX10
Ratio log(Human/Macaque)
Ratio log(Human/Chimp)
HumanDownFibroblast.final.bed
n=8
n=13
SOX10
Ratio log(Human/Macaque)
Ratio log(Human/Chimp)
commonFibroblast.final.bed
n=36
n=30
SOX10
Ratio log(Human/Macaque)
Ratio log(Human/Chimp)
ChimpUpFibroblast.final.bed
n=16
n=28
SOX10
Ratio log(Chimp/Macaque)
Ratio log(Chimp/Human)
ChimpDownFibroblast.final.bed
n=4
n=10
SOX10
Ratio log(Chimp/Macaque)
Ratio log(Chimp/Human)
0.705
0.731
0.58
0.878
SOX10
Human Gain
Chimp Gain
Common
Human Loss
Chimp Loss
