# Supplementary figures and images for: Extensive Evolutionary Changes in Regulatory Element Activity during Human Origins Are Associated with Altered Gene Expression and Positive Selection
Source: PLoS Genet. 2012 Jun 28;8(6):e1002789. doi: 10.1371/journal.pgen.1002789 (PMC3386175; doi:10.1371/journal.pgen.1002789)

## Chimpanzee DHS gain

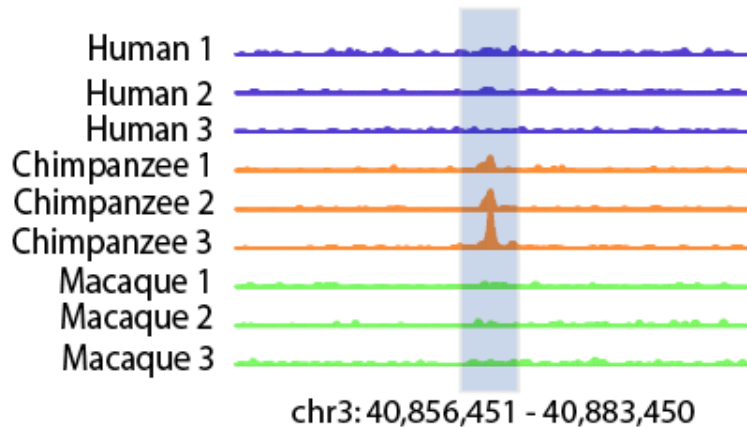

## Chimpanzee DHS loss

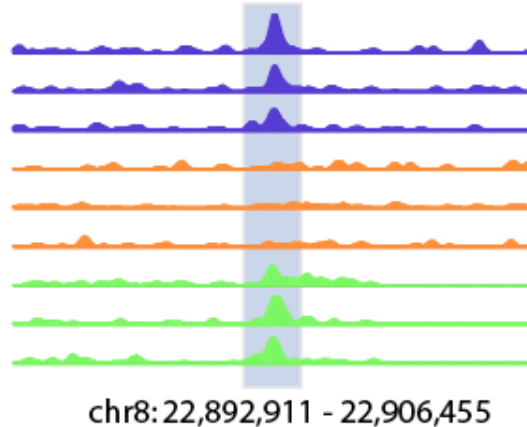

Supplement: Figure S1 — Representative examples of chimpanzee DHS gain and chimpanzee DHS loss. (PDF) [file pgen.1002789.s002.pdf]

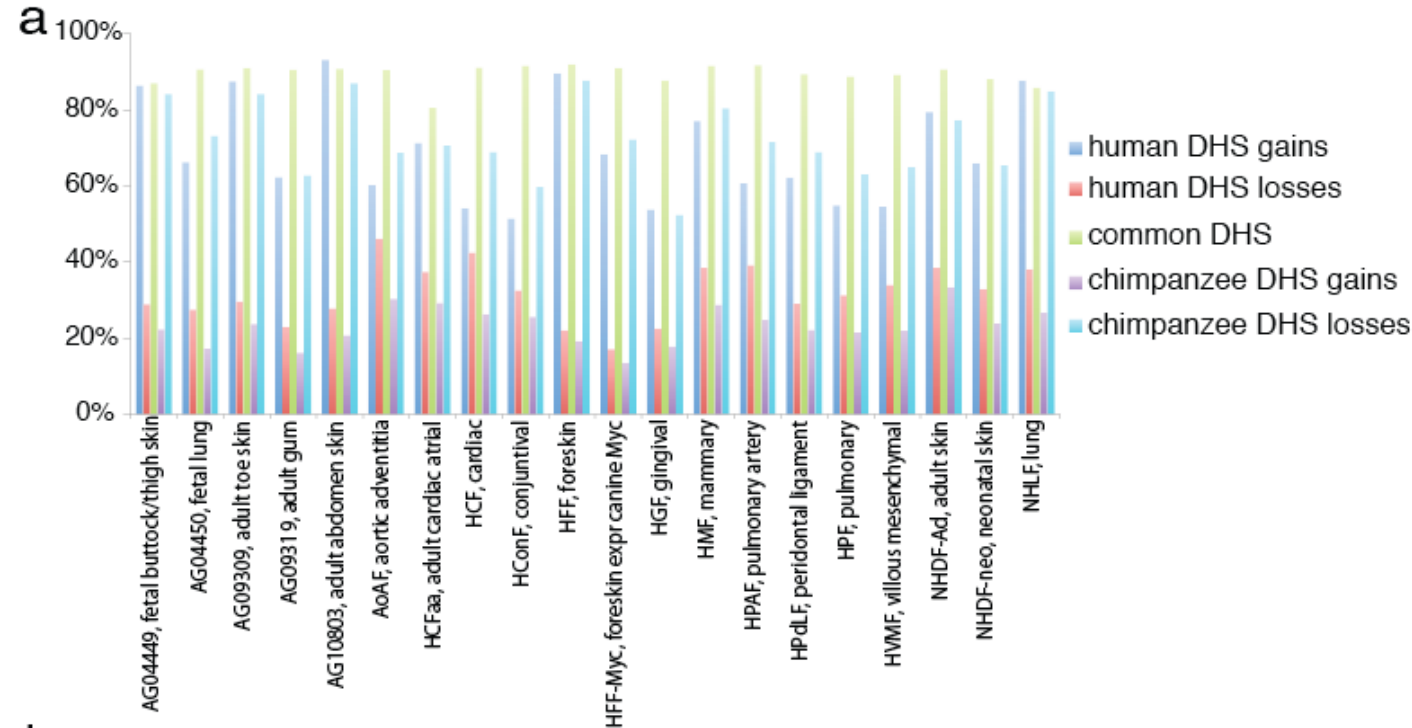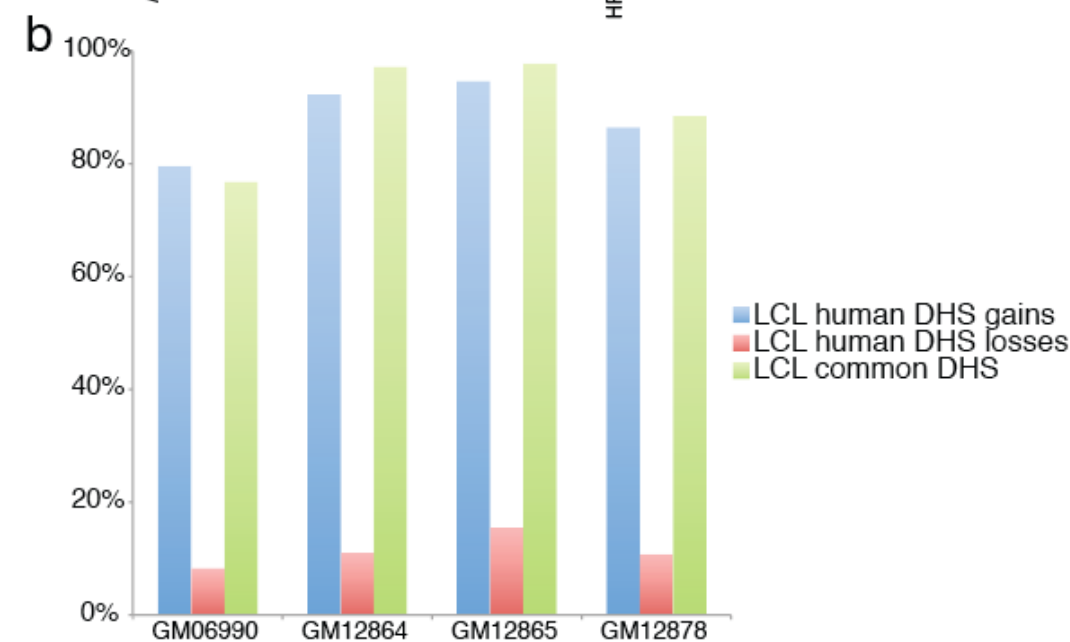

Supplement: Figure S2 — Species-specific gains and common DHS sites are highly reproducible in independently isolated matched cell types. (a) Human DHS gains/losses, Chimp DHS gains/losses, and Common DHS sites detected in fibroblast cells were compared to independently derived ENCODE Fibroblast DNase-seq data generated by the University of Washington ENCODE group. (b) LCL human DHS gains/losses/common compared to 4 independently derived ENCODE lymphoblastoid samples also identified by the University of Washington ENCODE group. (PDF) [file pgen.1002789.s003.pdf]

**a**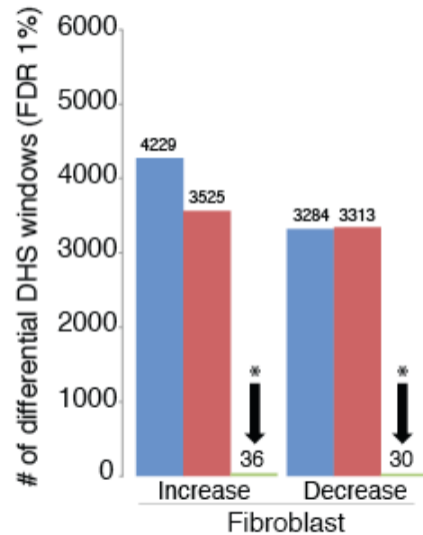**b**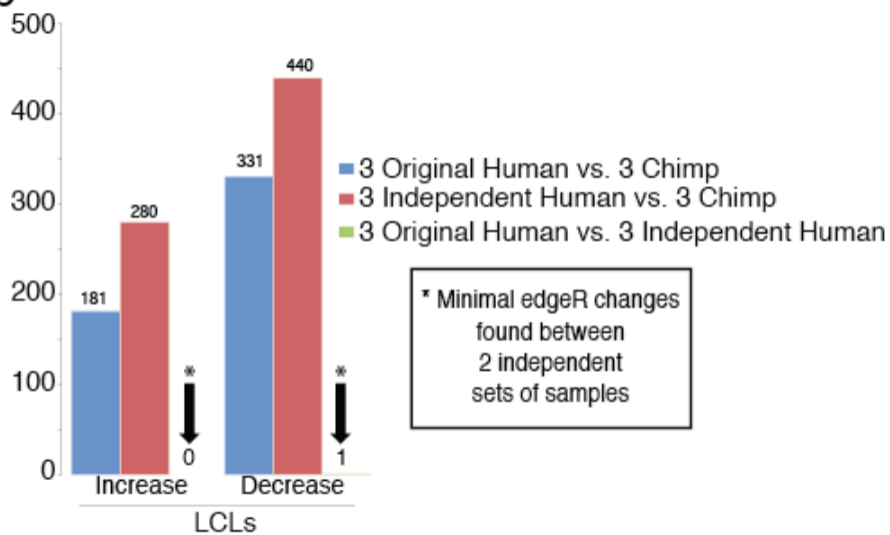

Supplement: Figure S3 — Differential edgeR analysis of original human and chimpanzee samples to independently analyzed cell samples. Similar numbers of human gains and losses are identified when comparing independently derived human fibroblasts (a) and LCLs (b) to chimpanzee fibroblasts and LCLs. Very few differentially open chromatin regions were identified by edgeR when comparing the 3 original human samples to 3 independently derived human fibroblast and LCL samples. (PDF) [file pgen.1002789.s004.pdf]

Number of cell types overlapping DHS sites

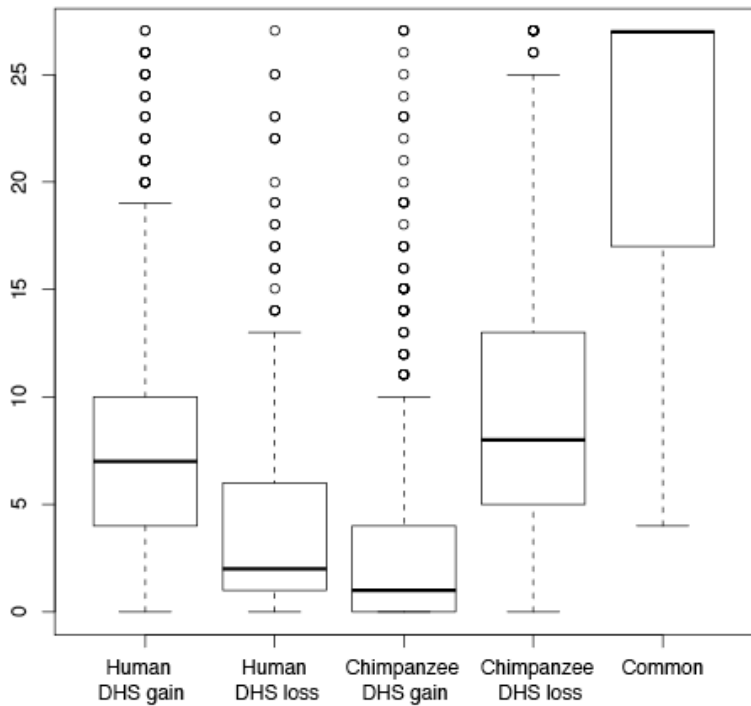

Supplement: Figure S5 — Boxplot of the binary comparison of human DHS gain/loss, chimpanzee DHS gain/loss, and Common regions to the DHS peak calls from the 27 other human cell types. (PDF) [file pgen.1002789.s006.pdf]

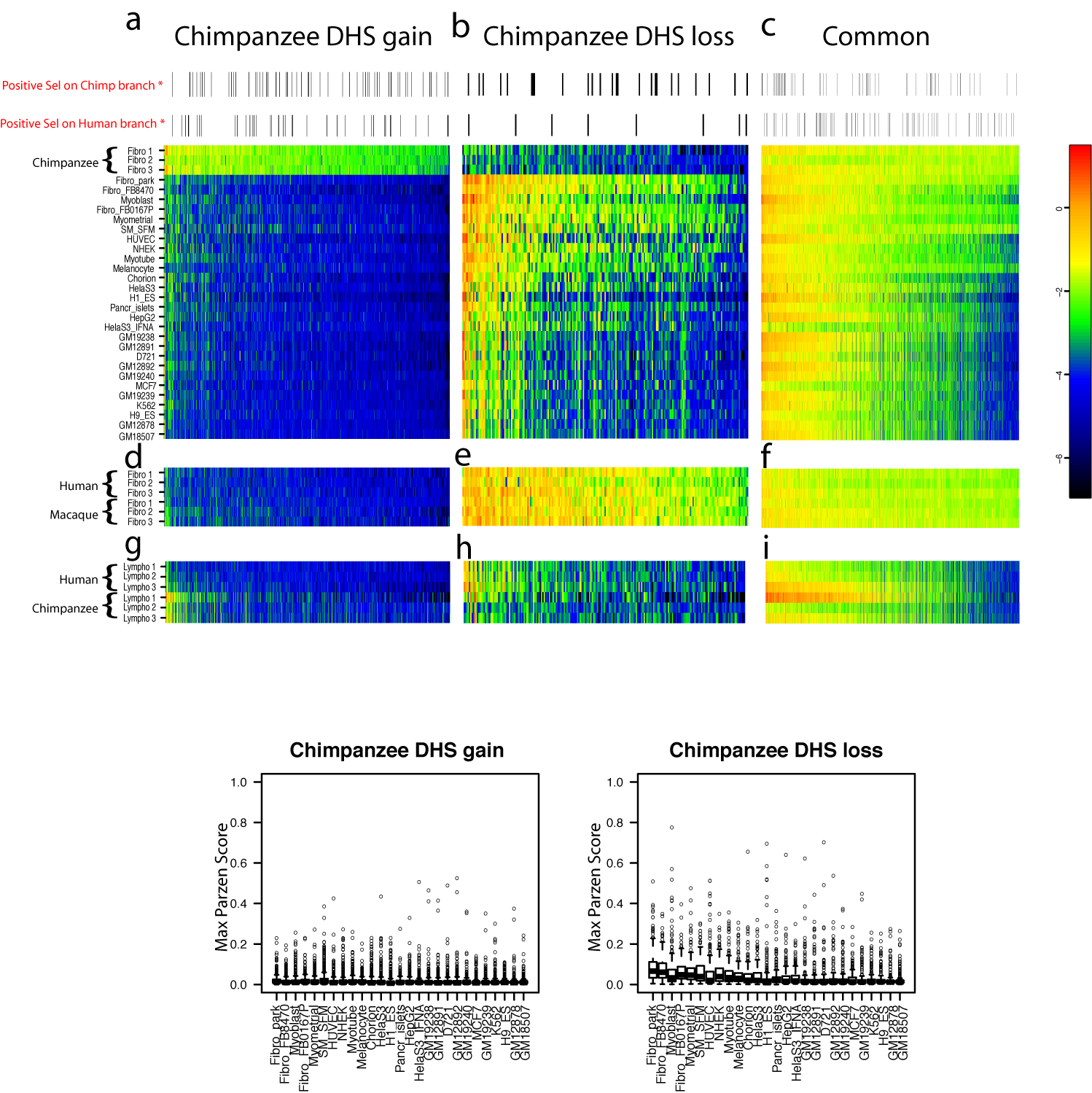

Supplement: Figure S6 — Comparison of chimpanzee DHS gains and DHS losses to DNase-seq data from other human cell types. These regions were compared to DNase-seq data generated from 27 other human cell types (Table S3). Heatmap signal intensities are of maximum DNase-seq parzen scores in log scale, where red indicates a higher DNase-seq score and blue indicates lower DNase-seq scores. (a) Chimpanzee DHS sites were identified as differentially open (chimpanzee DHS gain) in chimpanzee fibroblasts compared to human/macaque fibroblasts. (b) Chimpanzee DHS sites identified as differentially closed (Chimpanzee DHS loss) compared to human and macaque fibroblasts. (c) DNase-seq signal values for Common regions representing DHS sites in all three species. Note that more than 50% of Common regions are also DHS sites in other human tissues. (d, e, f) DNase-seq signal values for same regions as (a, b, c), but DNase-seq data is from orthologous region from human and macaque fibroblasts. (g, h, i) DNase-seq values for same regions as (a, b, c), but from human and chimpanzee LCLs. (bottom) Box plot shows intensity values shown in heatmaps. (TIF) [file pgen.1002789.s007.tif]

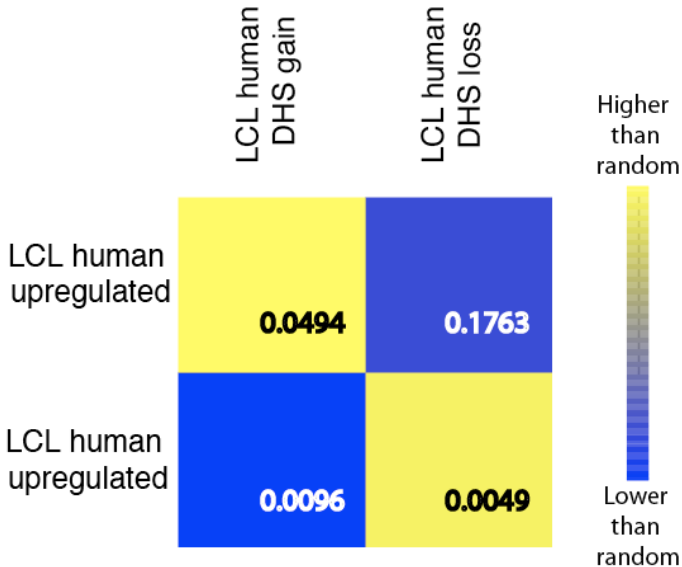

Supplement: Figure S8 — Comparison of chromatin DHS gains and DHS losses with genes that are upregulated and downregulated in human vs. chimpanzee lymphoblastoid cell lines (LCLs). Yellow represents chromatin and expression matches that occur more often than random permutations, while blue represent less often. P value indicated in each box. (PDF) [file pgen.1002789.s009.pdf]

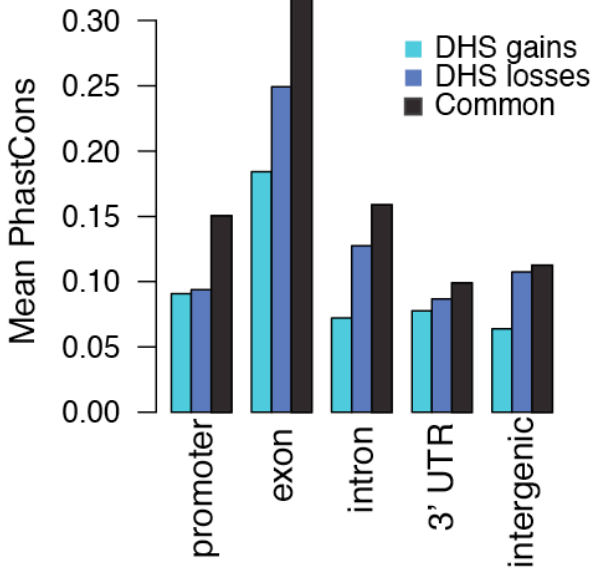

Supplement: Figure S9 — Phastcons figure showing sequence conservation. Histograms comparing the distribution of maximum PhastCons scores for pooled DHS gains (both human- and chimp-specific increases), pooled DHS losses (both human- and chimp-specific decreases), and Common regions. We pooled regions because the distributions look similar when divided (data not shown). Common regions have higher PhastCons scores. Average PhastCons scores show similar trends (data not shown). (PDF) [file pgen.1002789.s010.pdf]

Human DHS gain

Human DHS loss

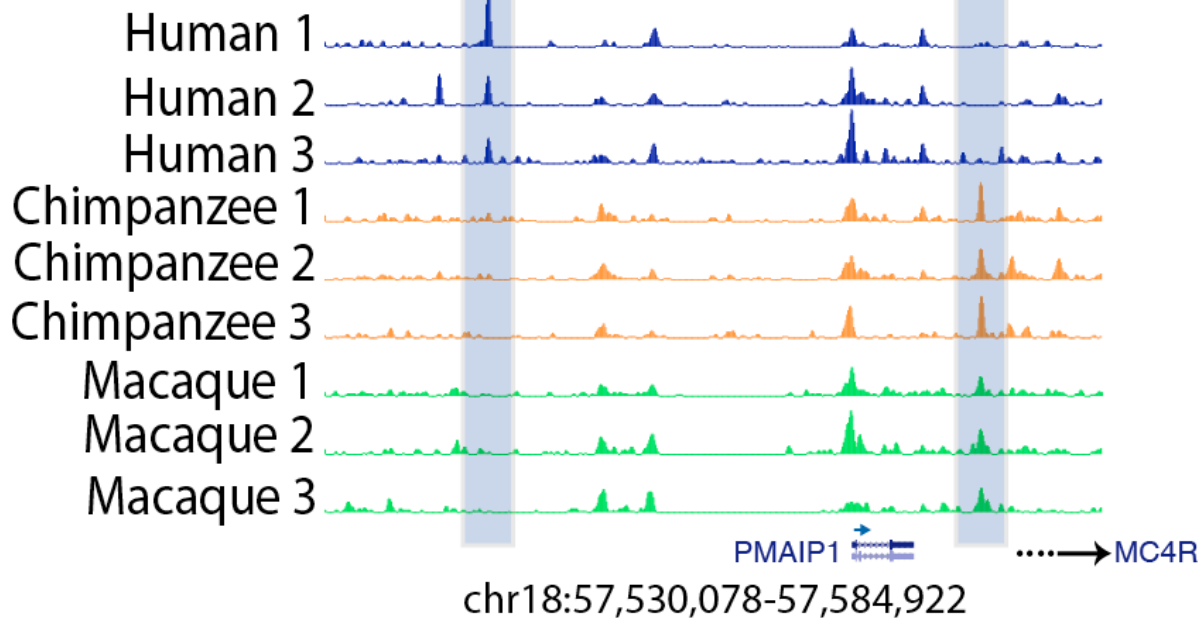

Supplement: Figure S11 — Potential regulatory element shuffling associated with obesity. Shown is a human DHS gain nearby a human DHS loss between the PMAIP1 and melanocortin 4 receptor (MC4R). MC4R is a gene shown to cause autosomal dominant obesity. (PDF) [file pgen.1002789.s012.pdf]

Human DHS gain

Human DHS loss

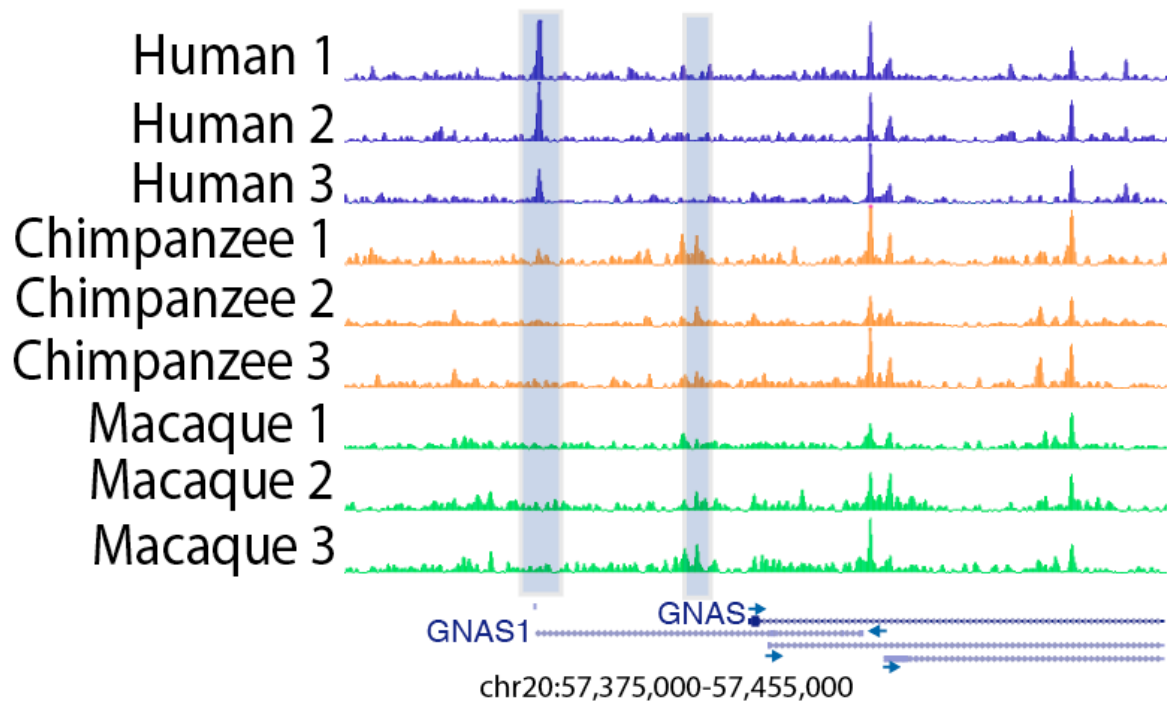

Supplement: Figure S12 — Potential regulatory element shuffling associated with an imprinted gene. GNAS is an imprinted gene that is regulated by the GNAS1 antisense transcript. A human DHS gain is located at the 3′ end of the GNAS1 gene, and human DHS loss in the intron of GNAS1 (upstream of GNAS). (PDF) [file pgen.1002789.s013.pdf]

Human DHS gain

Human DHS loss

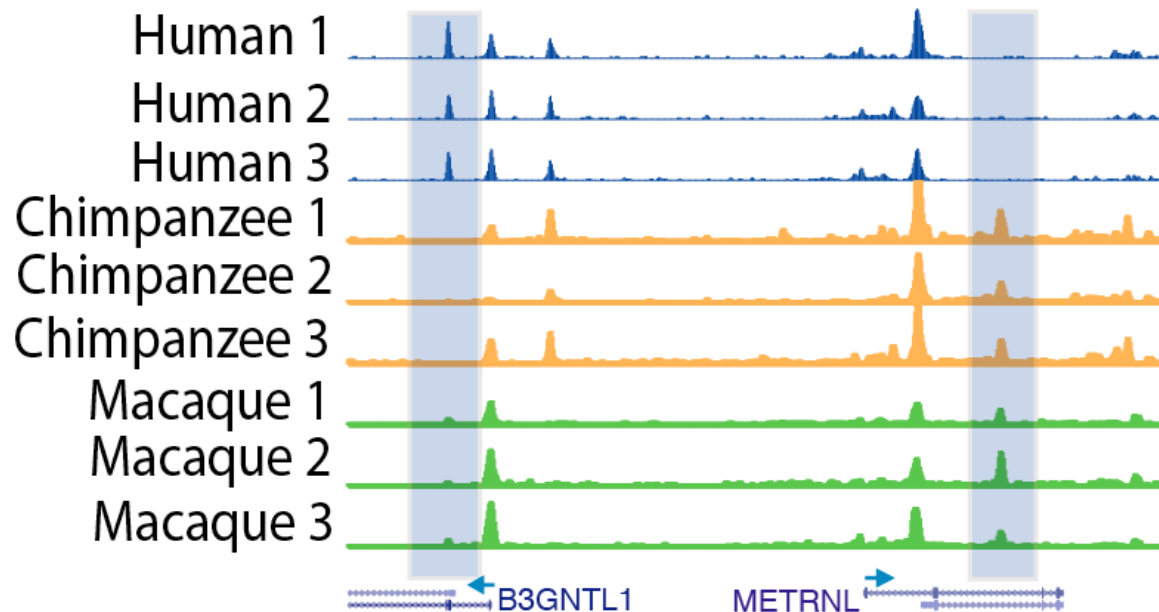

chr17:80,999,000-81,060,000

Supplement: Figure S13 — Potential regulatory element shuffling associated with alternative promoter and glial cell formation. Human DHS loss associated with meteorin-like (METRNL), a gene associated with glial cell formation. Nearby is a human DHS gain near an alternative promoter of the BG3NTL1, a gene with putative glycosyltransferase activity. Also note a great ape specific DHS between METRNL/BG2NTL1 genes (present in chimpanzee/human, but absent in macaque). (PDF) [file pgen.1002789.s014.pdf]

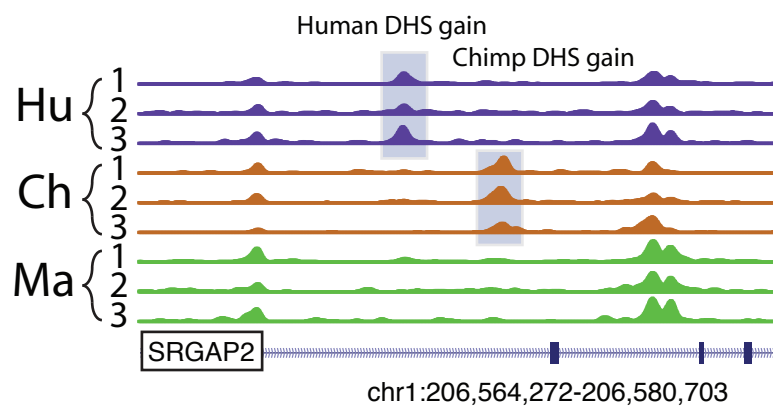

Supplement: Figure S14 — Potential case of an independently gained human and chimpanzee DHS sites associated with neuronal guidance. Human DHS gain found nearby (<50 kb) a chimpanzee DHS gain. Both mapped within an intron of the SRGAP2, a gene associated with neuronal guidance during brain development. (PDF) [file pgen.1002789.s015.pdf]

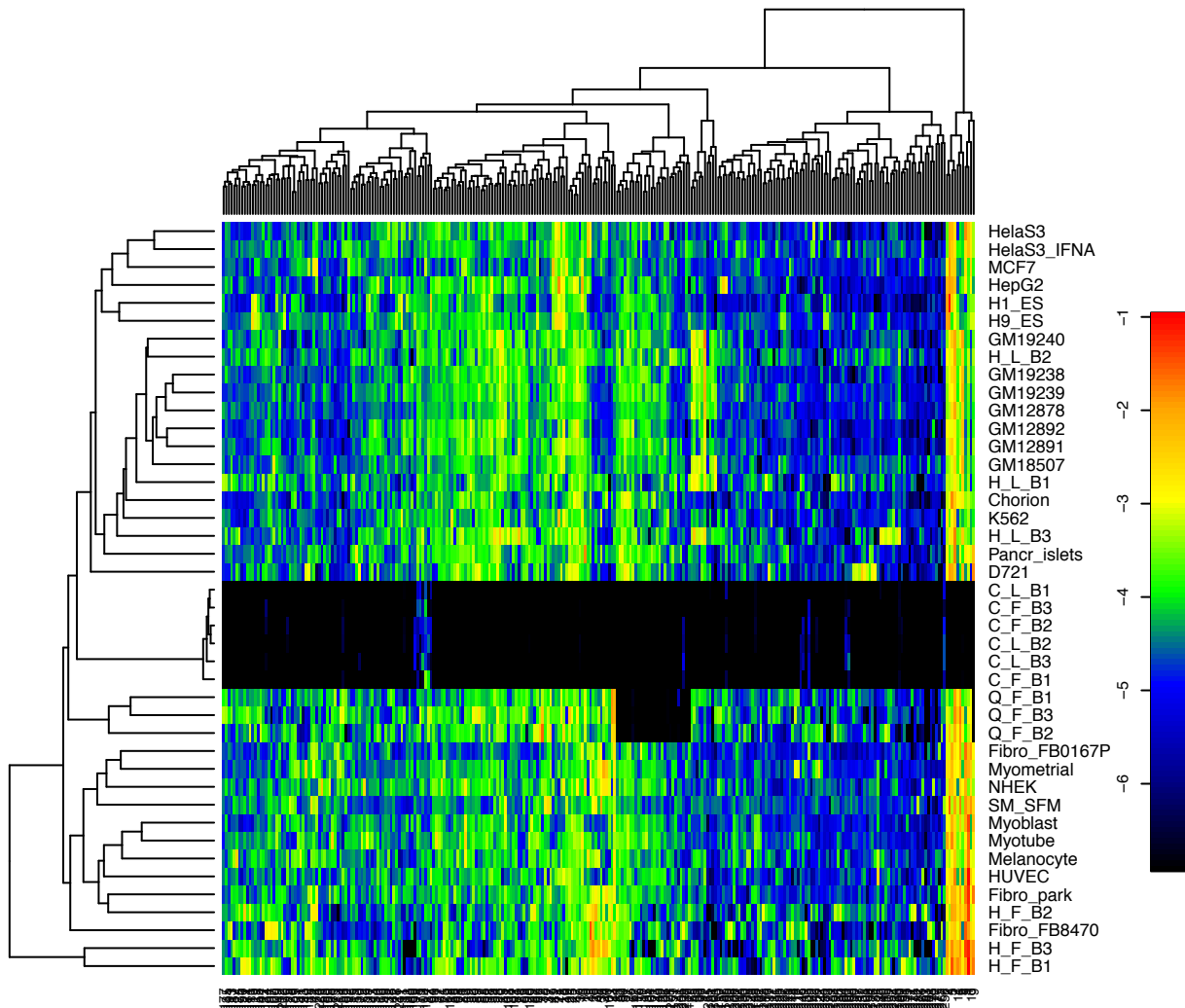

Supplement: Figure S15 — Chimp Condel (cCondel) regions heatmap. Shown are DNase-seq signal intensities (maximum parzen scores) of the 344 cCONDEL regions. The color scale (log) ranges from red, representing the highest signal intensities, down to black, representing little/no signal intensity. All 15 primate samples (fibroblasts and LCLs) used in our DNase-seq analysis and 27 different ENCODE cell lines are represented. Ubiquitous DHS sites that overlap cCONDELs are clustered on the right, and more common DHS sites are found in the middle. Note that these regions are do not have signal in chimpanzee data (C_L or C_F samples), and a small number also do not contain signal in macaque data (Q_F samples). (PDF) [file pgen.1002789.s016.pdf]
