# Supplementary material for: Extensive Evolutionary Changes in Regulatory Element Activity during Human Origins Are Associated with Altered Gene Expression and Positive Selection
Source: PLoS Genet. 2012 Jun 28;8(6):e1002789. doi: 10.1371/journal.pgen.1002789 (PMC3386175; doi:10.1371/journal.pgen.1002789)
Supplement: Figure S7 — Species-specific upregulated/downregulated gene expression levels are correlated with species-specific DHS gains/losses, respectively. (a) 48 Human upregulated genes (HumanExpUp) intersect genes that were located closest to human DHS gains (yellow arrow), which is higher than random permutations. 6 Human upregulated genes (HumanExpUp) overlap with genes located closest to human DHS losses (blue arrow), which is less than random permutations. (b) Comparison of upregulated genes (expression gains) and downregulated genes (expression losses) to genes located nearest to DHS gains and losses. P values were derived from 1000 random permutations (Materials and Methods). (PDF) [file pgen.1002789.s008.pdf]

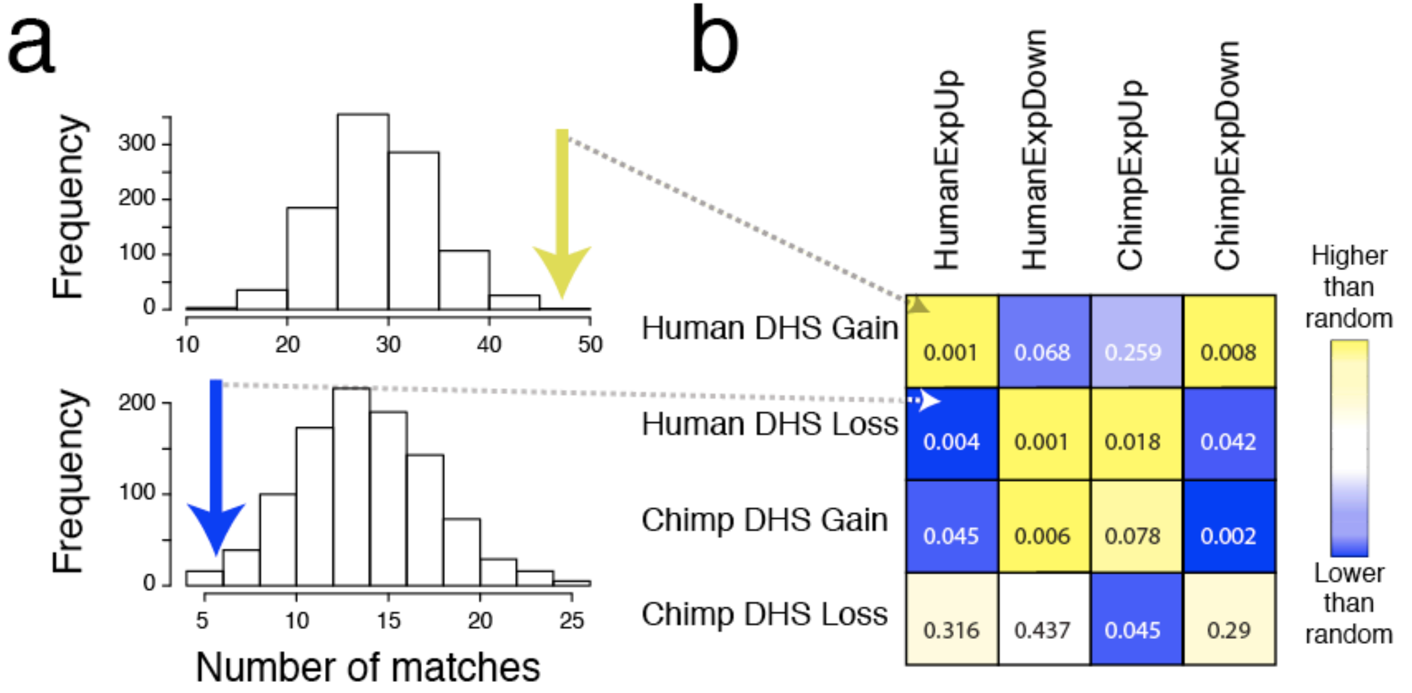
a
b
Frequency
0
100
200
300
10
20
30
40
50
Frequency
0
100
200
5
10
15
20
25
Number of matches
HumanExpUp
HumanExpDown
ChimpExpUp
ChimpExpDown
Human DHS Gain
0.001
0.068
0.259
0.008
Human DHS Loss
0.004
0.001
0.018
0.042
Chimp DHS Gain
0.045
0.006
0.078
0.002
Chimp DHS Loss
0.316
0.437
0.045
0.29
Higher than random
Lower than random
