# Supplementary material for: Extensive Evolutionary Changes in Regulatory Element Activity during Human Origins Are Associated with Altered Gene Expression and Positive Selection
Source: PLoS Genet. 2012 Jun 28;8(6):e1002789. doi: 10.1371/journal.pgen.1002789 (PMC3386175; doi:10.1371/journal.pgen.1002789)
Supplement: Figure S10 — Potential regulatory element shuffling: Number of instances (within 50 k) where fibroblast human DHS gain, human DHS loss, chimpanzee DHS gain, chimpanzee DHS loss, or Common regions map other nearby species-specific gains, losses, or common regions. This plot shows the number of DHS sites that fall within 50 kb of each other for each pairwise comparison. It also shows a P value for the significance of the overlap. To calculate the P values (upper number in each box), we used a permutation test to compare the number of true overlaps (lower number in each box) to the number of expected overlaps with a random set of DHS sites of matching size. Significant enrichments are bright yellow and depletions bright blue, with both significance and count shown in the appropriate box. The significance levels are limited by the number of permutations (1000), so a 0 indicates a permutation P value <10∧-3. We find insignificant or only marginally significant overlaps in most comparisons; for example, comparing human DHS gains to human DHS losses: human DHS gains are not located near human DHS losses more often than expected by chance. The same is true for chimp gains and losses. However, we do see more significance when comparing human DHS gains to chimp DHS losses, and chimp DHS gains to human DHS losses. This indicates that these regions tend to be located near one another more often than expected by chance. (PDF) [file pgen.1002789.s011.pdf]

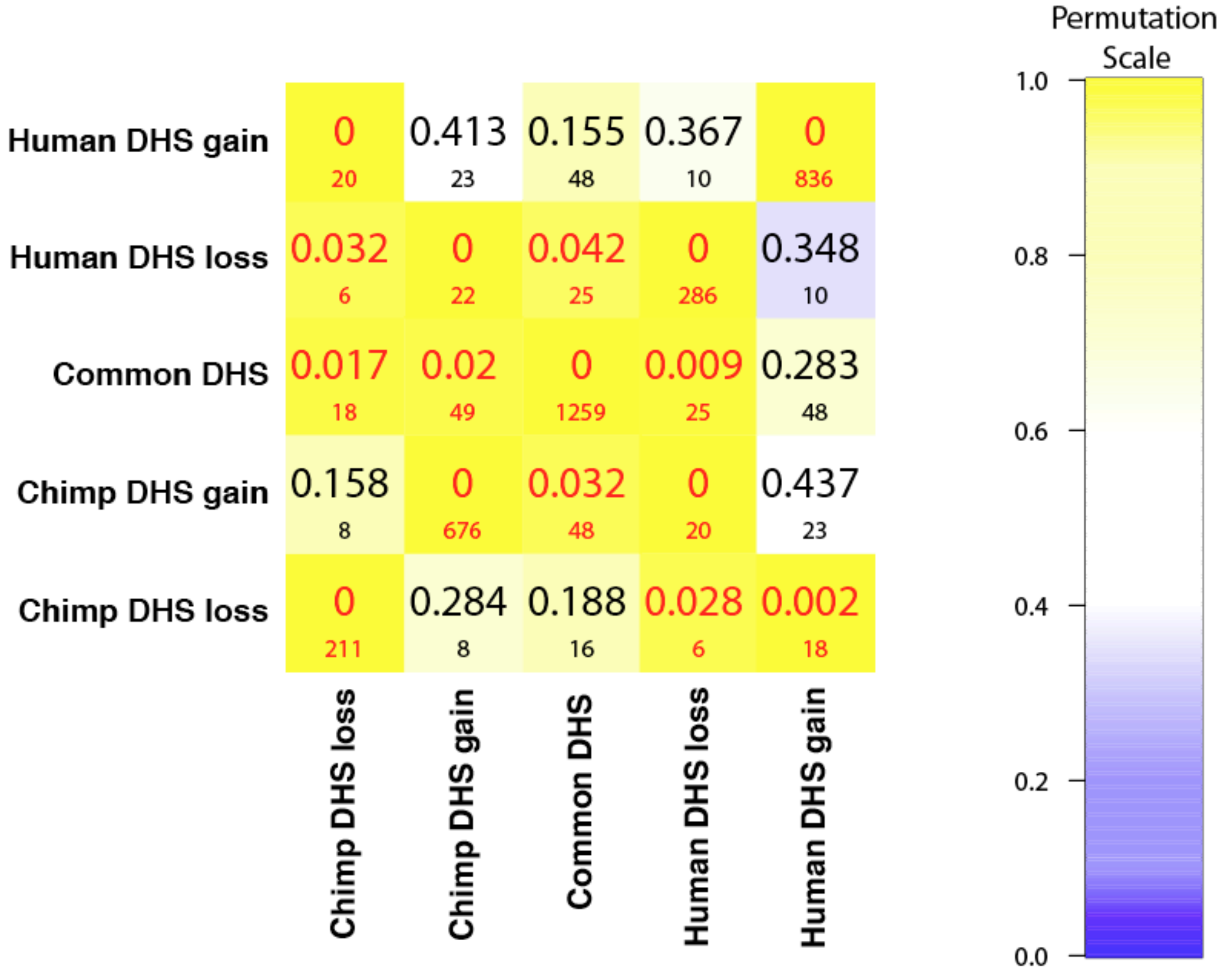

Permutation Scale
1.0
0.8
0.6
0.4
0.2
0.0
Human DHS gain
0 20
0.413 23
0.155 48
0.367 10
0 836
Human DHS loss
0.032 6
0 22
0.042 25
0 286
0.348 10
Common DHS
0.017 18
0.02 49
0 1259
0.009 25
0.283 48
Chimp DHS gain
0.158 8
0 676
0.032 48
0 20
0.437 23
Chimp DHS loss
0 211
0.284 8
0.188 16
0.028 6
0.002 18
Chimp DHS loss
Chimp DHS gain
Common DHS
Human DHS loss
Human DHS gain
