# Supplementary material for: Extensive Evolutionary Changes in Regulatory Element Activity during Human Origins Are Associated with Altered Gene Expression and Positive Selection
Source: PLoS Genet. 2012 Jun 28;8(6):e1002789. doi: 10.1371/journal.pgen.1002789 (PMC3386175; doi:10.1371/journal.pgen.1002789)
Supplement: Table S1 — Samples used for DNase-seq and DGE-seq analyses. Basic sequence count statistics are provided with each sample. (PDF) [file pgen.1002789.s017.pdf]

Sample ID	Species	Age (yr)	Gender	Coriell Catalog No.	Cell Type	Site of biopsy	DNase-seq, Total # of sequences (in hg19 space)	DNase-seq, % genome coverage of top100k peaks	DNase-seq, Median # of sequences per DHS site (top100k peaks)	DGE-seq, Total # of sequences
**HF1**	Human	12	M	AG16408	Lymphoblastoid	blood, peripheral vein	29,332,310	1.89%	51	10,950,474
**HL1**				AG16409	Primary Dermal Fibroblast	skin, unspecified	37,624,821	2.13%	26	8,876,138
**HF2**	Human	36	M	GM02184	Lymphoblastoid	blood, peripheral vein	30,272,133	2.2%	29	5,701,620
**HL2**				GM02185	Primary Dermal Fibroblast	skin, unspecified	25,253,295	1.77%	31	9,960,440
**HF3**	Human	48	F	GM05920	Lymphoblastoid	blood, peripheral vein	28,414,314	2.73%	25	6,729,690
**HL3**				GM05879	Primary Dermal Fibroblast	skin, upper arm	17,998,960	1.82%	40	8,470,902
**CF1**	Pan troglodytes	9	M	S003648	Lymphoblastoid	blood, peripheral vein	29,343,610	3.02%	55	4,223,955
**CL1**				S003649	Primary Dermal Fibroblast	skin, unspecified*	34,974,357	2.4%	50	9,711,114
**CF2**	Pan troglodytes	14	M	S003623	Lymphoblastoid	blood, peripheral vein	41,226,270	1.9%	94	5,788,846
**CL2**				S003624	Primary Dermal Fibroblast	skin, unspecified*	59,866,736	3.07%	33	10,349,784
**CF3**	Pan troglodytes	16	M	S007602	Lymphoblastoid	blood, peripheral vein	20,329,252	1.64%	33	4,721,760
**CL3**				S007603	Primary Dermal Fibroblast	skin, unspecified*	24,840,265	2.03%	17	7,420,880
**MF1**	Macaca mulatta	5	F	AG06252	Primary Dermal Fibroblast	skin, upper arm	29,330,217	2.04%	36	3,626,144
**MF2**	Macaca mulatta	1	M	AG08305	Primary Dermal Fibroblast	skin, upper arm	31,937,760	2.49%	50	5,142,449
**MF3**	Macaca mulatta	1	M	AG08308	Primary Dermal Fibroblast	skin, upper arm	20,324,578	1.99%	29	7,004,730

Standard location of skin punch biopsy for chimpanzees at Yerkes Regional Primate Research Center is the ear pinna.
