# Supplementary material for: Extensive Evolutionary Changes in Regulatory Element Activity during Human Origins Are Associated with Altered Gene Expression and Positive Selection
Source: PLoS Genet. 2012 Jun 28;8(6):e1002789. doi: 10.1371/journal.pgen.1002789 (PMC3386175; doi:10.1371/journal.pgen.1002789)
Supplement: Table S2 — DNase-seq data is validated by DNase-chip. DNase-chip libraries from the 15 samples were hybridized to 1% ENCODE DNA arrays of the corresponding species type. Probes consisted of segments of DNA sequences matching reference sequence builds hg18 (human), panTro2 (chimpanzee), and rheMac2 (macaque), respectively. The array intensities were compiled and significant DHS sites were called using ChIPotle (P<0.000001 peak cutoff). We intersected the DNase-chip sites from each of the 3 individuals samples for each species and determined the amount that overlap DNase-seq data. The top 100,000 F-seq called sites were used from each DNase-seq sample for this comparison. (PDF) [file pgen.1002789.s018.pdf]

Sample ID	Percent Overlap between DNase-chip and DNase-seq
HF1	71.28
HF2	71.60
HF3	73.31
CF1	79.00
CF2	87.15
CF3	77.12
MF1	87.76
MF2	87.93
MF3	82.41
HL1	79.23
HL2	85.29
HL3	93.57
CL1	91.54
CL2	79.23
CL3	84.23
