# Supplementary material for: Extensive Evolutionary Changes in Regulatory Element Activity during Human Origins Are Associated with Altered Gene Expression and Positive Selection
Source: PLoS Genet. 2012 Jun 28;8(6):e1002789. doi: 10.1371/journal.pgen.1002789 (PMC3386175; doi:10.1371/journal.pgen.1002789)
Supplement: Table S3 — Human cell types analyzed by DNase-seq for the ENCODE project by our group. All data is available on the UCSC genome browser (http://genome.ucsc.edu/) and more specifically at: http://genome.ucsc.edu/cgi-bin/hgTrackUi?hgsid=246298273&c=chr21&g=wgEncodeOpenChromDnase (PDF) [file pgen.1002789.s019.pdf]

UCSC Name	Additional Description	Sex
Chorion	Chorion	F
Medullo	Medulloblastoma (D721)	M
ProgFib	Fibroblast from progeria patient (FB0167P)	M
Fibrobl	Fibroblast (FB8470)	F
FibroP	Fibroblast from parkinson's patients	M(B1), F(B2), F(B3)
GM12878	Lymphoblastoid	F
GM12891	Lymphoblastoid	M
GM12892	Lymphoblastoid	F
GM18507	Lymphoblastoid	M
GM19238	Lymphoblastoid	F
GM19239	Lymphoblastoid	M
GM19240	Lymphoblastoid	F
H1-hESC	H1 Embryonic Stem Cells	M
H9-hESC	H9 Embryonic Stem Cells	F
HeLa-S3	cervical carcinoma	F
HeLa-S3	cervical carcinoma induced with interferon alpha (IFNa4h)	F
HepG2	hepatocellular carcinoma	M
HUVEC	human umbilical vein endothelial cell	M
K562	chronic myeloid leukemia	F
MCF-7	epithelial breast adenocarcinoma	F
Melano	Melanocyte, cat 2200, NHM22=lot#1002, NHM23=lot#1014	M
HSMM	CM33j(B1), CM01-201-001(B2), CM01-154-002(B3), CM01-135-003(B4)	F(B1), F(B2), M(B3), F(B4)
Myometr	myometrial	F
HSMMtube	CM33j(B1), CM01-201-001(B2), CM01-154-002(B3), CM01-135-003(B4)	F(B1), F(B2), M(B3), F(B4)
NHEK	Normal Human Epidermal Keratinocyte	F
PanIslets	VGP024(B1), VJH020(B2), VKJ113(B3)	M(B1), F(B2), M(B3)
AoSMC	Smooth_muscle, Serum Free (SM_SFM)	M
