# Supplementary material for: Extensive Evolutionary Changes in Regulatory Element Activity during Human Origins Are Associated with Altered Gene Expression and Positive Selection
Source: PLoS Genet. 2012 Jun 28;8(6):e1002789. doi: 10.1371/journal.pgen.1002789 (PMC3386175; doi:10.1371/journal.pgen.1002789)
Supplement: Table S4 — Percent overlap of human- and chimpanzee- DHS gains/losses/common detected in fibroblasts with DHS sites identified in ENCODE human cell types. FibroP, Fibrobl, and ProgFib cells were independently derived fibroblasts samples. (PDF) [file pgen.1002789.s020.pdf]

	human DHS gains (%)	human DHS losses (%)	common DHS (%)	chimpanzee DHS gains (%)	chimpanzee DHS losses (%)
**FibroP**	79.07%	22.38%	99.60%	15.98%	73.46%
**Fibrobl**	71.53%	11.19%	93.57%	8.28%	73.46%
**ProgFib**	73.21%	26.57%	99.29%	17.16%	72.99%
**Myometrial**	66.99%	23.78%	97.38%	16.86%	67.30%
**AoSMC**	52.27%	37.76%	98.97%	23.08%	56.87%
**Myoblast**	43.90%	18.88%	92.61%	15.53%	52.13%
**NHEK**	46.05%	19.58%	89.44%	11.24%	54.98%
**Melanocyte**	45.33%	9.79%	93.57%	9.76%	47.39%
**Myotube**	42.22%	21.33%	89.20%	17.46%	52.61%
**HUVEC**	25.36%	11.19%	85.70%	10.65%	37.44%
**Chorion**	23.33%	19.58%	74.90%	13.17%	38.86%
**HelaS3**	23.33%	20.28%	83.80%	13.31%	39.81%
**PanIslets**	19.74%	15.38%	80.46%	12.57%	31.28%
**HelaS3_IFNA**	27.39%	19.93%	84.43%	14.94%	40.28%
**GM19240**	8.85%	11.54%	73.71%	7.69%	18.01%
**HepG2**	9.81%	15.73%	76.89%	10.21%	28.91%
**GM19238**	9.21%	11.19%	73.31%	7.84%	18.01%
**GM12878**	12.20%	7.69%	74.98%	7.25%	19.43%
**GM19239**	8.97%	11.54%	72.44%	7.84%	20.85%
**GM18507**	7.89%	7.69%	71.01%	5.18%	16.11%
**MCF7**	13.04%	12.94%	76.97%	9.32%	29.38%
**H9_ES**	8.85%	16.08%	73.71%	7.54%	19.91%
**GM12892**	11.00%	15.73%	71.64%	12.43%	20.38%
**GM12891**	10.05%	12.94%	72.36%	10.21%	19.91%
**K562**	11.36%	15.38%	73.23%	8.88%	23.22%
**Medullo**	6.82%	9.79%	66.16%	7.10%	17.06%
**H1_ES**	7.89%	13.99%	69.10%	9.62%	21.80%
