# Supplementary material for: Extensive Evolutionary Changes in Regulatory Element Activity during Human Origins Are Associated with Altered Gene Expression and Positive Selection
Source: PLoS Genet. 2012 Jun 28;8(6):e1002789. doi: 10.1371/journal.pgen.1002789 (PMC3386175; doi:10.1371/journal.pgen.1002789)
Supplement: Table S5 — Percent overlap of human-DHS gains/losses/common detected in lymphoblasts with DHS sites identified in ENCODE human cell types. Note high degree of overlap with independently derived LCLs (GM). (PDF) [file pgen.1002789.s021.pdf]

	human DHS gains (%)	human DHS losses (%)	common DHS (%)
**GM12878**	96.12%	16.02%	99.94%
**GM19239**	96.12%	14.92%	99.87%
**GM19240**	93.20%	14.36%	99.87%
**GM18507**	92.23%	12.15%	99.43%
**GM19238**	92.23%	19.34%	99.87%
**GM12891**	85.44%	20.99%	99.43%
**GM12892**	85.44%	21.55%	97.79%
**Myotube**	37.86%	20.99%	77.95%
**Chorion**	36.89%	23.76%	65.70%
**HelaS3**	35.92%	21.55%	77.38%
**HelaS3_IFNA**	35.92%	17.68%	77.20%
**Myometrial**	35.92%	18.23%	76.25%
**MCF7**	33.98%	13.81%	74.48%
**Fibroblasts_park**	33.01%	16.57%	80.48%
**HUVEC**	33.01%	21.55%	79.91%
**Myoblast**	33.01%	23.20%	78.33%
**Pancreatic_islets**	33.01%	20.44%	78.02%
**FB0167P**	32.04%	16.57%	77.57%
**H1_ES**	32.04%	24.86%	72.46%
**NHEK**	32.04%	15.47%	77.26%
**H9_ES**	31.07%	17.68%	74.48%
**D721**	30.10%	12.71%	69.36%
**K562**	29.13%	19.89%	78.27%
**Melanocyte**	29.13%	12.15%	77.83%
**HepG2**	28.16%	18.23%	75.99%
**FB8470**	27.18%	10.50%	72.52%
**SM_SFM**	23.30%	11.60%	75.05%
