# Supplementary material for: Extensive Evolutionary Changes in Regulatory Element Activity during Human Origins Are Associated with Altered Gene Expression and Positive Selection
Source: PLoS Genet. 2012 Jun 28;8(6):e1002789. doi: 10.1371/journal.pgen.1002789 (PMC3386175; doi:10.1371/journal.pgen.1002789)
Supplement: Table S6 — Fisher's exact test for Histone modification and CTCF ChIP-seq peaks that intersect LCL Human DHS gains/losses/commons. (PDF) [file pgen.1002789.s022.pdf]

	Human DHS gain vs loss	Human DHS gain vs common	Human DHS loss vs common
**K4me1**	<2.2e-16	<2.2e-16	0.02426
**K4me2**	1.529e-15	0.2836	<2.2e-16
**K4me3**	3.459e-12	0.5739	<2.2e-16
**K9me3**	0.0005996	0.04043	0.008462
**K27me3**	0.03788	0.6775	0.0001605
**K27ac**	3.728e-12	0.3278	<2.2e-16
**CTCF**	4.34e-07	0.00914	<2.2e-16
