# Supplementary material for: Extensive Evolutionary Changes in Regulatory Element Activity during Human Origins Are Associated with Altered Gene Expression and Positive Selection
Source: PLoS Genet. 2012 Jun 28;8(6):e1002789. doi: 10.1371/journal.pgen.1002789 (PMC3386175; doi:10.1371/journal.pgen.1002789)
Supplement: Table S7 — Relationship between species-specific DHS sites and differentially expressed genes. Species-specific DHS sites (Human DHS gain/loss and Chimpanzee DHS gain/loss regions) for the fibroblast cell type were compared to the expression state of the closest gene (human up/down and chimp up/down expressed genes). (PDF) [file pgen.1002789.s023.pdf]

a **Number of overlap between species-specific DNaseHS and Expression**

	Human DHS gain	Human DHS loss	Chimpanzee DHS gain	Chimpanzee DHS loss
Human upregulated	58	5	23	14
Human downregulated	17	28	43	10
Chimpanzee upregulated	16	16	37	3
Chimpanzee downregulated	42	3	13	12

b **Likelihood of finding LESS matches by random chance (permuted 100,000x)**

	Human DHS gain	Human DHS loss	Chimpanzee DHS gain	Chimpanzee DHS loss
Human upregulated	>0.999	0.002	0.093	0.769
Human downregulated	0.008	>0.999	>0.999	0.472
Chimpanzee upregulated	0.018	0.95	0.999	0.009
Chimpanzee downregulated	0.999	0.002	0.013	0.861

c **Likelihood of finding MORE matches by random chance (permuted 100,000x)**

	Human DHS gain	Human DHS loss	Chimpanzee DHS gain	Chimpanzee DHS loss
Human upregulated	<0.001	0.998	0.907	0.231
Human downregulated	0.992	<0.001	<0.001	0.528
Chimpanzee upregulated	0.982	0.05	0.001	0.991
Chimpanzee downregulated	0.001	0.998	0.987	0.139
