# Supplementary material for: Extensive Evolutionary Changes in Regulatory Element Activity during Human Origins Are Associated with Altered Gene Expression and Positive Selection
Source: PLoS Genet. 2012 Jun 28;8(6):e1002789. doi: 10.1371/journal.pgen.1002789 (PMC3386175; doi:10.1371/journal.pgen.1002789)
Supplement: Table S8 — Relationship between differentially expressed species-specific genes (human DHS gains/losses, chimpanzee DHS gains/losses) and the likelihood of finding a species-specific DHS site within a 20 kb window surrounding those genes. (PDF) [file pgen.1002789.s024.pdf]

a **Number of species-specific DNaseHS regions near Differentially Expressed genes**

	Human upregulated	Human downregulated	Chimpanzee upregulated	Chimpanzee downregulated
Human DHS gain	48	18	20	34
Human DHS loss	6	25	18	6
Chimpanzee DHS gain	21	38	29	11
Chimpanzee DHS loss	13	10	4	10

b **Likelihood of finding LESS matches by random chance (permuted 1000x)**

	Human upregulated	Human downregulated	Chimpanzee upregulated	Chimpanzee downregulated
Human DHS gain	0.999	0.068	0.259	0.992
Human DHS loss	0.004	0.999	0.982	0.042
Chimpanzee DHS gain	0.045	0.994	0.922	0.002
Chimpanzee DHS loss	0.684	0.437	0.045	0.71

c **Likelihood of finding MORE matches by random chance (permuted 1000x)**

	Human upregulated	Human downregulated	Chimpanzee upregulated	Chimpanzee downregulated
Human DHS gain	0.001	0.932	0.741	0.008
Human DHS loss	0.996	0.001	0.018	0.958
Chimpanzee DHS gain	0.955	0.006	0.078	0.998
Chimpanzee DHS loss	0.316	0.563	0.955	0.29
