# Supplementary material for: Extensive Evolutionary Changes in Regulatory Element Activity during Human Origins Are Associated with Altered Gene Expression and Positive Selection
Source: PLoS Genet. 2012 Jun 28;8(6):e1002789. doi: 10.1371/journal.pgen.1002789 (PMC3386175; doi:10.1371/journal.pgen.1002789)
Supplement: Table S9 — Gene Ontology enrichments associated with species-specific DHS gain/loss and common regions and human up- and down- regulated genes. (a) Gene ontology analysis tool, GREAT, was used to look for any enrichments of biological process or disease ontologies of genes associated with human and chimpanzee DHS gain/loss/common regions. (b) Genes identified as differentially expressed in human or chimpanzee fibroblast samples were analyzed for GO biological process enrichments using DAVID. A P value threshold of 0.001 was set as the minimum. (PDF) [file pgen.1002789.s025.pdf]

**a.**

| # Ontology | Term Name | Binom Rank | Binom Raw P-Value | Binom FDR Q-Val | Binom Fold Enrichment | Binom Observed Region Hits | Binom Region Set Coverage | Hyper Rank | Hyper FDR Q-Val | Hyper Fold Enrichment | Hyper Observed Gene Hits | Hyper Total Genes | Hyper Gene Set Coverage |
|---|---|---|---|---|---|---|---|---|---|---|---|---|---|
| | | | | | | | | | | | | | |
| **Human DHS gains (836 regions):** | | | | | | | | | | | | | |
| GO Biological Process | regulation of action potential in neuron | 7 | 0.00000763757 | 0.007823054 | 3.012161 | 22 | 0.02631579 | 115 | 0.007019542 | 3.128633 | 14 | 67 | 0.01192504 |
| GO Biological Process | axon ensheathment | 8 | 0.00000786052 | 0.007044991 | 3.636246 | 17 | 0.02033493 | 143 | 0.022543212 | 3.229415 | 11 | 51 | 0.009369676 |
| GO Biological Process | regulation of action potential | 18 | 0.0000417578 | 0.016633536 | 2.497522 | 25 | 0.02990431 | 79 | 0.003546283 | 2.994549 | 17 | 85 | 0.01448041 |
| GO Biological Process | cellular response to hydrogen peroxide | 22 | 0.0000726281 | 0.023670142 | 4.714695 | 10 | 0.01196172 | 139 | 0.018571156 | 4.277927 | 8 | 28 | 0.00681431 |
| GO Biological Process | myelination | 23 | 0.0000754788 | 0.023529683 | 3.306935 | 15 | 0.01794258 | 170 | 0.038235965 | 3.18569 | 10 | 47 | 0.008517888 |
| GO Biological Process | regulation of striated muscle tissue development | 29 | 0.000137597 | 0.034019722 | 2.411843 | 23 | 0.02751196 | 89 | 0.004115235 | 3.539012 | 13 | 55 | 0.01107325 |
| GO Biological Process | regulation of muscle organ development | 33 | 0.000168307 | 0.036568564 | 2.377772 | 23 | 0.02751196 | 101 | 0.005400322 | 3.414836 | 13 | 57 | 0.01107325 |
| *No significant Disease processes identified | | | | | | | | | | | | | |
| | | | | | | | | | | | | | |
| **Human DHS losses (286 regions):** | | | | | | | | | | | | | |
| **No significant Biological processes identified* | | | | | | | | | | | | | |
| **No significant Disease processes identified* | | | | | | | | | | | | | |
| | | | | | | | | | | | | | |
| **Chimpanzee DHS gains (676 regions):** | | | | | | | | | | | | | |
| **No significant Biological processes identified* | | | | | | | | | | | | | |
| Disease Ontology | colonic neoplasm | 1 | 0.00000302625 | 0.005477516 | 2.121369 | 45 | 0.06656805 | 14 | 0.009633704 | 1.997111 | 35 | 292 | 0.03317536 |
| Disease Ontology | malignant tumor of colon | 2 | 0.0000124607 | 0.011276943 | 2.062751 | 42 | 0.06213018 | 29 | 0.019480787 | 1.917883 | 32 | 278 | 0.03033175 |
| Disease Ontology | habitual abortion | 6 | 0.000093841 | 0.028308705 | 11.37484 | 5 | 0.00739645 | 7 | 0.009590983 | 10.41351 | 5 | 8 | 0.004739336 |
| | | | | | | | | | | | | | |
| **Chimpanzee DHS losses (211 regions):** | | | | | | | | | | | | | |
| **No significant Biological processes identified* | | | | | | | | | | | | | |
| **No significant Disease processes identified* | | | | | | | | | | | | | |
| | | | | | | | | | | | | | |
| **Common DHS (1259 regions):** | | | | | | | | | | | | | |
| **No significant Biological processes identified* | | | | | | | | | | | | | |
| **No significant Disease processes identified* | | | | | | | | | | | | | |
| | | | | | | | | | | | | | |
| # GREAT version 1.8 | Species assembly: hg19 | Association rule: Basal+extension: 5000 bp upstream, 1000 bp downstream, 1000000 bp max extension, curated regulatory domains included | | | | | | | | | | | |

| b. | | | | | | | | |
|---|---|---|---|---|---|---|---|---|
| Category | Term | Count | % | PValue | Fold Enrichment | Bonferroni | Benjamini | FDR |
| | | | | | | | | |
| **Human Upregulated genes:** | | | | | | | | |
| GOTERM_BP_FAT | GO:0007155~cell adhesion | 53 | 6.95 | 0.000000715 | 2.08 | 0.001611697 | 0.001611697 | 0.00124656 |
| GOTERM_BP_FAT | GO:0022610~biological adhesion | 53 | 6.95 | 0.000000726 | 2.07 | 0.001637286 | 0.000819 | 0.001266367 |
| GOTERM_BP_FAT | GO:0016337~cell-cell adhesion | 24 | 3.15 | 0.000191 | 2.39 | 0.350739019 | 0.13408931 | 0.333243188 |
| GOTERM_BP_FAT | GO:0007156~homophilic cell adhesion | 14 | 1.83 | 0.000966 | 2.93 | 0.886936744 | 0.420130359 | 1.67050329 |
| | | | | | | | | |
| **Human Downregulated genes:** | | | | | | | | |
| GOTERM_BP_FAT | GO:0008585~female gonad development | 12 | 1.42 | 0.000103 | 4.23 | 0.246204589 | 0.246204589 | 0.18269255 |
| GOTERM_BP_FAT | GO:0046545~development of primary female sexual characteristics | 12 | 1.42 | 0.000206 | 3.92 | 0.432997289 | 0.247004176 | 0.366419612 |
| GOTERM_BP_FAT | GO:0046660~female sex differentiation | 12 | 1.42 | 0.000206 | 3.92 | 0.432997289 | 0.247004176 | 0.366419612 |
| GOTERM_BP_FAT | GO:0042127~regulation of cell proliferation | 57 | 6.75 | 0.000279 | 1.63 | 0.536759885 | 0.226247521 | 0.496622618 |
| GOTERM_BP_FAT | GO:0022602~ovulation cycle process | 11 | 1.30 | 0.00036 | 4.00 | 0.629831591 | 0.219990597 | 0.64090748 |
| GOTERM_BP_FAT | GO:0043523~regulation of neuron apoptosis | 13 | 1.54 | 0.000591 | 3.26 | 0.804204021 | 0.278293272 | 1.049478067 |
| GOTERM_BP_FAT | GO:0042698~ovulation cycle | 11 | 1.30 | 0.000682 | 3.70 | 0.847437036 | 0.269015469 | 1.209074752 |
| GOTERM_BP_FAT | GO:0035295~tube development | 22 | 2.60 | 0.000775 | 2.25 | 0.8821305 | 0.26321112 | 1.373840222 |
| | | | | | | | | |
| **Chimpanzee Upregulated genes:** | | | | | | | | |
| GOTERM_BP_FAT | GO:0045686~negative regulation of glial cell differentiation | 6 | 0.84 | 0.0000213 | 15.34 | 0.054817189 | 0.054817189 | 0.037834948 |
| GOTERM_BP_FAT | GO:0014014~negative regulation of gliogenesis | 6 | 0.84 | 0.0000213 | 15.34 | 0.054817189 | 0.054817189 | 0.037834948 |
| GOTERM_BP_FAT | GO:0045685~regulation of glial cell differentiation | 7 | 0.98 | 0.000049 | 9.84 | 0.121648588 | 0.06279596 | 0.087026886 |
| GOTERM_BP_FAT | GO:0032101~regulation of response to external stimulus | 18 | 2.53 | 0.0000497 | 3.18 | 0.123219374 | 0.042886045 | 0.088227299 |
| GOTERM_BP_FAT | GO:0014013~regulation of gliogenesis | 7 | 0.98 | 0.0000666 | 9.37 | 0.161536038 | 0.043089984 | 0.118190582 |
| GOTERM_BP_FAT | GO:0048713~regulation of oligodendrocyte differentiation | 5 | 0.70 | 0.000171 | 15.62 | 0.364044143 | 0.086548708 | 0.303357002 |
| GOTERM_BP_FAT | GO:0043062~extracellular structure organization | 17 | 2.39 | 0.000225 | 2.93 | 0.448228735 | 0.094351136 | 0.398334855 |
| GOTERM_BP_FAT | GO:0048715~negative regulation of oligodendrocyte differentiation | 4 | 0.56 | 0.00082 | 18.75 | 0.885656722 | 0.266401115 | 1.445061963 |
| | | | | | | | | |
| **Chimpanzee Downregulated genes:** | | | | | | | | |
| GOTERM_BP_FAT | GO:0040012~regulation of locomotion | 21 | 2.89 | 0.0000606 | 2.80 | 0.153130469 | 0.153130469 | 0.108032715 |
| GOTERM_BP_FAT | GO:0051270~regulation of cell motion | 21 | 2.89 | 0.0000652 | 2.79 | 0.163766918 | 0.085542193 | 0.116243241 |
| GOTERM_BP_FAT | GO:0030334~regulation of cell migration | 19 | 2.62 | 0.000105 | 2.88 | 0.250742864 | 0.091739774 | 0.187557766 |
| GOTERM_BP_FAT | GO:0035108~limb morphogenesis | 14 | 1.93 | 0.000118 | 3.62 | 0.276560071 | 0.077745849 | 0.210316176 |
| GOTERM_BP_FAT | GO:0035107~appendage morphogenesis | 14 | 1.93 | 0.000118 | 3.62 | 0.276560071 | 0.077745849 | 0.210316176 |
| GOTERM_BP_FAT | GO:0035113~embryonic appendage morphogenesis | 13 | 1.79 | 0.000133 | 3.83 | 0.305963752 | 0.070442133 | 0.237240307 |
| GOTERM_BP_FAT | GO:0030326~embryonic limb morphogenesis | 13 | 1.79 | 0.000133 | 3.83 | 0.305963752 | 0.070442133 | 0.237240307 |
| GOTERM_BP_FAT | GO:0048736~appendage development | 14 | 1.93 | 0.000177 | 3.48 | 0.385015169 | 0.077830543 | 0.315665555 |
| GOTERM_BP_FAT | GO:0060173~limb development | 14 | 1.93 | 0.000177 | 3.48 | 0.385015169 | 0.077830543 | 0.315665555 |
| GOTERM_BP_FAT | GO:0009954~proximal/distal pattern formation | 7 | 0.96 | 0.000195 | 7.80 | 0.413349516 | 0.073359351 | 0.346239237 |
| GOTERM_BP_FAT | GO:0001944~vasculature development | 22 | 3.03 | 0.000837 | 2.25 | 0.899154027 | 0.249315722 | 1.480895953 |
| | | | | | | | | |
| **Common genes:** | | | | | | | | |
| GOTERM_BP_FAT | GO:0044265~cellular macromolecule catabolic process | 97 | 7.38 | 0.000000000128 | 1.97 | 0.000000361 | 0.000000361 | 0.000000229 |
| GOTERM_BP_FAT | GO:0009057~macromolecule catabolic process | 100 | 7.60 | 0.000000000736 | 1.88 | 0.00000207 | 0.00000104 | 0.00000132 |
| GOTERM_BP_FAT | GO:0006350~transcription | 210 | 15.97 | 0.00000000183 | 1.47 | 0.00000517 | 0.00000172 | 0.00000328 |
| GOTERM_BP_FAT | GO:0034470~ncRNA processing | 37 | 2.81 | 0.0000000104 | 2.91 | 0.0000294 | 0.00000734 | 0.0000186 |
| GOTERM_BP_FAT | GO:0034660~ncRNA metabolic process | 42 | 3.19 | 0.0000000104 | 2.68 | 0.0000294 | 0.00000588 | 0.0000186 |
| GOTERM_BP_FAT | GO:0006974~response to DNA damage stimulus | 56 | 4.26 | 0.0000000384 | 2.21 | 0.000108 | 0.0000181 | 0.0000688 |
| GOTERM_BP_FAT | GO:0006396~RNA processing | 73 | 5.55 | 0.0000000391 | 1.96 | 0.00011 | 0.0000157 | 0.0000699 |
| GOTERM_BP_FAT | GO:0030163~protein catabolic process | 79 | 6.01 | 0.0000000854 | 1.87 | 0.000241 | 0.0000301 | 0.000153 |

| | | | | | | | | |
|---|---|---|---|---|---|---|---|---|
| GOTERM_BP_FAT | GO:0044257~cellular protein catabolic process | 77 | 5.86 | 0.0000000998 | 1.88 | 0.000281 | 0.0000313 | 0.000179 |
| GOTERM_BP_FAT | GO:0051603~proteolysis involved in cellular protein catabolic process | 76 | 5.78 | 0.000000174 | 1.86 | 0.000491 | 0.0000492 | 0.000312 |
| GOTERM_BP_FAT | GO:0006399~tRNA metabolic process | 26 | 1.98 | 0.000000278 | 3.24 | 0.000783 | 0.0000712 | 0.000497 |
| GOTERM_BP_FAT | GO:0045449~regulation of transcription | 237 | 18.02 | 0.000000456 | 1.34 | 0.001285375 | 0.000107 | 0.000816 |
| GOTERM_BP_FAT | GO:0019941~modification-dependent protein catabolic process | 72 | 5.48 | 0.000000565 | 1.84 | 0.001590863 | 0.000122 | 0.001009622 |
| GOTERM_BP_FAT | GO:0043632~modification-dependent macromolecule catabolic process | 72 | 5.48 | 0.000000565 | 1.84 | 0.001590863 | 0.000122 | 0.001009622 |
| GOTERM_BP_FAT | GO:0016568~chromatin modification | 42 | 3.19 | 0.00000143 | 2.25 | 0.004014057 | 0.000287 | 0.002550549 |
| GOTERM_BP_FAT | GO:0008033~tRNA processing | 19 | 1.44 | 0.00000238 | 3.67 | 0.006698326 | 0.000448 | 0.004261851 |
| GOTERM_BP_FAT | GO:0051276~chromosome organization | 61 | 4.64 | 0.00000429 | 1.85 | 0.012036859 | 0.000757 | 0.007679025 |
| GOTERM_BP_FAT | GO:0000184~nuclear-transcribed mRNA catabolic process, nonsense-mediated decay | 11 | 0.84 | 0.0000173 | 5.39 | 0.047573698 | 0.002863102 | 0.030904642 |
| GOTERM_BP_FAT | GO:0006402~mRNA catabolic process | 13 | 0.99 | 0.0000195 | 4.44 | 0.053524232 | 0.003051441 | 0.034877696 |
| GOTERM_BP_FAT | GO:0006259~DNA metabolic process | 60 | 4.56 | 0.0000312 | 1.74 | 0.0842638 | 0.004622281 | 0.055805503 |
| GOTERM_BP_FAT | GO:0033554~cellular response to stress | 65 | 4.94 | 0.0000378 | 1.69 | 0.101051332 | 0.005312307 | 0.067531309 |
| GOTERM_BP_FAT | GO:0000956~nuclear-transcribed mRNA catabolic process | 11 | 0.84 | 0.0000773 | 4.62 | 0.195893587 | 0.010328371 | 0.138161184 |
| GOTERM_BP_FAT | GO:0006325~chromatin organization | 47 | 3.57 | 0.0000839 | 1.83 | 0.21059191 | 0.01069116 | 0.149843012 |
| GOTERM_BP_FAT | GO:0006281~DNA repair | 38 | 2.89 | 0.000102 | 1.97 | 0.250134292 | 0.012437706 | 0.182376685 |
| GOTERM_BP_FAT | GO:0046907~intracellular transport | 71 | 5.40 | 0.000107 | 1.59 | 0.260573322 | 0.012499563 | 0.19125004 |
| GOTERM_BP_FAT | GO:0051726~regulation of cell cycle | 42 | 3.19 | 0.000137 | 1.86 | 0.320117681 | 0.015314937 | 0.244373244 |
| GOTERM_BP_FAT | GO:0008610~lipid biosynthetic process | 40 | 3.04 | 0.000335 | 1.82 | 0.611513914 | 0.03571203 | 0.597780671 |
| GOTERM_BP_FAT | GO:0006913~nucleocytoplasmic transport | 24 | 1.83 | 0.000353 | 2.26 | 0.630364845 | 0.036189613 | 0.629129683 |
| GOTERM_BP_FAT | GO:0008104~protein localization | 87 | 6.62 | 0.000385 | 1.45 | 0.661981653 | 0.037996974 | 0.685459086 |
| GOTERM_BP_FAT | GO:0051169~nuclear transport | 24 | 1.83 | 0.000425 | 2.23 | 0.6984702 | 0.040498031 | 0.757374729 |
| GOTERM_BP_FAT | GO:0015031~protein transport | 77 | 5.86 | 0.000432 | 1.48 | 0.703954049 | 0.039762555 | 0.768924949 |
| GOTERM_BP_FAT | GO:0008654~phospholipid biosynthetic process | 18 | 1.37 | 0.000487 | 2.59 | 0.746959377 | 0.043361021 | 0.867647415 |
| GOTERM_BP_FAT | GO:0006260~DNA replication | 27 | 2.05 | 0.000495 | 2.09 | 0.752623939 | 0.042712414 | 0.881878892 |
| GOTERM_BP_FAT | GO:0070647~protein modification by small protein conjugation or removal | 24 | 1.83 | 0.00051 | 2.20 | 0.762625317 | 0.042643325 | 0.907815417 |
| GOTERM_BP_FAT | GO:0045184~establishment of protein localization | 77 | 5.86 | 0.000579 | 1.47 | 0.804710791 | 0.046901918 | 1.030373006 |
| GOTERM_BP_FAT | GO:0016071~mRNA metabolic process | 43 | 3.27 | 0.000733 | 1.71 | 0.873455781 | 0.057351501 | 1.302308953 |
