# Supplementary material for: Extensive Evolutionary Changes in Regulatory Element Activity during Human Origins Are Associated with Altered Gene Expression and Positive Selection
Source: PLoS Genet. 2012 Jun 28;8(6):e1002789. doi: 10.1371/journal.pgen.1002789 (PMC3386175; doi:10.1371/journal.pgen.1002789)
Supplement: Table S10 — Testing for selection, P values (Two-tailed Mann-Whitney test). To test significance, for a given set of regions (Fibroblast: human DHS gain, human DHS loss, chimpanzee DHS gain, chimpanzee DHS loss, Common; LCLs: LCL human DHS gain, LCL human DHS loss, Common) we ordered the P values for selection on both hg19 and panTro2, then did a Mann-Whitney test to see if one branch has higher P values than the other. (PDF) [file pgen.1002789.s026.pdf]

**Fibroblasts**	
Chimpanzee DHS loss	0.0003289248
Chimpanzee DHS gain	0.001821841
Human DHS loss	0.0008922939
Human DHS gain	0.02283417
Common	0.936971
**LCLs**	
LCL Human DHS loss	0.951892
LCL Human DHS gain	0.9775451
LCL Common	0.3691065
