# Supplementary material for: Extensive Evolutionary Changes in Regulatory Element Activity during Human Origins Are Associated with Altered Gene Expression and Positive Selection
Source: PLoS Genet. 2012 Jun 28;8(6):e1002789. doi: 10.1371/journal.pgen.1002789 (PMC3386175; doi:10.1371/journal.pgen.1002789)
Supplement: Table S11 — The most rapidly evolving Conserved Non-coding Sequences (CNSs) minimally overlap species-specific DHS gains and losses. Human Accelerated Regions (HARs) and Chimpanzee CONDELs (cCONDELs) did not overlap any species-specific DHS gains, losses, or common regions. (a) Genome coordinates of Human Accelerated Conserved Non-coding Sequences (HACNSs) that overlapped 1 human-specific DHS loss and 1 common DHS region identified in fibroblasts. (b) Genome coordinates of Chimp Accelerated Conserved Non-coding Sequences (CACNSs) that overlapped 1 chimp-specific DHS losses and 1 common DHS region. (c) Percent of HACNSs, CACNSs, HARs, and cCONDELs that overlap the top 100 k DHS peaks (defined by p-value) from fibroblast and LCL samples in hg19 space. (d) Percent of Human CONDELs (hCONDELs) that overlap the top 100 k DHS peaks (defined by P value) from fibroblast and LCL samples in panTro2 space. (e) The number of HACNSs, CACNSs, HARs, and cCONDELs that overlap DHS sites identified in 27 diverse cell lines is consistently low. (PDF) [file pgen.1002789.s027.pdf]

a. HACNS (992 regions)- Human Accelerated Conserved Non-coding Sequences overlap with species-specific DHS gains and losses (in Hg19 space)

	chromosome	start	stop
1 hit in common(Fibro)	chr8	130697782	130698082
1 hit in human DHS loss	chr3	107725094	107725394

b. CACNS (1050 regions)- Chimp Accelerated Conserved Non-coding Sequences overlap with species-specific gains and losses (in Hg19 space)

	chromosome	start	stop
1 hit in common(Fibro)	chr2	121070842	121071142
1 hit in chimpanzee DHS loss	chr5	53279473	53279773

c. Percent of HACNS, CACNS, HARs, and cCONDELs that overlap with Top100k initial peaks (in Hg19 space)

	HACNSs (992 regions)	CACNSs (1050 regions)	HARs (202 regions)	cCONDELs (344 regions)
Fibroblasts:				
H_F_B1	4.33%	4.57%	8.42%	11.34%
H_F_B2	3.93%	4.57%	6.93%	9.88%
H_F_B3	4.44%	5.43%	5.94%	7.85%
C_F_B1	4.54%	5.24%	7.43%	0.87%
C_F_B2	5.85%	5.43%	6.93%	1.45%
C_F_B3	5.24%	5.24%	4.46%	0.29%
Q_F_B1	4.54%	4.19%	3.47%	8.72%
Q_F_B2	3.53%	4.38%	4.95%	9.59%
Q_F_B3	4.44%	4.48%	4.46%	10.17%
LCLs:				
H_L_B1	3.23%	2.76%	3.96%	10.76%
H_L_B2	2.42%	2.1%	2.97%	7.27%
H_L_B3	2.82%	2.29%	3.96%	9.3%
C_L_B1	3.33%	2.86%	4.46%	0.58%
C_L_B2	2.52%	1.81%	1.98%	0.29%
C_L_B3	1.61%	1.9%	0.99%	0.58%

d. Percent of hCONDELs that overlap with Top100k initial peaks (in panTro2 space)

	hCONDELs (583 regions)
Fibroblasts:	
C_F_B1	8.58%
C_F_B2	11.32%
C_F_B3	9.43%
LCLs:	
C_L_B1	6.17%
C_L_B2	9.78%
C_L_B3	10.98%

e. HACNS, CACNS, and HARs that overlap with ENCODE peaks (in Hg19 space)

ENCODE Cell Types	DNaseHS peaks overlapping HACNSs (992 regions)	Percent of HACNSs that overlap peaks	DNaseHS peaks overlapping CACNSs (1050 regions)	Percent of CACNSs that overlap peaks	DNaseHS peaks overlapping HARs (202 regions)	Percent of CACNSs that overlap peaks	DNaseHS peaks overlapping cCONDELS (344 regions)	Percent of cCONDELs that overlap peaks
Chorion	25	2.52%	26	2.48%	12	3.49%	26	7.56%
FB0167P	23	2.32%	31	2.95%	10	2.91%	31	9.01%
FB8470	16	1.61%	24	2.29%	7	2.03%	38	11.05%
Fibroblasts_park	17	1.71%	28	2.67%	10	2.91%	31	9.01%
GM12878	20	2.02%	17	1.62%	7	2.03%	27	7.85%
GM12891	20	2.02%	23	2.19%	11	3.2%	27	7.85%
GM12892	21	2.12%	21	2%	10	2.91%	26	7.56%
GM18507	19	1.92%	19	1.81%	6	1.74%	27	7.85%
GM19238	19	1.92%	19	1.81%	8	2.33%	24	6.98%
GM19239	23	2.32%	28	2.67%	7	2.03%	29	8.43%
GM19240	25	2.52%	18	1.71%	7	2.03%	30	8.72%
H1_ES	25	2.52%	22	2.1%	13	3.78%	23	6.69%
H9_ES	27	2.72%	24	2.29%	14	4.07%	30	8.72%
HelaS3_IFGA	23	2.32%	20	1.9%	4	1.16%	37	10.76%
HelaS3	22	2.22%	20	1.9%	3	0.87%	29	8.43%
HepG2	18	1.81%	22	2.1%	5	1.45%	25	7.27%
HUVEC	18	1.81%	24	2.29%	10	2.91%	20	5.81%
K562	22	2.22%	20	1.9%	8	2.33%	21	6.1%
MCF7	25	2.52%	34	3.24%	4	1.16%	34	9.88%
Medullo	19	1.92%	31	2.95%	15	4.36%	28	8.14%
Melanocyte	27	2.72%	47	4.48%	10	2.91%	26	7.56%
Myoblast	19	1.92%	28	2.67%	15	4.36%	21	6.1%
Myometrial	22	2.22%	36	3.43%	11	3.2%	29	8.43%
Myotube	22	2.22%	31	2.95%	16	4.65%	25	7.27%
NHEK	26	2.62%	26	2.48%	6	1.74%	33	9.59%
Pancreatic_islets	22	2.22%	22	2.1%	14	4.07%	22	6.4%
SM_SFM	20	2.02%	25	2.38%	8	2.33%	31	9.01%
