# Supplementary material for: Extensive Evolutionary Changes in Regulatory Element Activity during Human Origins Are Associated with Altered Gene Expression and Positive Selection
Source: PLoS Genet. 2012 Jun 28;8(6):e1002789. doi: 10.1371/journal.pgen.1002789 (PMC3386175; doi:10.1371/journal.pgen.1002789)
Supplement: Table S12 — Genes located closest to human-specific DHS gain/loss and common regions as well as genes identified as differentially expressed among the 3 primate species are not highly associated with genes used to identify Fibroblast biopsy locations in Rinn et al. 2006 PLoS Genetics [30]. (PDF) [file pgen.1002789.s028.pdf]

	Number of genes that intersect with Rinn 2006 PLoS Genetics 337 probes (299 unique genes)	Percentage of genes that overlap
**DHS**		
human DHS gain (836 closest genes)	20	2.4%
human DHS loss (286 closest genes)	5	1.7%
chimpanzee DHS gain (676 closest genes)	20	3%
chimpanzee DHS loss (211 closest genes)	6	2.8%
common DHS (1259 closest genes)	18	1.4%
**EXPRESSION**		
human upregulated genes (1047)	27	2.6%
human downregulated genes (881)	19	2.2%
chimpanzee upregulated genes (785)	20	2.5%
chimpanzee downregulated genes (788)	19	2.4%
commonly expressed genes (1365)	10	0.7%
