# Supplementary material for: Extensive Evolutionary Changes in Regulatory Element Activity during Human Origins Are Associated with Altered Gene Expression and Positive Selection
Source: PLoS Genet. 2012 Jun 28;8(6):e1002789. doi: 10.1371/journal.pgen.1002789 (PMC3386175; doi:10.1371/journal.pgen.1002789)
Supplement: Figure S4 — Box plot showing DNase intensity of human DHS gains/losses and common regions across 27 human cell types shown in Figure 4 heatmaps. (PDF) [file pgen.1002789.s005.pdf]

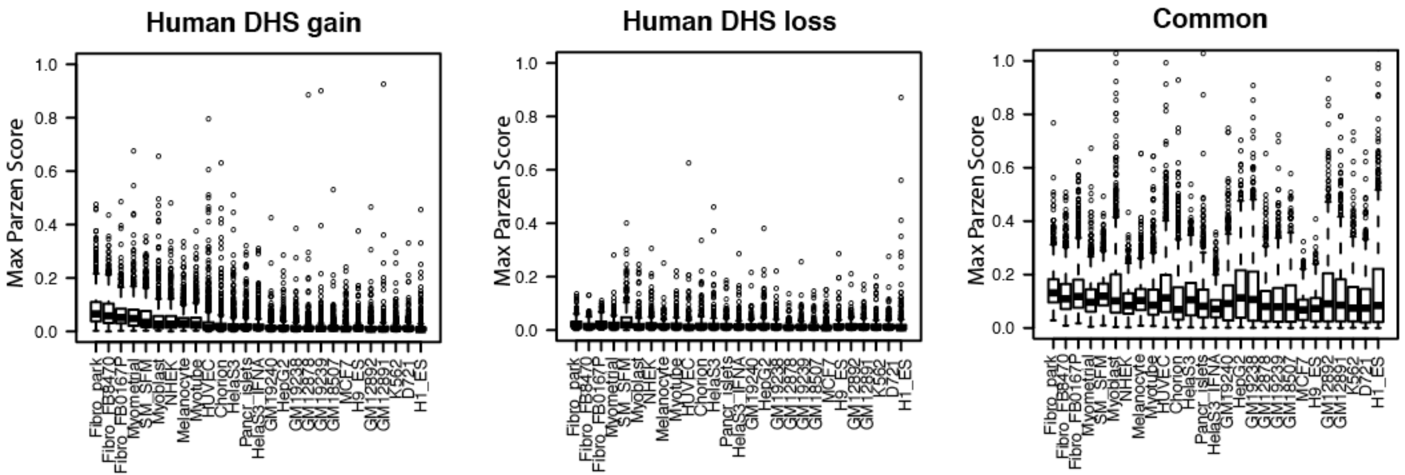

Human DHS gain
Human DHS loss
Common
Max Parzen Score
0.0
0.2
0.4
0.6
0.8
1.0
Fibro_park
Fibro_FB8470
Fibro_FB0167P
Myometrial
SM_SFM
Myoblast
NHEK
Melanocyte
Myotube
HUVEC
Chorion
HelaS3
Pancr_islets
HelaS3_IFNA
GM19240
HepG2
GM19238
GM12878
GM19239
GM18507
MCF7
H9_ES
GM12892
GM12891
K562
D721
H1_ES
